# Enantioselective Synthesis of 3,3-Disubstituted-2,3-dihydrobenzofurans by Intramolecular Heck-Matsuda/Carbonylation/Stille Coupling

**DOI:** 10.1021/acs.joc.4c02503

**Published:** 2025-06-25

**Authors:** Luiz Paulo Melchior de Oliveira Leão, Otto Daolio Köster, Leonardo José Duarte, Ataualpa Albert Carmo Braga, Carlos Roque Duarte Correia

**Affiliations:** ‡ Chemistry Institute, State University of Campinas, Unicamp, 13083-970 Campinas, São Paulo, Brazil; † Department of Fundamental Chemistry, Institute of Chemistry, 28133 University of São Paulo, USP, 05508-900 São Paulo, São Paulo, Brazil

## Abstract

The enantioselective one-pot synthesis of 3,3-disubstituted-2,3-dihydrobenzofuran was developed via a strategy involving a palladium-catalyzed Heck-Matsuda reaction, followed by subsequent carbonylation and/or an organotin transmetalation step employing chiral *N*,*N* ligands. The one-pot reaction requires mild conditions and tolerates a wide range of functional groups. This method provides straightforward access to a diverse array of enantioenriched dihydrobenzofurans bearing a ketone or an alkyl side chain adjacent to the generated quaternary stereogenic center in yields up to 91% and er up to 99:1.

## Graphical abstract

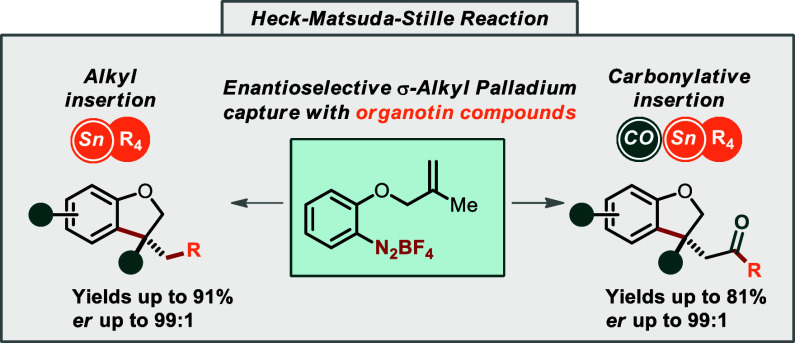

## Introduction

Since its initial report in 1969, the Heck reaction has evolved significantly. It has shown remarkable versatility transitioning from its earlier reports using aryl mercuric halides^1^ and aryl halides^2^ to the use of arenediazonium salts, the so-called Heck-Matsuda reaction.^3^ Due to its robustness and the broad scope of reagents and substrates, the Heck reaction has become a valuable tool for the synthesis of complex molecules.^4^

Although the construction of C–C bond via enantioselective Heck-Matsuda reactions have already been disclosed and refined throughout the past decade,^5^ its intramolecular version remains underexplored with few examples in the literature.^6,7^ In this regard, the development of the enantioselective intramolecular Heck-Matsuda reaction constitutes a powerful strategy for the efficient synthesis of complex frameworks bearing stereogenic centers. In particular, the dihydrobenzofuran motif is prevalent in numerous natural products and pharmaceuticals^8^ (Figure 1, top column and upper left). Its synthesis by a cyclization protocol involving a palladium-catalyzed Heck reaction of 2-iodophenol allyl ethers or its reductive variant has been extensively investigated.^9^ A previous approach has been explored by us making use of arenediazonium tetrafluoroborates to access this structural motif.^6a,10^ By this approach, the key alkyl palladium intermediate is intercepted with a diverse array of coupling reagents, including olefins,^6b^ boronic acids,^11^ carbon monoxide,^12^ halides,^13^ and by C–H activation,^14^ enhancing its potential for the synthesis of important heterocyclic compounds. In 1988, Grigg and co-workers disclosed a palladium-catalyzed tandem cyclization-anion captured with organotin compounds using aryl halides to synthesize dihydroindoles (Figure 1a).^15a^ However, despite reports on the coupling of arenediazonium salts with organotin compounds,^15^ to the best of our knowledge, there are no reports of a combined, one-pot enantioselective procedure involving a tandem Heck-Matsuda-Stille coupling (Figure 1c).

**Figure 1:**
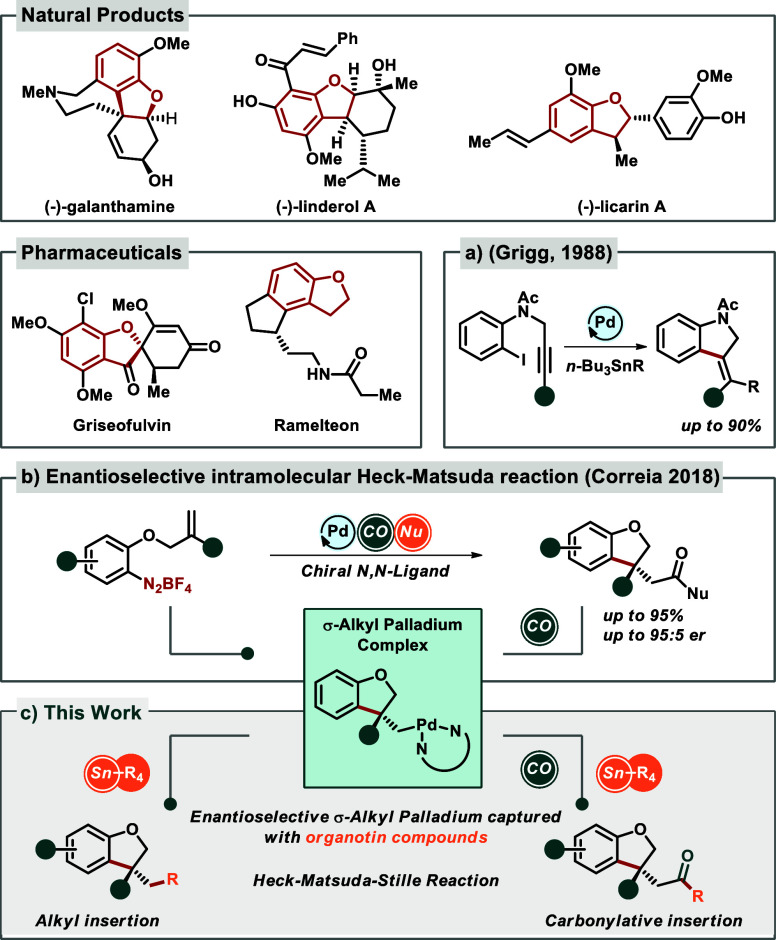
Importance for dihydrobenzofuran motif in natural products and pharmaceuticals. (a) σ-Alkyl palladium complex captured by organotin compounds (Griggs). (b) First example of the intermolecular enantioselective Heck-Matsuda reaction. (c) The first example of the enantioselective Heck-Matuda-Stille-Kosugi coupling.

Building upon our previous studies, we present herein the intramolecular enantioselective carbonylative and noncarbonylative Heck-Matsuda reaction coupled to organotin compounds employing chiral *N,N*-ligands. This method facilitates the synthesis of dihydrobenzofuran bearing either a ketone or an alkyl side chain adjacent to a quaternary stereogenic center in a single step under mild conditions. The successful integration of organotins significantly broadens the scope of possible functionalizations due to their vast structural diversity and stability under the required reaction conditions.

## Results and Discussion

Aiming at finding the best reaction conditions, different combinations of solvents and additives were assessed (Table 1). Polar solvents such as methanol, DMF, and acetone gave superior yields while maintaining excellent enantioselectivities. However, small amounts of methyl ester byproduct, resulting from methanol addition to the acyl-Pd intermediate, were observed when methanol was used as the solvent. Interestingly, acetonitrile as the solvent led to a decrease in enantioselectivity (Table 1, entry 11), possibly due to the decomplexation of the *N,N*-ligand from the palladium catalyst.

**Table 1:**
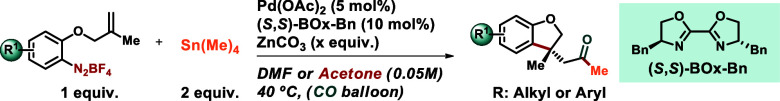
Optimization of the Heck-Matsuda-Stille Reaction^a^

entry	solvent	additive (equiv)	yield (%)	er
1	MeOH		62 (10)^b^	90:10
2	acetone		61	95:5
3	DMF		90 (78)^c^	99:1
4	acetone	ZnCO_3_ (0.1 equiv)	62	94:6
5	acetone	ZnCO_3_ (0.5 equiv)	87 (81)^c^	95:5
6	acetone	ZnCO_3_ (1 equiv)	60	95:5
7	DMF	ZnCO_3_ (0.5 equiv)	87	98:2
8	MeOH	ZnCO_3_ (0.5 equiv)	67	95:5
9	toluene	ZnCO_3_ (0.5 equiv)	<5	
10	THF	ZnCO_3_ (0.5 equiv)	79	93:7
11	MeCN	ZnCO_3_ (0.5 equiv)	73	80:20
12	1,4-dioxane	ZnCO_3_ (1 equiv)	<5	

a Reaction conditions: **1** (0.1 mmol), Pd­(OAc)_2_ (5 mol %), ligand 2,2′-bis­[(4*S*)-4-benzyl-2-oxazoline] (10 mol %), 40 °C, 6 h. Yield determined by ^1^H NMR using 1,3-bis­(trifluoromethyl)-5-bromobenzene as internal standard. Enantiomeric ratios were determined by HPLC using chiral columns.

b 10% of the side product methyl ester.

c Isolated yield.

Prior studies have suggested that some Lewis acids can exert a beneficial effect on the cross-coupling of arenediazonium salts in polar solvents.^22^ Thus, we investigated ZnCO_3_ as a potential additive in our system. Our experimental findings confirm that ZnCO_3_ enhances the reaction in acetone, although its precise mechanism of action remains unclear. Ideal conditions were achieved using 0.5 equiv of ZnCO_3_ resulting in higher yields while maintaining excellent enantiomeric ratios. Somewhat surprisingly, for reactions carried out in DMF, the addition of ZnCO_3_ offer no advantage in terms of overall outcome (entries 3 and 7).

In this context, optimal results were obtained using DMF or acetone as the solvent, 5 mol % of Pd­(OAc)_2_ and 10 mol % of ligand 2,2′-Bis­[(4*S*)-4-benzyl-2-oxazoline] (BOx-Bn). When acetone was used, 0.5 equiv of ZnCO_3_ were added. With optimal conditions in hand, we started to evaluate the scope of the reaction testing the electronic nature of the arenediazonium salts, the size of the resulting heterocycle and the nature of the organotin compound.

The monosubstituted dihydrobenzofuran ketones bearing 6-Me **1b** and 5-OMe **1c** were obtained in good yields (64 and 70%, respectively) and in high enantiomeric ratios (99:1 for both). The electron-withdrawing monosubstituted arenediazonium salts bearing a 7-Br or a 5-NO_2_ afforded their corresponding products **1d** and **1e** in excellent er in DMF (97:3 for both) and with 18 and 35% yield, respectively. It is worth mentioning that in the presence of CO, palladium catalyzes the reduction of nitroarenes leading to the formation of byproducts.^16^ In addition, we observed that arenediazonium salts bearing halides provided lower yields in DMF. In this regard, acetone served as an alternative solvent of choice enhancing the yields of these products (55 and 48% respectively), while preserving the high enantiomeric ratios. The dichlorobenzenediazonium salt afforded the product **1f** in 48% yield in 94:6 er in acetone and 10% yield in DMF. The preparation of a larger ring was successfully achieved for the 2,2,3,3-dihydrobenzopyran **1g** and good yields were obtained in both solvents (57 and 59%), but this sixmembered ring shows a decreasing enantioenrichment, with higher er in DMF (79:21 er) when compared to acetone (54:46 er).

With the encouraging results with tetramethyltin, we decided to explore more tin reagents. In general, the reactions with *n-*tetrabutyltin were less effective than with tretramethyltin. The one-pot reactions in DMF furnished ketobenzofurans **1h** and **1i** in yields of 35% and 25%, respectively, with an excellent er (98:2 er in both cases). The use of tetra *n*-butyl tin compound was challenging under these reaction conditions because of the potential β-elimination from the Pd-alkyl intermediate. Benzofuran **1j** was obtained in 21% yield and 85:15 er in acetone and only in trace amounts in DMF. Curiously, similar results were previously observed by Matsuda when coupling arenediazonium salts to tetraethyl or *n-*tetrabutyltin compounds.^15c^ Given that tetramethyl- and tetraphenyltin compounds are not susceptible to this decomposition pathway, we extended our investigation to include tetraphenyltin. Regarding tetraphenyl tin, the benzofuran products **1k**, **1l**, **1m** were prepared in yields and er comparable to those obtained with tetramethyl tin with a highlight to the excellent er of 99:1, 98:2 and 97:3 when the one-pot reaction was carried out in DMF.

The key issue of the absolute stereochemistry of the benzofurans **1a** to **1o**, as *R* was determined by converting methyl ketone **1a** into the known ester **1p** as described Scheme 1 and comparing its specific rotation ([α]_D_^25^ + 19 (c 1.00, CHCl_3_, er 95:5)) with that reported in the literature,^6a^ and by HPLC coinjection with an authentic sample (see Support Information for details).

**Scheme 1:**
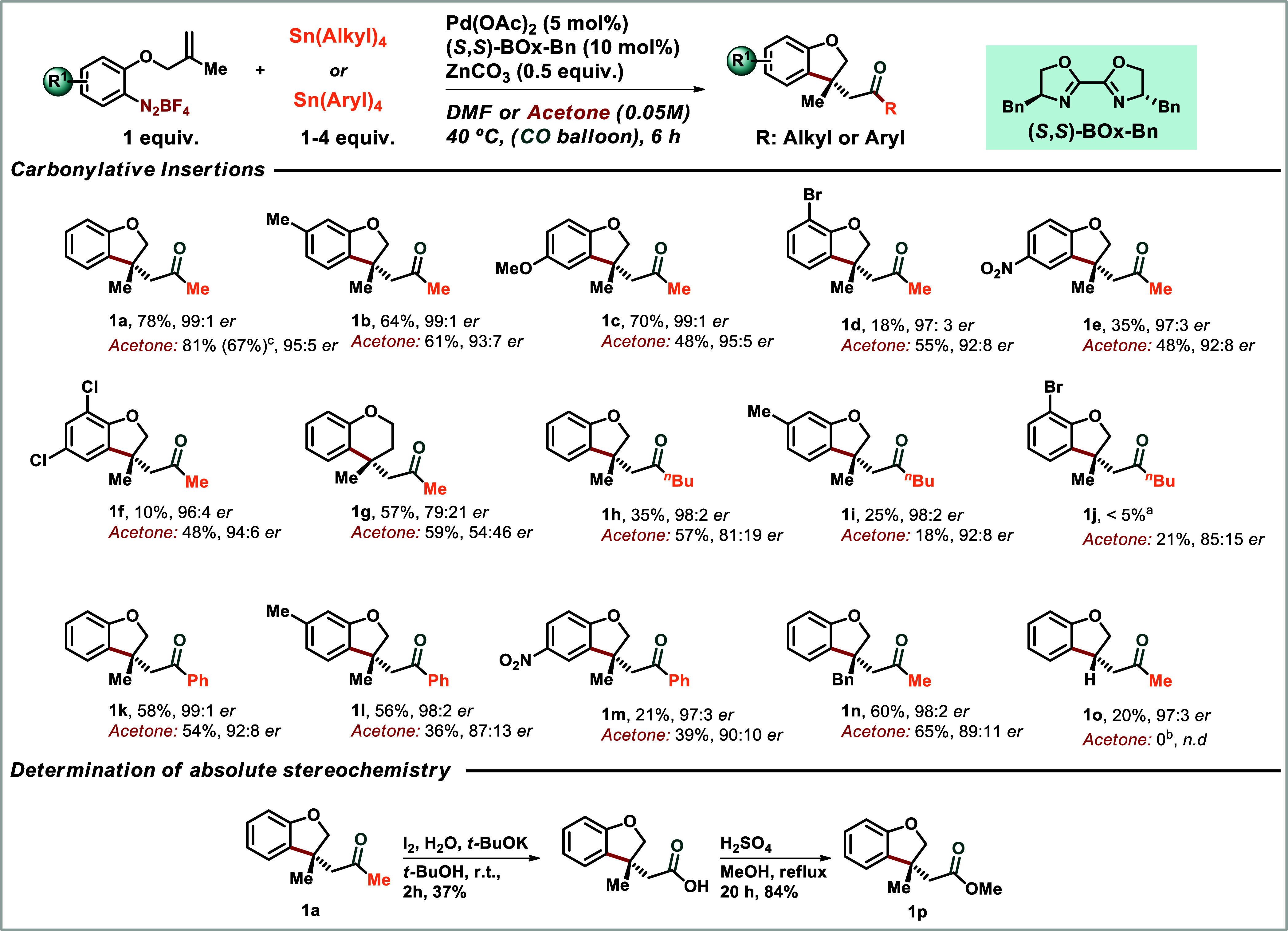
Scope of the Enantioselective Heck-Matsuda-Stille Carbonylative Reaction

The reactions were performed on a 0.1 mmol scale. Enantiomeric ratios were determined by chiral HPLC using chiral columns. The absolute stereochemistry was assigned based on compound 1a, which was derivatized and compared to a compound with known absolute configuration (see Supporting Information).
a Yields determined by ^1^H NMR using 1,3-bis­(trifluoromethyl)-5-bromobenzene as internal standard.
b 3-Methyl benzofuran as the sole product.
c Yield for the reaction performed in 1 mmol scale.

For the Heck-Matsuda-Stille coupling reactions leading to the benzofuran products **1d**, **1e**, and **1k**, we also observed byproducts resulting from the direct alkyl coupling of the Pd-alkyl intermediate with the organotin compound before the carbonylation step (Table S2, Support information). This finding prompted us to further investigate this reaction aiming at adding diversity to this one-pot protocol. As shown in Scheme 2, the Heck-Stille products were obtained in a good range of yields (39–91%) and moderate to good enantioselectivities (64:36–93:7 er) when acetone was used as the solvent despite their higher volatility when compared to their carbonylated counterparts. The same trend observed in the carbonylative Heck-Stille protocol was observed in the direct Heck-Stille alkylation: excellent er (95:5–99:1) and lower yields in DMF. For example: the Heck-Stille products **2a** and **2b** were obtained with reasonably good yields of 47 and 62%, in good er of 87:13 and 89:11, respectively, in acetone, with notably lower yields in DMF. On the other hand, the Heck-Stille products **2c**, **2d**, **2e** and **2f** were obtained in good yields (47–91% yield) in excellent er (95:5–98:2 er) in DMF. As observed before, the aryl bromide and the nitro aryl substrates led to more complex mixtures with benzofurans **2g** and **2h** obtained in low yields when reactions were carried out in DMF. However, in acetone, arylbenzofuran **2g** was obtained in a good yield of 74% in 64:36 er, while **2h** was obtained in 39% in 93:7 er. The assignment of the absolute stereochemistry of enantioselective Heck-Stille couplings as *R* was established by comparison of HPLC retention times with a sample of known absolute configuration^6a^ (see Support Information for details).

**Scheme 2:**
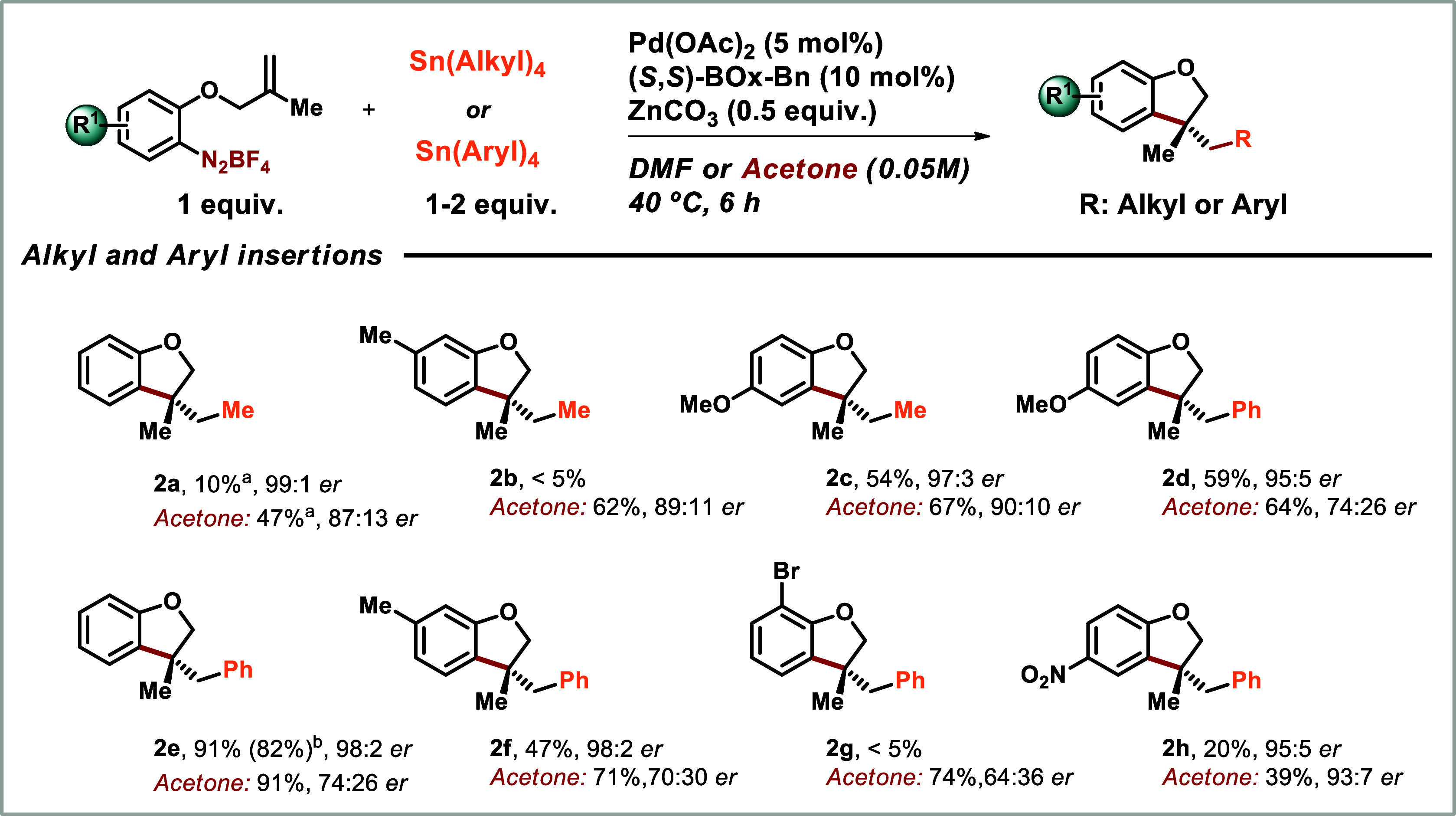
Scope of the Heck-Matsuda-Stille Alkylation

The reactions were performed on a 0.1 mmol scale. Enantiomeric ratios were determined by chiral HPLC using chiral columns. The absolute stereochemistry was assigned based on compound 2e, which was compared to a compound with known absolute configuration (see Supporting Information).
a Volatile compound, yield determined by ^1^H NMR using 1,3-bis­(trifluoromethyl)-5-bromobenzene as internal standard.
b Yield for the reaction performed in 1 mmol scale.

The mechanism of the Stille-Kosugi coupling remains a matter of debate and its combination with the Heck cycle adds one more layer of complexity to the process.^17a–17d^ Moreover, in the proposed Heck-Matsuda mechanism, a vacant coordination sphere of the Pd­(II)^+^ species is formed after migratory insertion, which can rapidly be transformed to a solvato complex [PdL_2_R^1^(S)]^+^ through solvent association (Scheme 3). Additionally, because of the isolation of SnPh_3_F from the reaction when using SnPh_4_ as a coupling partner (See Support Information), we hypothesize that a fluoride anion probably plays a critical role in the transmetalation step in this Heck-Matsuda-Stille coupling, which is similar to the one described by Jutand and co-workers^18^ and also observed in the Hiyama^19^ and Suzuki couplings.^20,21^ To support the proposed mechanism depicted in Scheme 3, exploratory DFT calculations were carried out at the B3LYP-D3 def2-SVP level of theory. Optimized structures and transitions states are provided in the Supporting Information.

**Scheme 3:**
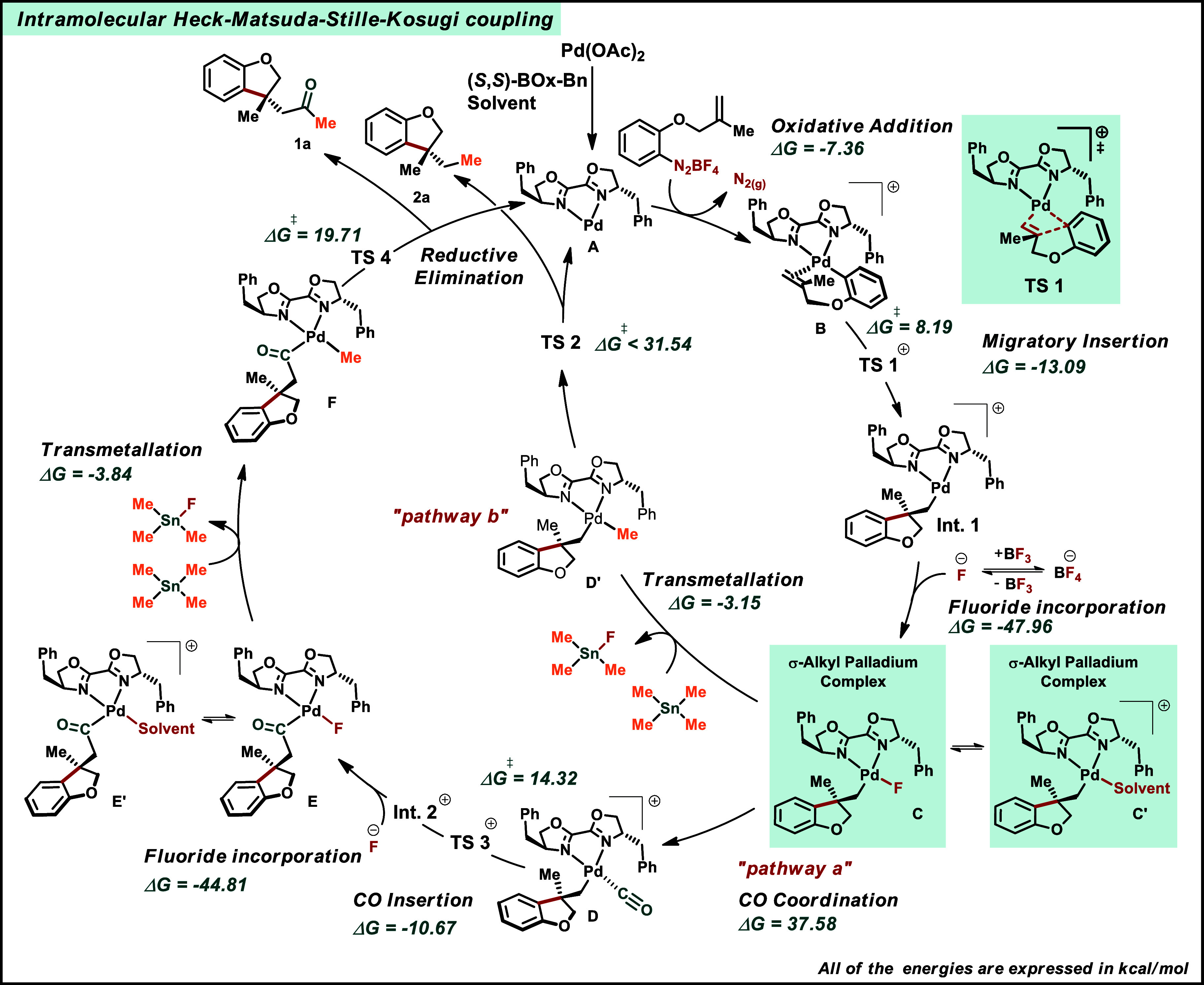
Proposed Mechanism

The reaction begins with the formation of the Pd-catalyst with *N*,*N*-chiral ligand in the solvent system, yielding intermediate **A** (Scheme 3). However, to properly describe the square-planar arrangement of Pd, the structure **A** needs to account for interactions with one solvent molecule. Additionally, it is highly likely that Pd will not have vacant coordination sites in a solvent capable of readily coordinating to the metal center. When only one molecule of the ligand is added to the Pd, the complex becomes monocoordinated. Following oxidative addition to the arenediazonium salt, intermediate **B** is formed providing the key intermediate alkyl palladium after migratory insertion. We identified two distinct conformers of intermediate B, referred to as *Si*-B and *Re*-B. These names correspond to the face of the olefin coordinated to the palladium center, which determines whether the *R* or *S* enantiomer will form following the migratory insertion step. Looking at the molar free energy of *Si*-TS1 and *Re*-TS1, we find that the former is lower in energy by ΔΔ*G* ^‡^ = 3.6 kcal/mol (Figure 2). According to the Curtin-Hammet principle,^23^ and considering that *Re*-B and *Si*-B are in equilibrium, such ΔΔ*G* ^‡^ will result in an enantiomeric ratio over 99:1 for the R product at 313 K, close to the empirically observed value of 95:5.

**Figure 2:**
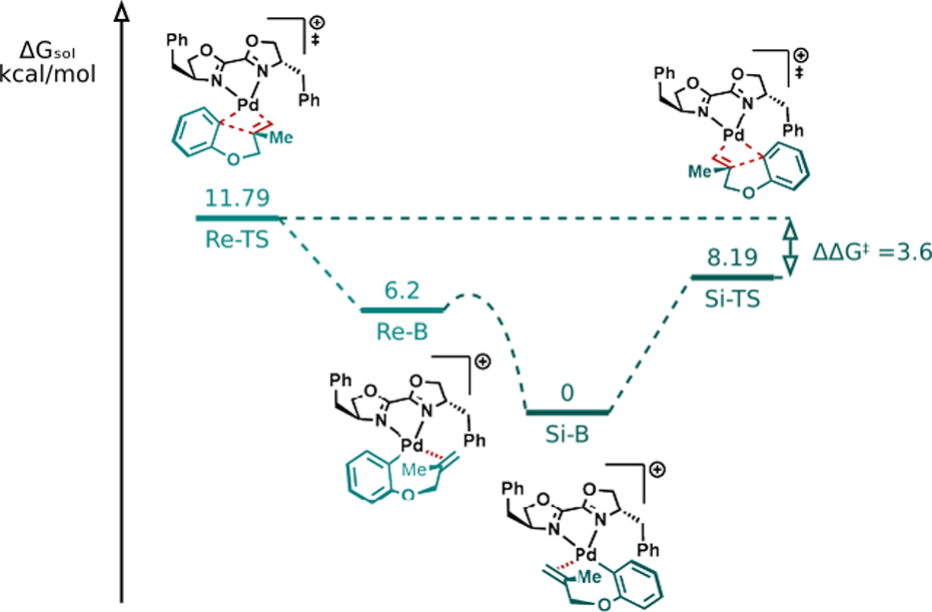
Potential energy surface of the enantiodetermining step.

The migratory insertion occurs with a free energy barrier of 8.19 kcal/mol, which is easily surpassed by the thermal energy of the system. The free energy change, Δ*G*, for this step is −13.09 kcal/mol. Subsequently, either a solvent molecule or a fluoride ion (originating from the dissociation or hydrolysis of the tetrafluoroborate anion)^17,18,20^ is then incorporated into the palladium coordination sphere, furnishing intermediate **C** (or **C′**). The whole step is exergonic with Δ*G* = −47.96 kcal/mol for fluoride incorporation. From this point, the reaction can proceed through two possible pathways, depending on the conditions applied. In the presence of CO, the “pathway a” leads to the formation of the CO-Pd complex **D.** The incorporation of CO in the coordination sphere is the most endergonic step in the catalytic cycle, with Δ*G* = 37.58 kcal/mol, **D** is then converted into the acyl palladium **E** with a free energy barrier of 14.32 kcal/mol after CO insertion and fluoride incorporation. The transmetalation step takes place with the tetraorganotin compound transferring an alkyl or aryl species to the palladium coordination sphere, forming intermediate **F**. The free energy change for the transmetalation step is small: Δ*G* = −3.84 kcal/mol for “pathway a” and Δ*G* = −3.15 kcal/mol for “pathway b”. However, the mechanism of this step is complex, as the transmetalation cannot occur in a single step. A detailed investigation of this process would require a dedicated study, which we intend to pursue in future work. Therefore, the corresponding energy barrier for this species will not be disclosed here. Following reductive elimination, the formation of product **1a** restores the catalyst to the cycle, considering the activation of the catalyst. This step is highly exergonic with Δ*G* = −78.36 kcal/mol considering the reactivation of the catalyst (See Support Information). In the absence of CO, an alternative “pathway b” is proposed, in which transmetalation occurs between intermediate **C** and the tetraorganotin compound generating intermediate **D′** leading to the formation of alkyl product **2a** via reductive elimination. However, unlike “pathway a”, this step is not straightforward. The absence of an sp^2^ carbon to coordinate with Pd after reductive elimination causes the Pd center to lose coordination with one *N*-terminal of the ligand. Alternatively, a solvent molecule can enter the coordination sphere to restore the bidentate coordination. The energy barrier for this step, considering the monodentate mechanism, is Δ*G* ^‡^ = 31.54 kcal/mol. However, this should be taken as an upper limit since this reaction may involve additional steps in which the coordination of solvent molecules will lead to a mechanism which is similar to “pathway a”. In this case, the free energy change is Δ*G* = −67.76 kcal/mol indicating that both pathways are energetically competitive.

## Conclusions

In summary, we described herein an efficient synthetic method combining the enantioselective intramolecular Heck-Matsuda reaction with an in situ carbonylative step and a Stille-Kosugi cross-coupling reaction. This method is effective in providing alkyl and aryl benzofurans ketones or alkyl and aryl benzofurans bearing a quaternary center in good yields (up to 91%) and excellent enantiomeric ratios (up to 99:1).

## Experimental Section

### General Procedure for In-Tandem Heck-Matsuda-Stille Coupling for Carbonylated Products in DMF (GP1)

To a 4 mL vial containing a magnetic stir bar, it was added Pd­(OAc)_2_ (5 mol %, 1.2 mg), the chiral *N*,*N*-ligand 2,2′-Bis­[(4*S*)-4-benzyl-2-oxazoline] (10 mol %, 3.2 mg), 2 mL of DMF, and the reaction was left stirring for 10 min at 40 °C using an aluminum heating block. After the precatalyst activation, the respective reactants were added in the following order: the arenediazonium salt (1 equiv) and the organotin compound (2 equiv, 28 μL, for SnMe_4_; 4 equiv, 132 μL for SnBu_4_, and 1 equiv for SnPh_4_, 42.7 mg). The vial was then sealed with a holed screw cap containing a PTFE septum, and CO was gently bubbled into the solution for 15 s (see Note 1 in the SI). The outlet needle was then removed, and the reaction was left stirring for 6 h at 40 °C. Next, the crude was diluted with distilled H_2_O, extracted with EtOAc (3 × 10 mL), washed with brine (3 × 10 mL), dried over anhydrous NaSO_4_, filtered, and concentrated under reduced pressure. The crude was filtered through a 10 cm silica-gel pad with EtOAc as eluent and purified by flash chromatography to furnish the carbonylated dihydrofuran products.

### General Procedure for *In-Tandem* Heck-Matsuda-Stille Coupling for Carbonylated Products in Acetone (GP2)

To a 4 mL vial containing a magnetic stir bar, it was added Pd­(OAc)_2_ (5 mol %, 1.2 mg), the chiral *N*,*N*-ligand 2,2′-Bis­[(4*S*)-4-benzyl-2-oxazoline] (10 mol %, 3.2 mg), 2 mL of acetone and the reaction was left stirring for 10 min at 40 °C using an aluminum heating block. After the precatalyst activation, the respective reactants were added in the following order: ZnCO_3_ (0.5 equiv, 6.5 mg), the arenediazonium salt (1 equiv) and the organotin compound (2 equiv, 28 μL, for SnMe_4_; 4 equiv, 132 μL for SnBu_4_, and 1 equiv for SnPh_4_, 42.7 mg). The vial was then sealed with a holed screw cap containing a PTFE septum and CO was gently bubbled into the solution for 15 s (see Note 1 in the SI). The outlet needle was then removed and the reaction was left stirring for 6 h at 40 °C. The crude was filtered through a 10 cm silica-gel pad using EtOAc as eluent and purified by flash chromatography to furnish the carbonylated dihydrofuran products.

### General Procedure for *In-Tandem* Heck-Matsuda-Stille Coupling for Direct Alkylation in DMF (GP3)

To a 4 mL vial containing a magnetic stir bar, it was added Pd­(OAc)_2_ (5 mol %, 1.2 mg), the chiral *N*,*N*-ligand 2,2′-Bis­[(4*S*)-4-benzyl-2-oxazoline] (10 mol %, 3.2 mg), 2 mL of DMF and the reaction left stirring for 10 min at 40 °C using an aluminum heating block. After the precatalyst activation the respective reactants were added in the following order: the arenediazonium salt (1 equiv), and the organotin compound (2 equiv for SnMe_4_; 4 equiv for SnBu_4_ and 1 equiv for SnPh_4_). The vial was then sealed and the reaction was left stirring for 6 h at 40 °C. Next, the crude was diluted with distilled H_2_O, extracted with EtOAc (3 × 10 mL), washed with brine (3 × 10 mL) dried over anhydrous NaSO_4,_ filtered, and concentrated under reduced pressure. The residue was filtered through a 10 cm silica-gel pad with EtOAc as eluent, and the crude was purified by flash chromatography to furnish the alkylated products.

### General Procedure for *In-Tandem* Heck-Matsuda-Stille Coupling for Direct Alkylation in Acetone (GP4)

To a 4 mL vial containing a magnetic stir bar, it was added Pd­(OAc)_2_ (5 mol %, 1.2 mg), the chiral *N*,*N*-ligand 2,2′-Bis­[(4*S*)-4-benzyl-2-oxazoline] (10 mol %, 3.2 mg), 2 mL of acetone and the reaction was left stirring for 10 min at 40 °C using an aluminum heating block. After the precatalyst activation, the respective reactants were added in the following order: ZnCO_3_ (0.5 equiv, 6.5 mg), the arenediazonium salt (1 equiv), and the organotin compound (2 equiv for SnMe_4_ and 1 equiv for SnPh_4_). The vial was then sealed and the reaction was left stirring for 6 h at 40 °C. The crude was filtered through a 10 cm silica-gel pad using EtOAc as eluent and purified by flash chromatography to furnish the alkylated products.

### Characterization of the Heck-Matsuda-Stille Products

#### (R)-1-(3-Methyl-2,3-dihydrobenzofuran-3-yl)­propan-2-one (**1a**)

The desired product was isolated by flash chromatography with 10% EtOAc in hexanes as eluent and was obtained as a colorless oil. The enantiomeric ratio was determined by HPLC analysis using the following parameters: Daicel Chiralcel IC column (4.6 mm × 250 mm): 10% iPrOH in Hexane (1.0 mL/min) as mobile phase at 25 °C (rt = 16.5 min (minor), 18.4 min (major)). **GP1:** 78% yield (14.8 mg), 99:1 er; [α]_D_^25^ + 64 (c 1.0, CHCl_3_). **GP2:** 81% yield (15.4 mg), 95:5 er; [α]_D_^25^ + 35 (c 0.5, CHCl_3_); 1 mmol scale: 67% (127.4 mg). ^**1**^ **H NMR** (500 MHz, CDCl_3_) δ 7.14 (td, *J* = 7.9, 1.4 Hz, 1H), 7.10 (dd, *J* = 7.4, 0.9 Hz, 1H), 6.88 (td, *J* = 7.4, 0.8 Hz, 1H), 6.80 (d, *J* = 8.0 Hz, 1H), 4.46 (d, *J* = 9.3 Hz, 1H), 4.41 (d, *J* = 9.3 Hz, 1H), 2.95 (d, *J* = 17.3 Hz, 1H), 2.76 (d, *J* = 17.3 Hz, 1H), 2.09 (s, 3H), 1.41 (s, 3H). ^**13**^ **C­{** ^**1**^ **H} NMR** (126 MHz, CDCl_3_) δ 206.9, 159.1, 135.0, 128.6, 122.8, 120.7, 110.0, 82.6, 53.0, 43.7, 31.2, 25.3. **HRMS (ESI-Q-Orbitrap)** *m* **/** *z*: **[M + H]^+^** calcd for C_12_H_15_O_2_: 191.1066. Found: 191.1065.

#### (*R*)-1-(3,6-Dimethyl-2,3-dihydrobenzofuran-3-yl)­propan-2-one (**1b**)

The desired product was isolated by flash chromatography with 10% EtOAc in hexanes as eluent and was obtained as a colorless oil. The enantiomeric ratio was determined by HPLC analysis using the following parameters: Daicel Chiralcel IC column (4.6 mm × 250 mm): 5% iPrOH in Hexane (1.0 mL/min) as mobile phase at 25 °C (rt = 7.2 min (minor), 8.3 min (major)). **GP1:** 64% yield (13.1 mg), 99:1; [α]_D_^25^ + 115 (c 1.00, CHCl_3_). **GP2:** 61% yield (12.4 mg), 93:7 er; [α]_D_^25^ + 63 (c 1.00, CHCl_3_). ^**1**^ **H NMR** (250 MHz, CDCl_3_) δ 6.98 (d, *J* = 7.5 Hz, 1H), 6.69 (d, *J* = 7.5 Hz, 1H), 6.62 (s, 1H), 4.45 (d, *J* = 9.3 Hz, 1H), 4.38 (d, *J* = 9.3 Hz, 1H), 2.93 (d, *J* = 17.2 Hz, 1H), 2.72 (d, *J* = 17.2 Hz, 1H), 2.30 (s, 3H), 2.09 (s, 3H), 1.39 (s, 3H). ^**13**^ **C­{** ^**1**^ **H} NMR** (75 MHz, CDCl_3_) δ 208.9, 155.5, 131.6, 124.8, 119.0, 115.1, 88.3, 58.4, 51.7, 39.2, 33.4, 31.8, 21.3. **HRMS (ESI-Q-Orbitrap)** *m* **/** *z*: [M + H]^+^ calcd for C_13_H_17_O_2_: 205.1228. Found: 205.1227.

#### (*R*)-1-(5-Methoxy-3-methyl-2,3-dihydrobenzofuran-3-yl)­propan-2-one (**1c**)

The desired product was isolated by flash chromatography with 10% EtOAc in hexanes as eluent and was obtained as a yellowish oil. **R_f_** in 10% of EtOAc: 0.16. The enantiomeric ratio was determined by HPLC analysis using the following parameters: Daicel Chiralcel OJ-3 column (4.6 mm × 250 mm): 5% iPrOH in Hexane (1.0 mL/min) as mobile phase at 30 °C (rt = 14.7 min (major), 18.9 min (minor)). **GP1:** 70% yield (15.3 mg), 99:1 er; [α]_D_^25^ + 66 (c 2.00, CHCl_3_). **GP2:** 48% yield (10.6 mg), 95:5 er; [α]_D_^25^ + 65 (c 2.00, CHCl_3_). ^**1**^ **H NMR** (500 MHz, CDCl_3_) δ 6.73–6.65 (m, 3H), 4.43 (d, *J* = 9.3 Hz, 1H), 4.38 (d, *J* = 9.9 Hz, 1H), 3.77 (s, 3H), 2.92 (d, *J* = 17.3 Hz, 1H), 2.75 (d, *J* = 17.3 Hz, 1H), 2.10 (s, 3H), 1.40 (s, 3H). ^**13**^ **C­{** ^**1**^ **H} NMR** (126 MHz, CDCl_3_) δ 206.7, 154.3, 153.0, 135.9, 113.1, 109.8, 109.3, 82.7, 56.1, 52.7, 44.1, 31.1, 24.8. **HRMS (ESI-Q-Orbitrap)** *m* **/** *z*: [M + H]^+^ calcd for C_13_H_17_O_3_: 221.1172. Found: 221.1168.

#### (*R*)-1-(7-Bromo-3-methyl-2,3-dihydrobenzofuran-3-yl)­propan-2-one (**1d**)

The product was isolated by flash column chromatography (2 to 20% of EtOAc in hexanes as eluent) and was obtained as a colorless oil. **R_f_** in 8% of EtOAc in hexanes: 0.17. The enantiomeric ratio was determined by HPLC analysis using the following parameters: Daicel Chiralcel OJ-3 column (4.6 mm × 250 mm): 5% iPrOH in Hexane (1.0 mL/min) as mobile phase at 30 °C (rt = 13.4 min (major), 16.9 min (minor)). **GP1:** 18% yield (4,7 mg), 97:3 er; [α]_D_^25^ + 7 (c 1.00, CHCl_3_).**GP2:** 55% yield (14.7 mg), 92:8 er; [α]_D_^25^ + 22 (c 2.00, CHCl_3_).^**1**^ **H NMR** (600 MHz, CDCl_3_) δ 7.29 (dd, *J* = 8.0, 1.2 Hz, 1H), 7.03 (dd, *J* = 7.4, 1.1 Hz, 1H), 6.79–6.73 (m, 1H), 4.54 (d, *J* = 9.4 Hz, 1H), 4.52 (d, *J* = 9.9 Hz, 1H), 2.96 (d, *J* = 17.7 Hz, 1H), 2.78 (d, *J* = 17.7 Hz, 1H), 2.12 (s, 3H), 1.42 (s, 3H). ^**13**^ **C­{** ^**1**^ **H} NMR** (151 MHz, CDCl_3_) δ 206.2, 156.2, 136.3, 131.5, 122.1, 121.8, 103.0, 83.0, 52.7, 44.7, 31.0, 25.2. **HRMS (ESI-Q-Orbitrap)** *m* **/** *z*: [M + H]^+^ calcd for C_12_H_14_BrO_2_: 269.0171. Found: 269.0167.

#### (*R*)-1-(3-Methyl-5-nitro-2,3-dihydrobenzofuran-3-yl)­propan-2-one (**1e**)

The desired product was isolated by flash chromatography with 10% EtOAc in hexanes as eluent and was obtained as a colorless oil. The enantiomeric ratio was determined by HPLC analysis using the following parameters: Daicel Chiralcel OJ-3 column (4.6 mm × 250 mm): 20% iPrOH in Hexane (1.0 mL/min) as mobile phase at 25 °C (rt = 21.7 min (minor), 25.9 min (major)). **GP1:** 35% yield (8.3 mg), 97:3 er; [α]_D_^25^ + 51 (c 1.00, CHCl_3_). **GP2:** 48% yield (11.3 mg), 92:8 er; [α]_D_^25^ + 37 (c 1.00, CHCl_3_). ^**1**^ **H NMR** (500 MHz, CDCl_3_) δ 8.12 (dd, *J* = 8.8, 2.4 Hz, 1H), 8.00 (d, *J* = 2.4 Hz, 1H), 6.84 (d, *J* = 8.8 Hz, 1H), 4.62 (s, 2H), 3.05 (d, *J* = 18.0 Hz, 1H), 2.83 (d, *J* = 18.0 Hz, 1H), 2.16 (s, 3H), 1.44 (s, 3H). ^**13**^ **C­{** ^**1**^ **H} NMR** (126 MHz, CDCl_3_) δ 205.9, 164.7, 142.2, 136.6, 126.2, 119.5, 110.0, 84.6, 52.6, 43.2, 31.0, 26.0. **HRMS (ESI-Q-Orbitrap)** *m* **/** *z*: [M + H]^+^ calcd for C_12_H_14_NO_4_: 236.0922. Found: 236.0925.

#### (*R*)-1-(5,7-Dichloro-3-methyl-2,3-dihydrobenzofuran-3-yl)­propan-2-one (**1f**)

The desired product was isolated by flash column chromatography (2 to 20% of EtOAc in hexanes as eluent) and was obtained as a yellow oil. **R_f_** in 7% EtOAc: 0.26. The enantiomeric ratio was determined by HPLC analysis using the following parameters: Daicel Chiralcel OJ-3 column (4.6 mm × 250 mm): 5% i-PrOH in Hexane (1.0 mL/min) as mobile phase at 30 °C (rt = 9.219 min (major), 10.506 min (minor)). **GP1:** 10% yield (2.6 mg), 96:4 er; [α]_D_^25^ + 1 (c 0.5, CHCl_3_). **GP2:** 48% yield (12.3 mg), 94:6 er; [α]_D_^25^ + 35 (c 2.00, CHCl_3_). ^**1**^ **H NMR** (600 MHz, CDCl_3_) δ 7.15 (d, *J* = 2.1 Hz, 1H), 6.96 (d, *J* = 2.1 Hz, 1H), 4.55 (s, 2H), 2.95 (d, *J* = 17.9 Hz, 1H), 2.78 (d, *J* = 17.9 Hz, 1H), 2.14 (s, 3H), 1.41 (s, 3H). ^**13**^ **C­{** ^**1**^ **H} NMR** (151 MHz, CDCl_3_) δ 205.8, 153.8, 137.9, 128.3, 125.6, 121.7, 115.8, 83.7, 52.4, 44.7, 30.9, 25.1. **HRMS (ESI-Q-Orbitrap)** *m* **/** *z*: [M + H]^+^ calcd for C_12_H_13_Cl_2_O_2_: 259.0287. Found: 259.0284.

#### (*R*)-1-(4-Methylchroman-4-yl)­propan-2-one (**1g**)

The desired product was isolated by flash chromatography with 10% EtOAc as eluent and was obtained as a colorless oil. **R_f_** in 10% of EtOAc in hexanes: 0.29. The enantiomeric ratio was determined by HPLC analysis using the following parameters: Daicel Chiralcel OJ-3 column (4.6 mm × 250 mm): 5% iPrOH in Hexane (1.0 mL/min) as mobile phase at 25 °C (rt = 12.0 min (major), 19.7 min (minor)). **GP1:** 57% yield (11.6 mg), 79:21 er; [α]_D_^25^ + 19 (c 2.00, CHCl_3_). **GP2:** 59% yield (12.1 mg), 54:46 er; [α]_D_^25^ + 8 (c 2.00, CHCl_3_). ^**1**^ **H NMR** (500 MHz, CDCl_3_) δ 7.21 (dd, *J* = 7.8, 1.6 Hz, 1H), 7.09 (td, *J* = 1.5, 8 Hz, 1H), 6.88 (td, *J* = 1.5, 8 Hz, 1H), 6.80 (dd, *J* = 8.2, 1.3 Hz, 1H), 4.25–4.11 (m, 2H), 2.80 (d, *J* = 1.2 Hz, 2H), 2.22 (ddd, *J* = 14.1, 7.9, 3.5 Hz, 1H), 2.03 (s, 3H), 1.92 (ddd, *J* = 14.1, 7.2, 3.3 Hz, 1H), 1.45 (s, 3H). ^**13**^ **C­{** ^**1**^ **H} NMR** (126 MHz, CDCl_3_) δ 207.6, 153.9, 129.9, 127.6, 126.6, 120.5, 117.3, 62.8, 54.5, 34.1, 33.2, 32.2, 28.7. **HRMS (ESI-Q-Orbitrap)** *m* **/** *z*: [M + H]^+^ calcd for C_13_H_17_O_2_: 205.1223. Found: 205.1220.

#### (*R*)-1-(3-Methyl-2,3-dihydrobenzofuran-3-yl)­hexan-2-one (**1h**)

The desired product was isolated by flash chromatography with 10% EtOAc in hexanes as eluent and was obtained as a colorless oil. **R_f_** in 8% of EtOAc in hexanes: 0.38. The enantiomeric ratio was determined by HPLC analysis using the following parameters: Daicel Chiralcel OJ-3 column (4.6 mm × 250 mm): 5% iPrOH in Hexane (1.0 mL/min) as mobile phase at 25 °C (rt = 5.7 min (major), 7.9 min (minor)). **GP1:** 35% yield (8.1 mg), 98:2 er; [α]_D_^25^ + 11 (c 1.00, CHCl_3_). **GP2:** 57%, yield (13.3 mg), 81:19 er; [α]_D_^25^ + 8 (c 1.00, CHCl_3_). ^**1**^ **H NMR** (600 MHz, CDCl_3_) δ 7.13 (td, *J* = 7.7, 1.4 Hz, 1H), 7.10 (dd, *J* = 7.4, 1.4 Hz, 1H), 6.87 (td, *J* = 7.4, 1.0 Hz, 1H), 6.80 (d, *J* = 7.9 Hz, 1H), 4.47 (d, *J* = 9.2 Hz, 1H), 4.42 (d, *J* = 9.2 Hz, 1H), 2.93 (d, *J* = 17.2 Hz, 1H), 2.71 (d, *J* = 17.2 Hz, 1H), 2.36–2.29 (m, 2H), 1.55–1.49 (m, 2H), 1.40 (s, 3H) 1.28 (h, *J* = 7.4 Hz, 2H), 0.88 (t, *J* = 7.3 Hz, 3H). ^**13**^ **C­{** ^**1**^ **H} NMR** (151 MHz, CDCl_3_) δ 209.3, 159.0, 134.9, 128.4, 122.7, 120.5, 109.9, 82.6, 52.0, 43.6, 43.6, 25.8, 25.2, 22.3, 13.8. **HRMS (ESI-Q-Orbitrap)** *m* **/** *z*: [M + Na]^+^ calcd for C_15_H_20_O_2_Na: 255.1355. Found: 255.1351.

#### (*R*)-1-(3,6-Dimethyl-2,3-dihydrobenzofuran-3-yl)­hexan-2-one (**1i**)

The desired product was isolated by flash chromatography with 10% EtOAc in hexanes as eluent and was obtained as a colorless oil. The enantiomeric ratio was determined by HPLC analysis using the following parameters: Daicel Chiralcel IC column (4.6 mm × 250 mm): 5% iPrOH in Hexane (1.0 mL/min) as mobile phase at 25 °C (rt = 6.70 min (major), 7.12 min (minor) for the acetone reaction, and 8% iPrOH in Hexane (1.0 mL/min) as mobile phase at 25 °C (rt = 5.3 min (major), 5.6 min (minor) for DMF)). **GP1:** 25% yield (6.1 mg), 98:2 er; [α]_D_^25^ + 35 (c 0.2, CHCl_3_). **GP2:** 18% yield (4.4 mg), 92:8 er; [α]_D_^25^ + 42 (c 1.00, CHCl_3_). ^**1**^ **H NMR** (500 MHz, CDCl_3_) δ 6.97 (d, *J* = 7.5 Hz, 1H), 6.69 (d, *J* = 7.5 Hz, 1H), 6.62 (s, 1H), 4.45 (d, *J* = 9.2 Hz, 1H), 4.40 (d, *J* = 9.2 Hz, 1H), 2.91 (d, *J* = 17.1 Hz, 1H), 2.69 (d, *J* = 17.1 Hz, 1H), 2.34–2.30 (m, 5H), 1.52 (quint, *J* = 7.9 Hz, 2H), 1.38 (s, 3H), 1.27 (sext, *J* = 7.6 Hz, 2H), 0.88 (t, *J* = 7.0 Hz, 3H). ^**13**^ **C­{** ^**1**^ **H} NMR** (126 MHz, CDCl_3_) δ 209.6, 159.4, 138.8, 132.2, 122.4, 121.4, 110.7, 83.0, 52.2, 43.8, 43.5, 26.0, 25.4, 22.4, 21.6, 14.0. **HRMS (ESI-Q-Orbitrap)** *m* **/** *z*: [M + H]^+^ calcd for C_16_H_23_O_2_: 247.1698. Found: 247.1692.

#### (*R*)-1-(7-Bromo-3-methyl-2,3-dihydrobenzofuran-3-yl)­hexan-2-one (**1j**)

The desired product was isolated by flash column chromatography (2–20% of EtOAc in hexanes as eluent) and was obtained as a colorless oil. **R_f_** in 7% EtOAc: 0.23. The enantiomeric ratio was determined by HPLC analysis using the following parameters: Daicel Chiralcel OJ-3 column (4.6 mm × 250 mm): 5% iPrOH in Hexane (1.0 mL/min) as mobile phase at 30 °C (rt = 8.1 min (major), 10.1 min (minor). **GP2:** 21% yield (6.5 mg), 85:15 er; [α]_D_^25^ – 2 (c 1.00, CHCl_3_). ^**1**^ **H NMR** (600 MHz, CDCl_3_) δ 7.29 (dd, *J* = 8.0, 1.2 Hz, 1H), 7.03 (dd, *J* = 7.4, 1.2 Hz, 1H), 6.79–6.74 (m, 1H), 4.56 (d, *J* = 9.4 Hz, 1H), 4.53 (d, *J* = 9.4 Hz, 1H), 2.93 (d, *J* = 17.5 Hz, 1H), 2.74 (d, *J* = 17.5 Hz, 1H), 2.35 (t, *J* = 7.4 Hz, 2H), 1.53 (p, *J* = 7.5 Hz, 2H), 1.41 (s, 3H), 1.28 (dt, *J* = 14.9, 7.5 Hz, 2H), 0.89 (t, *J* = 7.3 Hz, 3H). ^**13**^ **C­{** ^**1**^ **H} NMR** (151 MHz, CDCl_3_) δ 208.8, 156.2, 136.4, 131.4, 122.0, 121.8, 103.0, 83.1, 51.9, 44.7, 43.5, 25.8, 25.2, 22.3, 13.8. **HRMS (ESI-Q-Orbitrap)** *m* **/** *z*: [M + H]^+^ calcd for C_15_H_20_BrO_2_: 311.0641. Found: 311.0635.

#### (*R*)-2-(3-Methyl-2,3-dihydrobenzofuran-3-yl)-1-phenylethan-1-one (**1k**)

The desired product was isolated by flash column chromatography (1–20% of EtOAc in hexanes as eluent) and was obtained as a colorless oil. **R_f_** in 8% of EtOAc in hexanes: 0.22. The enantiomeric ratio was determined by HPLC analysis using the following parameters: Daicel Chiralcel OJ-3 column (4.6 mm × 250 mm): 5% iPrOH in Hexane (1.0 mL/min) as mobile phase at 25 °C (rt = 12.0 min (major), 17.0 min (minor)). **GP1:** 58% yield (14.6 mg), 99:1 er; [α]_D_^25^ + 58 (c 2.00, CHCl_3_). **GP2:** 54% yield (13.6 mg), 92:8 er; [α]_D_^25^ + 16 (c 1.00, CHCl_3_). ^**1**^ **H NMR** (500 MHz, CDCl_3_) δ 7.92 (d, *J* = 7.1 Hz, 2H), 7.56 (t, *J* = 7.4 Hz, 1H), 7.45 (t, *J* = 7.8 Hz, 2H), 7.18–7.11 (m, 2H), 6.89 (td, *J* = 7.4, 1.0 Hz, 1H), 6.82 (d, *J* = 7.9 Hz, 1H), 4.58 (d, J = 9.3 Hz, 1H), 4.54 (d, *J* = 9.4 Hz, 1H), 3.58 (d, *J* = 17.4 Hz, 1H), 3.25 (d, *J* = 17.4 Hz, 1H), 1.50 (s, 3H). ^**13**^ **C­{** ^**1**^ **H} NMR** (126 MHz, CDCl_3_) δ 198.1, 159.0, 137.2, 135.3, 133.3, 128.7, 128.5, 128.0, 122.8, 120.6, 109.9, 82.8, 47.9, 43.8, 25.3. **HRMS (ESI-Q-Orbitrap)** *m* **/** *z*: [M + Na]^+^ calcd for C_17_H_16_O_2_Na: 275.1042. Found: 275.1038. Note: the byproduct generated by the direct alkyl insertion was obtained in 22% yield (4.9 mg), in 10.5:89:5 er.

#### (*R*)-2-(3,6-Dimethyl-2,3-dihydrobenzofuran-3-yl)-1-phenylethan-1-one (**1l**)

The desired product was isolated by flash column chromatography (1–10% of EtOAc in hexanes as eluent) and was obtained as a yellowish oil. **R_f_** in 8% of EtOAc in hexanes: 0.22. The enantiomeric ratio was determined by HPLC analysis using the following parameters: Daicel Chiralcel OJ-3 column (4.6 mm × 250 mm): 5% iPrOH in Hexane (1.0 mL/min) as mobile phase at 25 °C (rt = 12.8 min (major), 18.2 min (minor)). **GP1:** 56% yield (14.9 mg), 98:2 er; [α]_D_^25^ + 32 (c 2.00, CHCl_3_).**GP2:** 36% yield (9.6 mg), 87:13 er; [α]_D_^25^ + 28 (c 2.00, CHCl_3_). The byproduct generated by the direct alkyl insertion was obtained in 19% yield (4.65 mg). ^**1**^ **H NMR** (500 MHz, CDCl_3_) δ 7.95–7.89 (m, 2H), 7.56 (t, *J* = 7.4 Hz, 1H), 7.45 (t, *J* = 7.8 Hz, 2H), 7.05 (d, *J* = 7.5 Hz, 1H), 6.71 (d, *J* = 7.5 Hz, 1H), 6.65 (s, 1H), δ 4.56 (d, *J* = 9.3 Hz, 1H), 4.53 (d, *J* = 9.3 Hz, 1H), 3.56 (d, *J* = 17.4 Hz, 1H), 3.22 (d, *J* = 17.4 Hz, 1H), 2.31 (s, 3H), 1.47 (s, 3H). ^**13**^ **C­{** ^**1**^ **H} NMR** (126 MHz, CDCl_3_) δ 198.2, 159.3, 138.7, 137.3, 133.2, 132.5, 128.6, 128.0, 122.4, 121.3, 110.6, 83.0, 48.0, 43.6, 25.3, 21.5. **HRMS (ESI-Q-Orbitrap)** *m* **/** *z*: [M + Na]^+^ calcd for C_18_H_18_O_2_Na: 289.1199. Found: 289.1193.

#### (*R*)-2-(3-Methyl-5-nitro-2,3-dihydrobenzofuran-3-yl)-1-phenylethan-1-one (**1m**)

The desired product was isolated by flash chromatography with 10% EtOAc in hexanes as eluent and was obtained as a slightly yellowish oil. The enantiomeric ratio was determined by HPLC analysis using the following parameters: Daicel Chiralcel IC column (4.6 mm × 250 mm): 10% iPrOH in Hexane (1.0 mL/min) as mobile phase at 25 °C (rt = 16.5 min (minor), 18.4 min (major)). **GP1:** 21% yield (6.4 mg), 97:3 er; [α]_D_^25^ + 107 (c 1.00, CHCl_3_). **GP2:** 39% yield (11.7 mg), 90:10 er; [α]_D_^25^ + 59 (c 0.50, CHCl_3_). ^**1**^ **H NMR** (600 MHz, CDCl3) δ 8.14 (dd, *J* = 8.8, 2.4 Hz, 1H), 8.08 (d, *J* = 2.4 Hz, 1H), 7.96–7.92 (m, 2H), 7.61–7.57 (m, 1H), 7.51–7.45 (m, 2H), 6.88–6.84 (m, 1H), 4.76 (d, *J* = 9.7 Hz, 1H), 4.74 (d, *J* = 9.6 Hz, 1H), 3.66 (d, *J* = 17.7 Hz, 1H), 3.32 (d, *J* = 17.7 Hz, 1H), 1.53 (s, 3H). ^**13**^ **C­{** ^**1**^ **H} NMR** (151 MHz, CDCl3) δ 197.3, 164.8, 142.2, 139.9, 136.8, 133.8, 128.9, 128.1, 126.3, 119.6, 110.0, 84.9, 47.9, 43.5, 26.3. **HRMS (ESI-Q-Orbitrap)** *m* **/** *z*: [M + Na]^+^ calcd for C_17_H_15_NNaO_4_: 320.0898. Found: 320.0893.

#### (*R*)-1-(3-Benzyl-2,3-dihydrobenzofuran-3-yl)­propan-2-one (**1n**)

The desired product was isolated by flash chromatography with 10% EtOAc as eluent and was obtained as a brown oil. **R_f_** in 10% of EtOAc in hexanes: 0.25. The enantiomeric ratio was determined by HPLC analysis using the following parameters: Daicel Chiralcel OJ-3 column (4.6 mm × 250 mm): 5% iPrOH in Hexane (1.0 mL/min) as mobile phase at 30 °C (rt = 13.2 min (major), 20.0 min (minor)). **GP1:** 60% yield (16.0 mg), 98:2 er; [α]_D_^25^ + 94 (c 2.00, CHCl_3_). **GP2:** 65% yield (17.2 mg), 89:11 er; [α]_D_^25^ + 37 (c 2.00, CHCl_3_). ^**1**^ **H NMR** (500 MHz, CDCl_3_) δ 7.23 (dd, *J* = 5.0, 1.8 Hz, 3H), 7.19–7.13 (m, 1H), 6.89–6.76 (m, 5H), 4.79 (d, *J* = 9.4 Hz, 1H), 4.32 (d, *J* = 9.4 Hz, 1H), 3.14–3.05 (m, 3H), 2.77–2.69 (m, 1H), 2.16 (s, 3H). ^**13**^ **C­{** ^**1**^ **H} NMR** (126 MHz, CDCl_3_) δ 207.0, 159.5, 137.1, 132.3, 130.5, 128.7, 128.0, 126.6, 124.4, 120.0, 109.9, 81.6, 50.2, 47.8, 43.3, 30.9. **HRMS (ESI-Q-Orbitrap)** *m* **/** *z*: [M + H]^+^ calcd for C_18_H_19_O_2_: 267.1379. Found: 267.1375.

#### (*R*)-3-Benzyl-5-methoxy-3-methyl-2,3-dihydrobenzofuran (**1o**)

The desired product was isolated by flash chromatography with 3% EtOAc in hexanes as eluent and was obtained as a colorless oil. **R_f_** in 10% of EtOAc in hexanes: 0.20. The enantiomeric ratio was determined by HPLC analysis using the following parameters: Daicel Chiralcel OJ-3 column (4.6 mm × 250 mm): 10% iPrOH in Hexane (1.0 mL/min) as mobile phase at 25 °C (rt = 10.8 min (major), 14.0 min (minor)). **GP1:** 20% yield (15.0 mg), 97:3 er; [α]_D_^25^ **+** 15 (c 2.00, CHCl_3_). ^**1**^ **H NMR** (300 MHz, CDCl_3_) δ 7.20–7.10 (m, 2H), 6.92–6.78 (m, 2H), 4.81 (t, *J* = 9.1 Hz, 1H), 4.20–4.08 (m, 1H), 3.89 (ddd, *J* = 14.6, 9.3, 5.7 Hz, 1H), 3.02 (dd, *J* = 6, 18 Hz), 2.77 (dd, *J* = 9, 18 Hz), 2.21 (s, 3H). ^**13**^ **C­{** ^**1**^ **H} NMR** (75 MHz, CDCl_3_) δ 206.9, 159.8, 129.5, 128.5, 124.2, 120.5, 109.7, 60.4, 49.2, 37.1, 30.2. **HRMS (ESI-Q-Orbitrap)** *m* **/** *z*: [M + H]^+^ calcd for C_11_H_13_O_2_: 177.0910. Found: 177.0909.

#### (*R*)-3-Ethyl-3-methyl-2,3-dihydrobenzofuran (**2a**)

The desired product was isolated by preparative TLC (5% of EtOAc in hexanes as mobile phase) due to the high volatility of the compound. **R_f_** in 8% of EtOAc in hexanes: 0.62. The enantiomeric ratio was determined by HPLC analysis using the following parameters: Daicel Chiralcel OJ-3 column (4.6 mm × 250 mm): 5% iPrOH in Hexane (1.0 mL/min) as mobile phase at 25 °C (rt = 4.884 min (major), 4.589 min (minor)). **GP3:** 10% (as calculated by ^1^H NMR using 1-bromo-3,5-bis­(trifluoromethyl)­benzene as internal standard), 99:1 er; [α]_D_^25^ – 4 (c 0.5, CHCl_3_). **GP4:** 47% (as calculated by ^1^H NMR using 1-bromo-3,5-bis­(trifluoromethyl)­benzene as internal standard), 86.5:13.5 er; [α]_D_^25^ – 6 (c 1.00, CHCl_3_). ^**1**^ **H NMR** (500 MHz, CDCl_3_) δ 7.10–7.04 (m, 1H), 7.02 (dd, *J* = 7.3, 1.5 Hz, 1H), 6.82 (td, *J* = 7.4, 1.0 Hz, 1H), 6.73 (dd, *J* = 8.1, 0.6 Hz, 1H), 4.30 (d, *J* = 8.5 Hz, 1H), 4.10 (d, *J* = 8.5 Hz, 1H), 1.61 (d, *J* = 7.5 Hz, 1H), 1.58 (d, *J* = 7.5 Hz, 1H), 1.27 (s, 3H), 0.79 (t, *J* = 7.5 Hz, 3H). ^**13**^ **C­{** ^**1**^ **H} NMR** (126 MHz, CDCl_3_) δ 159.6, 135.1, 127.9, 122.9, 120.4, 109.5, 82.2, 45.6, 33.4, 25.1, 9.0.

#### (*R*)-3-Ethyl-3,6-dimethyl-2,3-dihydrobenzofuran (**2b**)

The desired product was isolated by flash chromatography with 2% EtOAc in hexanes as eluent and was obtained as a colorless oil. **R_f_** in 2% of EtOAc in hexanes: 0.23. The enantiomeric ratio was determined by HPLC analysis using the following parameters: Daicel Chiralcel OJ-3 column (4.6 mm × 250 mm): 5% iPrOH in Hexane (1.0 mL/min) as mobile phase at 25 °C (rt = 4.697 min (major), 5.415 min (minor)). **GP4:** 62% yield (10.9 mg), 89:11 er; [α]_D_^25^ – 3 (c 1.00, CHCl_3_). ^**1**^ **H NMR** (300 MHz, CDCl_3_) δ 6.97 (d, *J* = 7.5 Hz, 1H), 6.71 (d, *J* = 7.5 Hz, 1H), 6.63 (s, 1H), 4.36 (d, *J* = 8.6 Hz, 1H), 4.16 (d, *J* = 8.6 Hz, 1H), 2.33 (s, 3H), 1.65 (q, *J* = 7.6 Hz, 2H), 1.33 (s, 3H), 0.86 (t, *J* = 7.5 Hz, 3H). ^**13**^ **C­{** ^**1**^ **H} NMR** (75 MHz, CDCl_3_) δ 159.9, 138.1, 132.2, 122.5, 121.0, 110.2, 82.5, 45.3, 33.4, 25.2, 21.5, 9.0. **HRMS (ESI-Q-Orbitrap)** *m* **/** *z*: [M + H]^+^ calcd for C_12_H_17_O: 177.1273. Found: 177.1272.

#### (*R*)-3-Ethyl-5-methoxy-3-methyl-2,3-dihydrobenzofuran (**2c**)

The desired product was isolated by flash chromatography with 5% EtOAc as eluent in hexanes and was obtained as a colorless oil. The enantiomeric ratio was determined by HPLC analysis using the following parameters: Daicel Chiralcel OJ-3 column (4.6 mm × 250 mm): 2% iPrOH in Hexane (1.0 mL/min) as mobile phase at 25 °C (rt = 5.8 min (minor), 5.5 min (major)). **GP3:** 54% yield (10.3 mg), 97:3 er; [α]_D_^25^ – 7 (c 1.00, CHCl_3_). **GP4:** 67% yield (12.6 mg), 90:10 er; [α]_D_^25^ – 4 (c 1.00, CHCl_3_). ^**1**^ **H NMR** (400 MHz, CDCl_3_) δ 6.72–6.67 (m, 3H), 4.36 (d, *J* = 8.6 Hz, 1H), 4.16 (d, *J* = 8.6 Hz, 1H), 3.79 (s, 3H), 1.66 (q, *J* = 7.5 Hz, 2H), 1.33 (s, 3H), 0.87 (t, *J* = 7.5 Hz, 3H). ^**13**^ **C­{** ^**1**^ **H} NMR** (101 MHz, CDCl_3_) δ 154.2, 153.7, 136.3, 112.4, 109.5, 109.3, 82.4, 56.0, 46.0, 33.2, 24.9, 8.9. **HRMS (ESI-Q-Orbitrap)** *m* **/** *z*: [M + H]^+^ calcd for C_12_H_17_O_2_: 193.1228. Found: 193.1223.

#### (*R*)-3-Benzyl-5-methoxy-3-methyl-2,3-dihydrobenzofuran (**2d**)

The desired product was isolated by flash chromatography with 3% EtOAc in hexanes as eluent and was obtained as a colorless oil. **R_f_** in 3% of EtOAc in hexanes: 0.23. The enantiomeric ratio was determined by HPLC analysis using the following parameters: Daicel Chiralcel OJ-3 column (4.6 mm × 250 mm): 5% iPrOH in Hexane (1.0 mL/min) as mobile phase at 25 °C (rt = 7.9 min (minor), 11.1 min (major)). **GP3:** 59% yield (15.0 mg), 98:2 er; [α]_D_^25^ + 22 (c 2.00, CHCl_3_). **GP4:** 64% yield (16.4 mg), 74:26 er; [α]_D_^25^ + 28 (c 2.00, CHCl_3_). ^**1**^ **H NMR** (600 MHz, CDCl_3_) δ 7.19–7.12 (m, 3H), 6.94 (dd, *J* = 7.9, 1.7 Hz, 2H), 6.60 (d, *J* = 1.6 Hz, 2H), 6.41 (t, *J* = 1.6 Hz, 1H), 4.40 (d, *J* = 8.6 Hz, 1H), 3.97 (d, *J* = 8.6 Hz, 1H), 3.65 (s, 3H), 2.82 (d, *J* = 13.4 Hz, 1H), 2.78 (d, *J* = 13.3 Hz, 1H), 1.26 (s, 3H). ^**13**^ **C­{** ^**1**^ **H} NMR** (151 MHz, CDCl_3_) δ 154.0, 153.6, 137.5, 135.9, 130.4, 128.0, 126.5, 113.2, 109.6, 109.6, 82.3, 56.0, 46.7, 46.4, 24.3. **HRMS (ESI-Q-Orbitrap)** *m* **/** *z*: [M + H]^+^ calcd for C_17_H_19_O_2_: 255.1379. Found: 255.1379.

#### (*R*)-3-benzyl-3-methyl-2,3-dihydrobenzofuran (**2e**)

The desired product was isolated by flash chromatography with 10% EtOAc in hexanes as eluent and was obtained as a colorless oil. **R_f_** in 2% of EtOAc in hexanes: 0.38. The enantiomeric ratio was determined by HPLC analysis using the following parameters: Daicel Chiralcel OJ-3 column (4.6 mm × 250 mm): 5% iPrOH in Hexane (1.0 mL/min) as mobile phase at 25 °C (rt = 8.887 min (major), 7.873 min (minor)). **GP3:** 91% yield (20.4 mg), 98:2 er; [α]_D_^25^ – 12 (c 1.00, CHCl_3_). **GP4:** 91% yield (20.4 mg), 74:26 er; [α]_D_^25^ – 9 (c 1.00, CHCl_3_); 1 mmol scale: 82% (183.9 mg). ^**1**^ **H NMR** (600 MHz, CDCl_3_) δ 7.26–7.21 (m, 3H), 7.15–7.11 (m, 1H), 7.00 (dd, *J* = 7.6, 1.8 Hz, 2H), 6.94 (dd, *J* = 7.4, 1.4 Hz, 1H), 6.86 (td, *J* = 7.4, 1.0 Hz, 1H), 6.76 (d, *J* = 8.0 Hz, 1H), 4.50 (d, *J* = 8.7 Hz, 1H), 4.06 (d, *J* = 8.6 Hz, 1H), 2.90 (d, *J* = 13.3 Hz, 1H), 2.86 (d, *J* = 13.3 Hz, 1H), 1.35 (s, 3H). ^**13**^ **C­{** ^**1**^ **H} NMR** (151 MHz, CDCl_3_) δ 159.5, 137.6, 134.8, 130.4, 128.2, 127.9, 126.5, 123.4, 120.3, 109.7, 81.9, 46.6, 46.3, 24.6. Spectral data match with those previously reported in the literature.^3^

#### (*R*)-3-benzyl-3,6-dimethyl-2,3-dihydrobenzofuran (**2f**)

The desired product was isolated by flash chromatography with 5% EtOAc in hexanes as eluent and was obtained as a colorless oil. **R_f_** in 5% of EtOAc in hexanes: 0.56. The enantiomeric ratio was determined by HPLC analysis using the following parameters: Daicel Chiralcel OJ-3 column (4.6 mm × 250 mm): 5% iPrOH in Hexane (1.0 mL/min) as mobile phase at 25 °C (rt = 7.957 min (major), 6.609 min (minor)). **GP3:** 47% yield (11.2 mg), 98:2 er; [α]_D_^25^ + 3 (c 0.5, CHCl_3_). **GP4:** 71% yield (16.8 mg), 70:30 er; [α]_D_^25^ + 2 (c 1.00, CHCl_3_). ^**1**^ **H NMR** (300 MHz, CDCl_3_) δ 7.32–7.23 (m, 3H), 7.10–7.02 (m, 2H), 6.85 (d, *J* = 7.5 Hz, 1H), 6.71 (d, *J* = 7.6 Hz, 1H), 6.64 (s, 1H), 4.52 (d, *J* = 8.7 Hz, 1H), 4.08 (d, *J* = 8.7 Hz, 1H), 2.93 (d, *J* = 12 Hz, 1H), 2.87 (d, *J* = 12 Hz, 1H), 2.35 (s, 3H), 1.36 (s, 3H). ^**13**^ **C­{** ^**1**^ **H} NMR** (75 MHz, CDCl_3_) δ 159.8, 138.4, 137.7, 132.0, 130.4, 127.9, 126.4, 122.9, 121.0, 110.4, 82.2, 46.6, 46.0, 24.7, 21.5. **HRMS (ESI-Q-Orbitrap)** *m* **/** *z* **:** [M + H]^+^ calcd for C_17_H_19_O: 239.1430. Found: 239.1425.

#### (*R*)-3-Benzyl-7-bromo-3-methyl-2,3-dihydrobenzofuran (**2g**)

The desired product was isolated by flash chromatography with 5% EtOAc in hexanes as eluent and was obtained as a colorless oil. **R_f_** in 5% of EtOAc in hexanes: 0.51. The enantiomeric ratio was determined by HPLC analysis using the following parameters: Daicel Chiralcel OJ-3 column (4.6 mm × 250 mm): 5% iPrOH in Hexane (1.0 mL/min) as mobile phase at 25 °C (rt = 9.428 min (major), 7.595 min (minor)). **GP4:** 74% yield (22.5 mg), 64:36 er; [α]_D_^25^ – 16 (c 2.00, CHCl_3_, 63.5:36.5 er). ^**1**^ **H NMR** (300 MHz, CDCl_3_) δ 7.36–7.24 (m, 4H), 7.08–6.98 (m, 2H), 6.87 (dd, *J* = 7.4, 1.3 Hz, 1H), 6.82–6.73 (m, 1H), 4.63 (d, *J* = 8.9 Hz, 1H), 4.19 (d, *J* = 8.8 Hz, 1H), 2.95 (d, *J* = 12 Hz, 1H), 2.89 (d, *J* = 12 Hz, 1H), 1.39 (s, 3H). ^**13**^ **C­{** ^**1**^ **H} NMR** (75 MHz, CDCl_3_) δ 156.6, 137.0, 136.3, 131.2, 130.4, 128.1, 126.7, 122.5, 121.8, 102.8, 82.3, 47.4, 46.5, 24.6. **HRMS (ESI-Q-Orbitrap)** *m* **/** *z*: [M + Na]^+^ calcd for C_16_H_15_BrONa: 325.0198. Found: 325.0194.

#### (*R*)-3-Benzyl-3-methyl-5-nitro-2,3-dihydrobenzofuran (**2h**)

The desired product was isolated by flash chromatography with 5% EtOAc in hexanes as eluent and was obtained as a colorless oil. The enantiomeric ratio was determined by HPLC analysis using the following parameters: Daicel Chiralcel IC column (4.6 mm × 250 mm): 10% iPrOH in Hexane (1.0 mL/min) as mobile phase at 25 °C (rt = 8.68 min (minor), 9.86 min (major)). **GP3:** 22% yield (5.9 mg), 95:5 er; [α]_D_^25^ + 25 (c 0.5, CHCl_3_). **GP4:** 39% yield (10.4 mg), 93:7 er; [α]_D_^25^ + 69 (c 1.0, CHCl_3_). ^**1**^ **H NMR** (300 MHz, CDCl_3_) δ 8.15 (dd, *J* = 8.9, 2.5 Hz, 1H), 7.87 (d, *J* = 2.5 Hz, 1H), 7.34–7.21 (m, 3H), 6.99 (dd, *J* = 6.6, 3.1 Hz, 2H), 6.80 (d, *J* = 8.8 Hz, 1H), 4.71 (d, *J* = 9.1 Hz, 1H), 4.27 (d, *J* = 9.1 Hz, 1H), 2.97 (d, *J* = 13.4 Hz, 1H), 2.91 (d, *J* = 13.3 Hz, 1H), 1.46 (s, 3H). ^**13**^ **C­{** ^**1**^ **H} NMR** (75 MHz, CDCl_3_) δ 165.1, 141.9, 136.3, 136.3, 130.2, 128.3, 127.0, 125.9, 120.1, 109.7, 83.6, 46.6, 46.0, 24.9. **HRMS (ESI-Q-Orbitrap)** *m* **/** *z*: [M + H]^+^ calcd for C_16_H_16_NO_3_: 270.1125. Found: 270.1124.

## Supplementary Material



## Data Availability

The data underlying this study are available in the published article and its Supporting Information.

## Notes

Experimental procedures along with characterizing data, copies of NMR, HRMS, and HPLC spectra, and calculated structures (PDF)
