# Supplementary material for: Enantioselective Synthesis of 3,3-Disubstituted-2,3-dihydrobenzofurans by Intramolecular Heck-Matsuda/Carbonylation/Stille Coupling
Source: J Org Chem. 2025 Jun 25;90(26):8835–45. doi: 10.1021/acs.joc.4c02503 (PMC12235620; doi:10.1021/acs.joc.4c02503)
Supplement: Supplementary file 1 [file jo4c02503_si_001.pdf]

Supporting Information

Enantioselective synthesis of 3,3-disubstituted-2,3-dihydrobenzofurans by intramolecular Heck-Matsuda/Carbonylation/Stille coupling

Luiz Paulo Melchior de Oliveira Leão‡, Otto Daolio Köster‡, Leonardo José Duarte†, Ataualpa A. C. Braga†, and Carlos Roque Duarte Correia*

‡Chemistry Institute, State University of Campinas, Unicamp, 13083-970, Campinas, São Paulo, Brazil

†Department of Fundamental Chemistry, Institute of Chemistry, University of São Paulo, USP, 05508-900, São Paulo, São Paulo, Brazil

E-mail: croque@unicamp.br

Table of Contents

1. General Information........S3
2. Preparation of Arenediazonium salts........S4

GP0 – Synthesis of Arenediazonium salts........S4

3. General Procedures for the Heck-Matsuda coupled to organotin compounds........S7

General Procedure for *In-Tandem* Heck-Matsuda-Stille coupling for carbonylated products in DMF (GP1)........S7

General Procedure for *In-Tandem* Heck-Matsuda-Stille coupling for carbonylated products in acetone (GP2)........S7

General Procedure for *In-Tandem* Heck-Matsuda-Stille coupling for direct alkylation in DMF (GP3)........S8

General Procedure for *In-Tandem* Heck-Matsuda-Stille coupling for direct alkylation in acetone (GP4)........S8

3.1. Notes........S10

4. Characterization of the products........S11

4.1. Absolute configuration determination for compounds 1a and 2e........S22

4.2. 1mmol scale experiments........S24

4.3. Triphenylfluorotin ^{1}H, ^{13}C and ^{19}F NMR spectra........S25

4.4. Effect of $ZnCO_3$........S24

5. ^{1}H and $^{13}C\{^{1}H\}$ NMR for the arenediazonium salts – SM1 to SM8........S25
6. ^{1}H and $^{13}C\{^{1}H\}$ for the Heck-Matsuda-Stille products........S43
7. Chromatograms........S89
8. Computational Data........S112

1. General Information

Reagents and Solvents: Solvents used for chromatography were technical grade and were distilled prior to use. Commercially available chemicals were purchased and used as received unless otherwise noted. The chiral BOx-Bn was purchased from Sigma-Aldrich (CAS: 133463-88-4). The achiral ligand was synthesized as described in the literature[1].

Chromatography: Analytical thin layer chromatography (TLC) was performed employing Merck® Silica gel 60 F254 plates. Visualization was accomplished with UV light (254 nm), $KMnO_4$, and *p*-anisaldehyde. Chromatographic purifications were performed on flash column chromatography using Merck® Silica gel 60 (230-400 mesh) as stationary phase and on a Biotage-Isolera One flash purification system, employing Biotage® SNAP Ultra 10 g, 25 g, or 50 g as stationary phase operating in a gradient mode (EtOAc/hexanes).

Optical Specific Rotation: Optical rotations were measured on a Perkin Elmer 341 polarimeter with a sodium lamp using a 1.0 cm quartz glass cell and are reported as follows: $[\alpha]_D^{25}$ (°C) (c (g/100 mL), solvent).

^{1}H, ^{19}F, and ^{13}C Nuclear Magnetic Resonance: ^{1}H, ^{19}F proton-decoupled, and ^{13}C NMR spectra were acquired on a Bruker DPX250 (250 MHz for ^{1}H NMR and 62.5 MHz for ^{13}C NMR), Bruker Avance II (300 MHz for ^{1}H NMR and 75 MHz for ^{13}C) Bruker Avance 400 (400 MHz for ^{1}H NMR and 100 MHz for ^{13}C NMR), Bruker Avance 500 (500 MHz for ^{1}H and 125 MHz for ^{13}C NMR), or Bruker Avance 600 (600 MHz for ^{1}H and 150 MHz for ^{13}C NMR). 1,3-Bis(trifluoromethyl)-5-bromobenzene was used as an internal standard for the determination of chemical yields by ^{1}H NMR. Chemical shifts (δ) are reported in ppm. Multiplicity data are reported as follows: s = singlet, d = doublet, t = triplet, q = quartet, quint = quintet, sext = sextet, br s = broad singlet, dd = doublet of doublets, dt = doublet of triplets, ddd = doublet of doublet of doublets, ddt = doublet of doublet of triplets, dtd = doublet of triplet of doublets, dqd = doublet of quartet of doublets, and m = multiplet. The multiplicity is followed by the coupling constant(s) in Hz and integration.

HRMS: High-resolution mass spectrometry (HRMS) was measured using electrospray ionization (ESI) (Waters Xevo Q-Tof, Bruker qTOF Impact II, and Q-Exactive Plus-Thermo Fisher Scientific).

HPLC: Enantiomeric ratios (*er*) were calculated through the integration of enantiomers' corresponding signals, set by racemic samples. The products were analyzed through High-Performance Liquid Chromatography (HPLC) on an Agilent Technologies 1260 Infinity with a DAD detector equipped with Daicel Chiralpak® chiral columns as stationary phase and hexane:iPrOH mixtures as mobile phase.

[1] Altenhoff, G.; Goddard, R.; Lehmann, C. W.; Glorius, F.; *J. Am. Chem. Soc.* **2004**, 126, 46. 15195-15201

2. Preparation of Arenediazonium salts

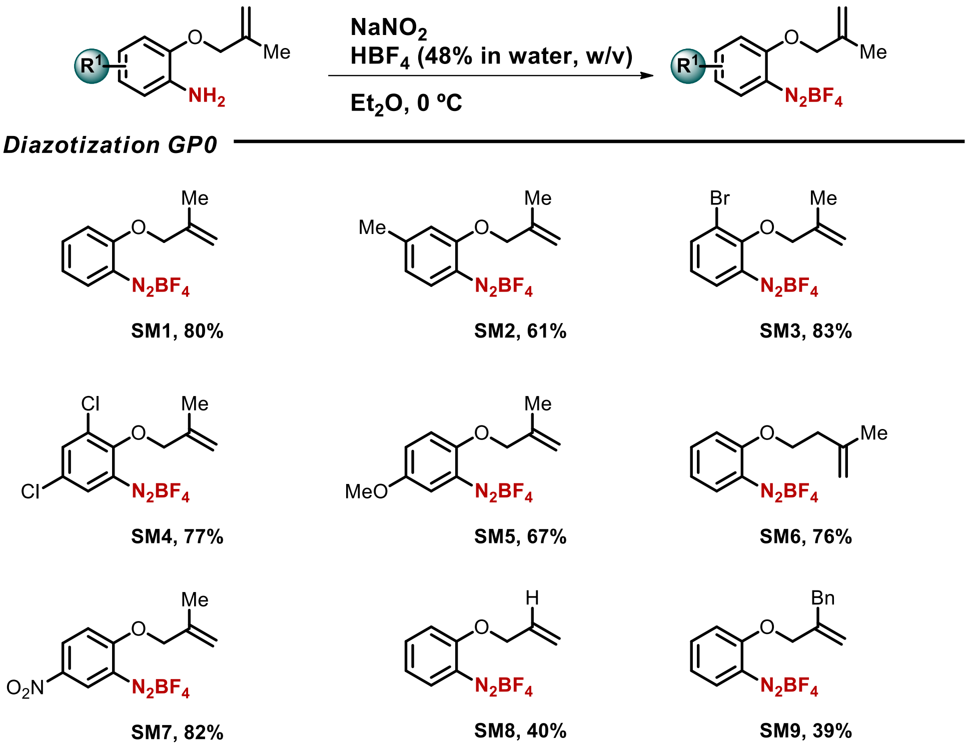

Figure S1. Synthesized arenediazonium salts used in this work.

GP0 – Synthesis of Arenediazonium salts

To a stirring solution of the aniline (1 equiv) in Et_2O (1.2 mmol/mL) at 0 °C was added a solution of HBF_4 (48% w/v in water, 3 equiv). The mixture was stirred for 30 min and then a solution of $NaNO_2$ (1.05 equiv) in water (0.17 mL/mmol of $NaNO_2$) was added dropwise, resulting in the formation of a precipitate. This precipitate was filtered and washed with cold Et_2O and with cold Et_2O:MeOH solution (4:1, v/v). The solid was then recrystallized as follows: it was dissolved in acetone at room temperature and Et_2O was added until the formation of a precipitate was complete (note: in some cases, it was necessary to cool the solution in liquid nitrogen, to precipitate the diazonium salt). Next, this solid was filtered and washed with cold Et_2O:MeOH (4:1, v/v). The diazonium salts are stable for weeks if stored in the freezer. No safety issues were observed during the preparation and handling of these salts.[2]

[2] Chorro, T. H. D.; Souza, E. L. S.; Köster, O. D.; Polo, E. C.; Carmona, R. C.; Silva, V. H. M.; Junior, J. M. B.; Correia, C. R. D. *Adv. Synth. Catal.* **2023**, *365*, 211 – 223.

1-(2-((2-methylallyl)oxy)phenyl)-2-(tetrafluoro-l5-boraneyl)diazene (SM1)

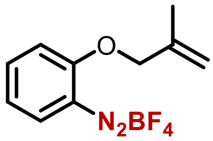

SM1

Salmon solid, 80% yield (1.387g).

^{1}H NMR (300 MHz, Acetone-d_6) δ 8.58 (dd, J = 8.4, 1.7 Hz, 1H), 8.28 (ddd, J = 9.1, 7.5, 1.7 Hz, 1H), 7.74 (dd, J = 9.0, 0.8 Hz, 1H), 7.50 (t, J = 8.4 Hz, 1H), 5.26 (s, 1H), 5.13 (s, 1H), 5.06 (s, 2H), 1.94 – 1.88 (s, 3H).

^{13}C{^{1}H} NMR (75 MHz, Acetone-d_6) δ 162.3, 144.2, 138.8, 132.4, 123.2, 115.7, 114.4, 101.9, 74.7, 18.4.

The spectral data match with those reported in the literature[3]

1-(4-methyl-2-((2-methylallyl)oxy)phenyl)-2-(tetrafluoro-l5-boraneyl)diazene (SM2)

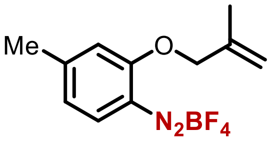

SM2

White solid, 61% yield (0.5014g).

^{1}H NMR (500 MHz, Acetone-d_6) δ 8.50 (d, J = 8.6 Hz, 1H), 7.64 (s, 1H), 7.37 (dd, J = 8.6, 1.3 Hz, 1H), 5.26 (s, 1H), 5.14 (s, 1H), 5.05 (s, 2H), 2.64 (s, 3H), 1.91 (s, 3H).

^{13}C{^{1}H} NMR (126 MHz, Acetone-d_6) δ 162.4, 158.8, 138.8, 131.9, 124.7, 115.8, 114.2, 98.2, 74.6, 22.7, 18.4.

The spectral data match with those reported in the literature[2,4]

1-(3-bromo-2-((2-methylallyl)oxy)phenyl)-2-(tetrafluoro-l5-boraneyl)diazene (SM3)

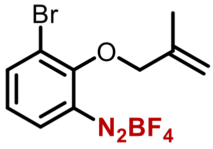

SM3

White solid, 83% yield (0.6331g).

^{1}H NMR (500 MHz, Acetone-d_6) δ 8.75 (dd, J = 8.4, 1.5 Hz, 1H), 8.62 (dd, J = 8.2, 1.5 Hz, 1H), 7.72 (t, J = 8.3 Hz, 1H), 5.33 (s, 1H), 5.19 (s, 1H), 5.17 (s, 2H), 1.99 (s, 3H).

^{13}C{^{1}H} NMR (126 MHz, Acetone-d_6) δ 158.3, 147.6, 139.6, 132.9, 127.5, 117.0, 115.5, 111.3, 80.1, 18.7.

1-(3,5-dichloro-2-((2-methylallyl)oxy)phenyl)-2-(tetrafluoro-l5-boraneyl)diazene (SM4)

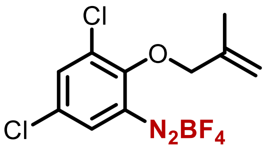

SM4

Yellow solid, 77% yield (0.9925g).

^{1}H NMR (600 MHz, Acetone-d_6) δ 8.79 (d, J = 2.5 Hz, 1H), 8.57 (d, J = 2.5 Hz, 1H), 5.32 (s, 1H), 5.22 (s, 2H), 5.19 (s, 1H), 1.98 (s, 3H).

^{13}C{H} NMR (151 MHz, Acetone-d_6) δ 157.0, 144.3, 139.4, 130.6, 129.9, 129.5, 115.7, 111.9, 80.5, 18.5.

The spectral data match with those reported in the literature[3]

---

[3] Carmona, R. C.; Kõster, O. D.; Correia, C. R. D.; *Angew. Chem. Int. Ed.* **2018**, *57*, 12067.

[4] Ju, B.; Chen, S.; Kong, W. *Org. Lett.* **2019**, *21*, 23, 9343–9347

1-(5-methoxy-2-((2-methylallyl)oxy)phenyl)-2-(tetrafluoro-l5-boraneyl)diazene (SM5)

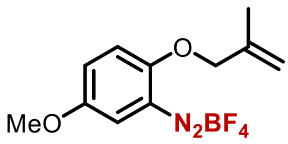

SM5

Green solid, 67% yield (0.0804g).

^{1}H NMR (600 MHz, Acetone-d_6) δ 8.11 (d, J = 3.3 Hz, 1H), 7.90 (dd, J = 9.6, 3.3 Hz, 1H), 7.71 (d, J = 9.7 Hz, 1H), 5.25 (s, 1H), 5.14 (s, 1H), 5.04 (s, 2H), 3.93 (s, 3H), 1.91 (s, 3H).

^{13}C{^{1}H} NMR (151 MHz, Acetone-d_6) δ 158.7, 153.9, 138.9, 133.9, 116.9, 114.3, 111.4, 101.1, 74.8, 56.4, 18.3.

The spectral data match with those reported in the literature[3].

1-(2-((3-methylbut-3-en-1-yl)oxy)phenyl)-2-(tetrafluoro-l5-boraneyl)diazene (SM6)

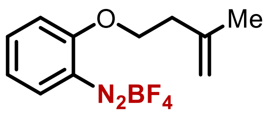

SM6

White solid, 76% yield (0.6164g).

^{1}H NMR (500 MHz, Acetone-d_6) δ 8.60 (dd, J = 8.5, 1.7 Hz, 1H), 8.32 (ddd, J = 9.0, 7.4, 1.7 Hz, 1H), 7.81 (d, J = 8.1 Hz, 1H), 7.57 – 7.48 (m, 1H), 4.91 (s, 2H), 4.71 (t, J = 6.6 Hz, 2H), 2.71 (t, J = 6.5 Hz, 2H), 1.85 (s, 3H).

^{13}C{^{1}H} NMR (126 MHz, Acetone-d_6) δ 162.7, 144.4, 141.2, 132.3, 123.1, 115.5, 112.6, 101.8, 70.1, 36.3, 21.6.

The spectral data match with those reported in the literature[3].

1-(2-((2-methylallyl)oxy)-5-nitrophenyl)-2-(tetrafluoro-l5-boraneyl)diazene (SM7)

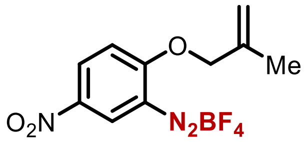

SM7

White solid, 82% yield (0.2925g).

^{1}H NMR (400 MHz, Acetone-d_6) δ 9.61 (d, J = 2.7 Hz, 1H), 9.03 (dd, J = 9.7, 2.8 Hz, 1H), 8.01 (d, J = 9.7 Hz, 1H), 5.31 (s, 1H), 5.26 (s, 2H), 5.19 (s, 1H), 1.94 (s, 3H).

^{13}C{^{1}H} NMR (126 MHz, Acetone-d_6) δ ^{13}C NMR (101 MHz, Acetone) δ 165.3, 141.2, 138.2, 138.1, 129.2, 116.8, 115.3, 104.0, 76.5, 18.3.

The spectral data match with those reported in the literature[3].

1-(2-(allyloxy)phenyl)-2-(tetrafluoro-l5-boraneyl)diazene (SM8)

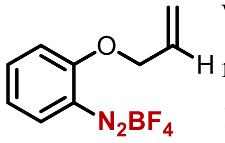

SM8

White solid, 40% yield (0.4918g).

^{1}H NMR (400 MHz, Acetone-d_6) δ 8.60 (d, J = 8.4 Hz, 1H), 8.30 (t, J = 8.8 Hz, 1H), 7.75 (d, J = 8.9 Hz, 1H), 7.52 (t, J = 7.9 Hz, 1H), 6.19 (ddt, J = 16.3, 10.8, 5.5 Hz, 1H), 5.62 (d, J = 17.3 Hz, 1H), 5.43 (d, J = 10.6 Hz, 1H), 5.15 (d, J = 5.5 Hz, 2H).

^{13}C{^{1}H} NMR (101 MHz, Acetone-d_6) δ 162.3, 144.2, 132.4, 131.0, 123.2, 119.5, 119.5, 115.7, 102.0, 72.1.

The spectral data match with those reported in the literature[2]

1-(2-((2-benzylallyl)oxy)phenyl)-2-(tetrafluoro-l5-boraneyl)diazene (SM9)

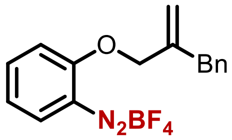

SM9

White solid, 39% yield (0.3573g).

^{1}H NMR (400 MHz, Acetone-*d*$_6$) δ 8.61 (dd, *J* = 8.5, 1.7 Hz, 1H), 8.33 – 8.20 (m, 1H), 7.68 (d, *J* = 9.0 Hz, 1H), 7.51 (ddd, *J* = 8.4, 7.4, 0.9 Hz, 1H), 7.38 – 7.21 (s, 5H), 5.45 (s, 1H), 5.23 (s, 1H), 5.09 (s, 2H), 3.63 (s, 2H).

^{13}C{^{1}H} NMR (63 MHz, Acetone -*d*$_6$) δ 162.1, 144.2, 142.2, 138.5, 132.5, 129.0, 128.5, 126.5, 123.3, 116.2, 115.5, 101.9, 73.5, 39.2.

3. General Procedures for the Heck-Matsuda coupled to organotin compounds

General Procedure for *In-Tandem* Heck-Matsuda-Stille coupling for carbonylated products in DMF (GP1)

To a 4 mL vial containing a magnetic stir bar, it was added $Pd(OAc)_2$ (5 mol%, 1.2 mg), the chiral *N*,*N*-ligand 2,2′-Bis[(4*S*)-4-benzyl-2-oxazoline] (10 mol%, 3.2 mg), 2 mL of DMF, and the reaction was left stirring for 10 minutes at 40 ºC using an aluminium heating block. After the pre-catalyst activation, the respective reactants were added in the following order: the arenediazonium salt (1 equiv.) and the organotin compound (2 equiv., 28 µL, for $SnMe_4$; 4 equiv., 132 µL for $SnBu_4$, and 1 equiv. for $SnPh_4$, 42.7 mg). The vial was then sealed with a holed screw cap containing a PTFE septum, and CO was gently bubbled into the solution for 15 seconds (see Note 1). The outlet needle was then removed, and the reaction was left stirring for 6 h at 40 ºC. Next, the crude was diluted with distilled H_2O, extracted with EtOAc (3x 10 mL), washed with brine (3x 10 mL), dried over anhydrous $NaSO_4$, filtered, and concentrated under reduced pressure. The crude was filtered through a 10 cm silica-gel pad with EtOAc as eluent and was purified by flash chromatography (Biotage Isolera®) to furnish the carbonylated dihydrofuran products.

General Procedure for *In-Tandem* Heck-Matsuda-Stille coupling for carbonylated products in acetone (GP2)

To a 4 mL vial containing a magnetic stir bar, it was added $Pd(OAc)_2$ (5 mol%, 1.2 mg), the chiral *N*,*N*-ligand 2,2′-Bis[(4*S*)-4-benzyl-2-oxazoline] (10 mol%, 3.2 mg), 2 mL of acetone, and the reaction was left stirring for 10 minutes at 40 ºC using an aluminium heating block. After the pre-catalyst activation the respective reactants were added in the following order: $ZnCO_3$ (0.5 equiv., 6.5 mg), the arenediazonium salt (1 equiv.) and the organotin compound (2 equiv., 28 µL, for $SnMe_4$; 4 equiv., 132 µL for $SnBu_4$, and 1 equiv. for $SnPh_4$, 42.7 mg). The vial was then sealed with a holed screw cap containing a PTFE septum, and CO was gently bubbled into the solution for 15 seconds (see Note 1). The outlet needle was then removed, and the reaction was left stirring for 6 h at 40 ºC. The crude was filtered through a 10 cm silica-gel pad using EtOAc as eluent, and purified by flash chromatography (Biotage Isolera®, unless otherwise noted) to furnish the carbonylated dihydrofuran products.

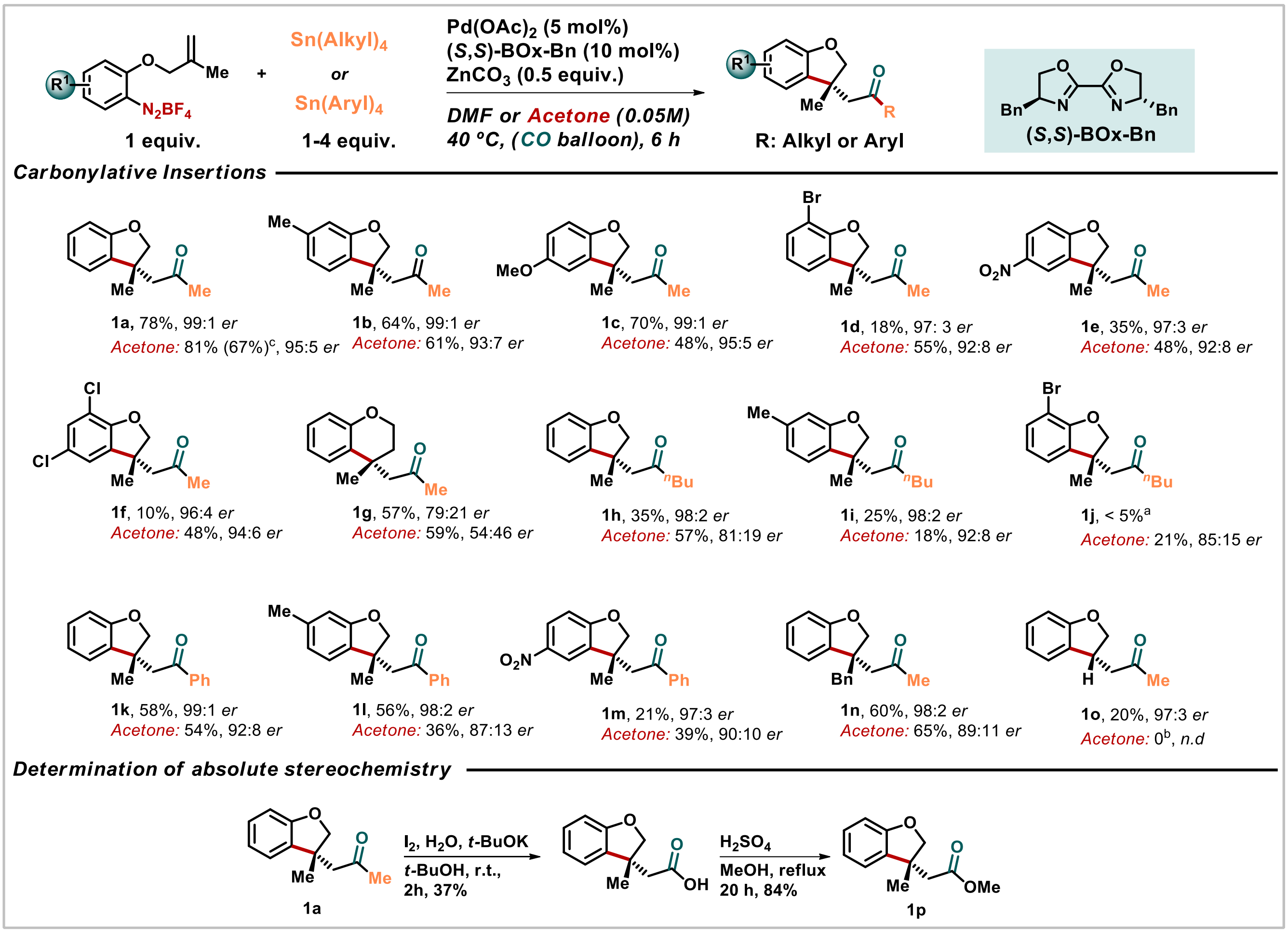

Figure S2. Scope for Carbonylative Enantioselective Heck-Matsuda-Stille reaction.

General Procedure for *In-Tandem* Heck-Matsuda-Stille coupling for direct alkylation in DMF (GP3)

To a 4 mL vial containing a magnetic stir bar, it was added $Pd(OAc)_2$ (5 mol%, 1.2 mg), the chiral *N*,*N*-ligand 2,2′-Bis[(4*S*)-4-benzyl-2-oxazoline] (10 mol%, 3.2 mg), 2 mL of DMF and the reaction left stirring for 10 minutes at 40 ºC using an aluminium heating block. After the pre-catalyst activation, the respective reactants were added in the following order: the arenediazonium salt (1 equiv.) and the organotin compound (2 equiv. for $SnMe_4$; 4 equiv. for $SnBu_4$ and 1 equiv. for $SnPh_4$). The vial was then sealed and the reaction was left stirring for 6 h at 40 ºC. Next, the crude was diluted with distilled H_2O, extracted with EtOAc (3x 10 mL), washed with brine (3x 10 mL) dried over anhydrous $NaSO_4$, filtered, and concentrated under reduced pressure. The residue was filtered through a 10 cm silica-gel pad with EtOAc as eluent, and purified by flash chromatography (Biotage Isolera®) to furnish the carbonylated products.

General Procedure for *In-Tandem* Heck-Matsuda-Stille coupling for direct alkylation in acetone (GP4)

To a 4 mL vial containing a magnetic stir bar, it was added $Pd(OAc)_2$ (5 mol%, 1.2 mg), the chiral *N*,*N*-ligand 2,2′-Bis[(4*S*)-4-benzyl-2-oxazoline] (10 mol%, 3.2 mg), 2 mL of acetone and the reaction was left stirring for 10 minutes at 40 ºC ºC using an aluminium heating block. After the pre-catalyst activation, the respective reactants were added in the following order: $ZnCO_3$ (0.5 equiv., 6.5 mg), the arenediazonium salt (1 equiv.), and the organotin compound (2 equiv. for $SnMe_4$ and 1 equiv. for $SnPh_4$). The vial was then sealed and the reaction was left stirring for 6 h at 40 ºC. The crude was

filtered through a 10 cm silica-gel pad using EtOAc as eluent, and purified by flash chromatography (Biotage Isolera®, unless otherwise noted) to furnish the alkylated products.

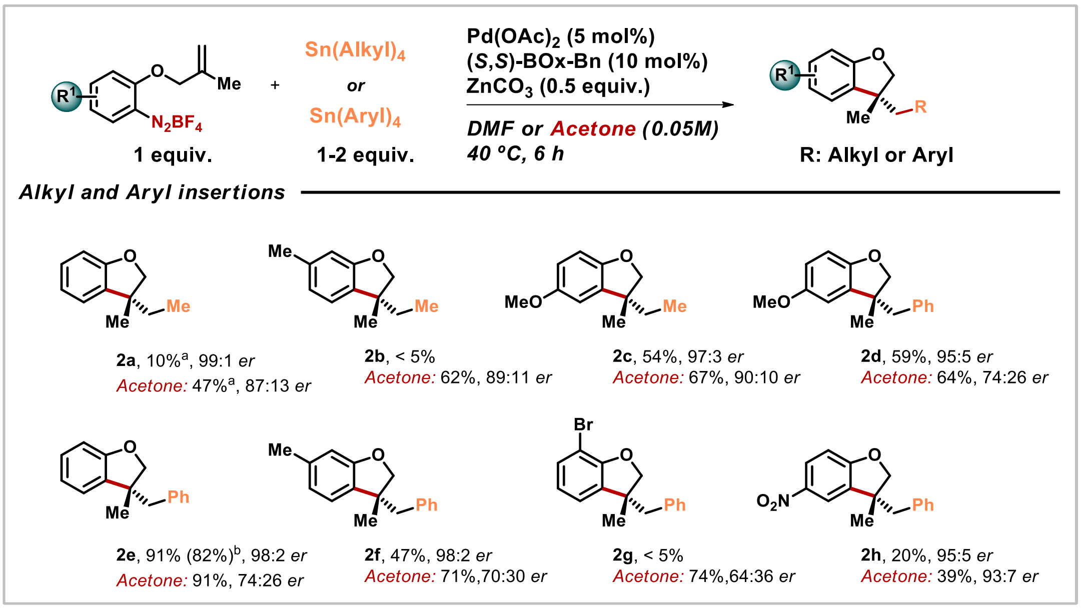

Figure S3. Scope for Enantioselective Heck-Matsuda-Stille reaction.

3.1. Notes

Note 1: How the CO gas is introduced into the reaction system:

The carbonylative reactions were performed in a 4 mL dram vial with a holed screw cap containing a PTFE septum. After the formation of the Pd-*N*,*N*-ligand pre-catalyst in the indicated solvent, the addition of the arenediazonium salt and the organotin compound, the PTFE septum was punctured with a CO balloon and an outlet needle to allow the atmosphere exchange. The CO balloon needle was then inserted into the solvent and CO was left bubbling for 15 seconds, after which the outlet needle was removed to create a CO atmosphere in the headspace of the vial.

Note 2: How the volatile compounds **1o**, **2a** and **2b** were obtained:

These compounds are highly volatile. Therefore, rotaevaporation is **not** recommended. Instead, after completion of the reaction, the solvent was removed by a gentle flow of N_2. These compounds were then purified by preparative TLC.

Note 3: Color changes during the reaction course:

It was observed that after a few hours, the reaction goes from a clear solution to a black and turbid one.

Note 4: How the racemates were synthesized:

The racemates were prepared following the same general procedures (GP1, GP2, GP3 and GP4) for the chiral compounds, but using the commercial achiral *N*,*N*-ligand Bisox-$(Me)_4$ (CAS: 49585-66-2), which was synthesized as described in the literature[1].

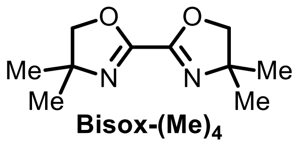

Bisox-$(Me)_4$

Note 5: When preparing small amounts of the arenediazonium salts:

To avoid freezing the solution during the preparation of small amounts of arenediazonium salts (starting from about 150 mg of aniline), a larger volume of ether should be added to achieve total diazotization of the respective aniline.

Note 6: Visualization of the Heck-Matsuda-Stille products on the TLC plates:

The majority of the 2,3-dihydrobenzofurans synthesized stains pink, red, or blue color in the TLC plate with *p*-Anisaldehyde stainer solution. Some of these products are hard to visualize using only long (365 nm) and short (254 nm) wave lamps on the UV-Chamber.

4. Characterization of the products

(*R*)-1-(3-methyl-2,3-dihydrobenzofuran-3-yl)propan-2-one (1a)

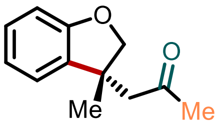

1a

The desired product was isolated by Biotage Isolera with 10% EtOAc in hexanes and was obtained as a colorless oil.

The enantiomeric ratio was determined by HPLC analysis using the following parameters: Daicel Chiralcel IC column (4.6 mm x 250 mm): 10% iPrOH in Hexane (1.0 mL/min) as mobile phase at 25 ºC. (rt = 16.5 min (minor), 18.4 min (major)).

GP1: 78% (14.8 mg), 99:1 *er*; $[\alpha]_D^{25}$ +64 (c 1.0, $CHCl_3$).

GP2: 81% (15.4 mg), 95:5 *er*; $[\alpha]_D^{25}$ +35 (c 0.5, $CHCl_3$); 1 mmol scale: 67% (127.4 mg).

^{1}H NMR (500 MHz, $CDCl_3$) δ 7.14 (td, *J* = 7.9, 1.4 Hz, 1H), 7.10 (dd, *J* = 7.4, 0.9 Hz, 1H), 6.88 (td, *J* = 7.4, 0.8 Hz, 1H), 6.80 (d, *J* = 8.0 Hz, 1H), 4.46 (d, *J* = 9.3 Hz, 1H), 4.41 (d, *J* = 9.3 Hz, 1H), 2.95 (d, *J* = 17.3 Hz, 1H), 2.76 (d, *J* = 17.3 Hz, 1H), 2.09 (s, 3H), 1.41 (s, 3H).

^{13}C{^{1}H} NMR (126 MHz, $CDCl_3$) δ 206.9, 159.1, 135.0, 128.6, 122.8, 120.7, 110.0, 82.6, 53.0, 43.7, 31.2, 25.3.

HRMS (ESI-Q-Orbitrap) m/z: [M+H]+ Calculed for $C_{12}H_{15}O_2$: 191.1066; Found: 191.1065.

(*R*)-1-(3,6-dimethyl-2,3-dihydrobenzofuran-3-yl)propan-2-one (1b)

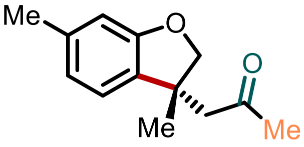

1b

The desired product was isolated by Biotage Isolera with 10% EtOAc in hexanes and was obtained as a colorless oil.

The enantiomeric ratio was determined by HPLC analysis using the following parameters: Daicel Chiralcel IC column (4.6 mm x 250 mm): 5% iPrOH in Hexane (1.0 mL/min) as mobile phase at 25 ºC. (rt = 7.2 min (minor), 8.3 min (major)).

GP1: 64% (13.1 mg), 99:1; $[\alpha]_D^{25}$ +115 (c 1.00, $CHCl_3$).

GP2: 61% (12.4 mg), 93:7 *er*; $[\alpha]_D^{25}$ +63 (c 1.00, $CHCl_3$).

^{1}H NMR (250 MHz, $CDCl_3$) δ 6.98 (d, *J* = 7.5 Hz, 1H), 6.69 (d, *J* = 7.5 Hz, 1H), 6.62 (s, 1H), 4.45 (d, *J* = 9.3 Hz, 1H), 4.38 (d, *J* = 9.3 Hz, 1H), 2.93 (d, *J* = 17.2 Hz, 1H), 2.72 (d, *J* = 17.2 Hz, 1H), 2.30 (s, 3H), 2.09 (s, 3H), 1.39 (s, 3H).

^{13}C{^{1}H} NMR (75 MHz, $CDCl_3$) δ 208.9, 155.5, 131.6, 124.8, 119.0, 115.1, 88.3, 58.4, 51.7, 39.2, 33.4, 31.8, 21.3.

HRMS (ESI-Q-Orbitrap) m/z: $[M+H]^+$ Calculated for $C_{13}H_{17}O_2$: 205.1228. Found: 205.1227.

(*R*)-1-(5-methoxy-3-methyl-2,3-dihydrobenzofuran-3-yl)propan-2-one (1c)

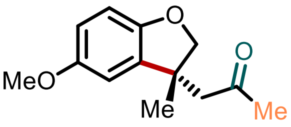

1c

The desired product was isolated by Bitoage Isolera with 10% EtOAc in hexanes as a mobile phase and was obtained as a yellowish oil. **Rf** in 10% of EtOAc: 0.16.

The enantiomeric ratio was determined by HPLC analysis using the following parameters: Daicel Chiralcel OJ-3 column (4.6 mm x 250 mm): 5% iPrOH in Hexane (1.0 mL/min) as mobile phase at 30 ºC (rt = 14.7 min (major), 18.9 min (minor)).

GP1: 70% (15.3 mg), 99:1 *er*; $[\alpha]_D^{25}$+66 (c 2.00, $CHCl_3$).

GP2: 48% (10.6 mg), 95:5 *er*; $[\alpha]_D^{25}$+65 (c 2.00, $CHCl_3$).

^{1}H NMR (500 MHz, $CDCl_3$) δ 6.73 – 6.65 (m, 3H), 4.43 (d, *J* = 9.3 Hz, 1H), 4.38 (d, *J* = 9.9 Hz, 1H), 3.77 (s, 3H), 2.92 (d, *J* = 17.3 Hz, 1H), 2.75 (d, *J* = 17.3 Hz, 1H), 2.10 (s, 3H), 1.40 (s, 3H).

^{13}C{^{1}H} NMR (126 MHz, $CDCl_3$) δ 206.7, 154.3, 153.0, 135.9, 113.1, 109.8, 109.3, 82.7, 56.1, 52.7, 44.1, 31.1, 24.8.

HRMS (ESI-Q-Orbitrap) m/z: $[M+H]^+$ Calculated for $C_{13}H_{17}O_3$: 221.1172. Found: 221.1168.

(*R*)-1-(7-bromo-3-methyl-2,3-dihydrobenzofuran-3-yl)propan-2-one (1d)

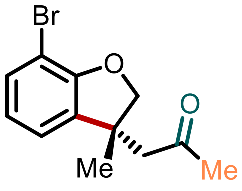

1d

The desired product was isolated by flash column chromatography from 2 to 20% of EtOAc and was obtained as a colorless oil. **Rf** in 8% of EtOAc in hexanes: 0.17.

The enantiomeric ratio was determined by HPLC analysis using the following parameters: Daicel Chiralcel OJ-3 column (4.6 mm x 250 mm): 5% iPrOH in Hexane (1.0 mL/min) as mobile phase at 30 ºC. (rt = 13.4 min (major), 16.9 min (minor)).

GP1: 18% (4,7 mg), 97:3 *er*; $[\alpha]_D^{25}$+7 (c 1.00, $CHCl_3$).

GP2: 55% (14.7 mg), 92:8 *er*; $[\alpha]_D^{25}$+22 (c 2.00, $CHCl_3$). A mixture of products was observed in 60% of the carbonylated product and 40% of the direct insertion (not isolated) of the alkyl tin compound.

^{1}H NMR (600 MHz, $CDCl_3$) δ 7.29 (dd, *J* = 8.0, 1.2 Hz, 1H), 7.03 (dd, *J* = 7.4, 1.1 Hz, 1H), 6.79 – 6.73 (m, 1H), 4.54 (d, *J* = 9.4 Hz, 1H), 4.52 (d, *J* = 9.9 Hz, 1H), 2.96 (d, *J* = 17.7 Hz, 1H), 2.78 (d, *J* = 17.7 Hz, 1H), 2.12 (s, 3H), 1.42 (s, 3H).

^{13}C{^{1}H} NMR (151 MHz, $CDCl_3$) δ 206.2, 156.2, 136.3, 131.5, 122.1, 121.8, 103.0, 83.0, 52.7, 44.7, 31.0, 25.2.

HRMS (ESI-Q-Orbitrap) m/z: $[M+H]^+$ Calculated for $C_{12}H_{14}BrO_2$: 269.0171. Found: 269.0167.

(*R*)-1-(3-methyl-5-nitro-2,3-dihydrobenzofuran-3-yl)propan-2-one (1e)

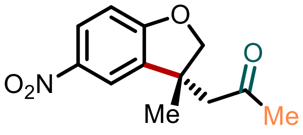

1e

The desired product was isolated by Biotage Isolera with 10% EtOAc in hexanes and was obtained as a colorless oil.

The enantiomeric ratio was determined by HPLC analysis using the following parameters: Daicel Chiralcel OJ-3 column (4.6 mm x 250 mm): 20% iPrOH in Hexane (1.0 mL/min) as mobile phase at 25 ºC. (rt = 21.7 min (minor), 25.9 min (major)).

GP1: 35% (8.3 mg), 97:3 *er*; $[\alpha]_D^{25}$ +51 (c 1.00, $CHCl_3$).

GP2: 48% (11.3 mg), 92:8 *er*; $[\alpha]_D^{25}$ +37 (c 1.00, $CHCl_3$).

^{1}H NMR (500 MHz, $CDCl_3$) δ 8.12 (dd, *J* = 8.8, 2.4 Hz, 1H), 8.00 (d, *J* = 2.4 Hz, 1H), 6.84 (d, *J* = 8.8 Hz, 1H), 4.62 (s, 2H), 3.05 (d, *J* = 18.0 Hz, 1H), 2.83 (d, *J* = 18.0 Hz, 1H), 2.16 (s, 3H), 1.44 (s, 3H).

^{13}C{^{1}H} NMR (126 MHz, $CDCl_3$) δ 205.9, 164.7, 142.2, 136.6, 126.2, 119.5, 110.0, 84.6, 52.6, 43.2, 31.0, 26.0.

HRMS (ESI-Q-Orbitrap) m/z: $[M+H]^+$ Calculated for $C_{12}H_{14}NO_4$: 236.0922. Found: 236.0925.

(*R*)-1-(5,7-dichloro-3-methyl-2,3-dihydrobenzofuran-3-yl)propan-2-one (1f)

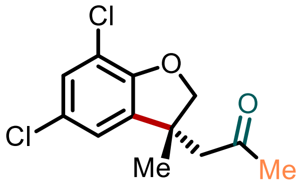

1f

The desired product was isolated by flash column chromatography from 2 to 20% of EtOAc and was obtained as a yellow oil. **Rf** in 7% EtOAc: 0.26.

The enantiomeric ratio was determined by HPLC analysis using the following parameters: Daicel Chiralcel OJ-3 column (4.6 mm x 250 mm): 5% iPrOH in Hexane (1.0 mL/min) as mobile phase at 30 ºC. (rt = 9.219 min (major), 10.506 min (minor)).

GP1: 10% (2.6 mg), 96:4 *er*; $[\alpha]_D^{25}$ +1 (c 0.5, $CHCl_3$).

GP2: 48% (12.3 mg), 94:6 *er*; $[\alpha]_D^{25}$ +35 (c 2.00, $CHCl_3$).

^{1}H NMR (600 MHz, $CDCl_3$) δ 7.15 (d, *J* = 2.1 Hz, 1H), 6.96 (d, *J* = 2.1 Hz, 1H), 4.55 (s, 2H), 2.95 (d, *J* = 17.9 Hz, 1H), 2.78 (d, *J* = 17.9 Hz, 1H), 2.14 (s, 3H), 1.41 (s, 3H).

^{13}C{^{1}H} NMR (151 MHz, $CDCl_3$) δ 205.8, 153.8, 137.9, 128.3, 125.6, 121.7, 115.8, 83.7, 52.4, 44.7, 30.9, 25.1.

HRMS (ESI-Q-Orbitrap) m/z: $[M+H]^+$ Calculated for $C_{12}H_{13}Cl_2O_2$: 259.0287. Found: 259.0284.

(*R*)-1-(4-methylchroman-4-yl)propan-2-one (1g)

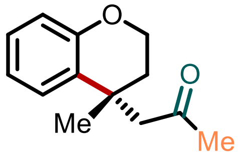

1g

The desired product was isolated by flash chromatography (Isolera Biotage) with 10% EtOAc and was obtained as a colorless oil. **Rf** in 10% of EtOAc in hexanes: 0.29.

The enantiomeric ratio was determined by HPLC analysis using the following parameters: Daicel Chiralcel OJ-3 column (4.6 mm x 250 mm): 5% iPrOH in Hexane (1.0 mL/min) as mobile phase at 25 ºC. (rt = 12.0 min (major), 19.7 min (minor)).

GP1: 57% (11.6 mg), 79:21 *er*; $[\alpha]_D^{25}$ +19 (c 2.00, $CHCl_3$).

GP2: 59% (12.1 mg), 54:46 *er*; $[\alpha]_D^{25}$ +8 (c 2.00, $CHCl_3$).

^{1}H NMR (500 MHz, $CDCl_3$) δ 7.21 (dd, *J* = 7.8, 1.6 Hz, 1H), 7.09 (td, *J* = 1.5, 8 Hz, 1H), 6.88 (td, *J* = 1.5, 8 Hz, 1H), 6.80 (dd, *J* = 8.2, 1.3 Hz, 1H), 4.25 – 4.11 (m, 2H), 2.80 (d, *J* = 1.2 Hz, 2H), 2.22 (ddd, *J* = 14.1, 7.9, 3.5 Hz, 1H), 2.03 (s, 3H), 1.92 (ddd, *J* = 14.1, 7.2, 3.3 Hz, 1H), 1.45 (s, 3H).

^{13}C{^{1}H} NMR (126 MHz, $CDCl_3$) δ 207.6, 153.9, 129.9, 127.6, 126.6, 120.5, 117.3, 62.8, 54.5, 34.1, 33.2, 32.2, 28.7.

HRMS (ESI-Q-Orbitrap) m/z: $[M+H]^+$ Calculated for $C_{13}H_{17}O_2$: 205.1223. Found: 205.1220.

(*R*)-1-(3-methyl-2,3-dihydrobenzofuran-3-yl)hexan-2-one (1h)

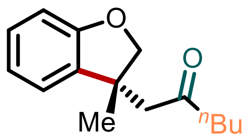

1h

The desired product was isolated by Biotage Isolera with 10% EtOAc and was obtained as a colorless oil. **Rf** in 8% of EtOAc in hexanes: 0.38.

The enantiomeric ratio was determined by HPLC analysis using the following parameters: Daicel Chiralcel OJ-3 column (4.6 mm x 250 mm): 5% iPrOH in Hexane (1.0 mL/min) as mobile phase at 25 ºC. (rt = 5.7 min (major), 7.9 min (minor)).

GP1: 35% (8.1mg), 98:2 *er*; $[\alpha]_D^{25}$ +11 (c 1.00, $CHCl_3$).

GP2: 57%, (13.3 mg), 81:19 *er*; $[\alpha]_D^{25}$ +8 (c 1.00, $CHCl_3$).

^{1}H NMR (600 MHz, $CDCl_3$) δ 7.13 (td, *J* = 7.7, 1.4 Hz, 1H), 7.10 (dd, *J* = 7.4, 1.4 Hz, 1H), 6.87 (td, *J* = 7.4, 1.0 Hz, 1H), 6.80 (d, *J* = 7.9 Hz, 1H), 4.47 (d, *J* = 9.2 Hz, 1H), 4.42 (d, *J* = 9.2 Hz, 1H), 2.93 (d, *J* = 17.2 Hz, 1H), 2.71 (d, *J* = 17.2 Hz, 1H), 2.36 – 2.29 (m, 2H), 1.55 – 1.49 (m, 2H), 1.40 (s, 3H) 1.28 (h, *J* = 7.4 Hz, 2H), 0.88 (t, *J* = 7.3 Hz, 3H).

^{13}C{^{1}H} NMR (151 MHz, $CDCl_3$) δ 209.3, 159.0, 134.9, 128.4, 122.7, 120.5, 109.9, 82.6, 52.0, 43.6, 43.6, 25.8, 25.2, 22.3, 13.8.

HRMS (ESI-Q-Orbitrap) m/z: $[M+Na]^+$ Calculated for $C_{15}H_{20}O_2Na$: 255.1355. Found: 255.1351.

(*R*)-1-(3,6-dimethyl-2,3-dihydrobenzofuran-3-yl)hexan-2-one (1i)

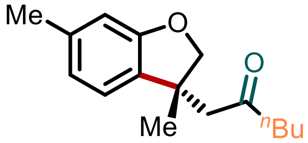

1i

The desired product was isolated by Biotage Isolera with 10% EtOAc in hexanes and was obtained as a colorless oil.

The enantiomeric ratio was determined by HPLC analysis using the following parameters: Daicel Chiralcel IC column (4.6 mm x 250 mm): 5% iPrOH in Hexane (1.0 mL/min) as mobile phase at 25 ºC. (rt = 6.70 min (major), 7.12 min (minor) for the acetone reaction, and 8% iPrOH in Hexane (1.0 mL/min) as mobile phase at 25 ºC. (rt = 5.3 min (major), 5.6 min (minor) for DMF)).

GP1: 25% (6.1 mg), 98:2 *er*; $[\alpha]_D^{25}$ +35 (c 0.2, $CHCl_3$).

GP2: 18% (4.4 mg), 92:8 *er*; $[\alpha]_D^{25}$ +42 (c 1.00, $CHCl_3$).

^{1}H NMR (500 MHz, $CDCl_3$) δ 6.97 (d, *J* = 7.5 Hz, 1H), 6.69 (d, *J* = 7.5 Hz, 1H), 6.62 (s, 1H), 4.45 (d, *J* = 9.2 Hz, 1H), 4.40 (d, *J* = 9.2 Hz, 1H), 2.91 (d, *J* = 17.1 Hz, 1H), 2.69 (d, *J* = 17.1 Hz, 1H), 2.34-2.30 (m, 5H), 1.52 (quint, *J* = 7.9 Hz, 2H), 1.38 (s, 3H), 1.27 (sext, *J* = 7.6 Hz, 2H), 0.88 (t, *J* = 7.0 Hz, 3H).

^{13}C{^{1}H} NMR (126 MHz, $CDCl_3$) δ 209.6, 159.4, 138.8, 132.2, 122.4, 121.4, 110.7, 83.0, 52.2, 43.8, 43.5, 26.0, 25.4, 22.4, 21.6, 14.0.

HRMS (ESI-Q-Orbitrap) m/z: $[M+H]^+$ Calculated for $C_{16}H_{23}O_2$: 247.1698. Found: 247.1692.

(*R*)-1-(7-bromo-3-methyl-2,3-dihydrobenzofuran-3-yl)hexan-2-one (1j)

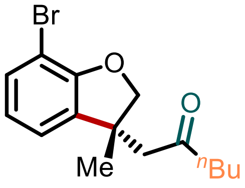

1j

The desired product was isolated by flash column chromatography from 2 to 20% of EtOAc and was obtained as a colorless oil. **Rf** in 7% EtOAc: 0.23

The enantiomeric ratio was determined by HPLC analysis using the following parameters: Daicel Chiralcel OJ-3 column (4.6 mm x 250 mm): 5% iPrOH in Hexane (1.0 mL/min) as mobile phase at 30 ºC. (rt = 8.1 min (major), 10.1 min (minor)).

GP1: < 5%, estimated.

GP2: 21% (6.5 mg), 85:15 *er*; $[\alpha]_D^{25}$ -2 (c 1.00, $CHCl_3$).

^{1}H NMR (600 MHz, $CDCl_3$) δ 7.29 (dd, *J* = 8.0, 1.2 Hz, 1H), 7.03 (dd, *J* = 7.4, 1.2 Hz, 1H), 6.79 – 6.74 (m, 1H), 4.56 (d, J = 9.4 Hz, 1H), 4.53 (d, J = 9.4 Hz, 1H), 2.93 (d, *J* = 17.5 Hz, 1H), 2.74 (d, *J* = 17.5 Hz, 1H), 2.35 (t, *J* = 7.4 Hz, 2H), 1.53 (p, *J* = 7.5 Hz, 2H), 1.41 (s, 3H), 1.28 (dt, *J* = 14.9, 7.5 Hz, 2H), 0.89 (t, *J* = 7.3 Hz, 3H).

^{13}C{^{1}H} NMR (151 MHz, $CDCl_3$) δ 208.8, 156.2, 136.4, 131.4, 122.0, 121.8, 103.0, 83.1, 51.9, 44.7, 43.5, 25.8, 25.2, 22.3, 13.8.

HRMS (ESI-Q-Orbitrap) m/z: $[M+H]^+$ Calculated for $C_{15}H_{20}BrO_2$: 311.0641. Found: 311.0635.

(*R*)-2-(3-methyl-2,3-dihydrobenzofuran-3-yl)-1-phenylethan-1-one (1k)

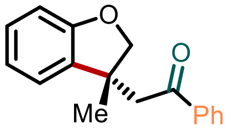

1k

The desired product was isolated by flash column chromatography from 1 to 20% of EtOAc in hexanes and was obtained as a colorless oil. **Rf** in 8% of EtOAc in hexanes: 0.22.

The enantiomeric ratio was determined by HPLC analysis using the following parameters: Daicel Chiralcel OJ-3 column (4.6 mm x 250 mm): 5% iPrOH in Hexane (1.0 mL/min) as mobile phase at 25 ºC. (rt = 12.0 min (major), 17.0 min (minor)).

GP1: 58% (14.6 mg), 99:1 *er*; $[\alpha]_D^{25}$ +58 (c 2.00, $CHCl_3$).

GP2: 54% (13.6 mg), 92:8 *er*; $[\alpha]_D^{25}$ +16 (c 1.00, $CHCl_3$).

The byproduct generated by the direct alkyl insertion was obtained in 22% (4.9 mg), in 10.5:89:5 *er*.

Byproduct $[\alpha]^{20}_D$ = -7 (c 1.00, $CHCl_3$).

^{1}H NMR (500 MHz, $CDCl_3$) δ 7.92 (d, *J* = 7.1 Hz, 2H), 7.56 (t, *J* = 7.4 Hz, 1H), 7.45 (t, *J* = 7.8 Hz, 2H), 7.18 – 7.11 (m, 2H), 6.89 (td, *J* = 7.4, 1.0 Hz, 1H), 6.82 (d, *J* = 7.9 Hz, 1H), 4.58 (d, J = 9.3 Hz, 1H), 4.54 (d, J = 9.4 Hz, 1H), 3.58 (d, *J* = 17.4 Hz, 1H), 3.25 (d, *J* = 17.4 Hz, 1H), 1.50 (s, 3H).

^{13}C{^{1}H} NMR (126 MHz, $CDCl_3$) δ 198.1, 159.0, 137.2, 135.3, 133.3, 128.7, 128.5, 128.0, 122.8, 120.6, 109.9, 82.8, 47.9, 43.8, 25.3.

HRMS (ESI-Q-Orbitrap) m/z: $[M+Na]^+$ Calculated for $C_{17}H_{16}O_2Na$: 275.1042. Found: 275.1038.

(*R*)-2-(3,6-dimethyl-2,3-dihydrobenzofuran-3-yl)-1-phenylethan-1-one (1l)

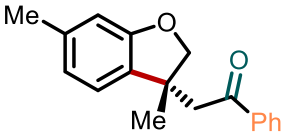

1l

The desired product was isolated by flash column chromatography from 1 to 10% of EtOAc in hexanes and was obtained as a yellowish oil. **Rf** in 8% of EtOAc in hexanes: 0.22.

The enantiomeric ratio was determined by HPLC analysis using the following parameters: Daicel Chiralcel OJ-3 column (4.6 mm x 250 mm): 5% iPrOH in Hexane (1.0 mL/min) as mobile phase at 25 ºC. (rt = 12.8 min (major), 18.2 min (minor)).

GP1: 56% (14.9 mg), 98:2 *er*; $[\alpha]_D^{25}$ +32 (c 2.00, $CHCl_3$).

GP2: 36% (9.6 mg), 87:13 *er*; $[\alpha]_D^{25}$ +28 (c 2.00, $CHCl_3$).

The byproduct generated by the direct alkyl insertion was obtained in 19% (4.65 mg).

^{1}H NMR (500 MHz, $CDCl_3$) δ 7.95 – 7.89 (m, 2H), 7.56 (t, *J* = 7.4 Hz, 1H), 7.45 (t, *J* = 7.8 Hz, 2H), 7.05 (d, *J* = 7.5 Hz, 1H), 6.71 (d, *J* = 7.5 Hz, 1H), 6.65 (s, 1H), δ 4.56 (d, J = 9.3 Hz, 1H), 4.53 (d, J = 9.3 Hz, 1H), 3.56 (d, *J* = 17.4 Hz, 1H), 3.22 (d, *J* = 17.4 Hz, 1H), 2.31 (s, 3H), 1.47 (s, 3H).

^{13}C{^{1}H} NMR (126 MHz, $CDCl_3$) δ 198.2, 159.3, 138.7, 137.3, 133.2, 132.5, 128.6, 128.0, 122.4, 121.3, 110.6, 83.0, 48.0, 43.6, 25.3, 21.5.

HRMS (ESI-Q-Orbitrap) m/z: $[M+Na]^+$ Calculated for $C_{18}H_{18}O_2Na$: 289.1199. Found: 289.1193

(*R*)-2-(3-methyl-5-nitro-2,3-dihydrobenzofuran-3-yl)-1-phenylethan-1-one (1m)

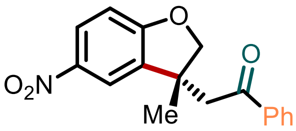

1m

The desired product was isolated by Biotage Isolera with 10% EtOAc in hexanes and was obtained as a slightly yellowish oil.

The enantiomeric ratio was determined by HPLC analysis using the following parameters: Daicel Chiralcel IC column (4.6 mm x 250 mm): 10% iPrOH in Hexane (1.0 mL/min) as mobile phase at 25 ºC. (rt = 16.5 min (minor), 18.4 min (major)).

GP1: 21% (6.4 mg), 97:3 *er*; $[\alpha]_D^{25}$ +107 (c 1.00, $CHCl_3$).

GP2: 39% (11.7 mg), 90:10 *er*; $[\alpha]_D^{25}$ +59 (c 0.50, $CHCl_3$).

^{1}H NMR (600 MHz, CDC13) δ 8.14 (dd, *J* = 8.8, 2.4 Hz, 1H), 8.08 (d, *J* = 2.4 Hz, 1H), 7.96 – 7.92 (m, 2H), 7.61 – 7.57 (m, 1H), 7.51 – 7.45 (m, 2H), 6.88 – 6.84 (m, 1H), 4.76 (d, *J* = 9.7 Hz, 1H), 4.74 (d, *J* = 9.6 Hz, 1H), 3.66 (d, *J* = 17.7 Hz, 1H), 3.32 (d, *J* = 17.7 Hz, 1H), 1.53 (s, 3H).

^{13}C{^{1}H} NMR (151 MHz, CDC13) δ 197.3, 164.8, 142.2, 139.9, 136.8, 133.8, 128.9, 128.1, 126.3, 119.6, 110.0, 84.9, 47.9, 43.5, 26.3.

HRMS (ESI-Q-Orbitrap) m/z: $[M+Na]^+$ Calculated for $C_{17}H_{15}NNaO_4$: 320.0898. Found: 320.0893.

(*R*)-1-(3-benzyl-2,3-dihydrobenzofuran-3-yl)propan-2-one (1n)

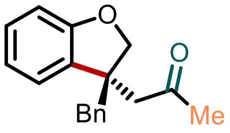

1n

The desired product was isolated by Biotage Isolera with 10% EtOAc and was obtained as a brown oil. **Rf** in 10% of EtOAc in hexanes: 0.25.

The enantiomeric ratio was determined by HPLC analysis using the following parameters: Daicel Chiralcel OJ-3 column (4.6 mm x 250 mm): 5% iPrOH in Hexane (1.0 mL/min) as mobile phase at 30 ºC. (rt = 13.2 min (major), 20.0 min (minor)).

GP1: 60% (16.0 mg), 98:2 *er*; $[\alpha]_D^{25}$ +94 (c 2.00, $CHCl_3$).

GP2: 65% (17.2 mg), 89:11 *er*; $[\alpha]_D^{25}$ +37 (c 2.00, $CHCl_3$).

^{1}H NMR (500 MHz, $CDCl_3$) δ 7.23 (dd, *J* = 5.0, 1.8 Hz, 3H), 7.19 – 7.13 (m, 1H), 6.89 – 6.76 (m, 5H), 4.79 (d, *J* = 9.4 Hz, 1H), 4.32 (d, *J* = 9.4 Hz, 1H), 3.14 – 3.05 (m, 3H), 2.77 – 2.69 (m, 1H), 2.16 (s, 3H).

^{13}C{^{1}H} NMR (126 MHz, $CDCl_3$) δ 207.0, 159.5, 137.1, 132.3, 130.5, 128.7, 128.0, 126.6, 124.4, 120.0, 109.9, 81.6, 50.2, 47.8, 43.3, 30.9.

HRMS (ESI-Q-Orbitrap) m/z: $[M+H]^+$ Calculated for $C_{18}H_{19}O_2$: 267.1379. Found: 267.1375.

(*R*)-3-benzyl-5-methoxy-3-methyl-2,3-dihydrobenzofuran (1o)

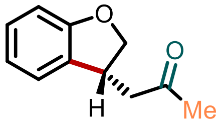

1o

The desired product was isolated by Biotage Isolera with 3% EtOAc in hexanes and was obtained as a colorless oil. **Rf** in 10% of EtOAc in hexanes: 0.20.

The enantiomeric ratio was determined by HPLC analysis using the following parameters: Daicel Chiralcel OJ-3 column (4.6 mm x 250 mm): 10% iPrOH in Hexane (1.0 mL/min) as mobile phase at 25 ºC. (rt = 10.8 min (major), 14.0 min (minor)).

GP1: 20% (15.0 mg), 97:3 *er*; $[\alpha]_D^{25}$ +15 (c 2.00, $CHCl_3$).

^{1}H NMR (300 MHz, $CDCl_3$) δ 7.20 – 7.10 (m, 2H), 6.92 – 6.78 (m, 2H), 4.81 (t, J = 9.1 Hz, 1H), 4.20 – 4.08 (m, 1H), 3.89 (ddd, *J* = 14.6, 9.3, 5.7 Hz, 1H), 3.02 (dd, *J* = 6, 18 Hz), 2.77 (dd, *J* = 9, 18 Hz), 2.21 (s, 3H).

^{13}C{^{1}H} NMR (75 MHz, $CDCl_3$) δ 206.9, 159.8, 129.5, 128.5, 124.2, 120.5, 109.7, 60.4, 49.2, 37.1, 30.2.

HRMS (ESI-Q-Orbitrap) m/z: $[M+H]^+$ Calculated for $C_{11}H_{13}O_2$: 177.0910. Found: 177.0909.

(*R*)-3-ethyl-3-methyl-2,3-dihydrobenzofuran (2a)

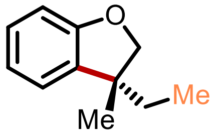

2a

The desired product was isolated by preparative TLC (5% of EtOAc in hexanes as mobile phase) due to the high volatility of the compound. **Rf** in 8% of EtOAc in hexanes: 0.62.

The enantiomeric ratio was determined by HPLC analysis using the following parameters: Daicel Chiralcel OJ-3 column (4.6 mm x 250 mm): 5% iPrOH in Hexane (1.0 mL/min) as mobile phase at 25 ºC. (rt = 4.884 min (major), 4.589 min (minor)).

GP3: Estimated yield using 29.3 mg of 1-bromo-3,5-bis(trifluoromethyl)benzene as internal standard of 10%, 99:1 *er*; $[\alpha]_D^{25}$ -4 (c 0.5, $CHCl_3$).

GP4: Estimated yield using 29.3 mg of 1-bromo-3,5-bis(trifluoromethyl)benzene as internal standard of 47%, 86.5:13.5 *er*; $[\alpha]_D^{25}$ -6 (c 1.00, $CHCl_3$).

^{1}H NMR (500 MHz, $CDCl_3$) δ 7.10 – 7.04 (m, 1H), 7.02 (dd, *J* = 7.3, 1.5 Hz, 1H), 6.82 (td, *J* = 7.4, 1.0 Hz, 1H), 6.73 (dd, *J* = 8.1, 0.6 Hz, 1H), 4.30 (d, *J* = 8.5 Hz, 1H), 4.10 (d, *J* = 8.5 Hz, 1H), 1.61 (d, *J* = 7.5 Hz, 1H), 1.58 (d, *J* = 7.5 Hz, 1H), 1.27 (s, 3H), 0.79 (t, *J* = 7.5 Hz, 3H).

^{13}C{^{1}H} NMR (126 MHz, $CDCl_3$) δ 159.6, 135.1, 127.9, 122.9, 120.4, 109.5, 82.2, 45.6, 33.4, 25.1, 9.0.

(*R*)-3-ethyl-3,6-dimethyl-2,3-dihydrobenzofuran (2b)

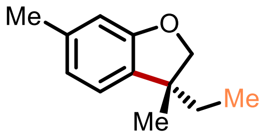

2b

The desired product was isolated by Biotage Isolera with 2% EtOAc in hexanes and was obtained as a colorless oil. **Rf** in 2% of EtOAc in hexanes: 0.23.

The enantiomeric ratio was determined by HPLC analysis using the following parameters: Daicel Chiralcel OJ-3 column (4.6 mm x 250 mm): 5% iPrOH in Hexane (1.0 mL/min) as mobile phase at 25 ºC. (rt = 4.697 min (major), 5.415 min (minor)).

GP3: <5%.

GP4: 62% (10.9 mg), 89:11 *er*; $[\alpha]_D^{25}$ -3 (c 1.00, $CHCl_3$).

^{1}H NMR (300 MHz, $CDCl_3$) δ 6.97 (d, *J* = 7.5 Hz, 1H), 6.71 (d, *J* = 7.5 Hz, 1H), 6.63 (s, 1H), 4.36 (d, *J* = 8.6 Hz, 1H), 4.16 (d, *J* = 8.6 Hz, 1H), 2.33 (s, 3H), 1.65 (q, *J* = 7.6 Hz, 2H), 1.33 (s, 3H), 0.86 (t, *J* = 7.5 Hz, 3H).

^{13}C{^{1}H} NMR (75 MHz, $CDCl_3$) δ 159.9, 138.1, 132.2, 122.5, 121.0, 110.2, 82.5, 45.3, 33.4, 25.2, 21.5, 9.0.

HRMS (ESI-Q-Orbitrap) m/z: $[M+H]^+$ Calculated for $C_{12}H_{17}O$: 177.1273. Found: 177.1272.

(*R*)-3-ethyl-5-methoxy-3-methyl-2,3-dihydrobenzofuran (2c)

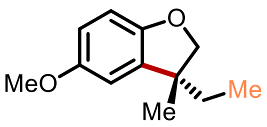

2c

The desired product was isolated by Biotage Isolera with 5% EtOAc in hexanes and was obtained as a colorless oil.

The enantiomeric ratio was determined by HPLC analysis using the following parameters: Daicel Chiralcel OJ-3 column (4.6 mm x 250 mm): 2% iPrOH in Hexane (1.0 mL/min) as mobile phase at 25 ºC. (rt = 5.8 min (minor), 5.5 min (major)).

GP3: 54% (10.3 mg), 97:3 *er*; $[\alpha]_D^{25}$ -7 (c 1.00, $CHCl_3$).

GP4: 67% (12.6 mg), 90:10 *er*; $[\alpha]_D^{25}$ -4 (c 1.00, $CHCl_3$).

^{1}H NMR (400 MHz, $CDCl_3$) δ 6.72-6.67 (m, 3H), 4.36 (d, *J* = 8.6 Hz, 1H), 4.16 (d, *J* = 8.6 Hz, 1H), 3.79 (s, 3H), 1.66 (q, *J* = 7.5 Hz, 2H), 1.33 (s, 3H), 0.87 (t, *J* = 7.5 Hz, 3H).

^{13}C{^{1}H} NMR (101 MHz, $CDCl_3$) δ 154.2, 153.7, 136.3, 112.4, 109.5, 109.3, 82.4, 56.0, 46.0, 33.2, 24.9, 8.9.

HRMS (ESI-Q-Orbitrap) m/z: $[M+H]^+$ Calculated for $C_{12}H_{17}O_2$: 193.1228. Found: 193.1223.

(*R*)-3-benzyl-5-methoxy-3-methyl-2,3-dihydrobenzofuran (2d)

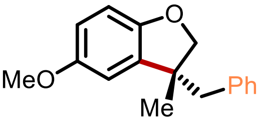

2d

The desired product was isolated by Biotage Isolera with 3% EtOAc in hexanes and was obtained as a colorless oil. **Rf** in 3% of EtOAc in hexanes: 0.23.

The enantiomeric ratio was determined by HPLC analysis using the following parameters: Daicel Chiralcel OJ-3 column (4.6 mm x 250 mm): 5% iPrOH in Hexane (1.0 mL/min) as mobile phase at 25 ºC. (rt = 7.9 min (minor), 11.1 min (major)).

GP3: 59% (15.0 mg), 98:2 *er*; $[\alpha]_D^{25}$ +22 (c 2.00, $CHCl_3$).

GP4: 64% (16.4 mg), 74:26 *er*; $[\alpha]_D^{25}$ +28 (c 2.00, $CHCl_3$).

^{1}H NMR (600 MHz, $CDCl_3$) δ 7.19 – 7.12 (m, 3H), 6.94 (dd, *J* = 7.9, 1.7 Hz, 2H), 6.60 (d, *J* = 1.6 Hz, 2H), 6.41 (t, *J* = 1.6 Hz, 1H), 4.40 (d, *J* = 8.6 Hz, 1H), 3.97 (d, *J* = 8.6 Hz, 1H), 3.65 (s, 3H), 2.82 (d, *J* = 13.4 Hz, 1H), 2.78 (d, *J* = 13.3 Hz, 1H), 1.26 (s, 3H).

^{13}C{^{1}H} NMR (151 MHz, $CDCl_3$) δ 154.0, 153.6, 137.5, 135.9, 130.4, 128.0, 126.5, 113.2, 109.6, 109.6, 82.3, 56.0, 46.7, 46.4, 24.3.

HRMS (ESI-Q-Orbitrap) m/z: $[M+H]^+$ Calculated for $C_{17}H_{19}O_2$: 255.1379. Found: 255.1379.

(*R*)-3-benzyl-3-methyl-2,3-dihydrobenzofuran (2e)

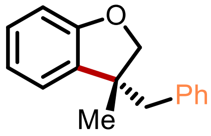

2e

The desired product was isolated by Biotage Isolera with 10% EtOAc and was obtained as a colorless oil. **Rf** in 2% of EtOAc in hexanes: 0.38.

The enantiomeric ratio was determined by HPLC analysis using the following parameters: Daicel Chiralcel OJ-3 column (4.6 mm x 250 mm): 5% iPrOH in Hexane (1.0 mL/min) as mobile phase at 25 ºC. (rt = 8.887 min (major), 7.873 min (minor)).

GP3: 91% (20.4 mg), 98:2 *er*; $[\alpha]_D^{25}$ -12 (c 1.00, $CHCl_3$).

GP4: 91% (20.4 mg), 74:26 *er*; $[\alpha]_D^{25}$ -9 (c 1.00, $CHCl_3$); 1 mmol scale: 82% (183.9 mg).

^{1}H NMR (600 MHz, $CDCl_3$) δ 7.26 – 7.21 (m, 3H), 7.15 – 7.11 (m, 1H), 7.00 (dd, *J* = 7.6, 1.8 Hz, 2H), 6.94 (dd, *J* = 7.4, 1.4 Hz, 1H), 6.86 (td, *J* = 7.4, 1.0 Hz, 1H), 6.76 (d, *J* = 8.0 Hz, 1H), 4.50 (d, *J* = 8.7 Hz, 1H), 4.06 (d, *J* = 8.6 Hz, 1H), 2.90 (d, *J* = 13.3 Hz, 1H), 2.86 (d, *J* = 13.3 Hz, 1H), 1.35 (s, 3H).

^{13}C{^{1}H} NMR (151 MHz, $CDCl_3$) δ 159.5, 137.6, 134.8, 130.4, 128.2, 127.9, 126.5, 123.4, 120.3, 109.7, 81.9, 46.6, 46.3, 24.6.

Spectral data match with those previously reported in the literature[3]

(*R*)-3-benzyl-3,6-dimethyl-2,3-dihydrobenzofuran (2f)

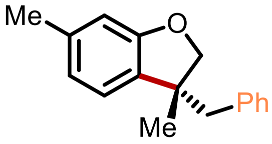

2f

The desired product was isolated by Biotage Isolera with 5% EtOAc in hexanes and was obtained as a colorless oil. **Rf** in 5% of EtOAc in hexanes: 0.56.

The enantiomeric ratio was determined by HPLC analysis using the following parameters: Daicel Chiralcel OJ-3 column (4.6 mm x 250 mm): 5% iPrOH in Hexane (1.0 mL/min) as mobile phase at 25 ºC. (rt = 7.957 min (major), 6.609 min (minor)).

GP3: 47% (11.2 mg), 98:2 *er*; $[\alpha]_D^{25}$ +3 (c 0.5, $CHCl_3$).

GP4: 71% (16.8 mg), 70:30 *er*; $[\alpha]_D^{25}$ +2 (c 1.00, $CHCl_3$).

^{1}H NMR (300 MHz, $CDCl_3$) δ 7.32 – 7.23 (m, 3H), 7.10 – 7.02 (m, 2H), 6.85 (d, *J* = 7.5 Hz, 1H), 6.71 (d, *J* = 7.6 Hz, 1H), 6.64 (s, 1H), 4.52 (d, *J* = 8.7 Hz, 1H), 4.08 (d, *J* = 8.7 Hz, 1H), 2.93 (d, *J* = 12 Hz, 1H), 2.87 (d, *J* = 12 Hz, 1H), 2.35 (s, 3H), 1.36 (s, 3H).

^{13}C{^{1}H} NMR (75 MHz, $CDCl_3$) δ 159.8, 138.4, 137.7, 132.0, 130.4, 127.9, 126.4, 122.9, 121.0, 110.4, 82.2, 46.6, 46.0, 24.7, 21.5.

HRMS (ESI-Q-Orbitrap) m/z: $[M+H]^+$ Calculated for $C_{17}H_{19}O$: 239.1430. Found: 239.1425

(*R*)-3-benzyl-7-bromo-3-methyl-2,3-dihydrobenzofuran (2g)

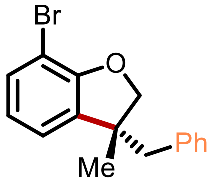

2g

The desired product was isolated by Biotage Isolera with 5% EtOAc in hexanes and was obtained as a colorless oil. **Rf** in 5% of EtOAc in hexanes: 0.51.

The enantiomeric ratio was determined by HPLC analysis using the following parameters: Daicel Chiralcel OJ-3 column (4.6 mm x 250 mm): 5% iPrOH in Hexane (1.0 mL/min) as mobile phase at 25 ºC. (rt = 9.428 min (major), 7.595 min (minor)).

GP3: < 5%.

GP4: 74% (22.5 mg), 64:36 *er*; $[\alpha]_D^{25}$ -16 (c 2.00, $CHCl_3$, 63.5:36.5 *er*).

^{1}H NMR (300 MHz, $CDCl_3$) δ 7.36 – 7.24 (m, 4H), 7.08 – 6.98 (m, 2H), 6.87 (dd, *J* = 7.4, 1.3 Hz, 1H), 6.82 – 6.73 (m, 1H), 4.63 (d, *J* = 8.9 Hz, 1H), 4.19 (d, *J* = 8.8 Hz, 1H), 2.95 (d, *J* = 12 Hz, 1H), 2.89 (d, *J* = 12 Hz, 1H), 1.39 (s, 3H).

^{13}C{^{1}H} NMR (75 MHz, $CDCl_3$) δ 156.6, 137.0, 136.3, 131.2, 130.4, 128.1, 126.7, 122.5, 121.8, 102.8, 82.3, 47.4, 46.5, 24.6.

HRMS (ESI-Q-Orbitrap) m/z: $[M+Na]^+$ Calculated for $C_{16}H_{15}BrONa$: 325.0198. Found: 325.0194

(*R*)-3-benzyl-3-methyl-5-nitro-2,3-dihydrobenzofuran (2h)

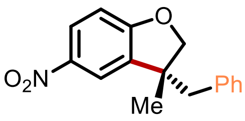

2h

The desired product was isolated by Biotage Isolera with 5% EtOAc in hexanes and was obtained as a colorless oil.

The enantiomeric ratio was determined by HPLC analysis using the following parameters: Daicel Chiralcel IC column (4.6 mm x 250 mm): 10% iPrOH in Hexane (1.0 mL/min) as mobile phase at 25 ºC. (rt = 8.68 min (minor), 9.86 min (major)).

GP3: 22% (5.9 mg), 95:5 *er*; $[\alpha]_D^{25}$ +25 (c 0.5, $CHCl_3$).

GP4: 39% (10.4 mg), 93:7 *er*; $[\alpha]_D^{25}$ +69 (c 1.0, $CHCl_3$).

^{1}H NMR (300 MHz, $CDCl_3$) δ 8.15 (dd, *J* = 8.9, 2.5 Hz, 1H), 7.87 (d, *J* = 2.5 Hz, 1H), 7.34 – 7.21 (m, 3H), 6.99 (dd, *J* = 6.6, 3.1 Hz, 2H), 6.80 (d, *J* = 8.8 Hz, 1H), 4.71 (d, *J* = 9.1 Hz, 1H), 4.27 (d, *J* = 9.1 Hz, 1H), 2.97 (d, *J* = 13.4 Hz, 1H), 2.91 (d, *J* = 13.3 Hz, 1H), 1.46 (s, 3H).

^{13}C{^{1}H} NMR (75 MHz, $CDCl_3$) δ 165.1, 141.9, 136.3, 136.3, 130.2, 128.3, 127.0, 125.9, 120.1, 109.7, 83.6, 46.6, 46.0, 24.9.

HRMS (ESI-Q-Orbitrap) m/z: $[M+H]^+$ Calculated for $C_{16}H_{16}NO_3$: 270.1125. Found: 270.1124.

4.1. Absolute configuration determination for compounds 1a and 2e

Compound 1a

The compound **1a** was derivatized into the corresponding ester following literature procedures[5,6], yielding compound **1p**. This compound can be synthesized using a known literature method, which also establishes its absolute configuration[7].

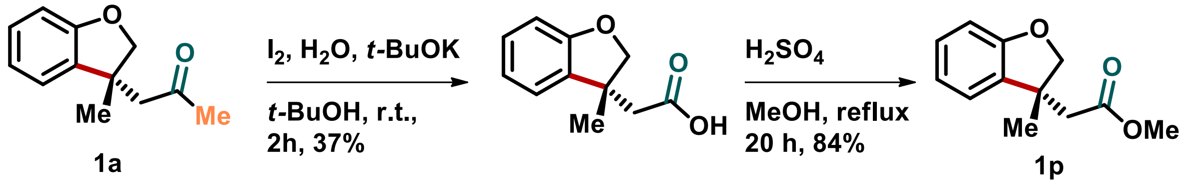

^{1}H NMR (400 MHz, $CDCl_3$) δ 7.16 – 7.11 (m, 2H), 6.90 – 6.88 (m, 1H), 6.82 – 6.80 (m, 1H), 4.62 (d, J = 9.1 Hz, 1H), 4.32 (d, J = 9.1 Hz, 1H), 3.67 (s, 3H), 2.71 (d, J = 15.3 Hz, 1H), 2.64 (d, J = 15.3 Hz, 1H), 1.43 (s, 3H).

^{13}C{^{1}H} NMR (101 MHz, $CDCl_3$) δ 171.7, 159.2, 134.3, 128.7, 122.8, 120.8, 110.1, 82.5, 51.7, 44.3, 43.9, 25.2.

The spectral data of compound **1p** the match those reported in the literature[7].

HPLC Comparison

We compared the HPLC retention times of the major and minor enantiomers of compound 1p obtained using our method with those obtained using the literature procedure.

Conditions: Column OJ-H (4.6 mm x 250 mmL); Mobile phase 10% iPrOH in hexanes

Literature procedure: (rt major = 7.557 min; rt minor = 11.147 min)

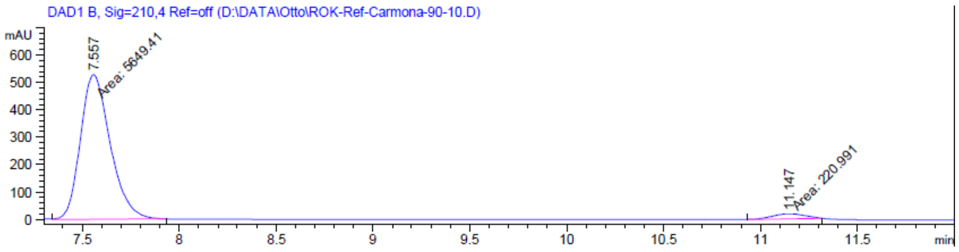

1p: (rt major = 7.547 min; rt minor = 11.082 min)

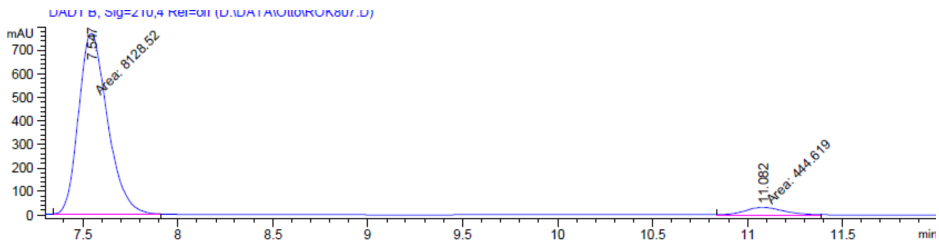

---

5 Kawasumi, R., Narita, S., Miyamoto, K. et al. *Sci Rep, **2017**,* 7, 17967.

6 Mohammadlou, M; Halimehjani, A. Z*; Synthesis* **2024;** 56(14): 2284-2294

7 Carmona, R. C.; Kõster, O. D.; Correia, C. R. D.; *Angew. Chem. Int. Ed*. **2018**, *57*, 12067

Polarimetry Comparison

Literature: $[\alpha]_D^{25}$ +21 (c 1.00, $CHCl_3$, *er* 96:4).

1p: $[\alpha]_D^{25}$ +19 (c 1.00, $CHCl_3$, *er* 95:5).

Compound 2e

We compared the HPLC retention times of the major and minor enantiomers of the compound 2e prepared with our method and a previously described method in the literature by Brown and You[8], which is described in our support information and referenced with proper literature.

Conditions: Column IA (4.6 mm x 250 mmL); Mobile phase 0.2% iPrOH in hexanes

Brown's work: (rt minor = 17.187 min; rt major = 21.424 min)

This work: (rt minor = 15.744 min; rt major = 23.421 min)

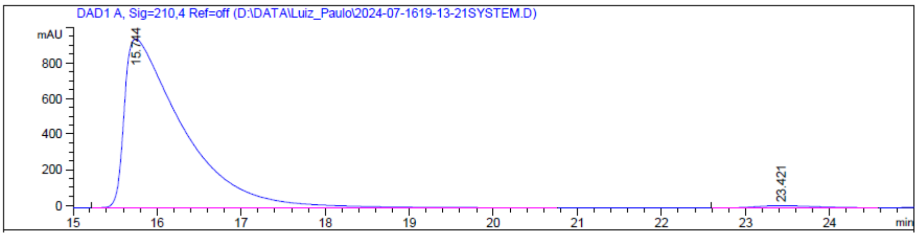

[8] You, W.; Brown, K. J. Am. Chem. Soc. 2015, 137, 46, 14578–14581

4.2. 1mmol scale experiments

The reactions were also performed at a 1 mmol scale to assess their behavior at larger scales. The experimental procedure was identical to the one described earlier for the selected substrates using acetone as solvent, with the exception that for the reaction involving carbon monoxide, the acetone solvent was first saturated with carbon monoxide prior to the reaction.

Product	0.1 mmol	1.0 mmol
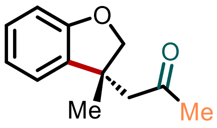	81%	67%
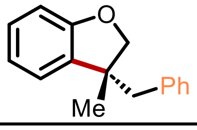	91%	82%

4.3.Triphenylfluorotin ^{1}H, ^{13}C and ^{19}F NMR spectra

When performing reaction **2e**, we noticed a formation of a grey solid. This solid was isolated by filtration and analyzed by ^{1}H, ^{13}C, and ^{19}F NMR spectroscopy in solution using DMSO-d_6 as solvent. It was noticed that the phenyl group was present in this material as shown in ^{1}H NMR (**Figure S4**).

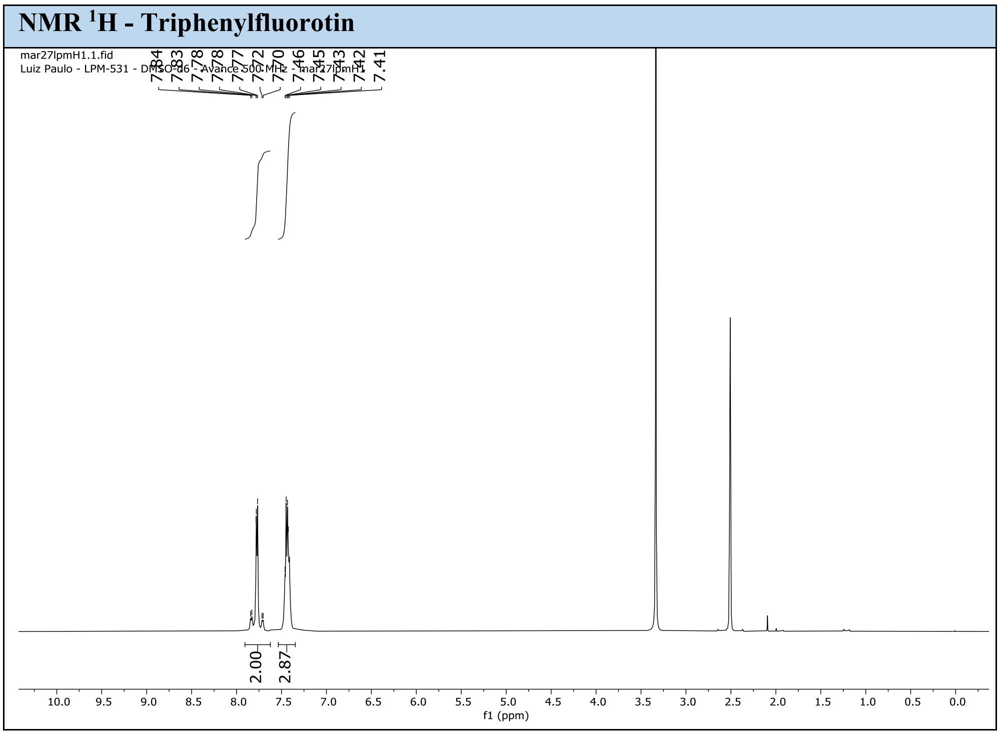

Figure S4. ^{1}H NMR Spectra from the isolated grey solid from the reaction regarding product **2e** (DMSO-d_6).

In the ^{13}C we observe the signals corresponding to the aromatic carbons of the phenyl rings (**Figure S5**).

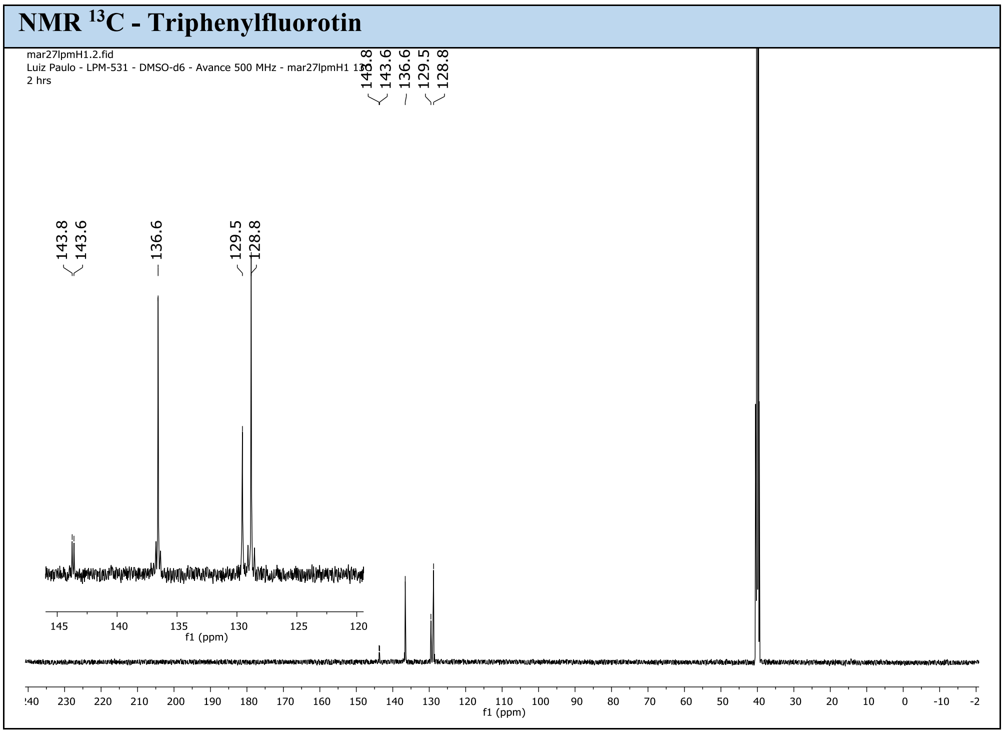

Figure S5. ^{13}C NMR Spectra from the isolated grey solid from the reaction regarding product **2e** (DMSO-d_6).

In the ^{19}F decoupled NMR (**Figure S6**) it was noticed the presence of Fluorine at –174.4 ppm. We observed the presence of two satellite signals corresponding to the Fluorine-Tin coupling ($^{1}J_{^{19}F-^{117}Sn}$) = 2021 Hz and ($^{1}J_{^{19}F-^{119}Sn}$) = 2115 Hz. These NMR data led us to the conclusion that this isolated solid is $SnPh_3F$.

NMR ^{19}F decoupled – Triphenylfluoro tin (470 MHz, DMSO-*d6*)

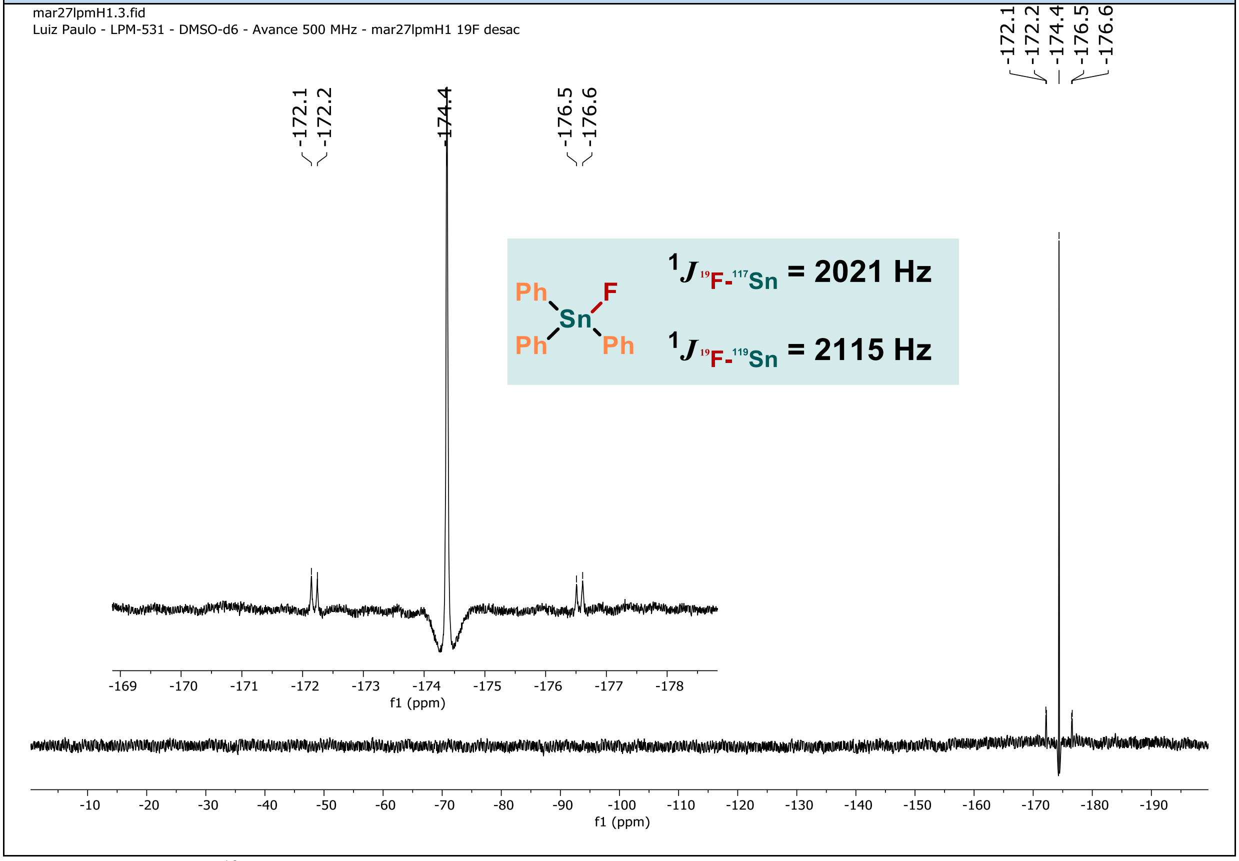

Figure S6. Decoupled ^{19}F NMR Spectra from the isolated grey solid from the reaction regarding product **2e** (DMSO-d_6).

Table S1. Nuclei data for Tin Isotopes

**Tin Isotopes**[9]	^{117}**Sn**	^{119}**Sn**
**Abundance (%)**	7.51	8.58
**Spin**	1/2	1/2
**Gyromagnetic Ratio (γ, 10^7rad.$T^{-1}s^{-1}$)**	-9.578	-10.021

[9] Encyclopedia of Spectroscopy and Spectrometry. Pettinari, C, **1999**. Academic Press.

We compared the BF_4^- ion using ^{19}F NMR spectra with the $SnPh_3F$ isolated from the reaction. There are significant differences between the fluorides present in the BF_4^- ion and $SnPh_3F$. Also, a study of HRMS using the isolated $SnPh_3F$ shows a fragment that corroborates the authenticity of this compound.

1 ^{19}F NMR studies

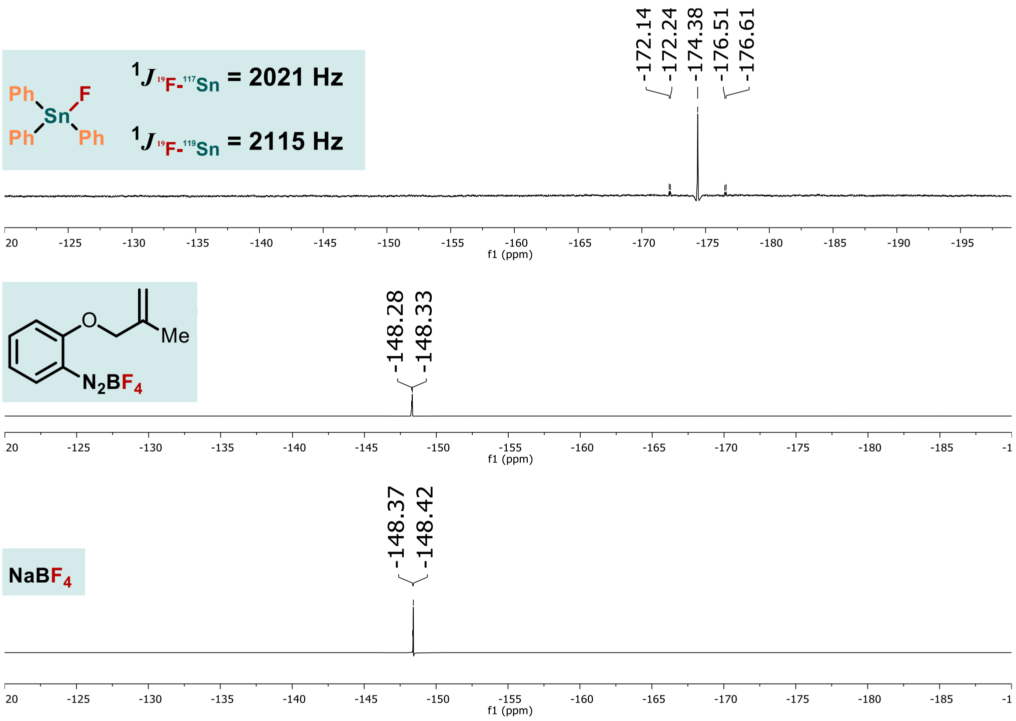

2 HRMS studies

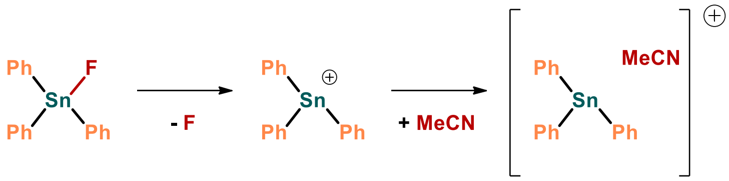

Figure S7. ^{19}F NMR (470 MHz, DMSO-d_6) spectra of Fluoride compounds and ions detected by HRMS.

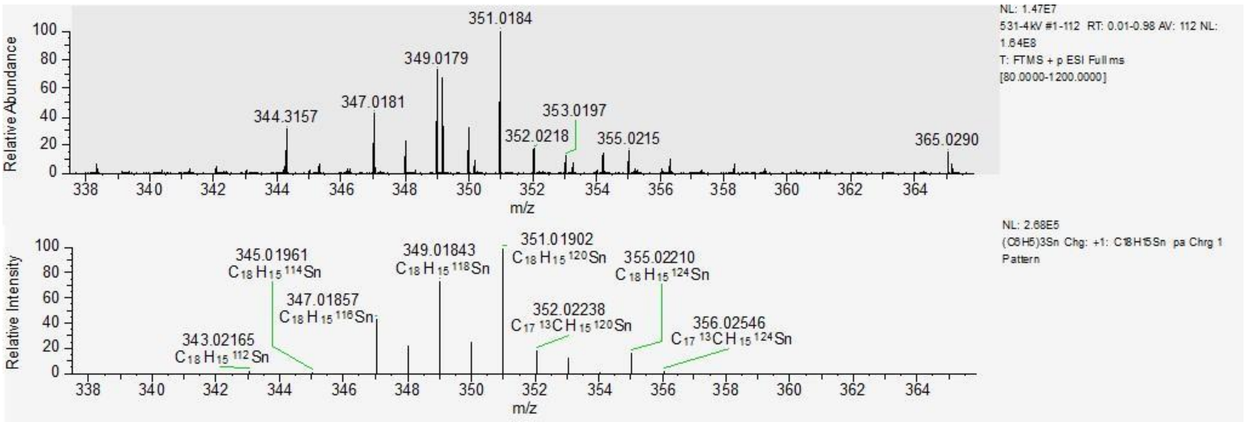

Figure S8. HRMS for the ion $[SnPh_3]^+$.

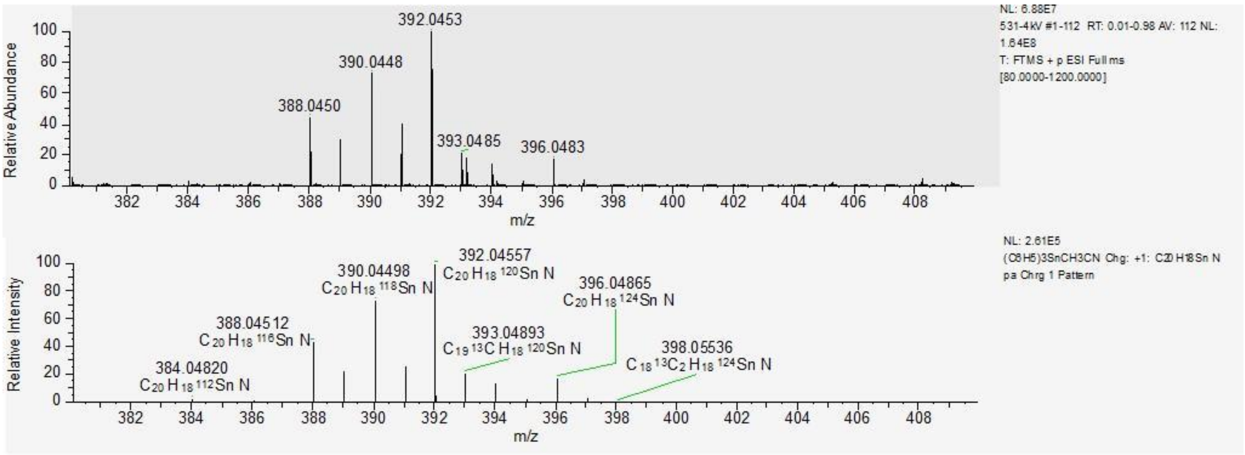

Figure S9. HRMS for the cationic adduct $[SnPh_3+MeCN]^+$.

4.4.Effect of $ZnCO_3$

Table S2. Effect of $ZnCO_3$ on carbonylative Heck-Matsuda-Stille reaction.

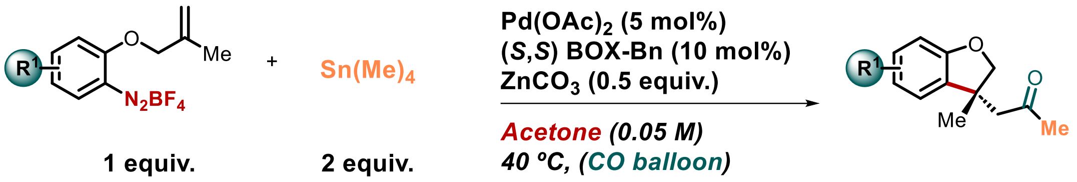

	SM1	SM2	SM3 (Produced mixture of products)		SM7 (Produced mixture of products)	
**Entry**	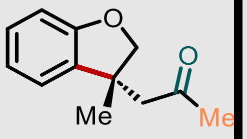	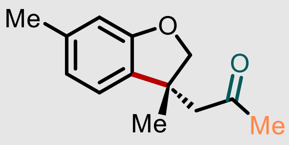	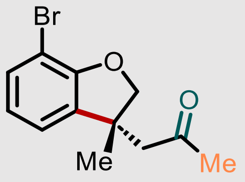	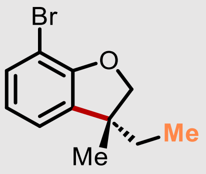	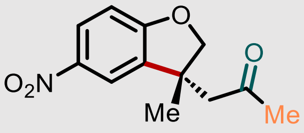	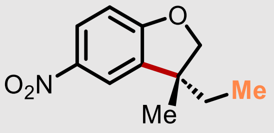
w/ $ZnCO_3$	**91%** **95:5 *er***	**73%** **92:8 *er***	55% 92:8 *er*	40% -	57% 92:8 *er*	25% -
w/o $ZnCO_3$	67% 93:7 *er*	54% 87:12 *er*	54% 92:8 *er*	34% -	**64%** **95:5 *er***	28% -

In this table, in shown results regarding studies using $ZnCO_3$ as Lewis acid in the system. In most of the cases $ZnCO_3$ increased the general yield for carbonylated products and *er*. Substrates containing EWG showed lower selectivity for the carbonylated product. Differently from the other carbonylated products, **1e** resulted in lesser yields when $ZnCO_3$ was used.

5. ^{1}H and $^{13}C\{^{1}H\}$ NMR for the arenediazonium salts – SM1 to SM8.

SM1 – ^{1}H NMR (300 MHz, Acetone-d_6)

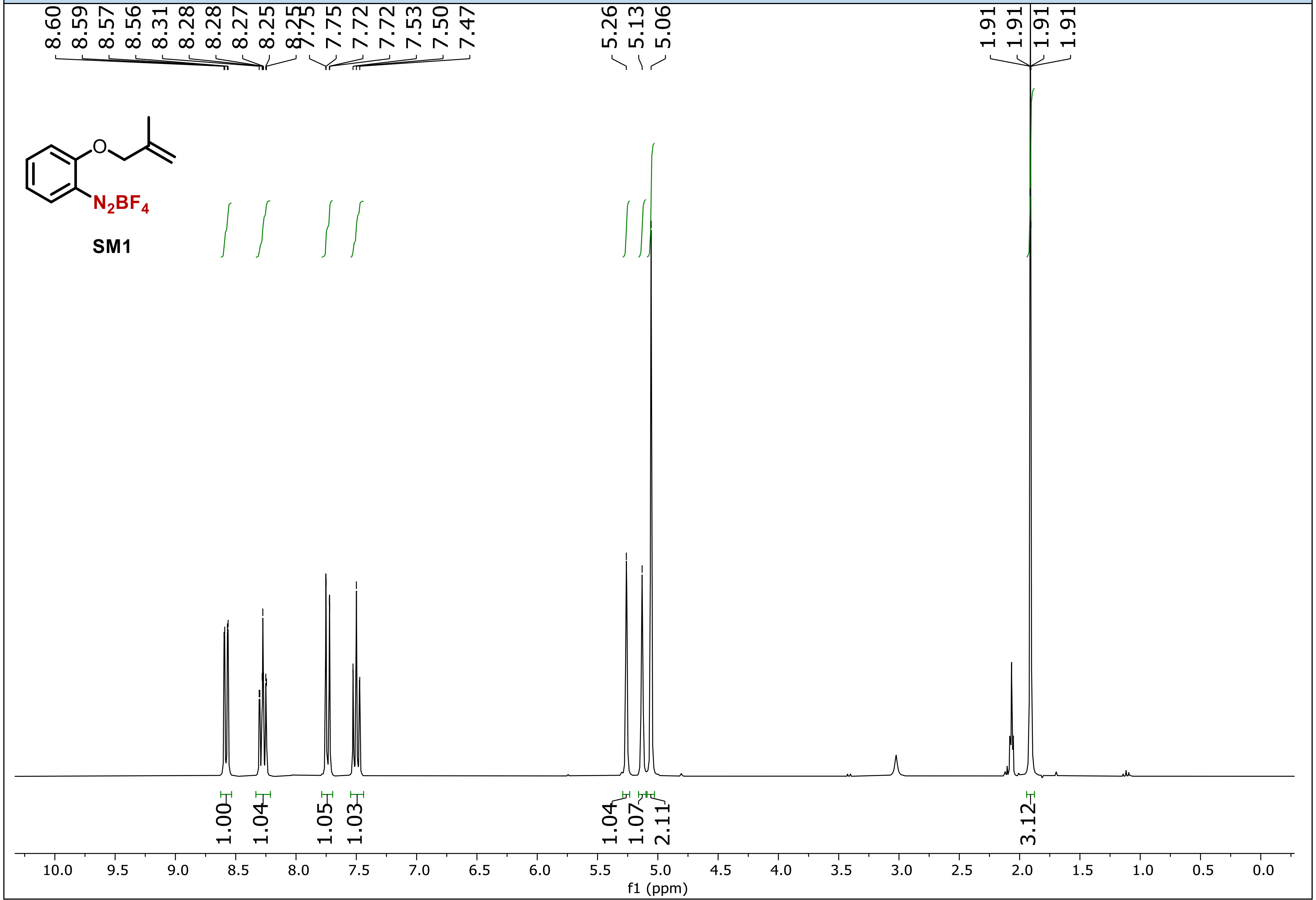

SM1 - $^{13}C\{^1H\}$ NMR (75 MHz, Acetone-d_6)

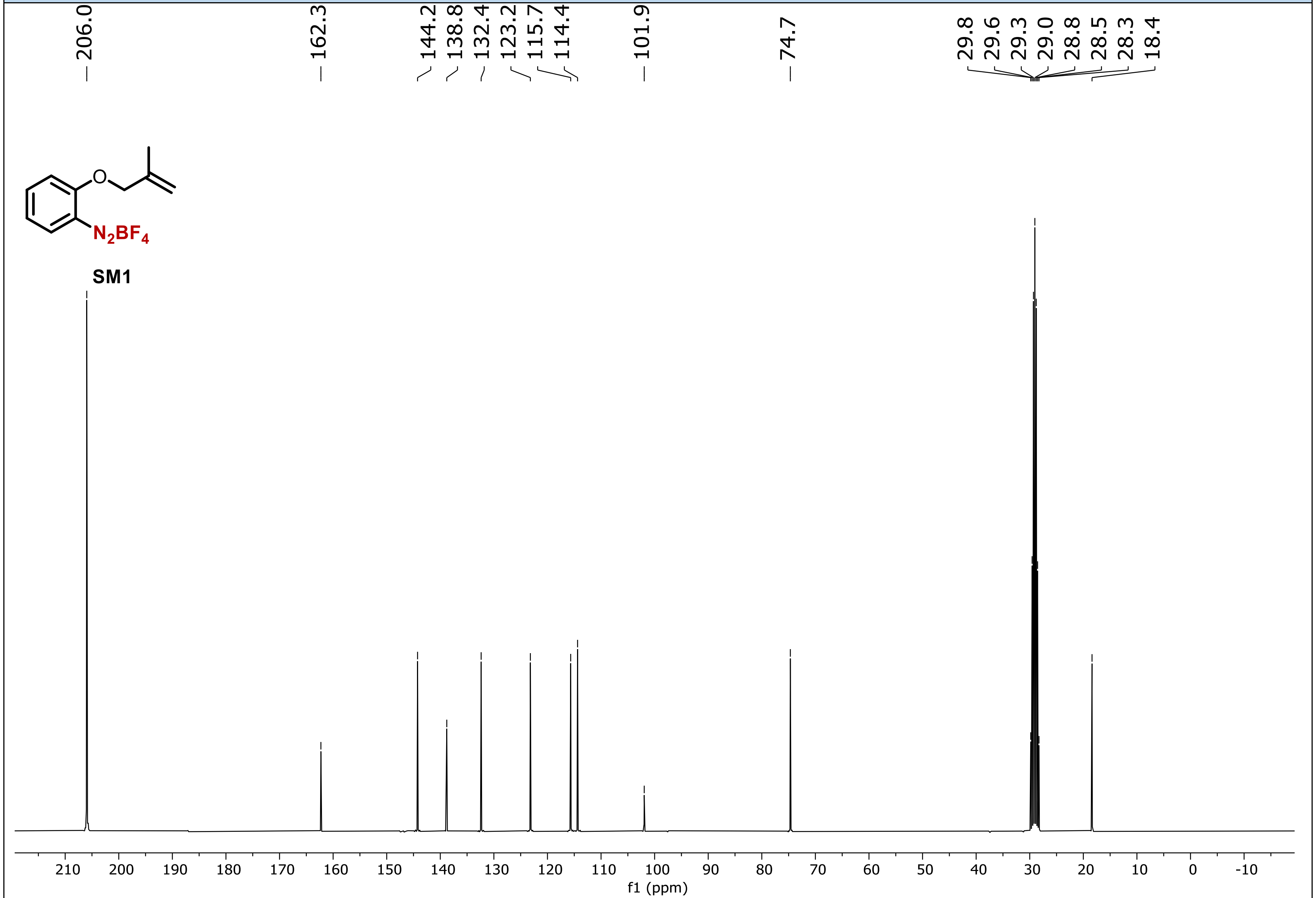

SM2 – ¹H NMR (500 MHz, Acetone-d$_6$)

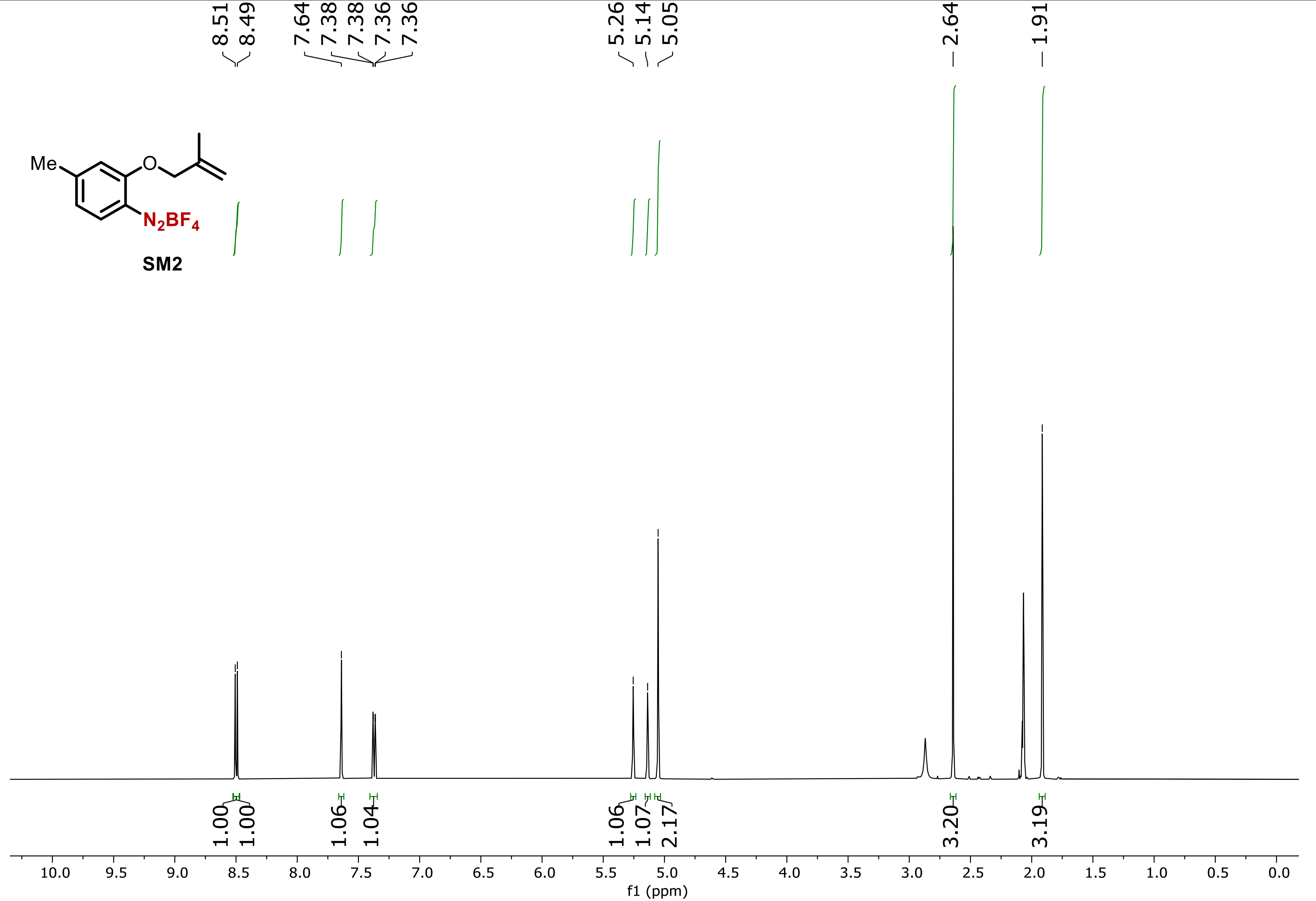

SM2 - $^{13}C\{^1H\}$ NMR (126 MHz, Acetone-d$_6$)

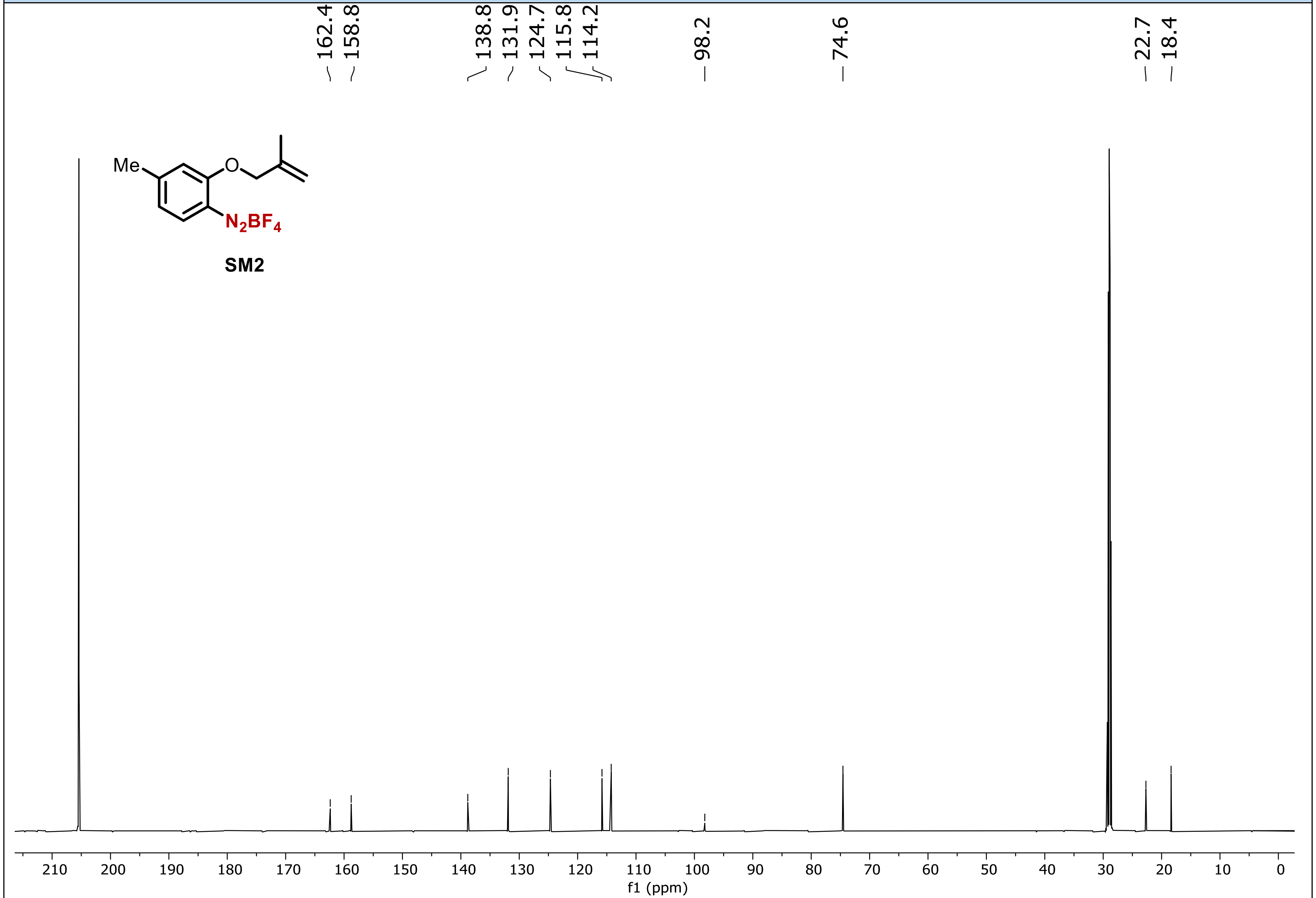

SM3 – ^{1}H NMR (500 MHz, Acetone-d$_6$)

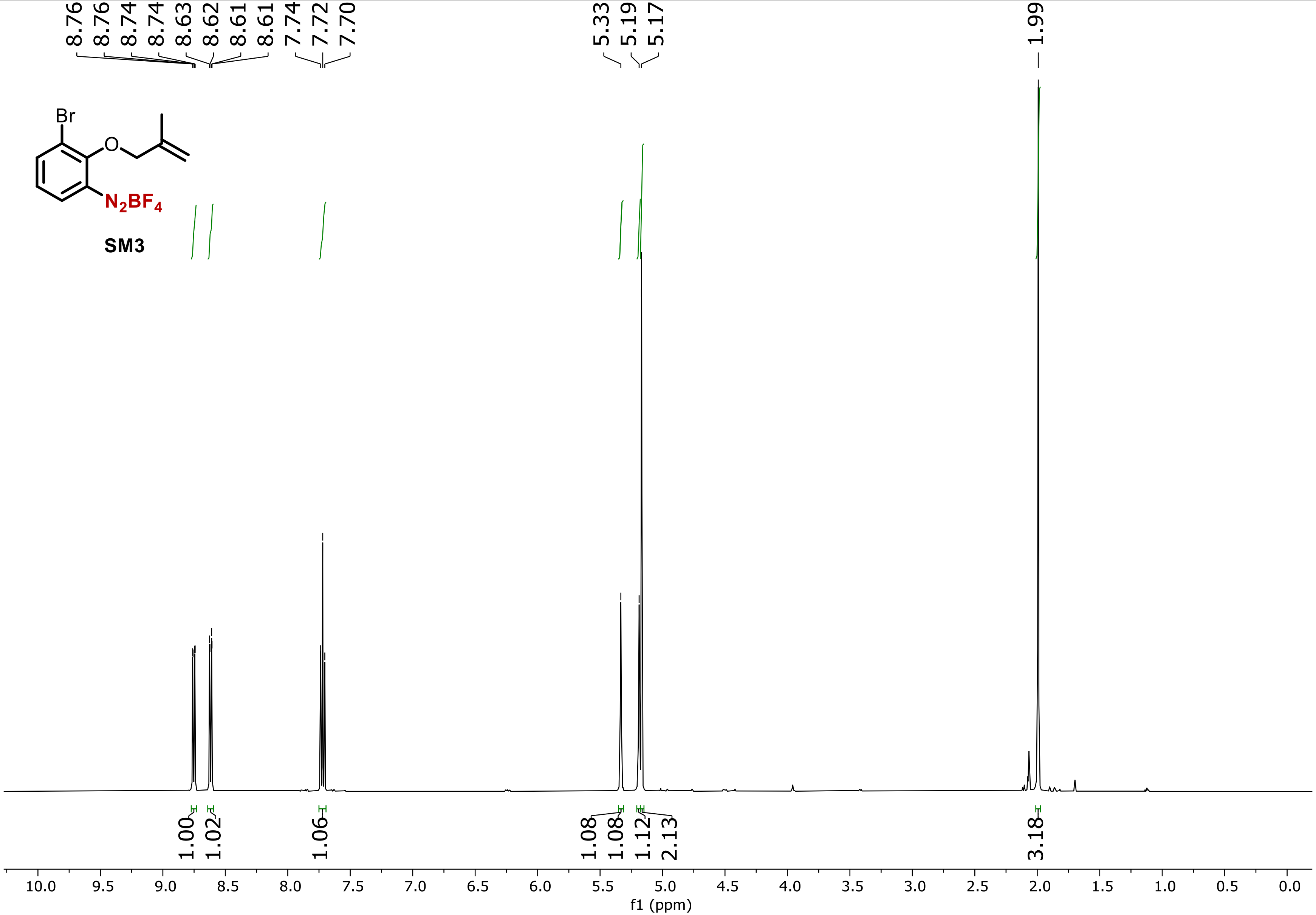

SM3 - $^{13}C\{^{1}H\}$ NMR (126 MHz, Acetone-d_6)

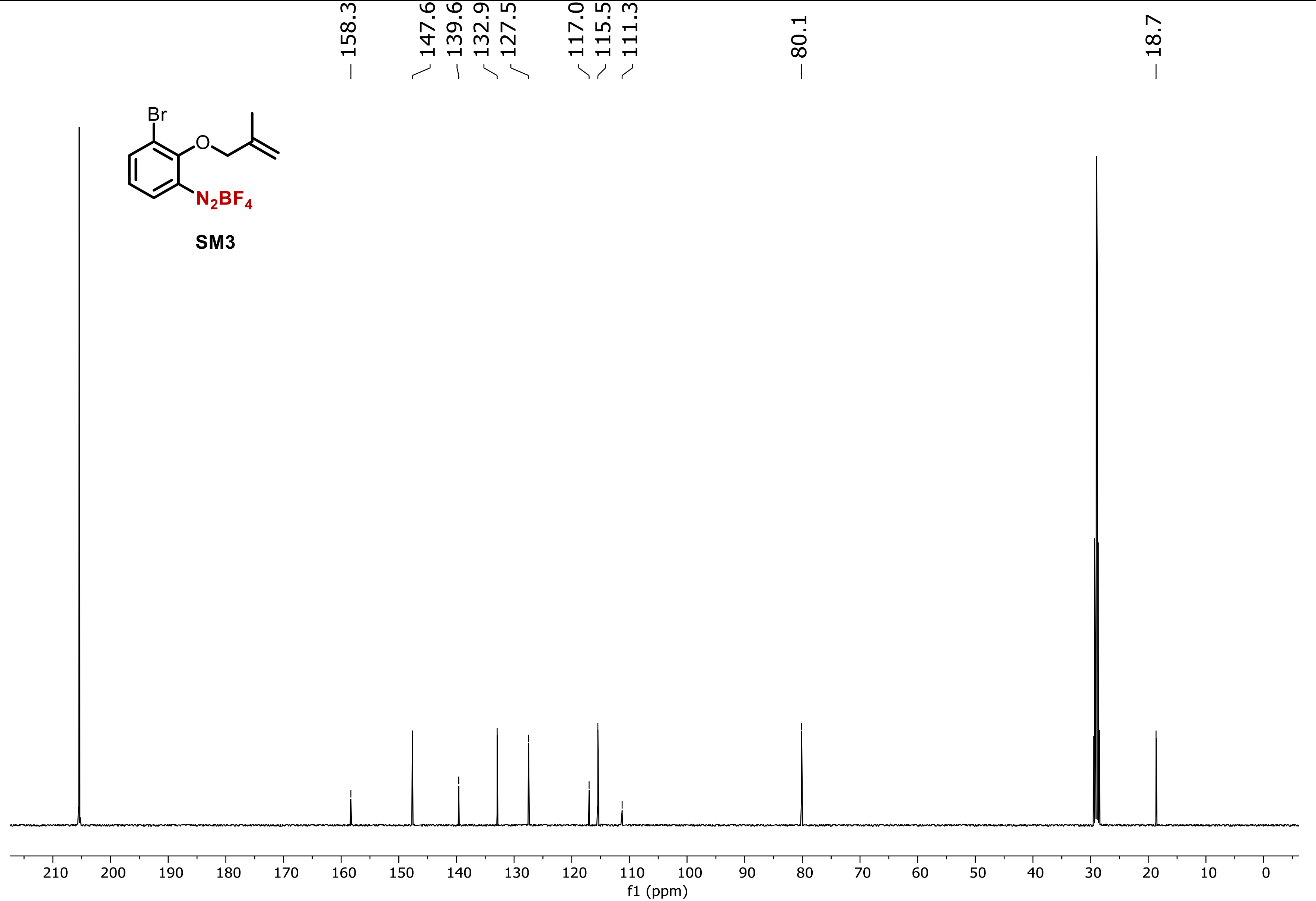

SM4 – ^{1}H NMR (500 MHz, Acetone-d6)

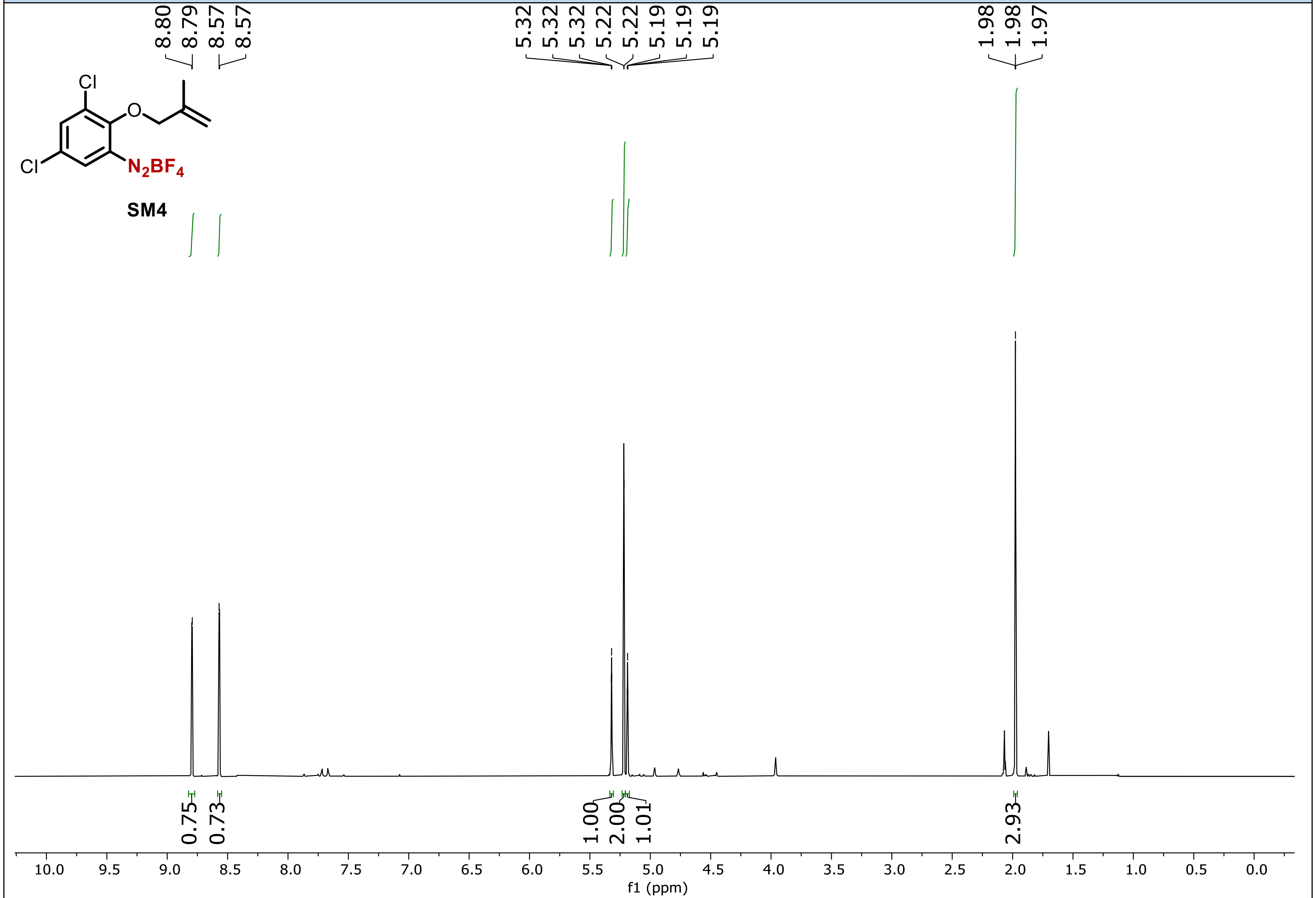

SM4 - $^{13}C\{^{1}H\}$ NMR (151 MHz, Acetone-d_6)

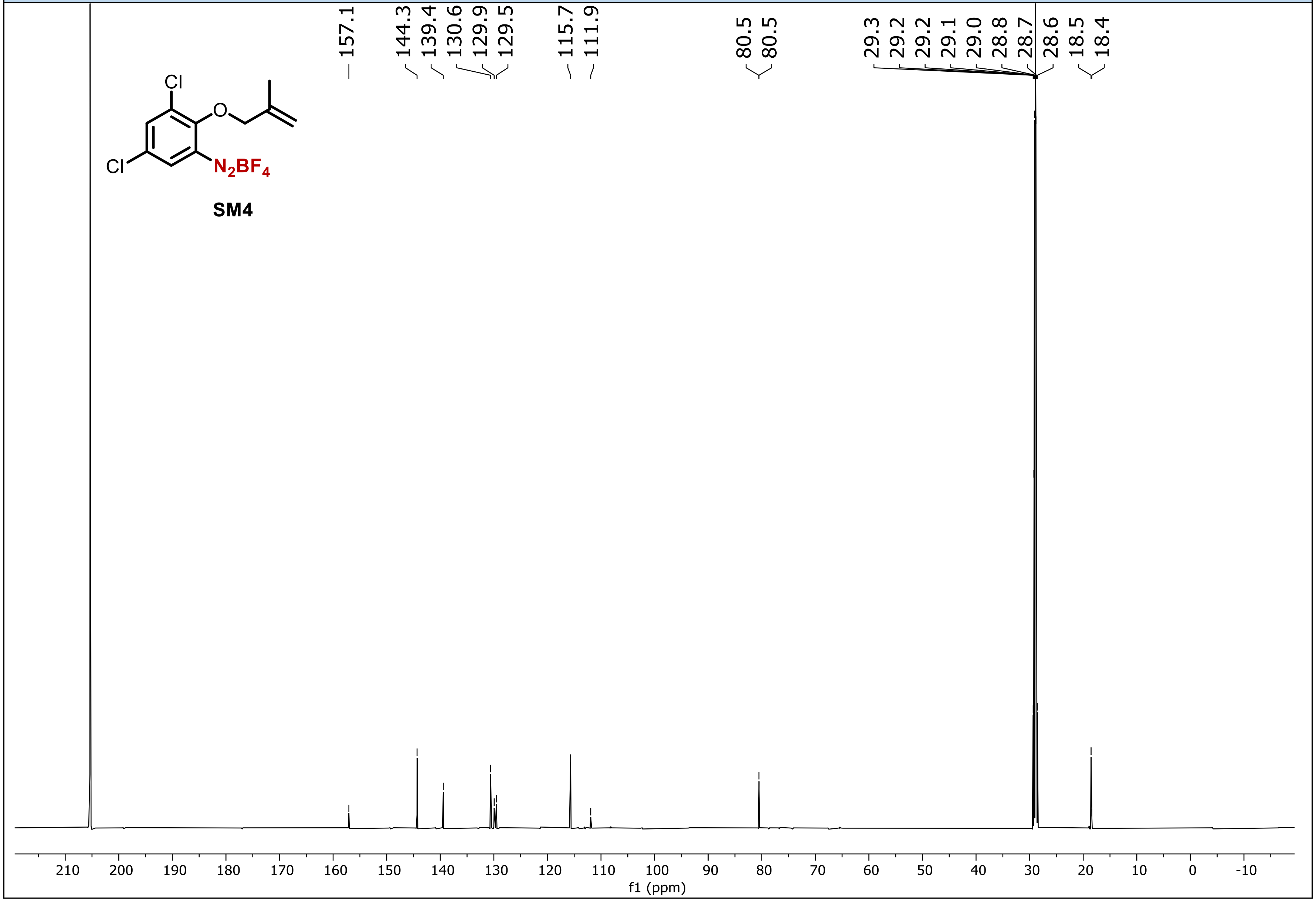

SM5 – ^{1}H NMR (600 MHz, Acetone-d_6)

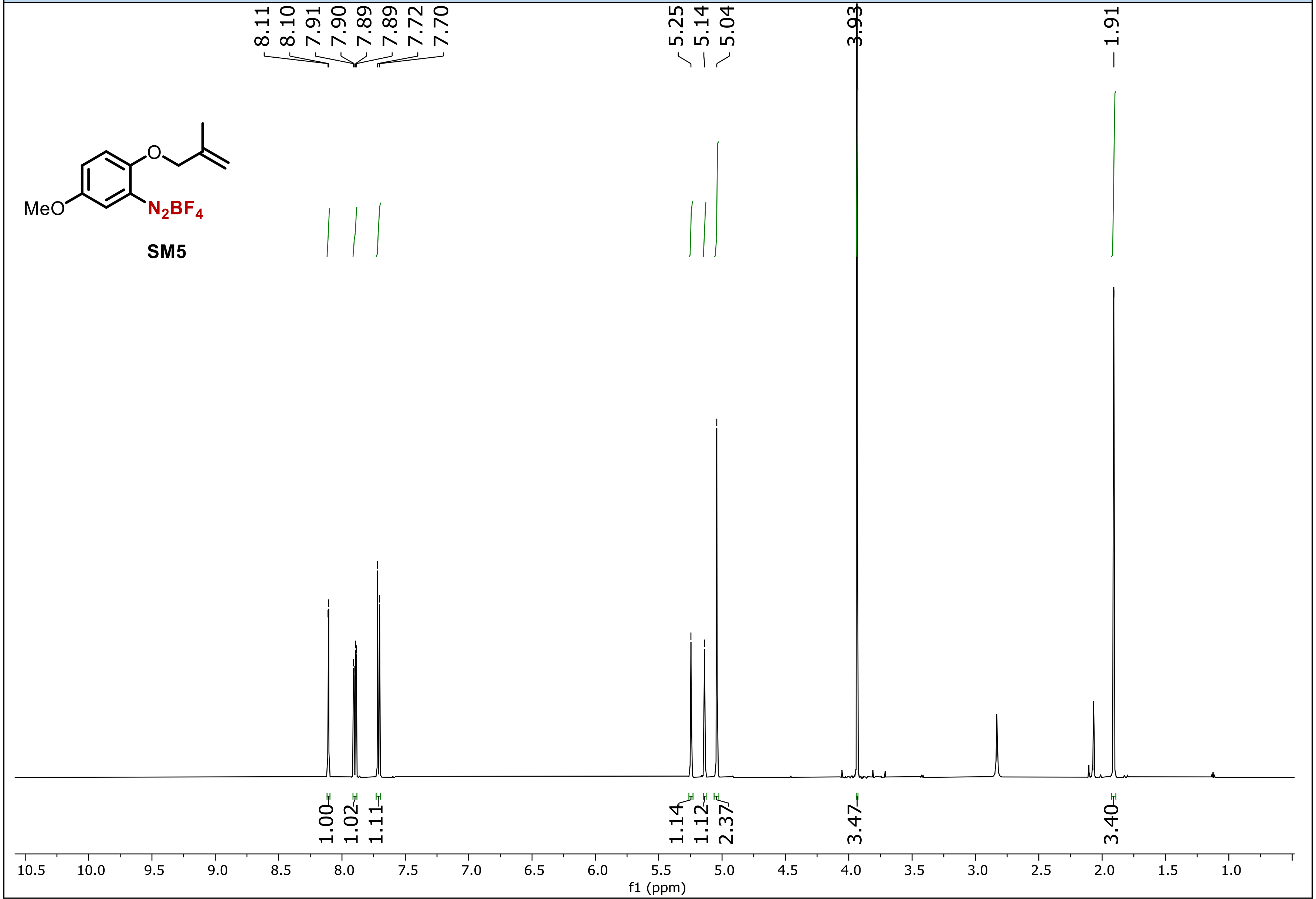

SM5 - $^{13}C\{^1H\}$ NMR (151 MHz, Acetone-d_6)

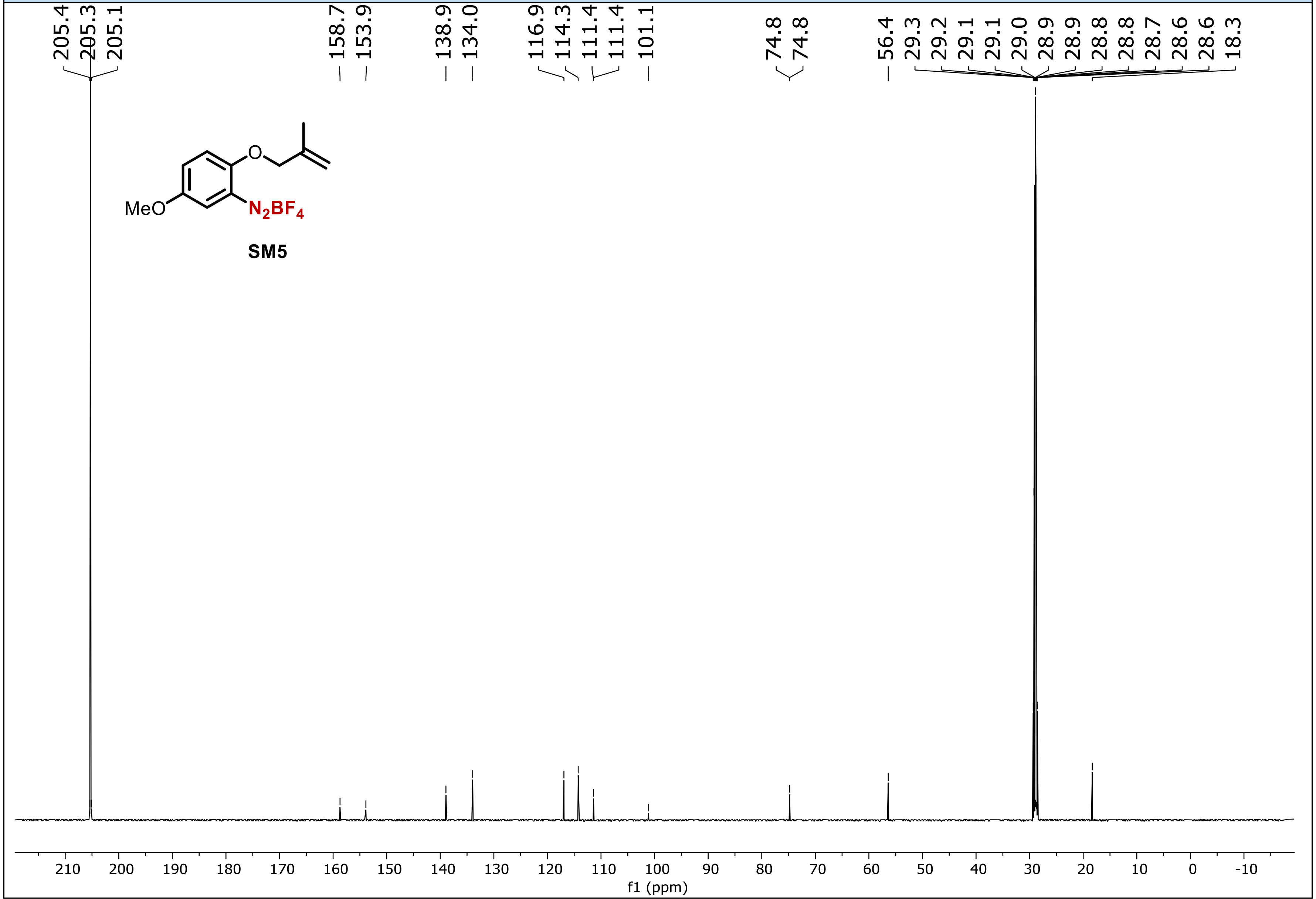

SM6 – ^{1}H NMR (500 MHz, Acetone-d_6)

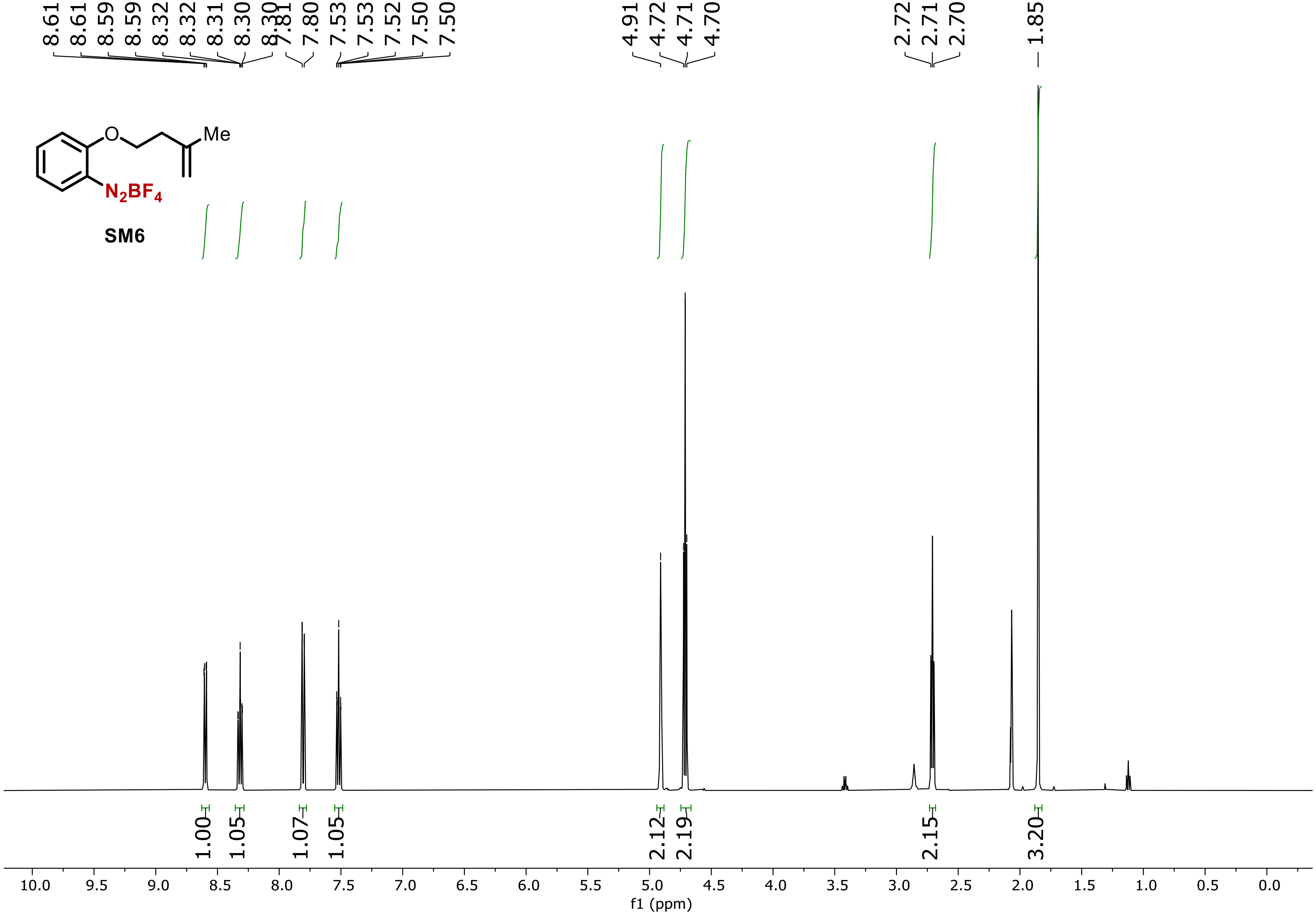

SM6 - $^{13}C\{^1H\}$ NMR (126 MHz, Acetone-d_6)

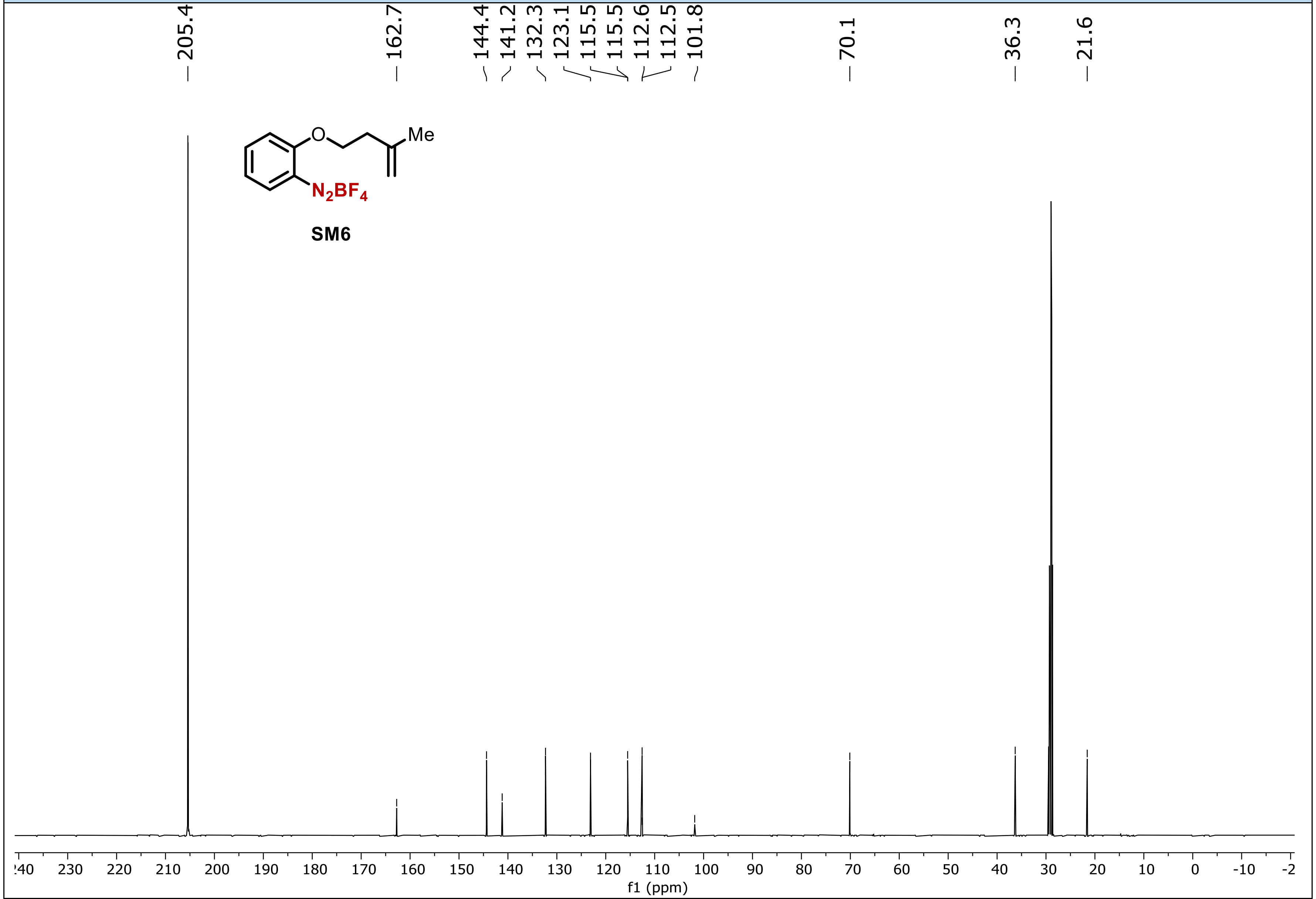

SM7 – ^{1}H NMR (500 MHz, Acetone-d_6)

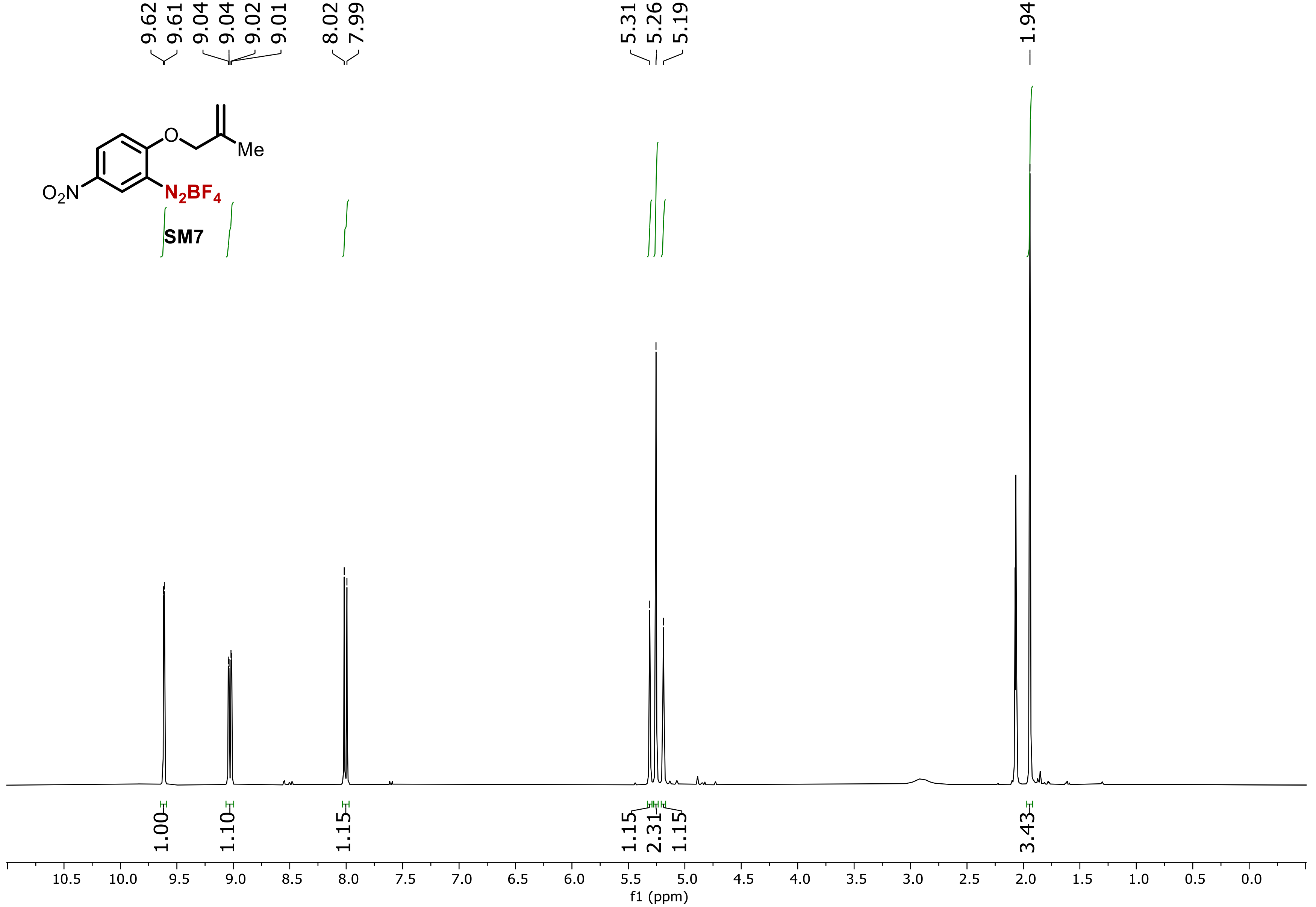

SM7 - $^{13}C\{^1H\}$ NMR (126 MHz, Acetone-d_6)

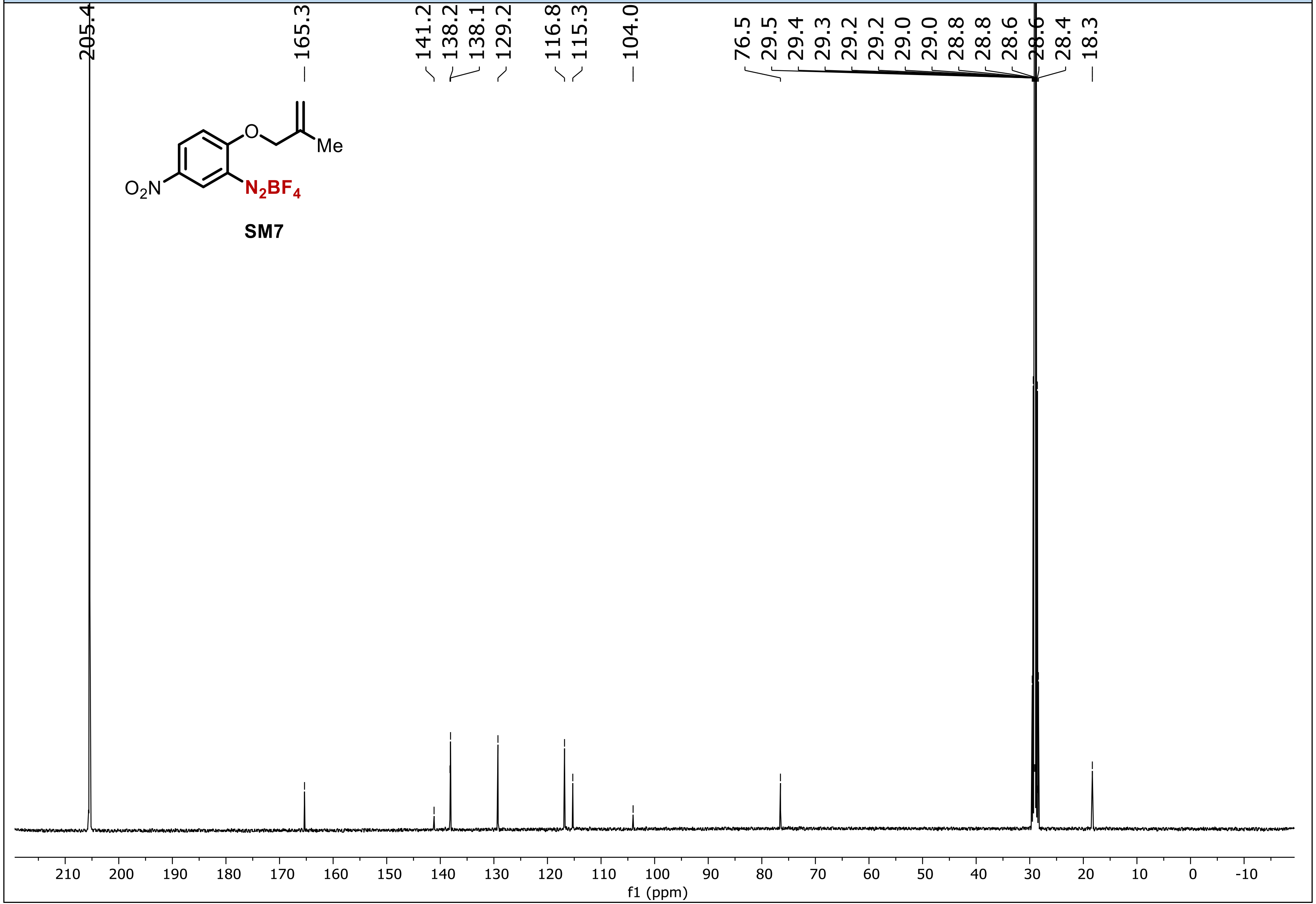

SM8 – ^{1}H NMR (400 MHz, Acetone-d_6)

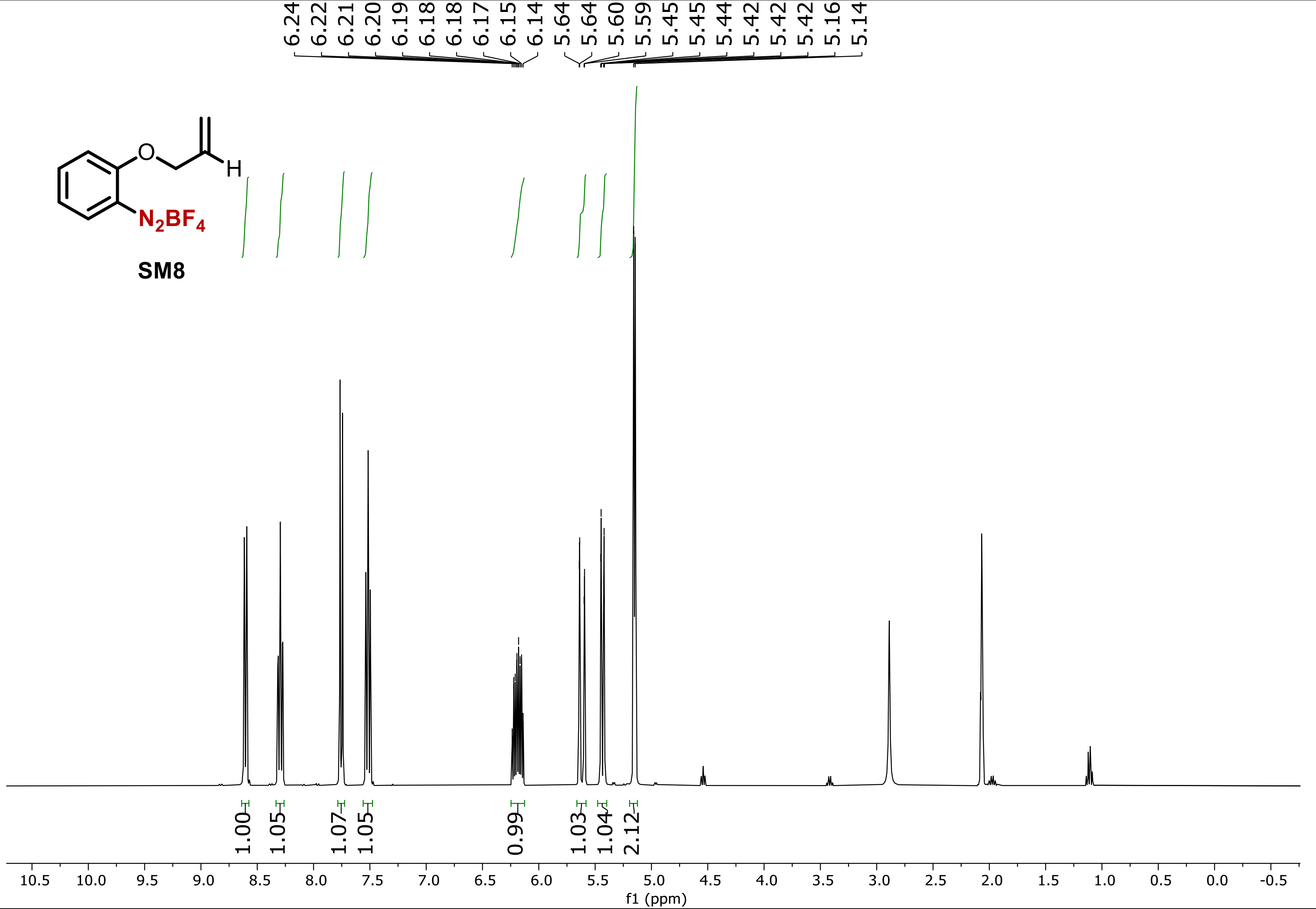

SM8 - $^{13}C\{^{1}H\}$ NMR (101 MHz, Acetone-d_6)

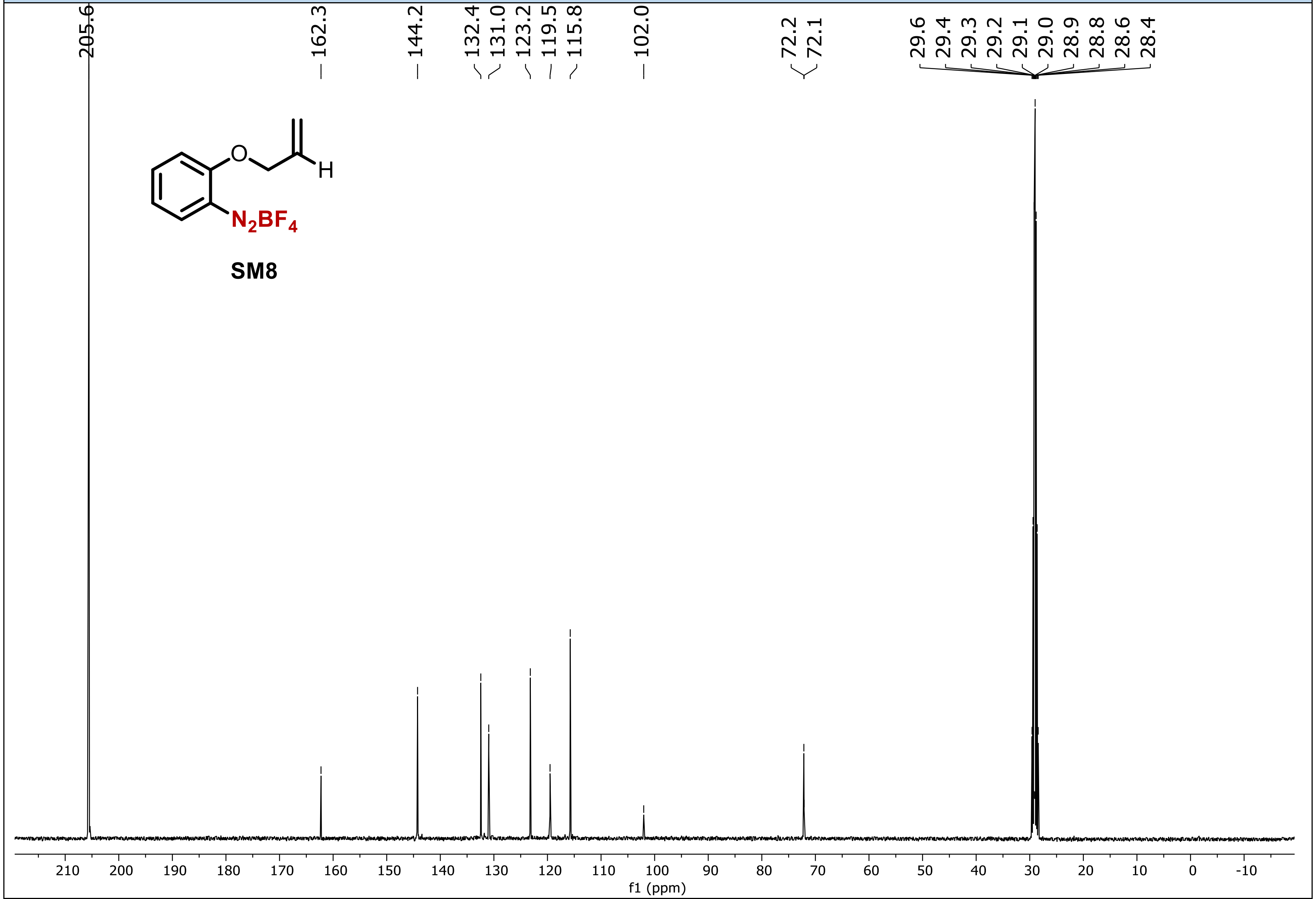

SM9 – ^{1}H NMR (250 MHz, Acetone-d$_6$)

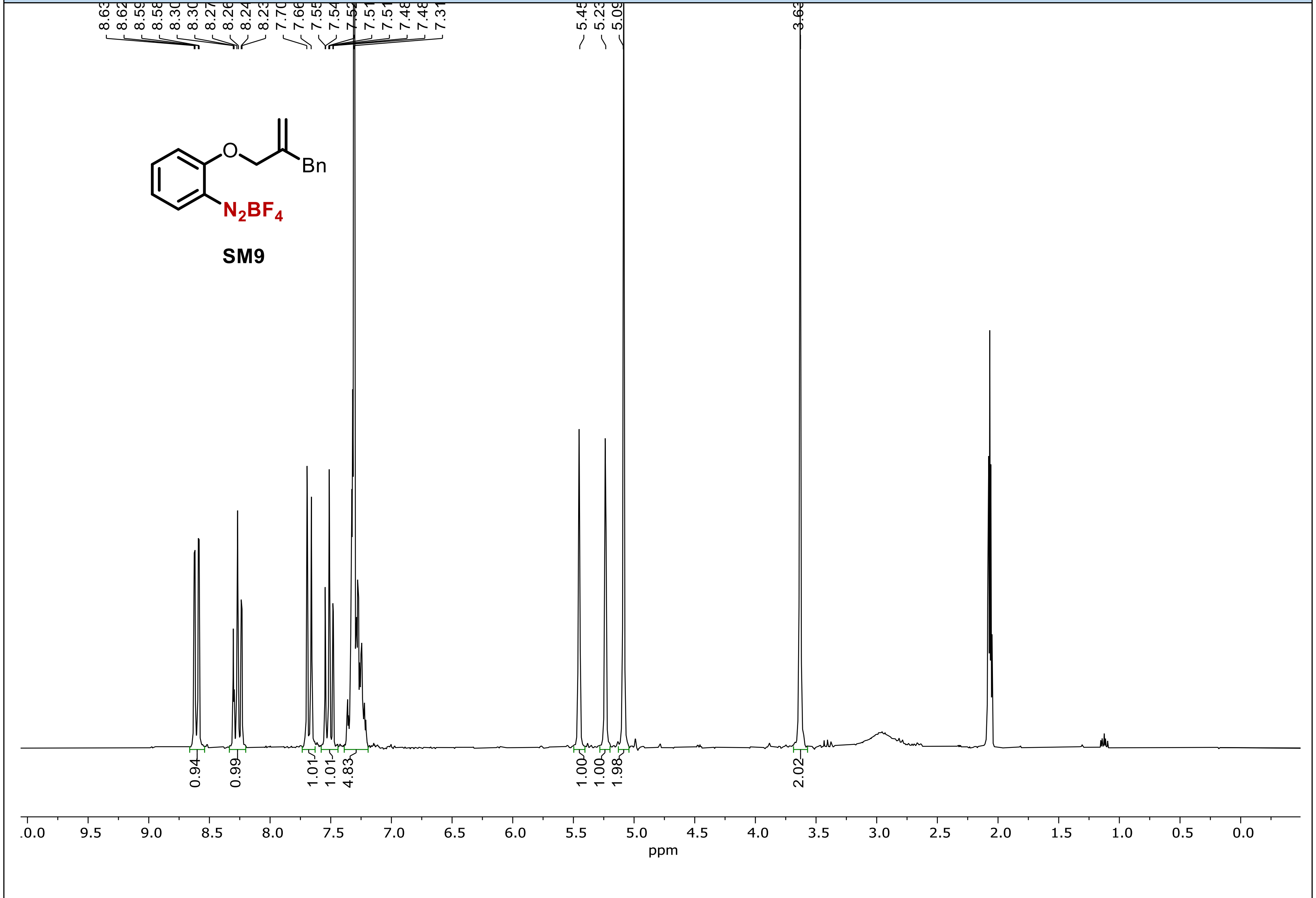

SM9 - $^{13}C\{^{1}H\}$ NMR (63 MHz, Acetone-d_6)

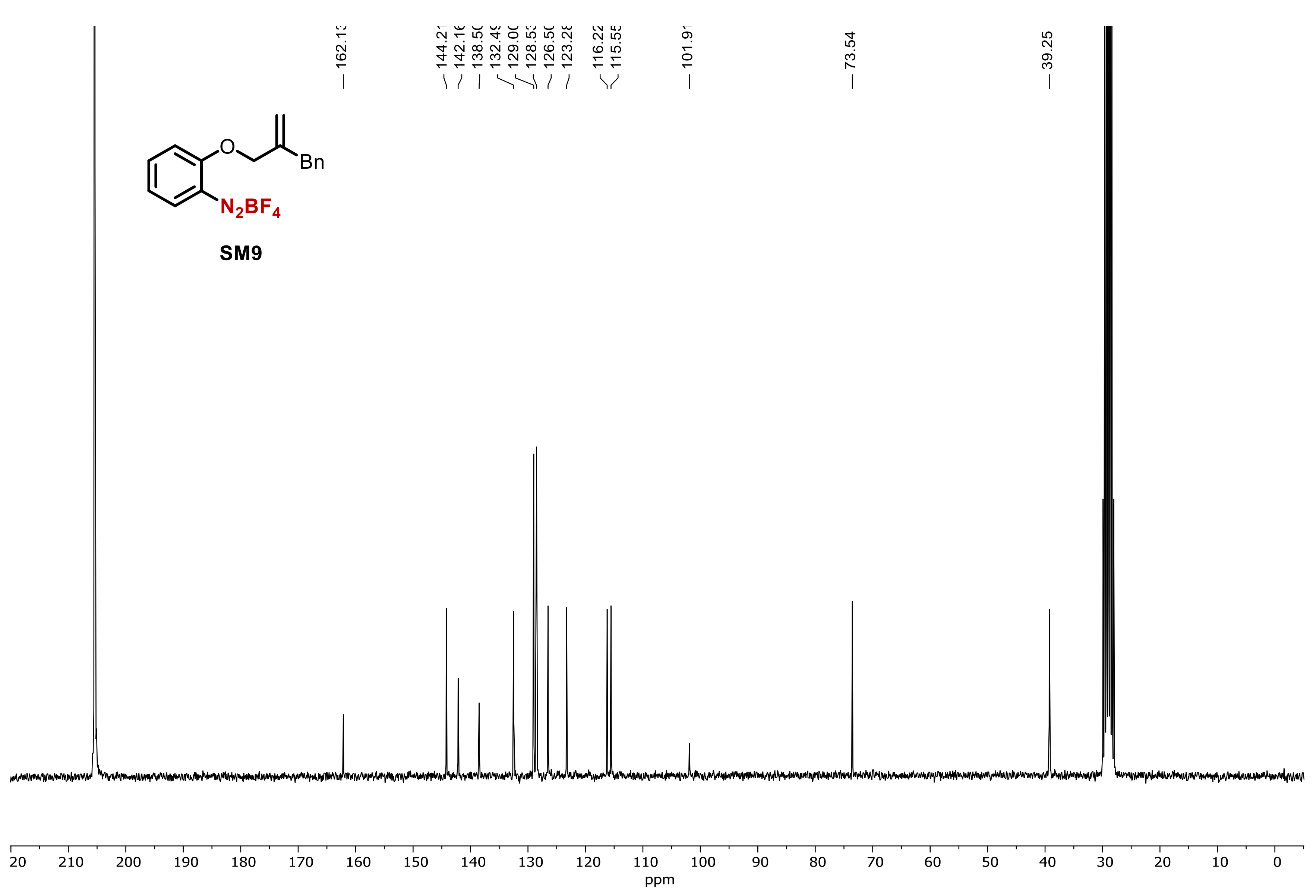

6. [1]H and [13]C{[1]H} for the Heck-Matsuda-Stille products

1a – ^{1}H NMR (500 MHz, $CDCl_3$)

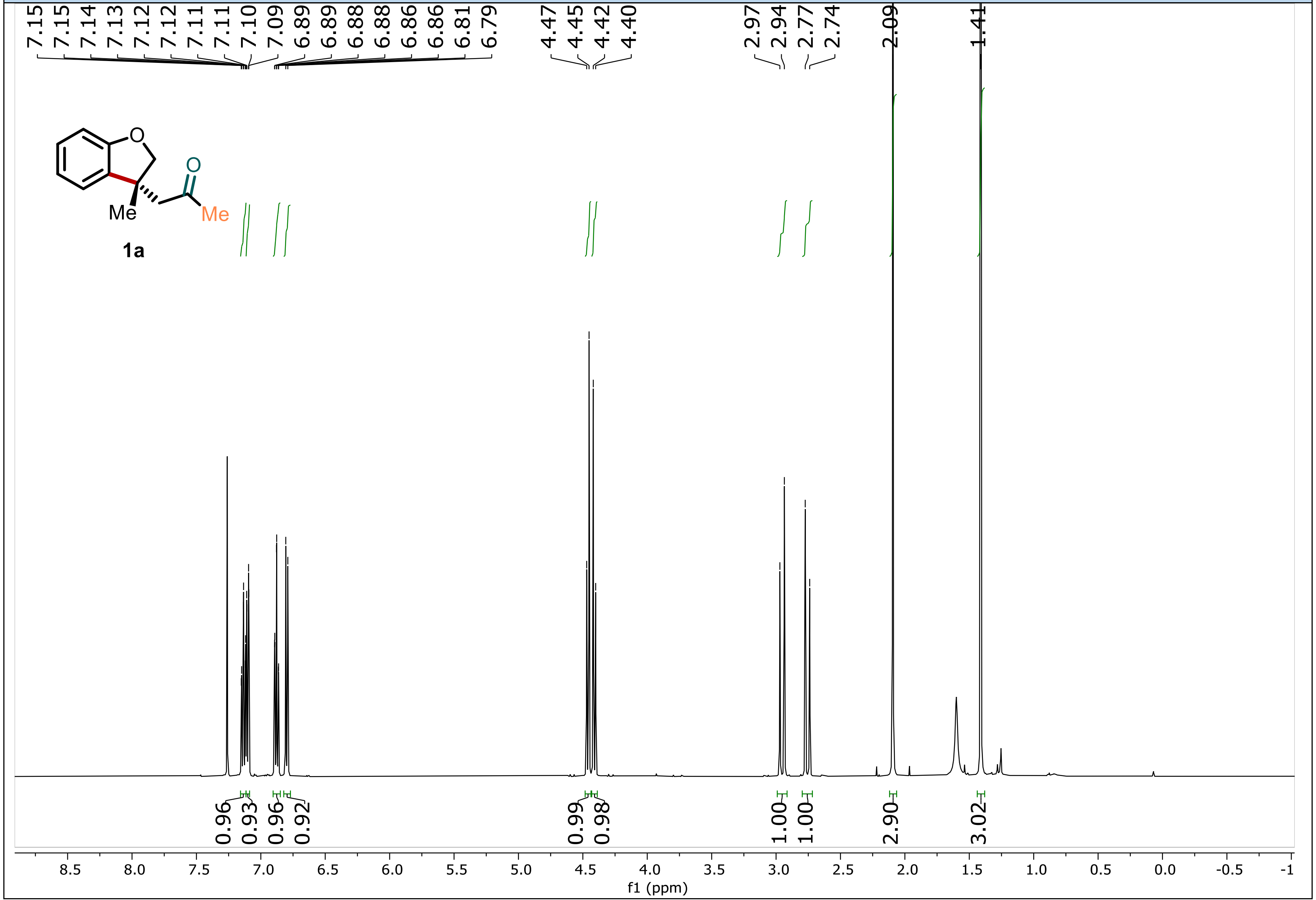

1a – $^{13}C\{^1H\}$ NMR (126 MHz, $CDCl_3$)

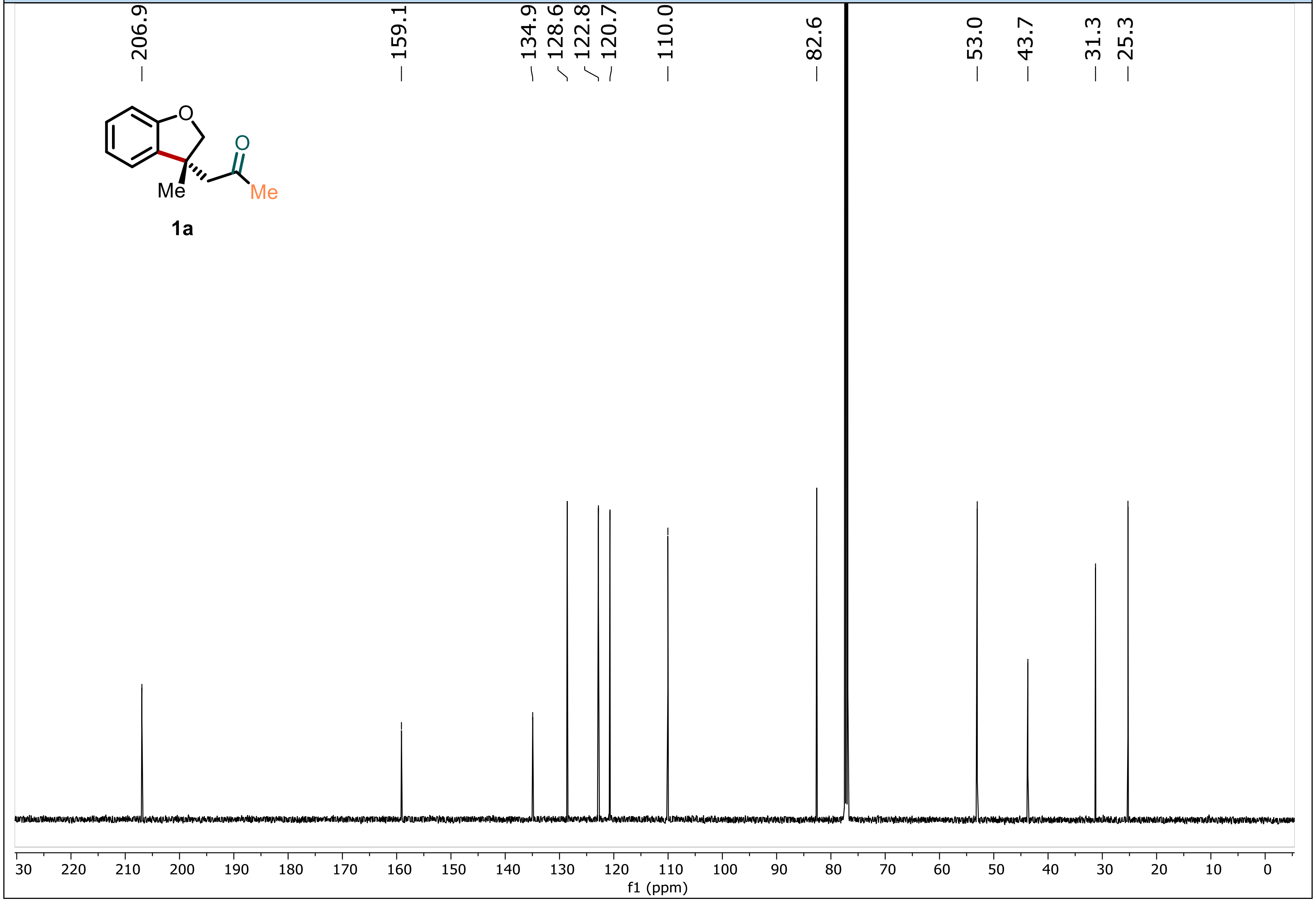

1b – ^{1}H NMR (250 MHz, $CDCl_3$)

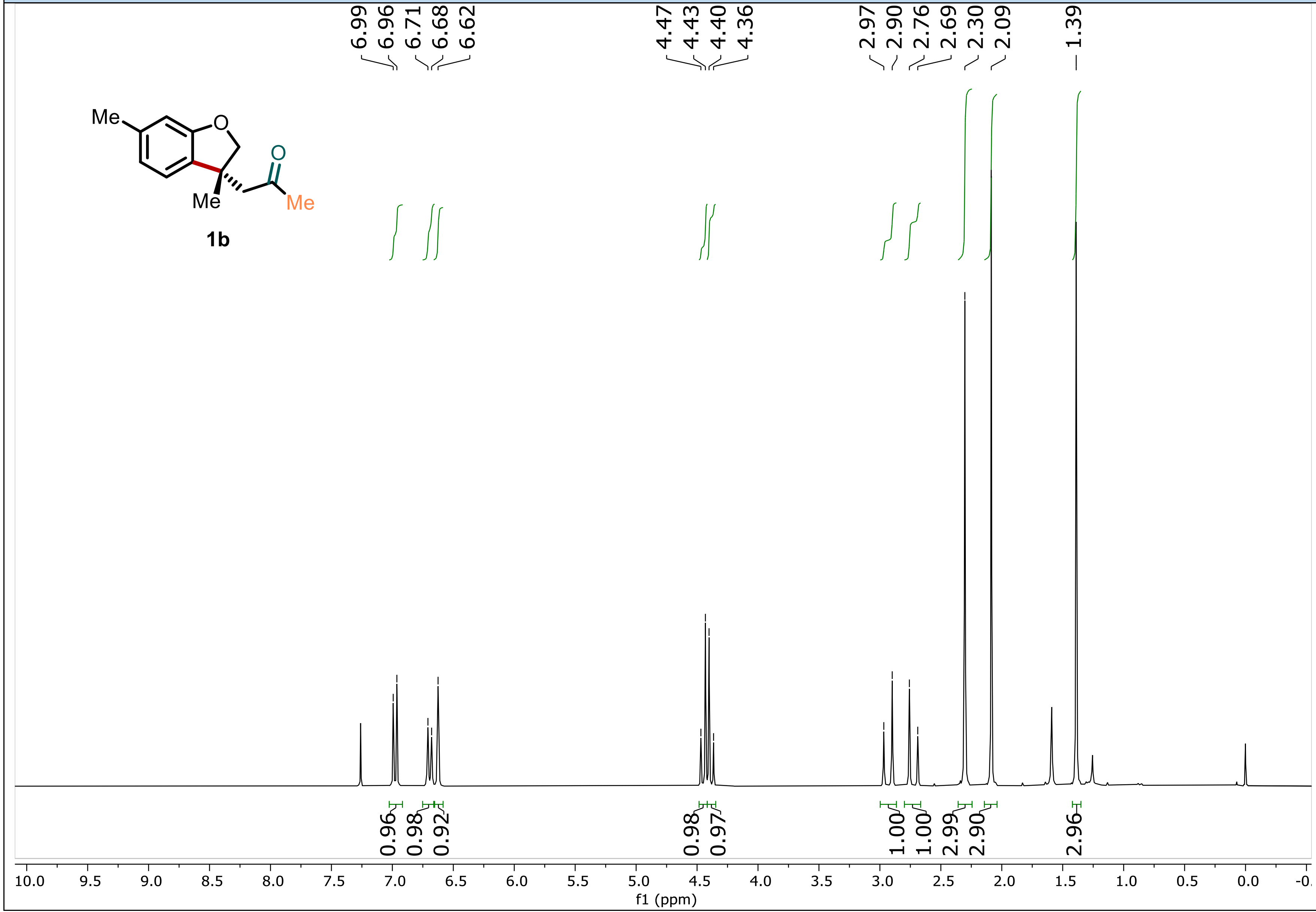

1b – $^{13}C\{^1H\}$ NMR (75 MHz, $CDCl_3$)

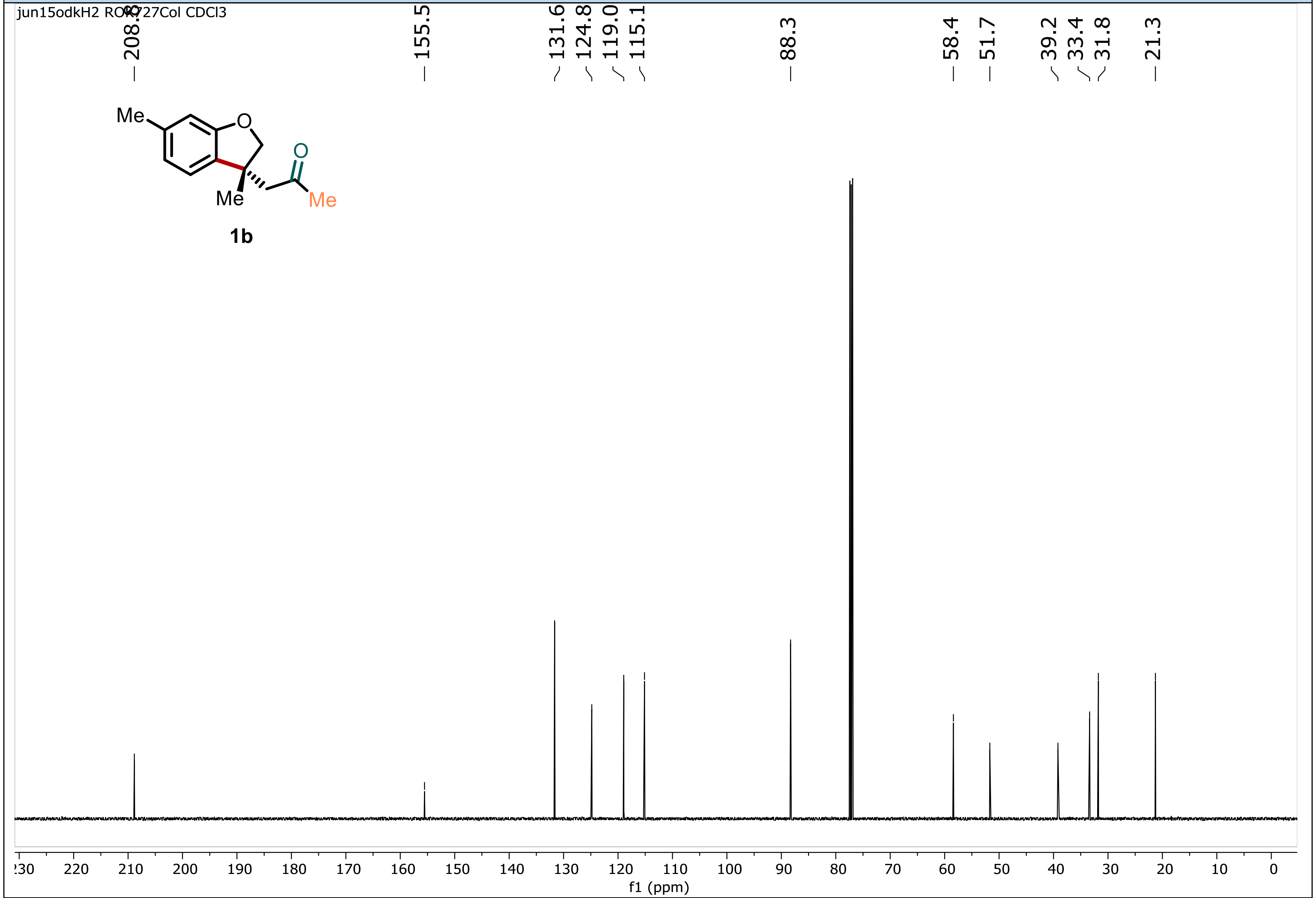

1c – ^{1}H NMR (500 MHz, $CDCl_3$)

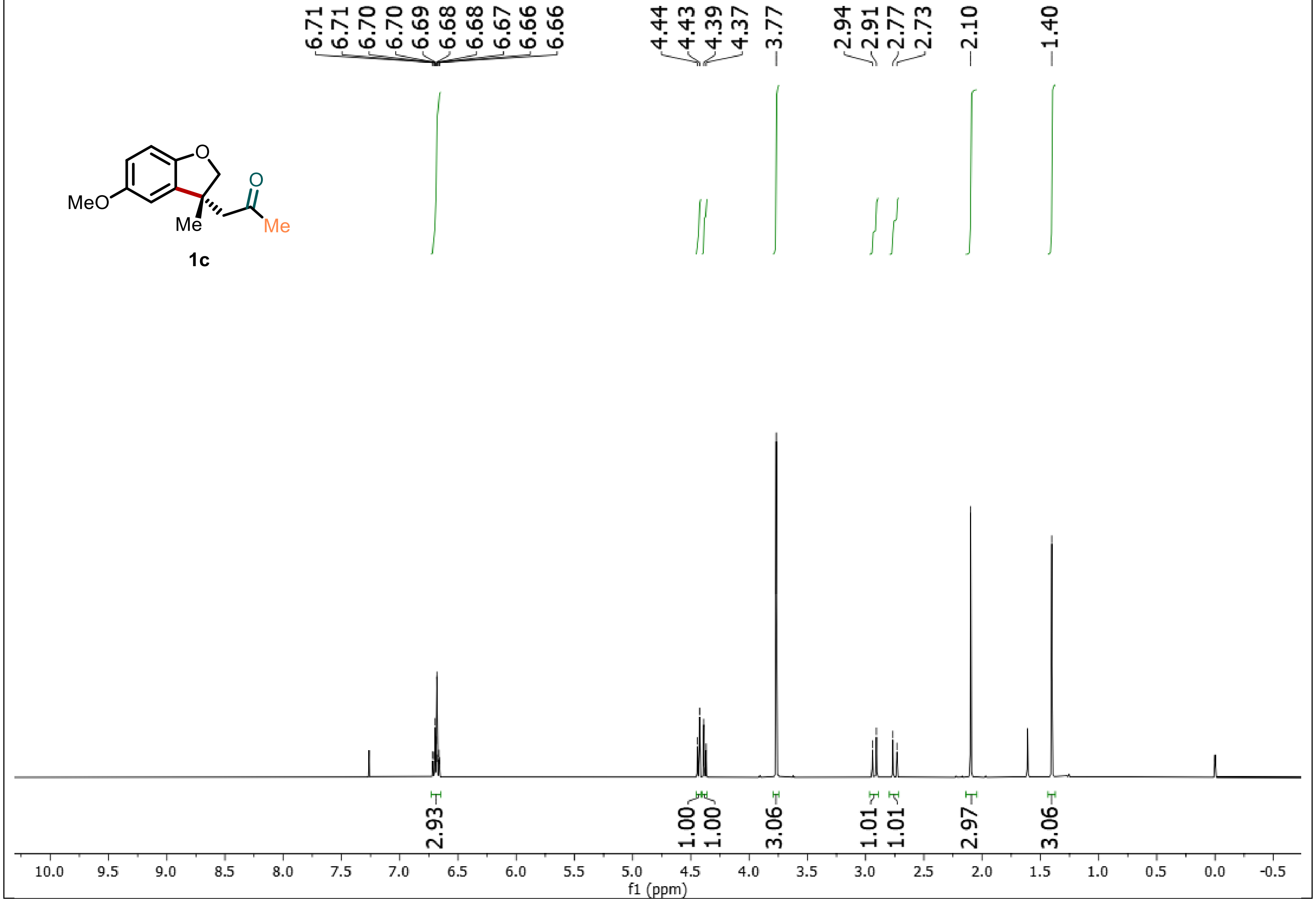

1c – $^{13}C\{^1H\}$ NMR (126 MHz, $CDCl_3$)

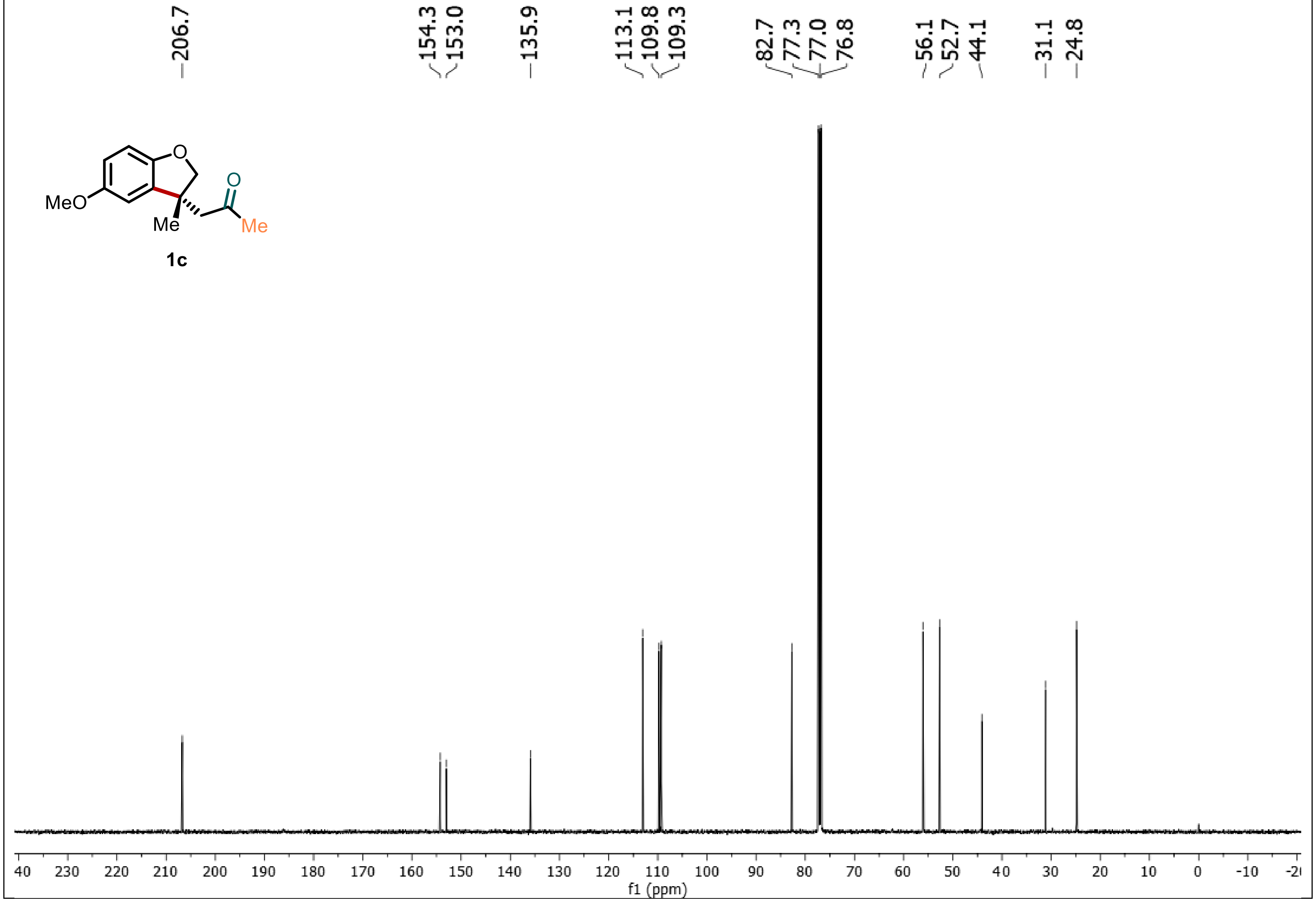

1d – ^{1}H NMR (600 MHz, $CDCl_3$)

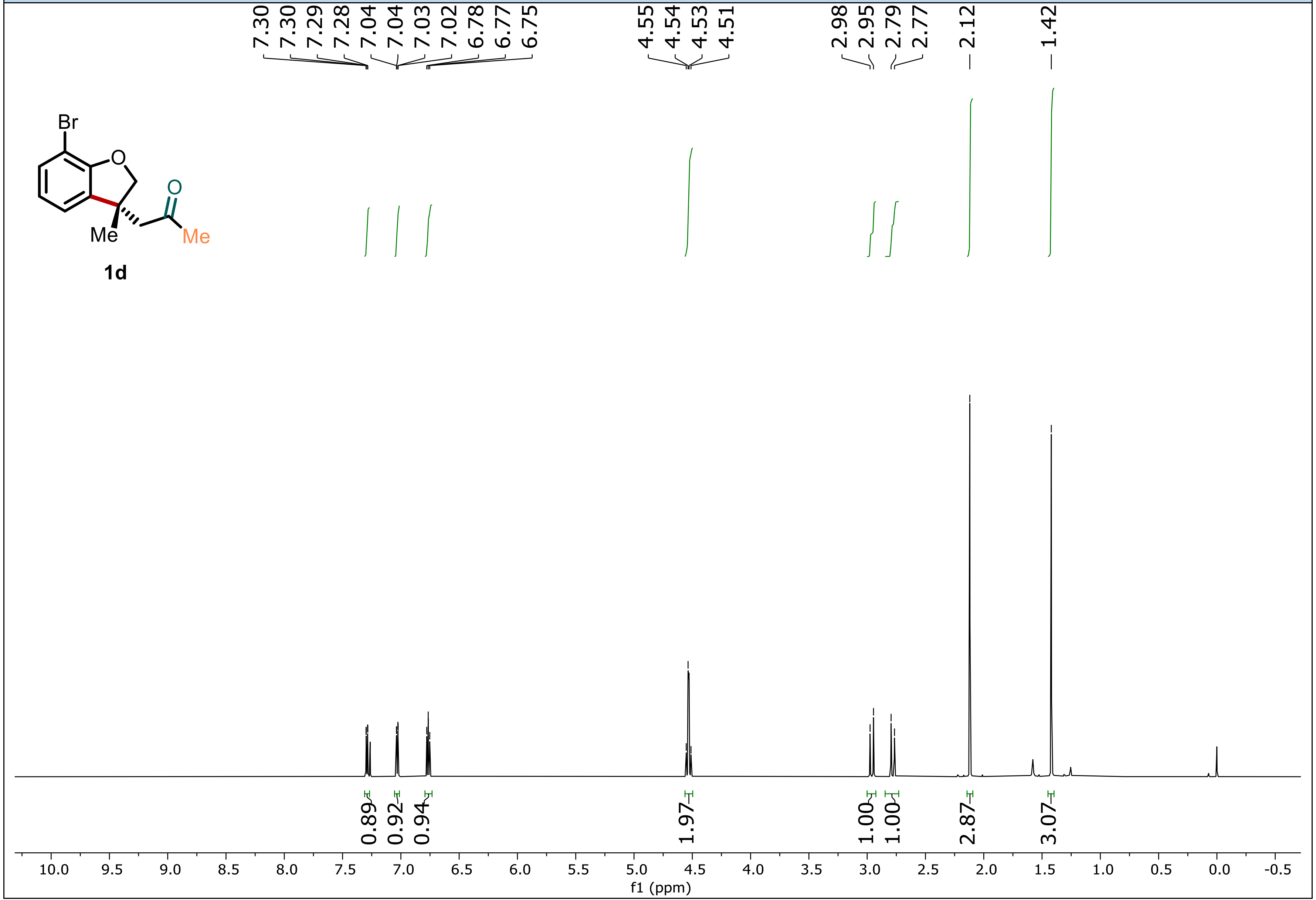

1d – $^{13}C\{^{1}H\}$ NMR (151 MHz, $CDCl_3$)

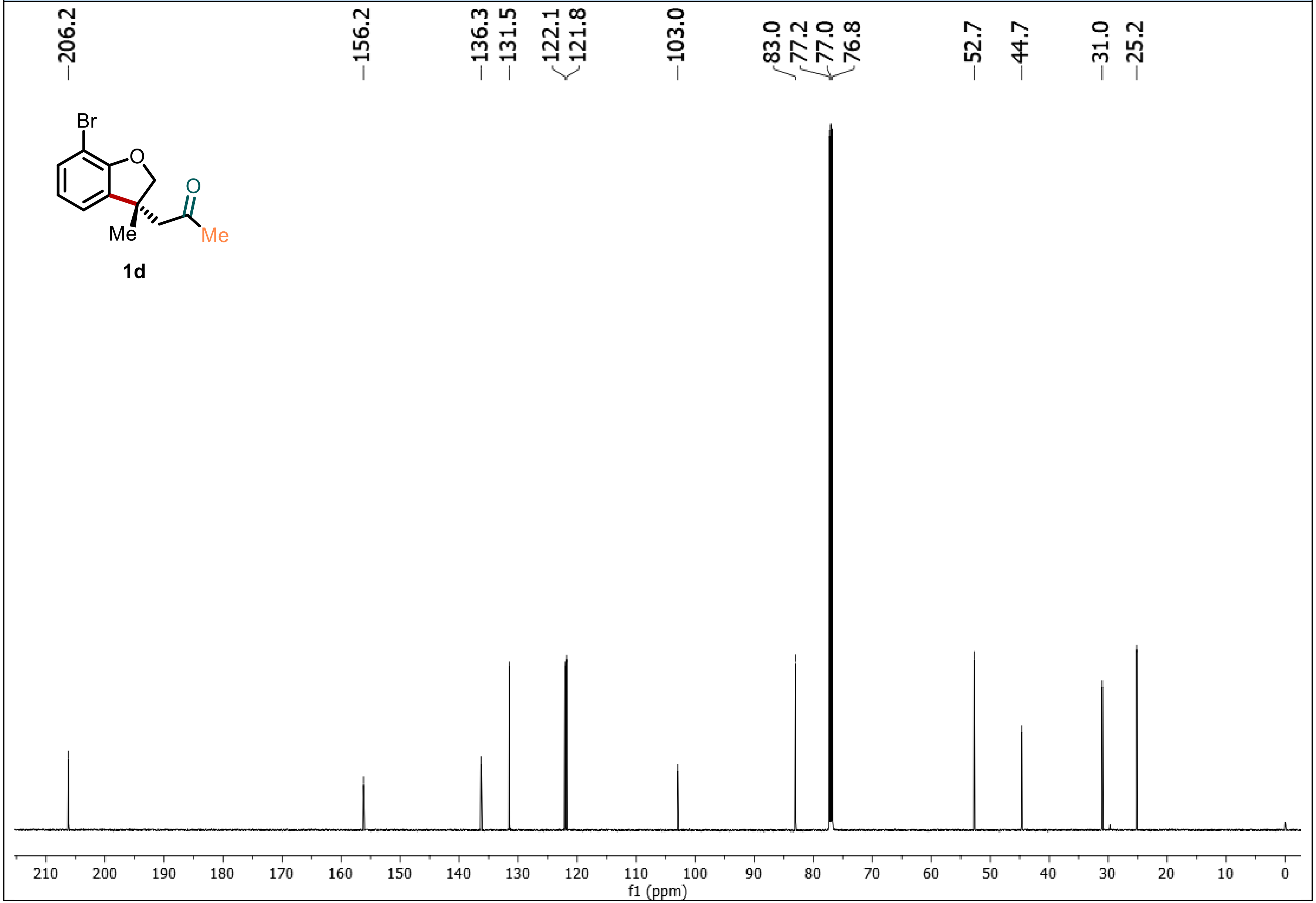

1e – ^{1}H NMR (500 MHz, $CDCl_3$)

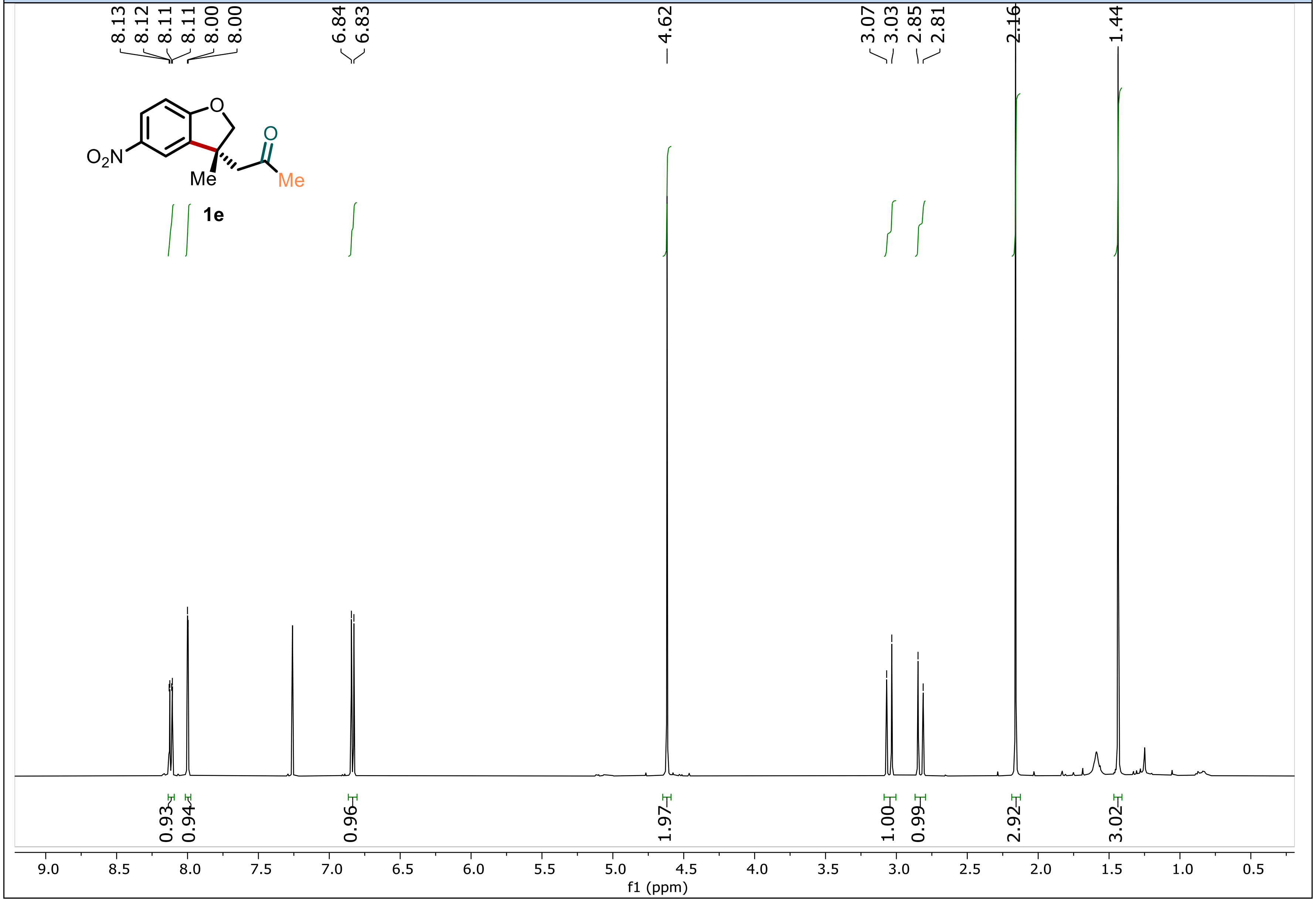

1e – $^{13}C\{^{1}H\}$ NMR (126 MHz, $CDCl_3$)

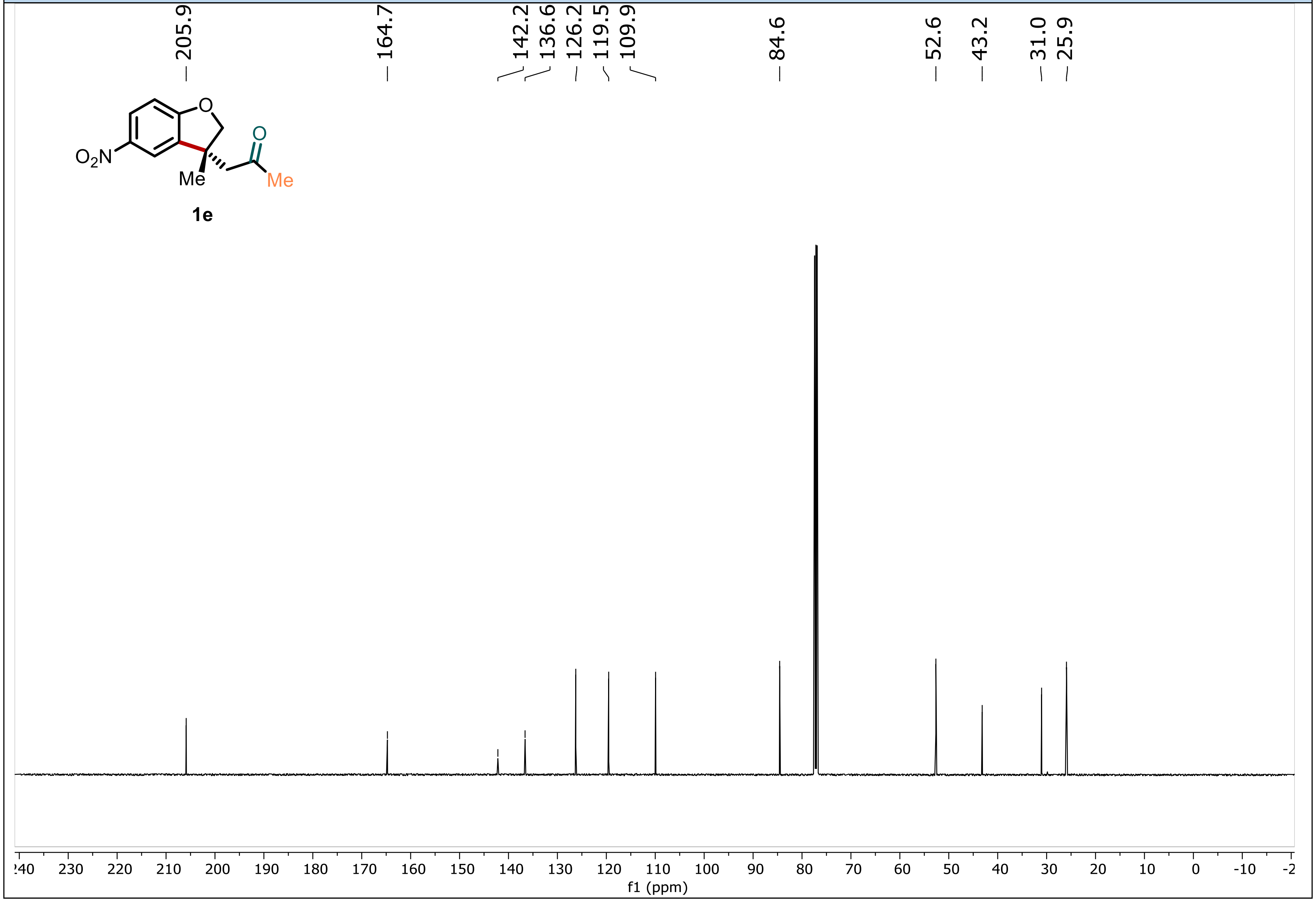

1f – ^{1}H NMR (600 MHz, $CDCl_3$)

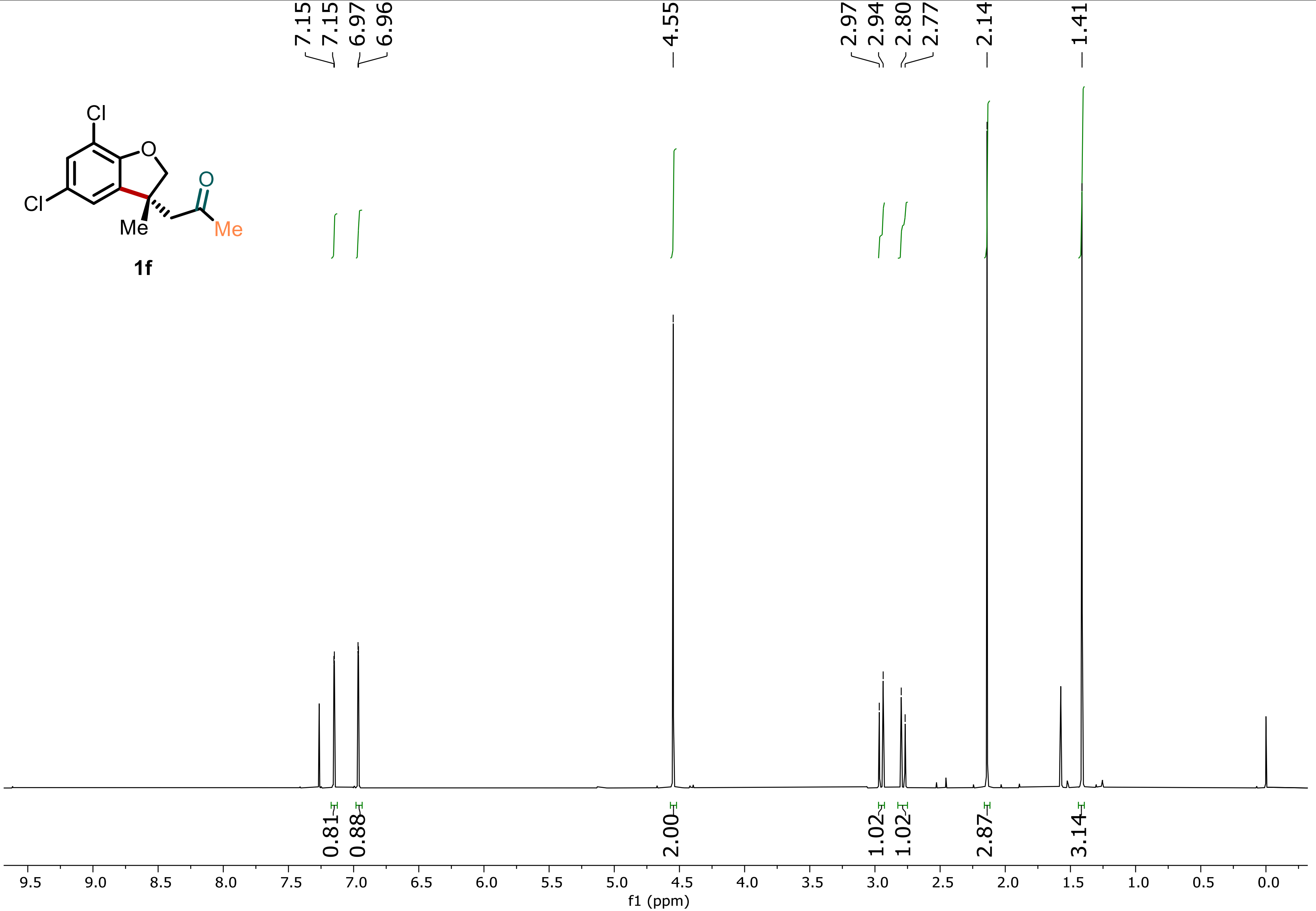

1f – $^{13}C\{^1H\}$ NMR (151 MHz, $CDCl_3$)

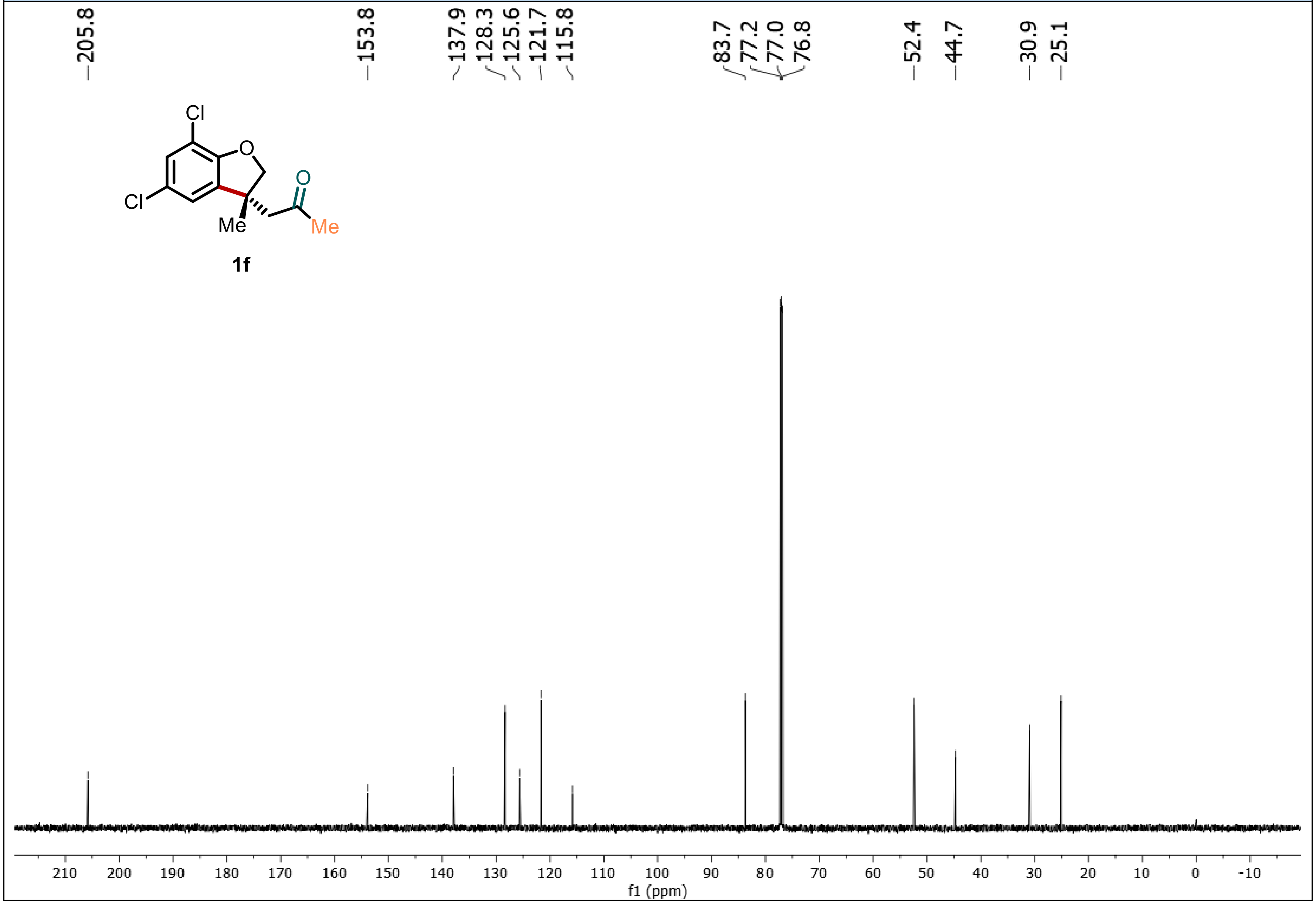

1g – ^{1}H NMR (500 MHz, $CDCl_3$)

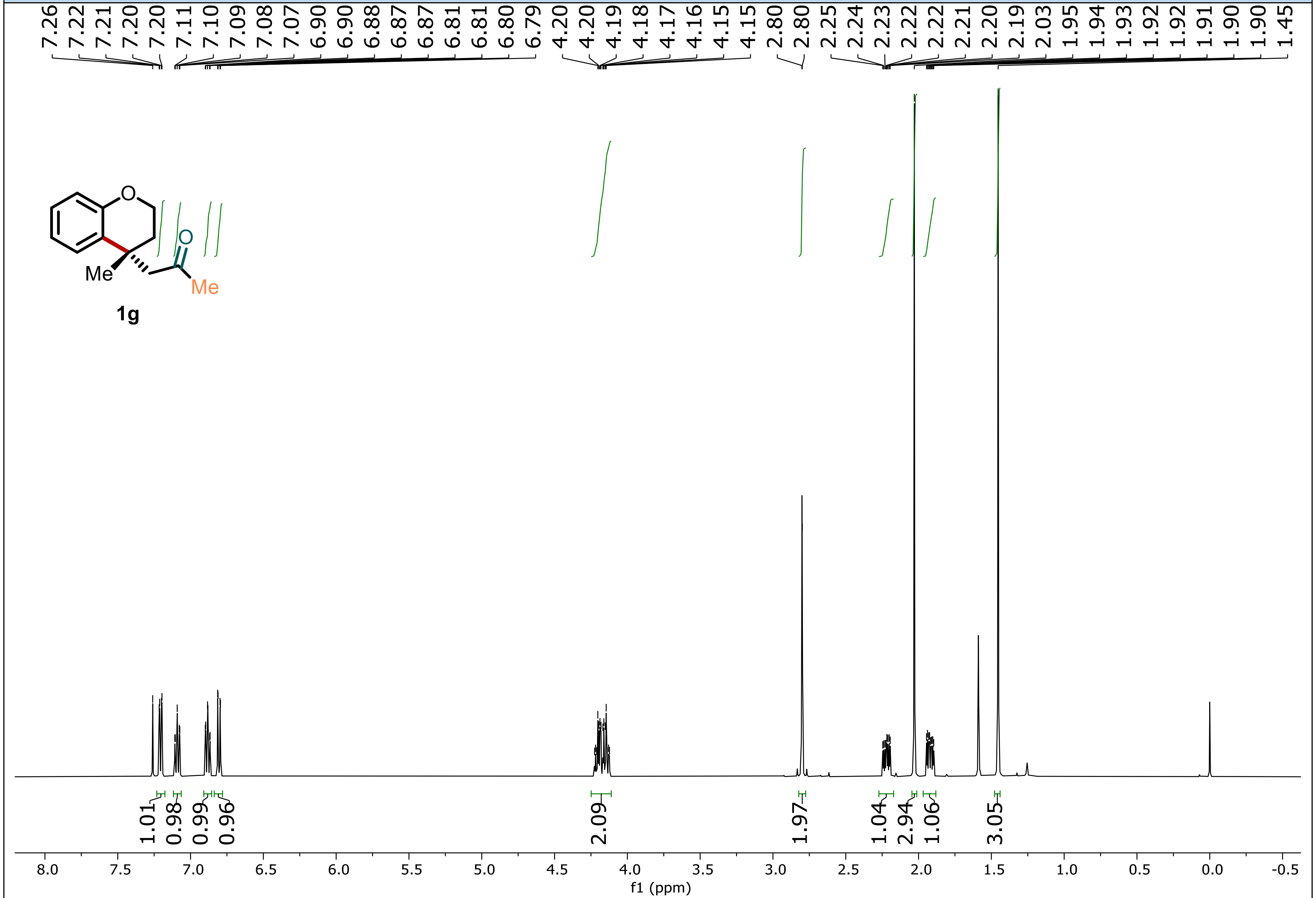

1g – $^{13}C\{^1H\}$ NMR (126 MHz, $CDCl_3$)

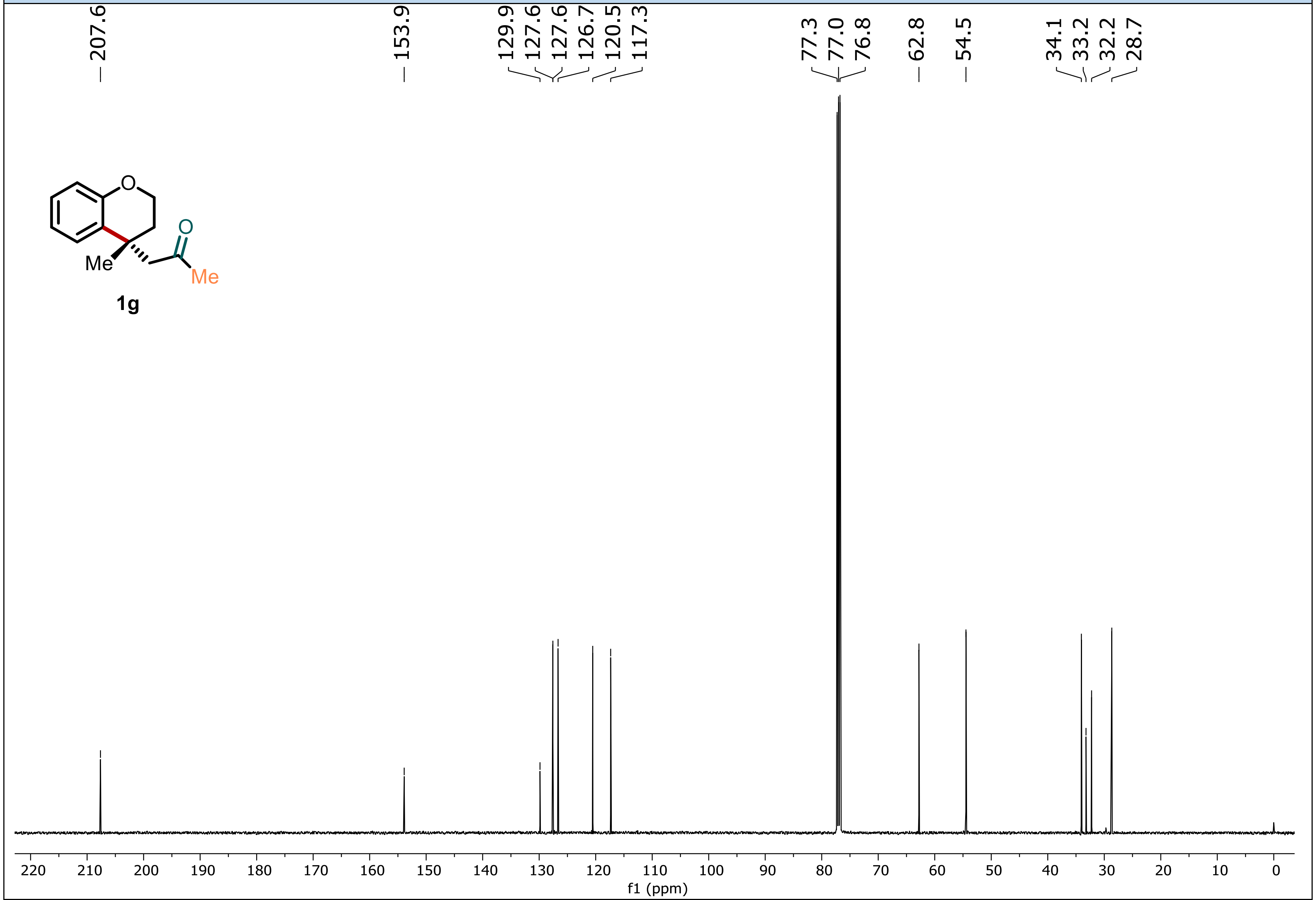

1h – ^{1}H NMR (600 MHz, $CDCl_3$)

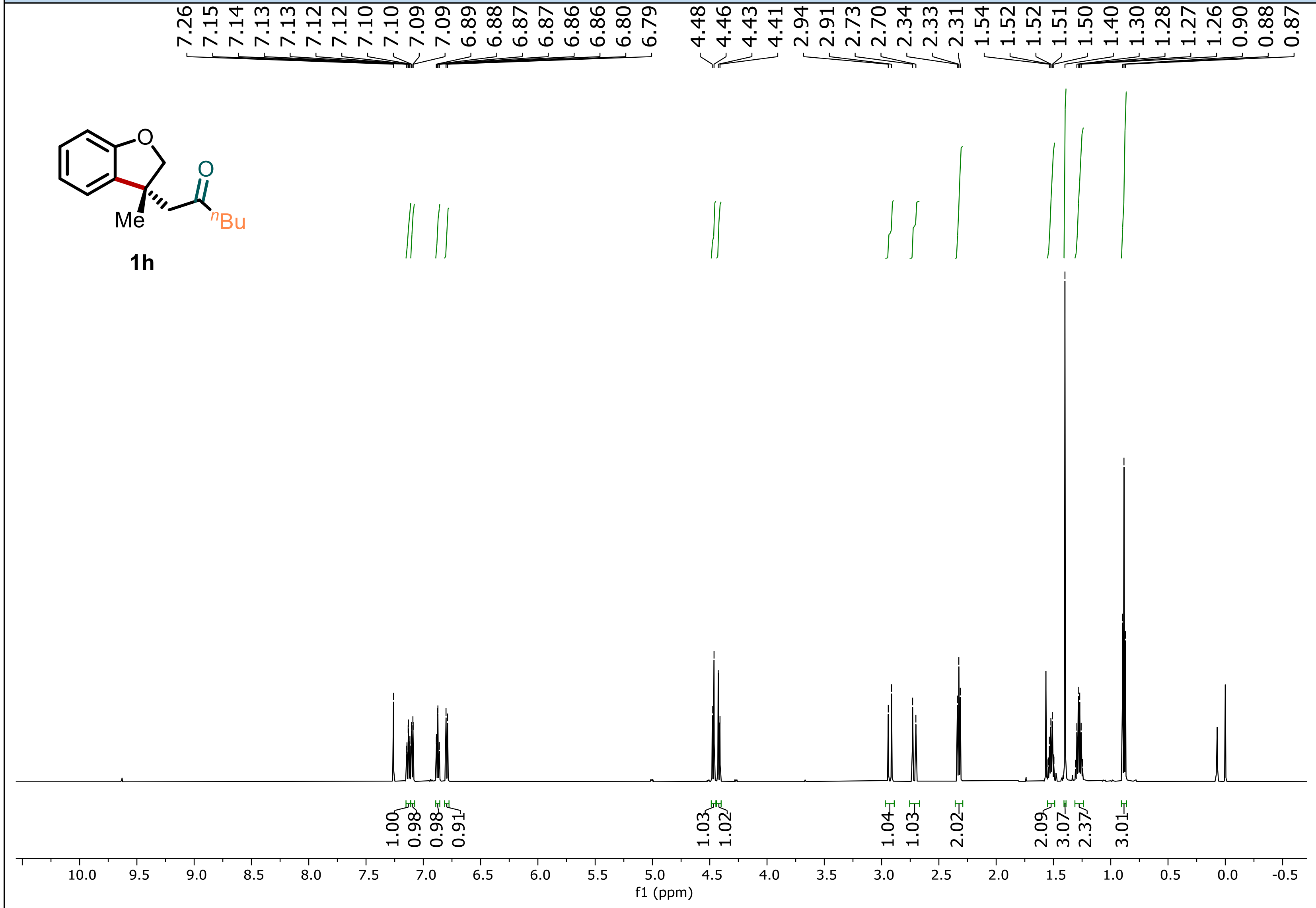

1h – $^{13}C\{^{1}H\}$ NMR (151 MHz, $CDCl_3$)

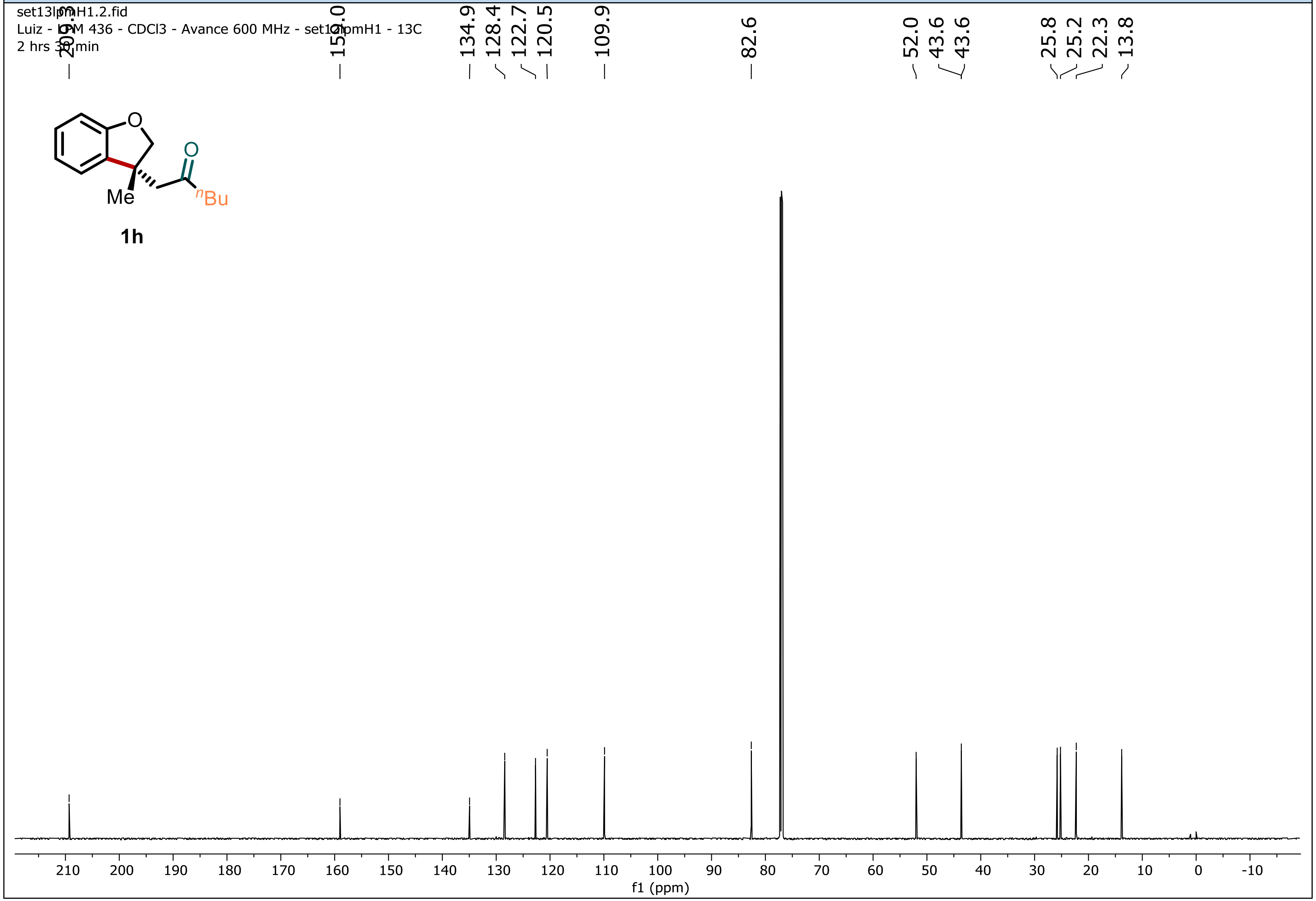

1i – ^{1}H NMR (500 MHz, $CDCl_3$)

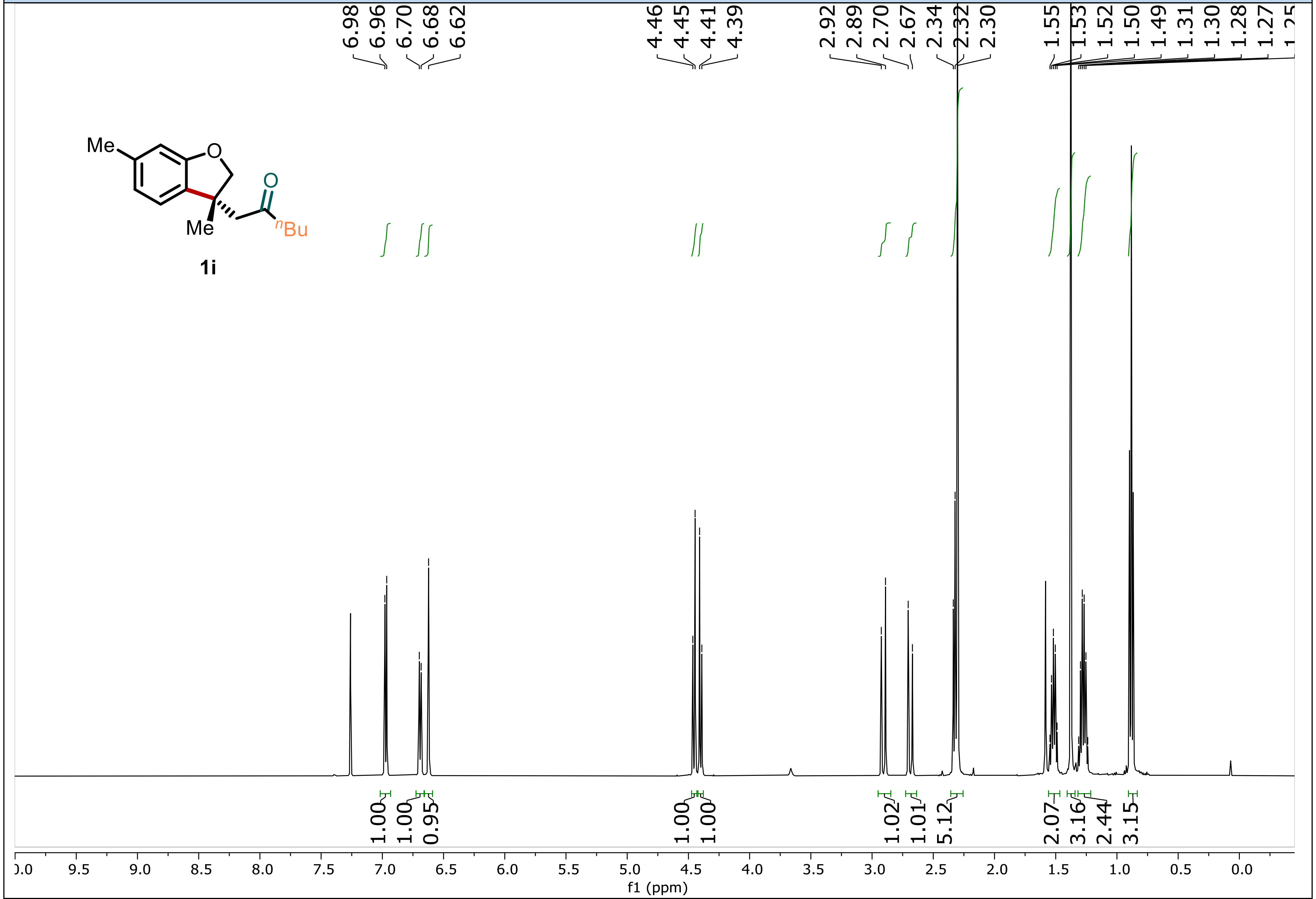

1i – $^{13}C\{^1H\}$ NMR (126 MHz, $CDCl_3$)

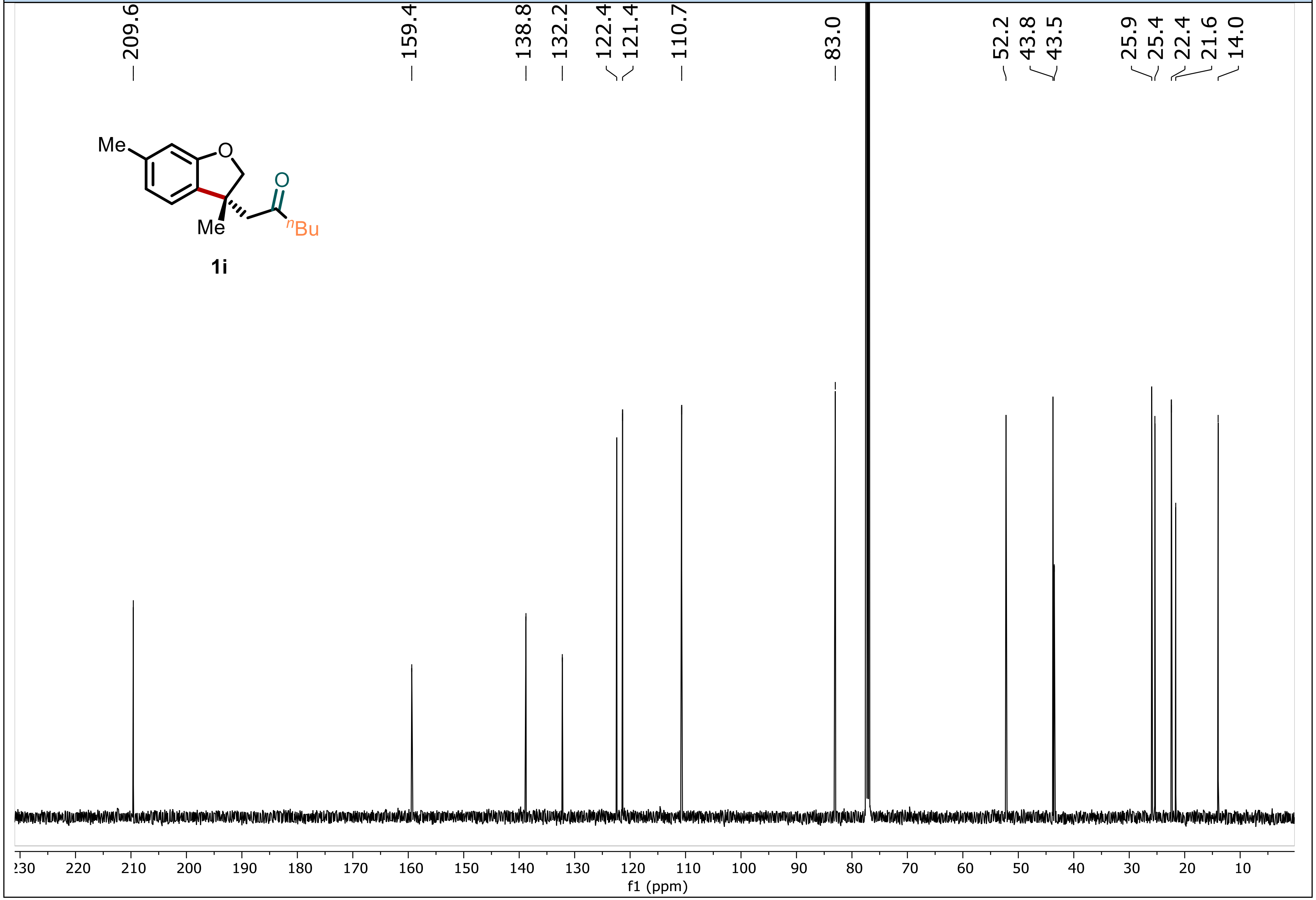

1j – ^{1}H NMR (600 MHz, CDCl$_3$)

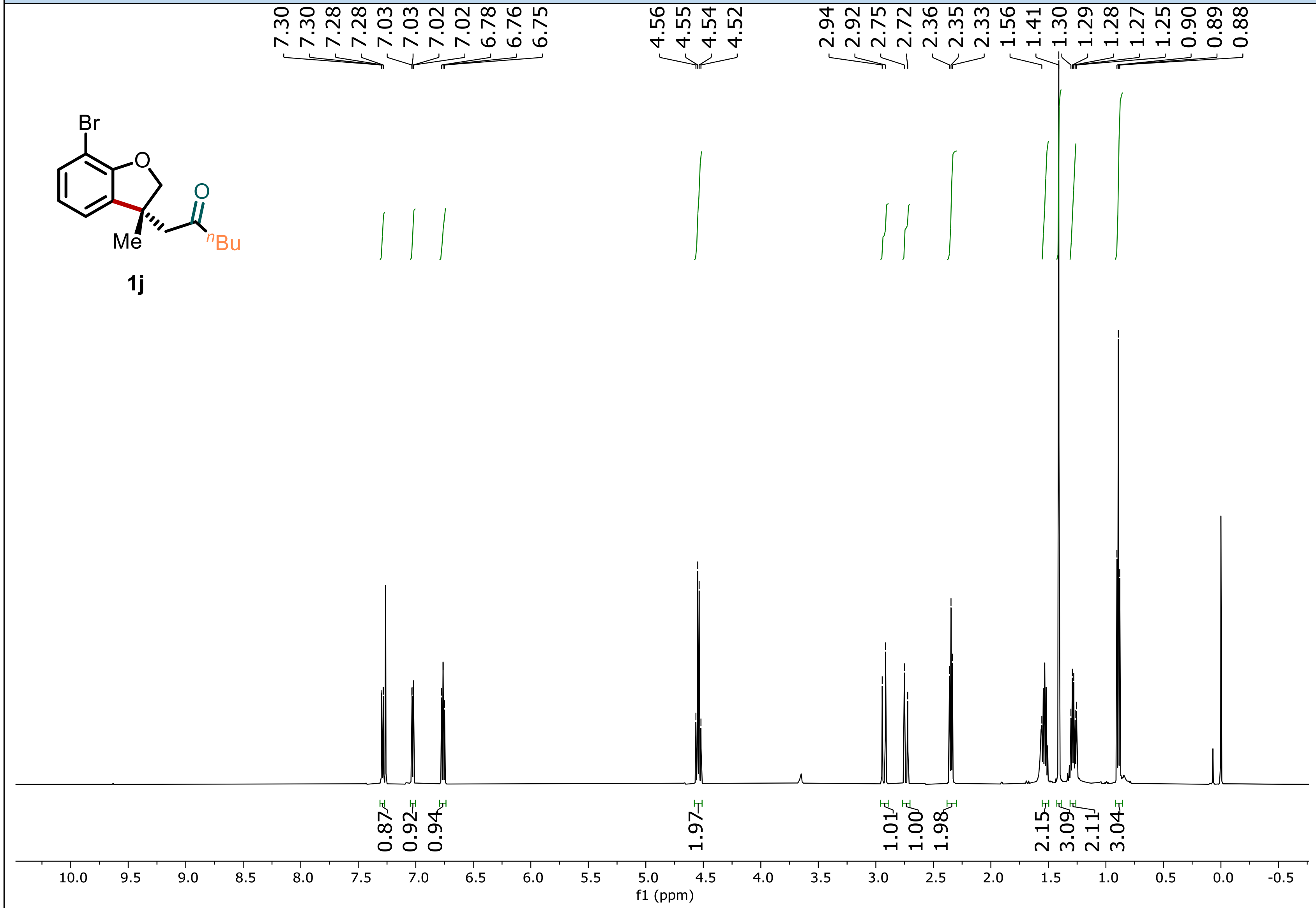

1j – $^{13}C\{^{1}H\}$ NMR (151 MHz, $CDCl_3$)

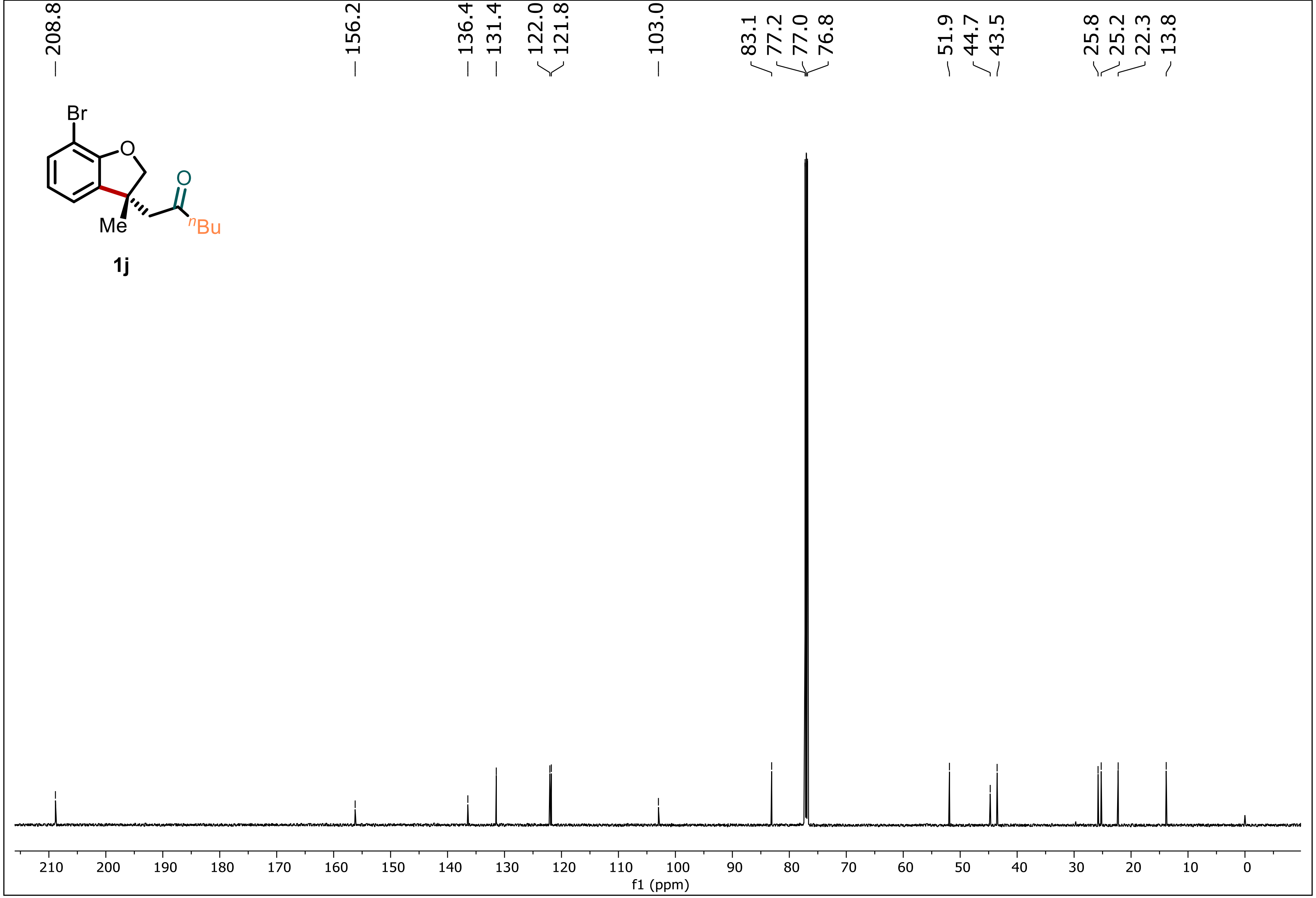

1k – ^{1}H NMR (500 MHz, $CDCl_3$)

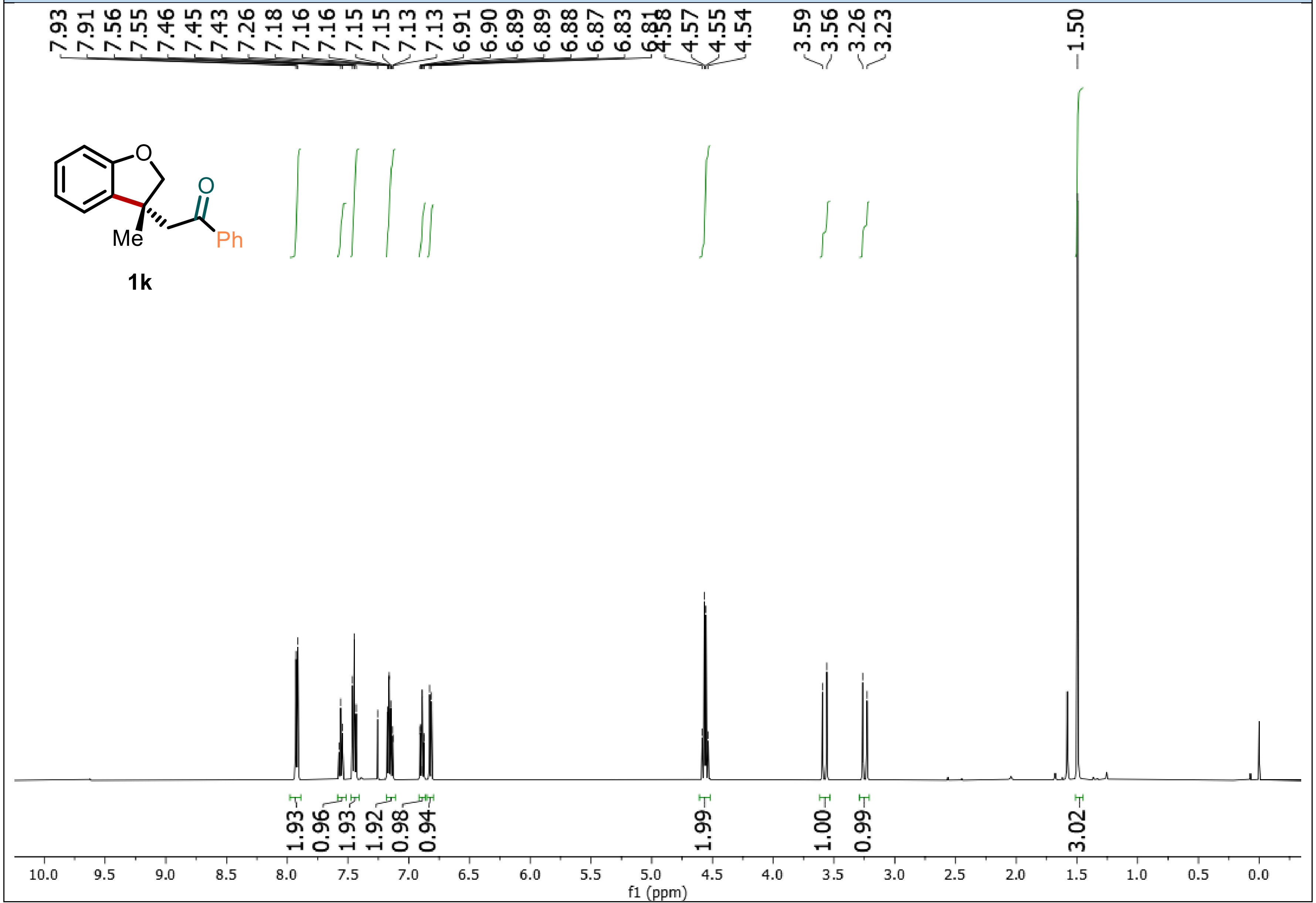

1k – $^{13}C\{^1H\}$ NMR (126 MHz, $CDCl_3$)

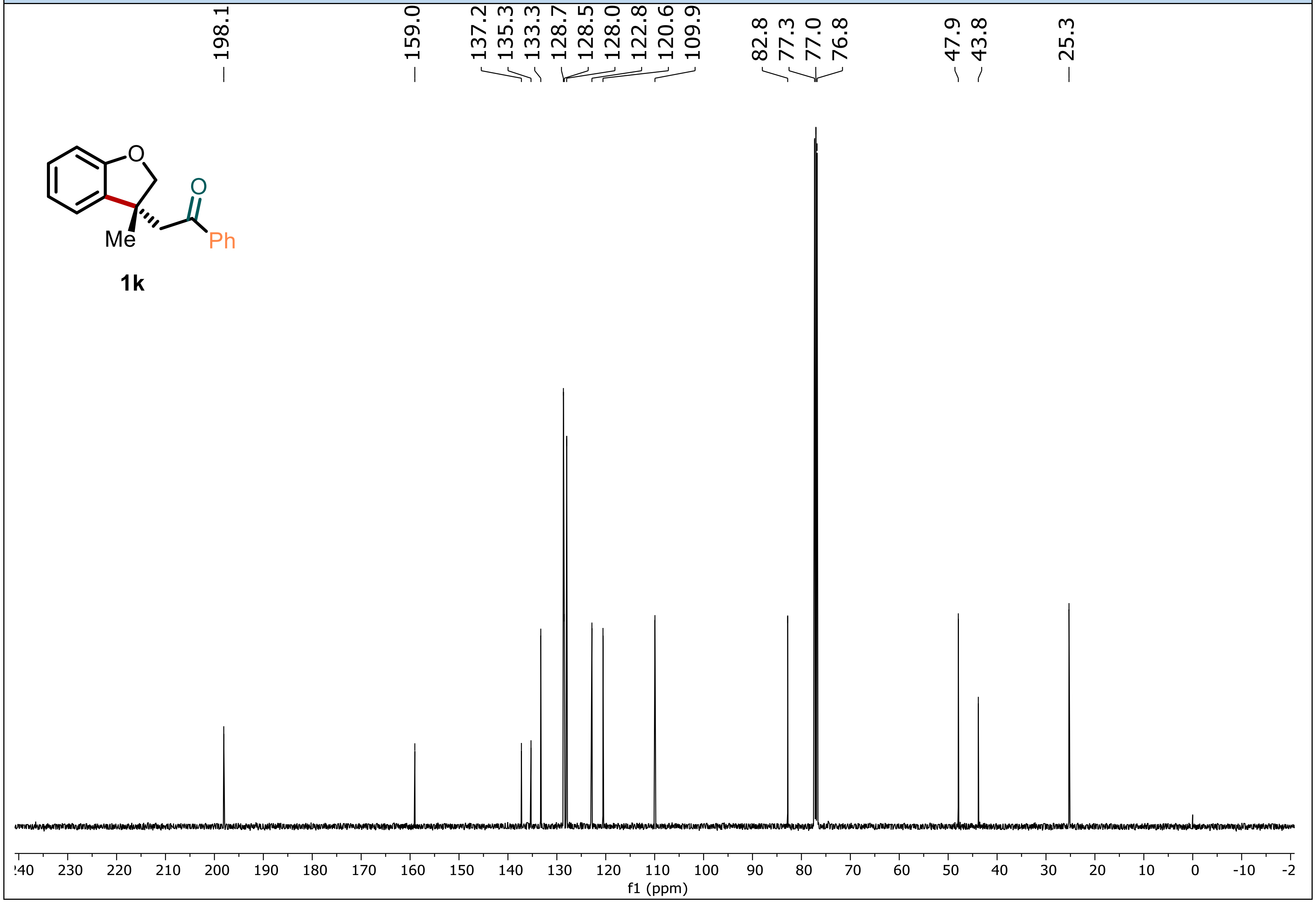

1l – ^{1}H NMR (500 MHz, $CDCl_3$)

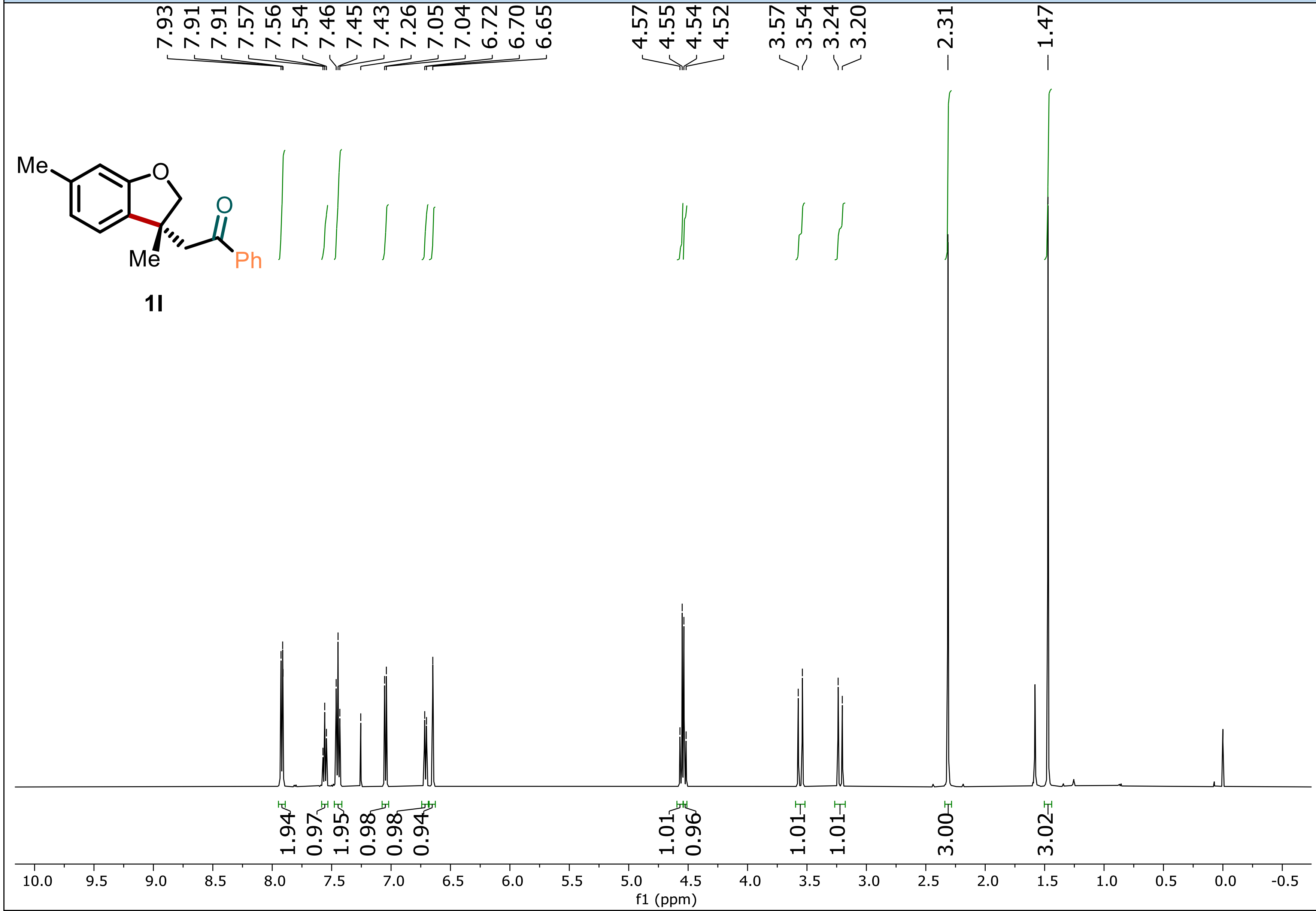

1l – $^{13}C\{^{1}H\}$ NMR (126 MHz, $CDCl_3$)

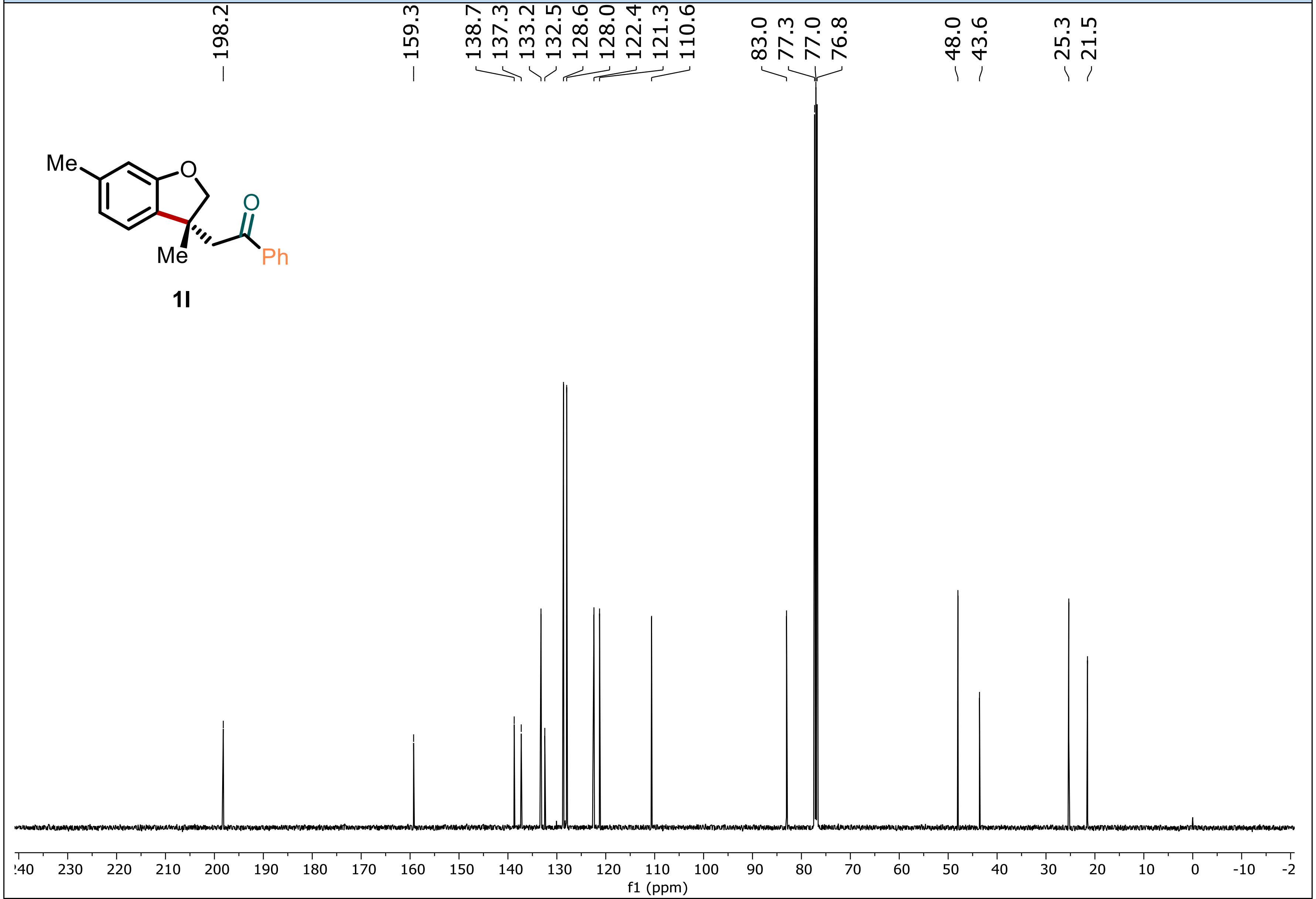

1m – ^{1}H NMR (600 MHz, CDCl₃)

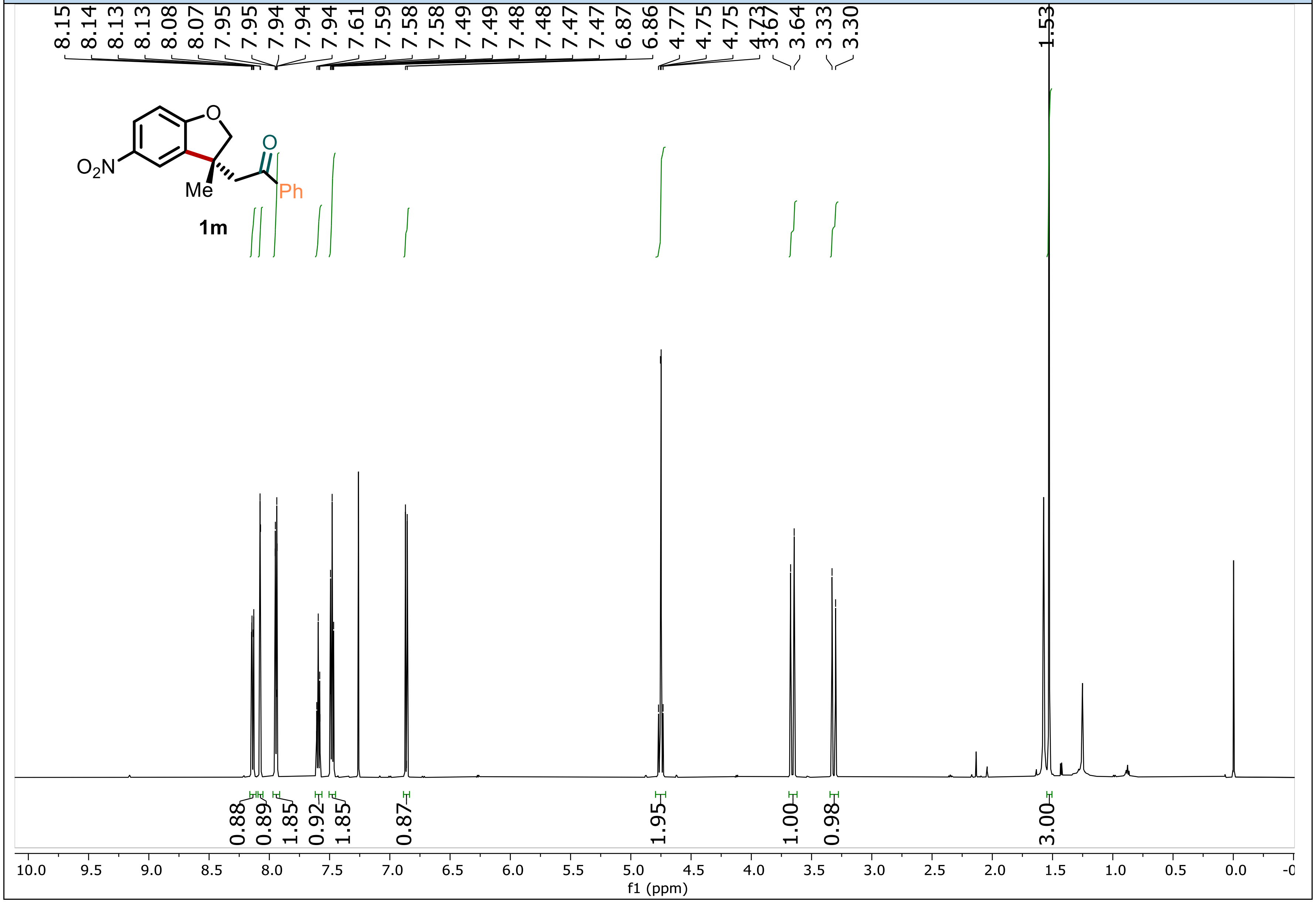

1m – $^{13}C\{^{1}H\}$ NMR (151 MHz, $CDCl_3$)

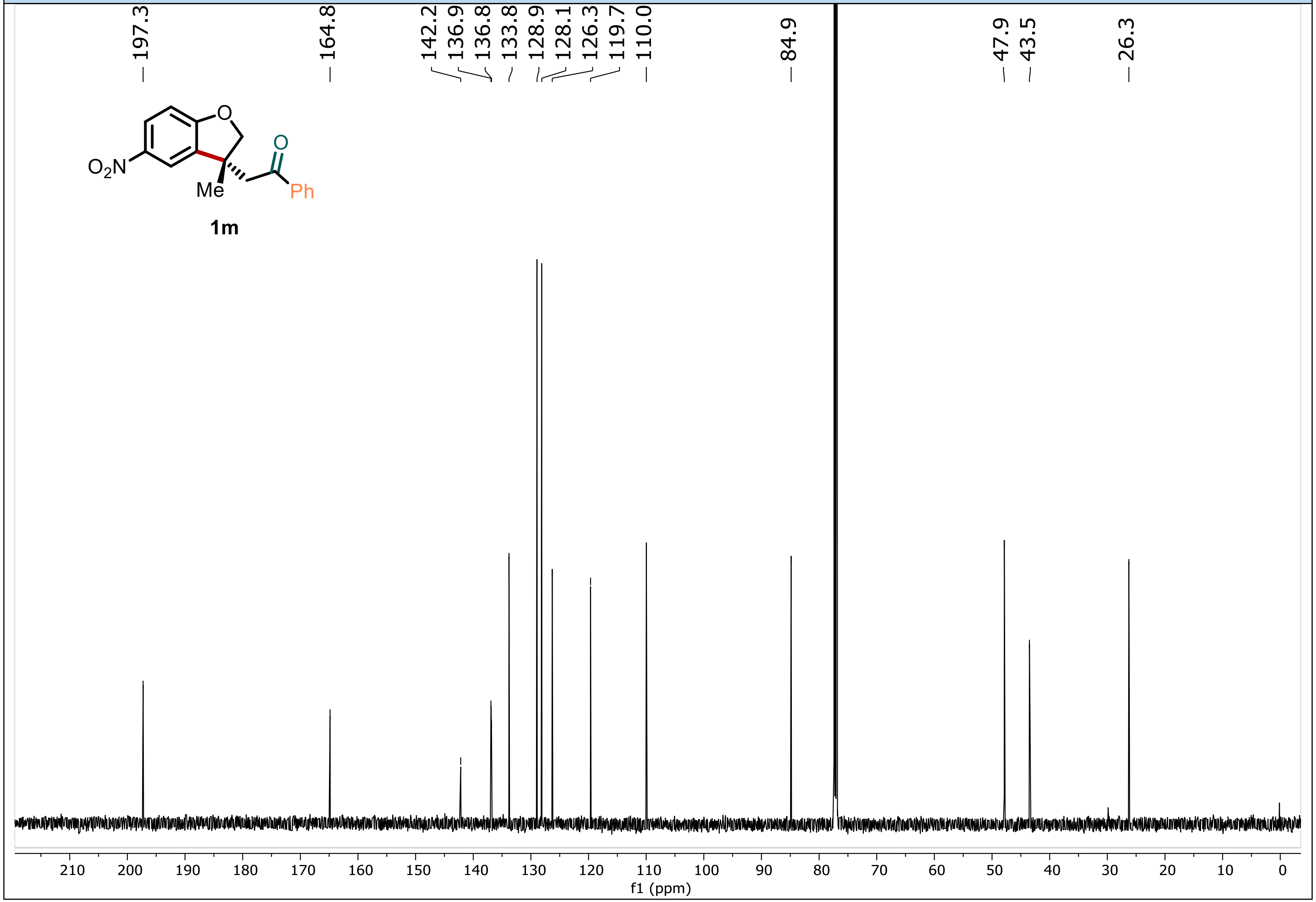

1n – ^{1}H NMR (500 MHz, $CDCl_3$)

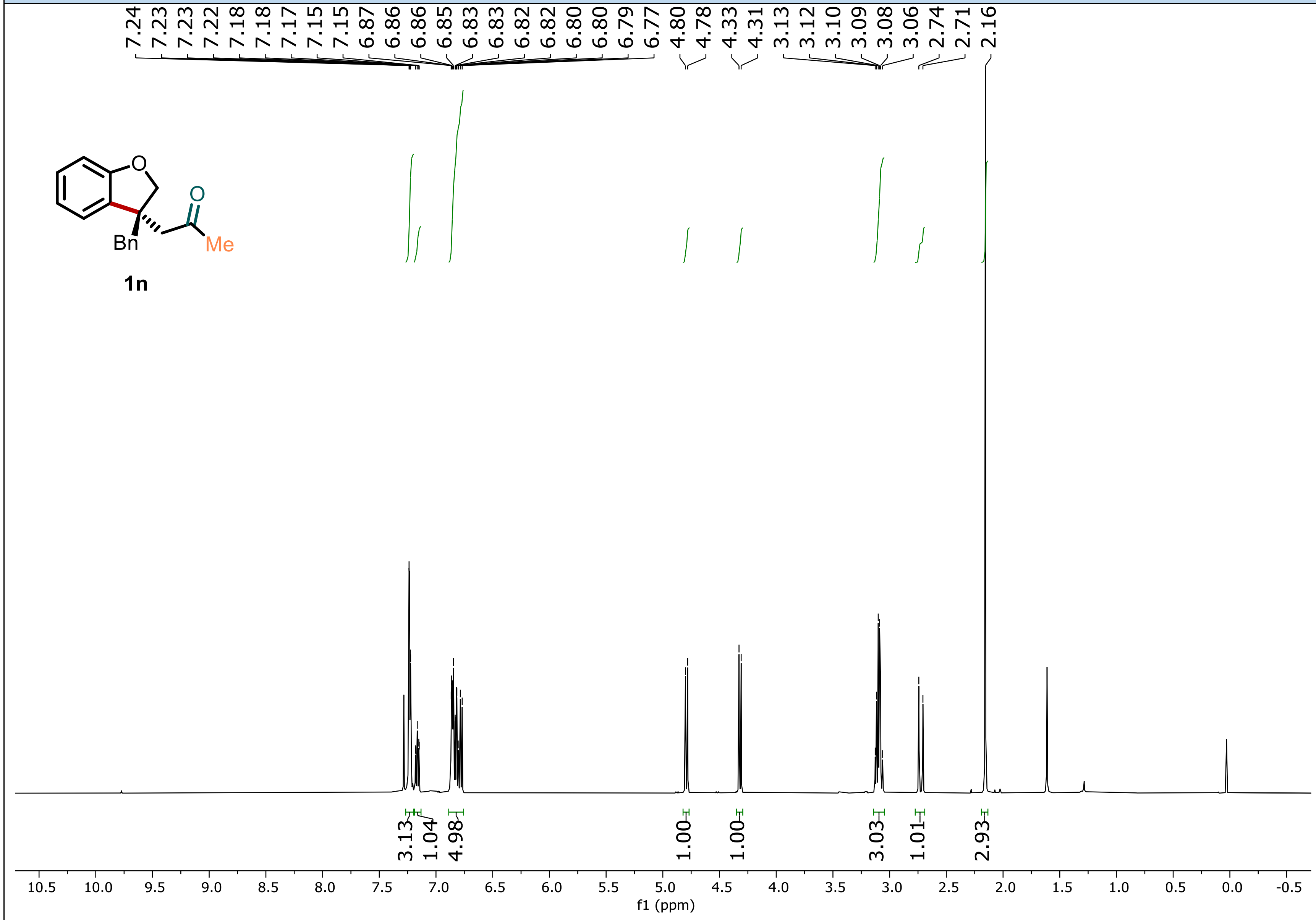

1n

1n – $^{13}C\{^{1}H\}$ NMR (126 MHz, CDCl$_3$)

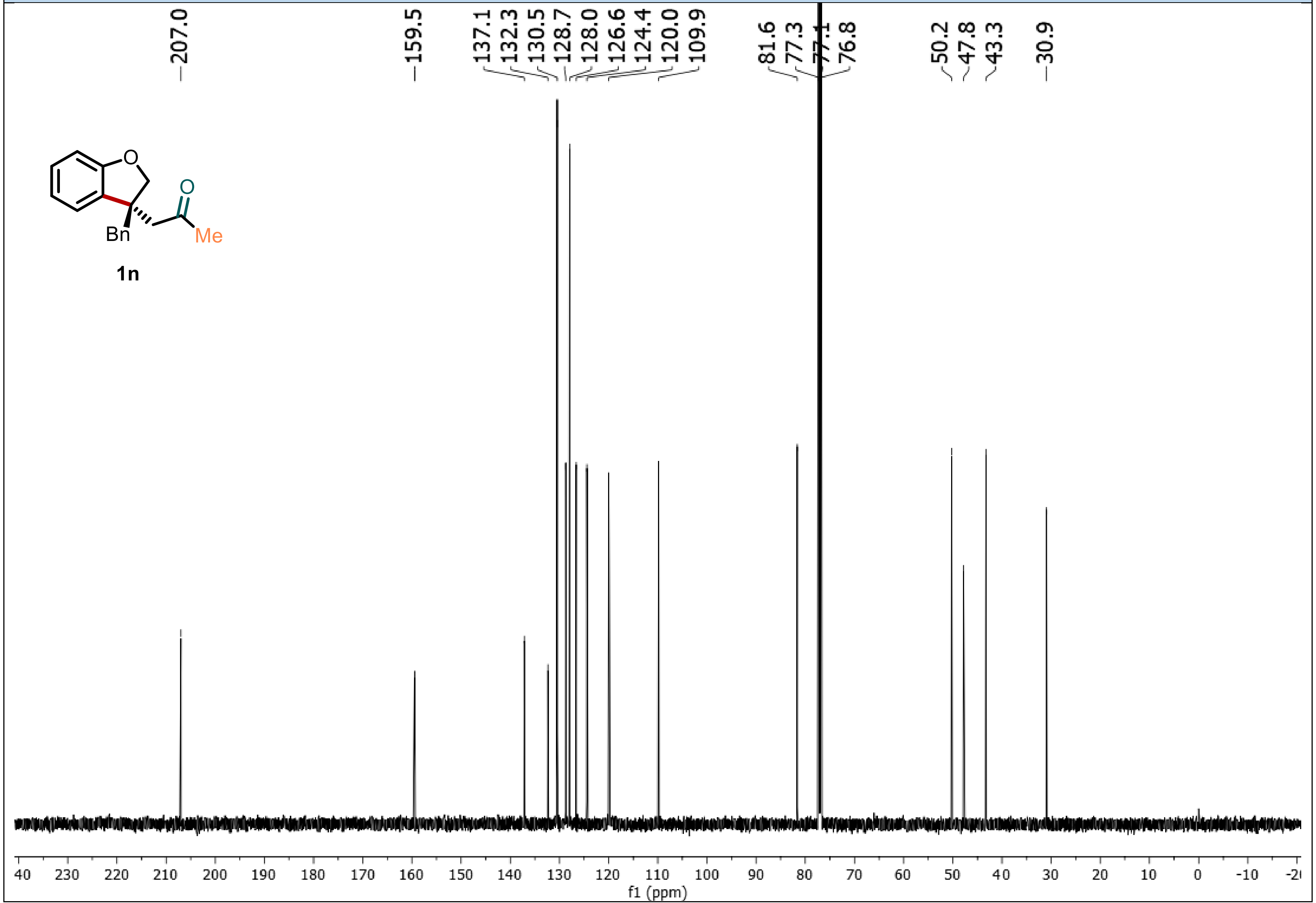

1o – ^{1}H NMR (300 MHz, $CDCl_3$)

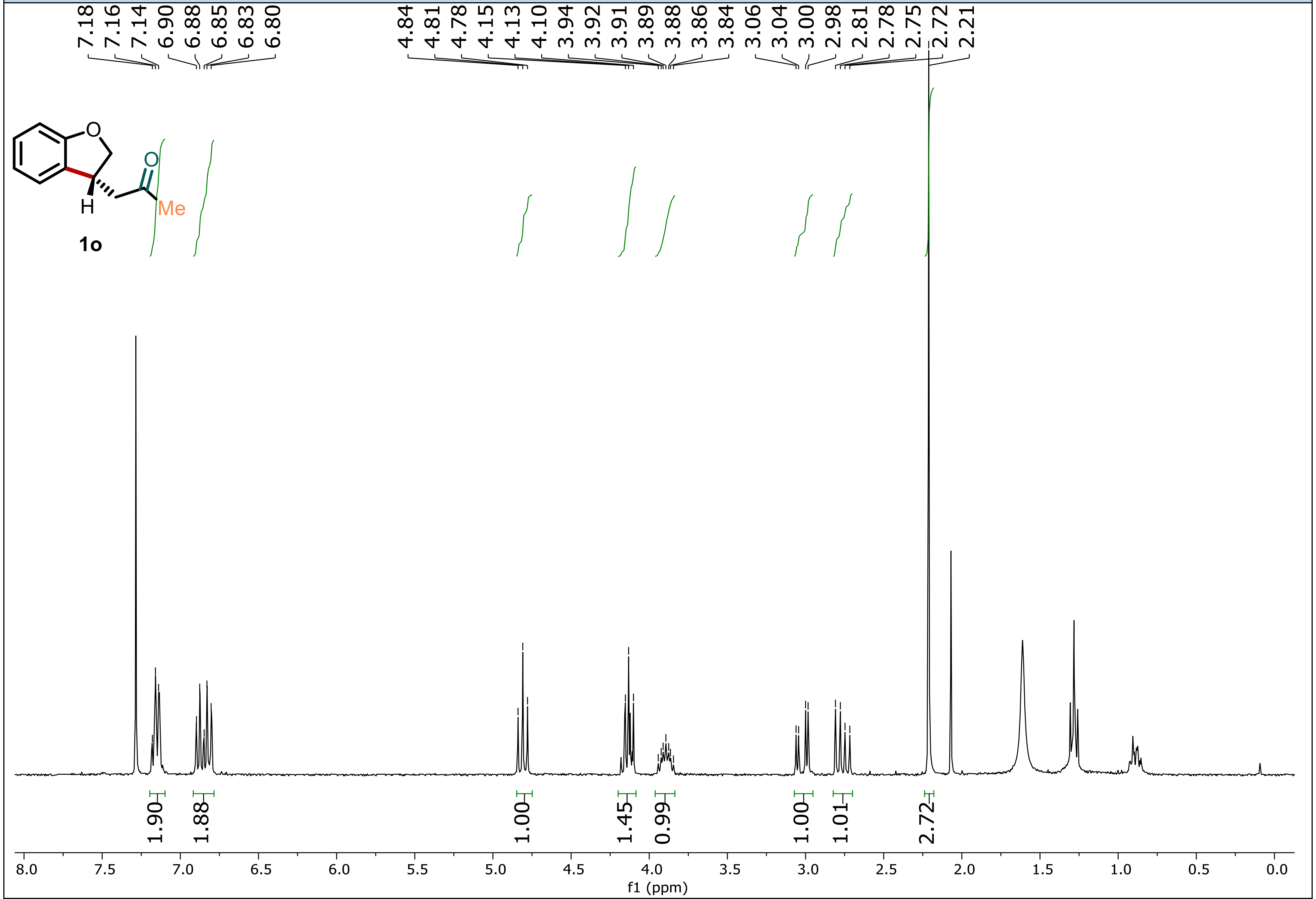

1o – $^{13}C\{^{1}H\}$ NMR (75 MHz, $CDCl_3$)

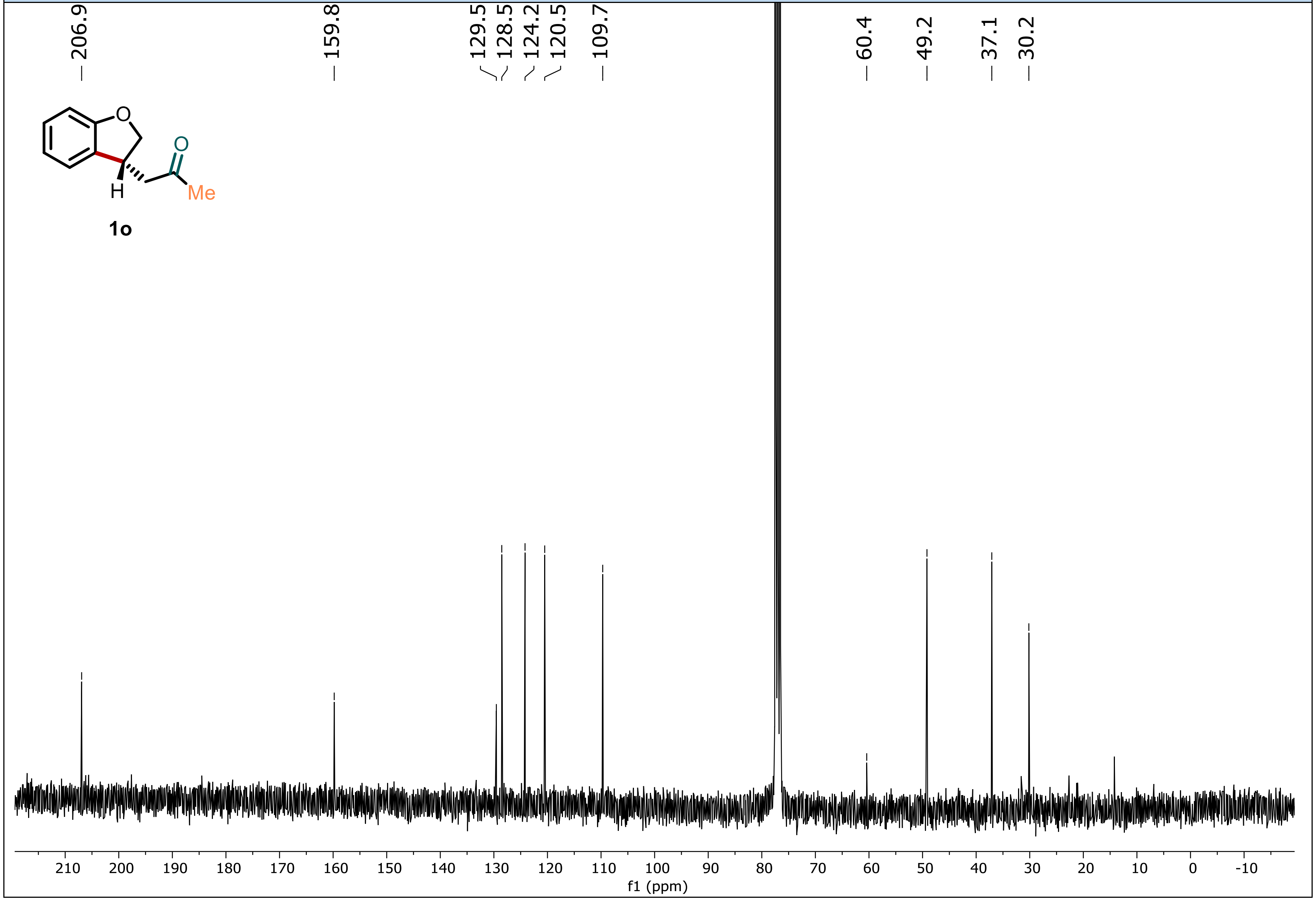

2a – ^{1}H NMR (500 MHz, CDCl$_3$)

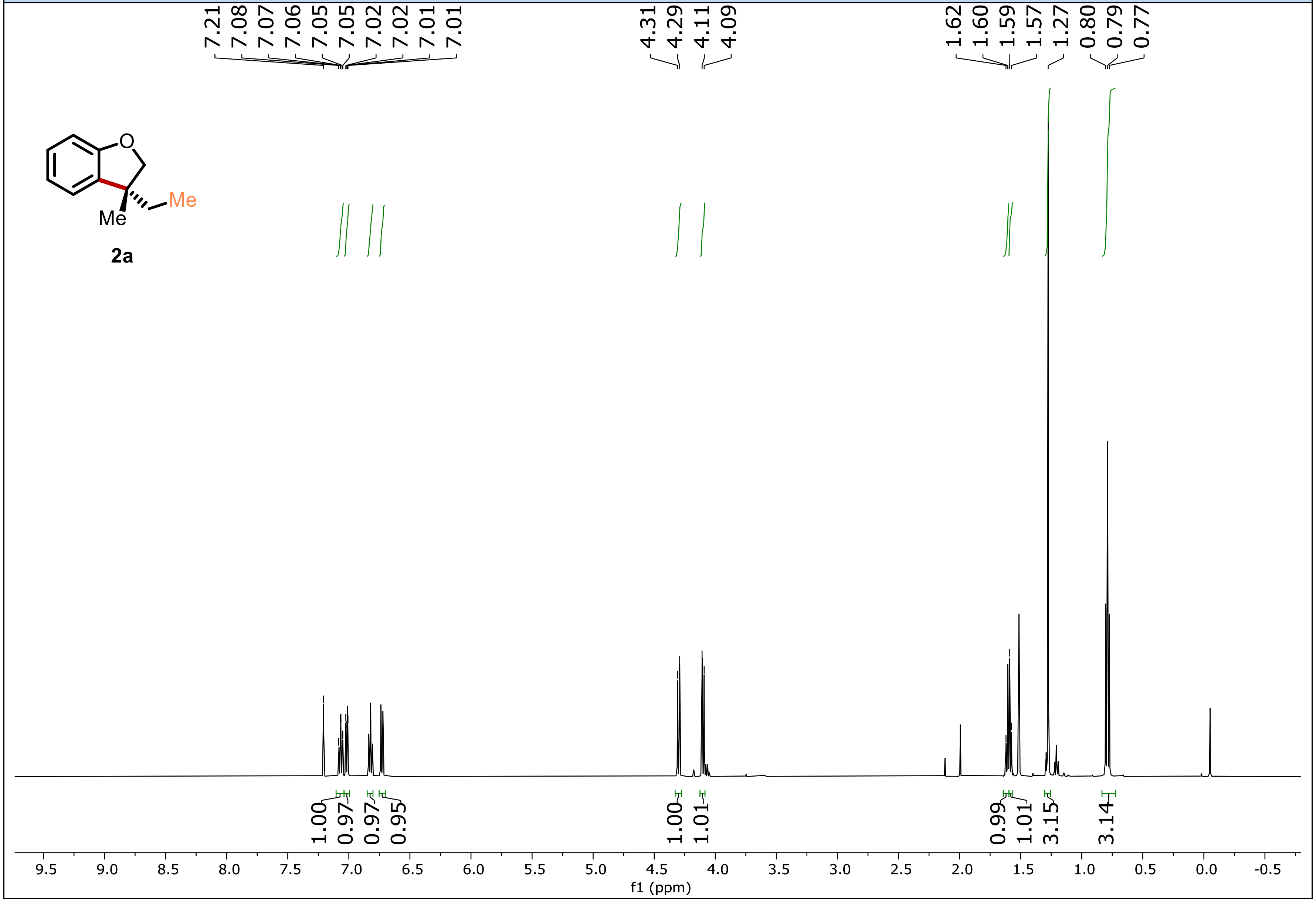

2a – $^{13}C\{^{1}H\}$ NMR (126 MHz, $CDCl_3$)

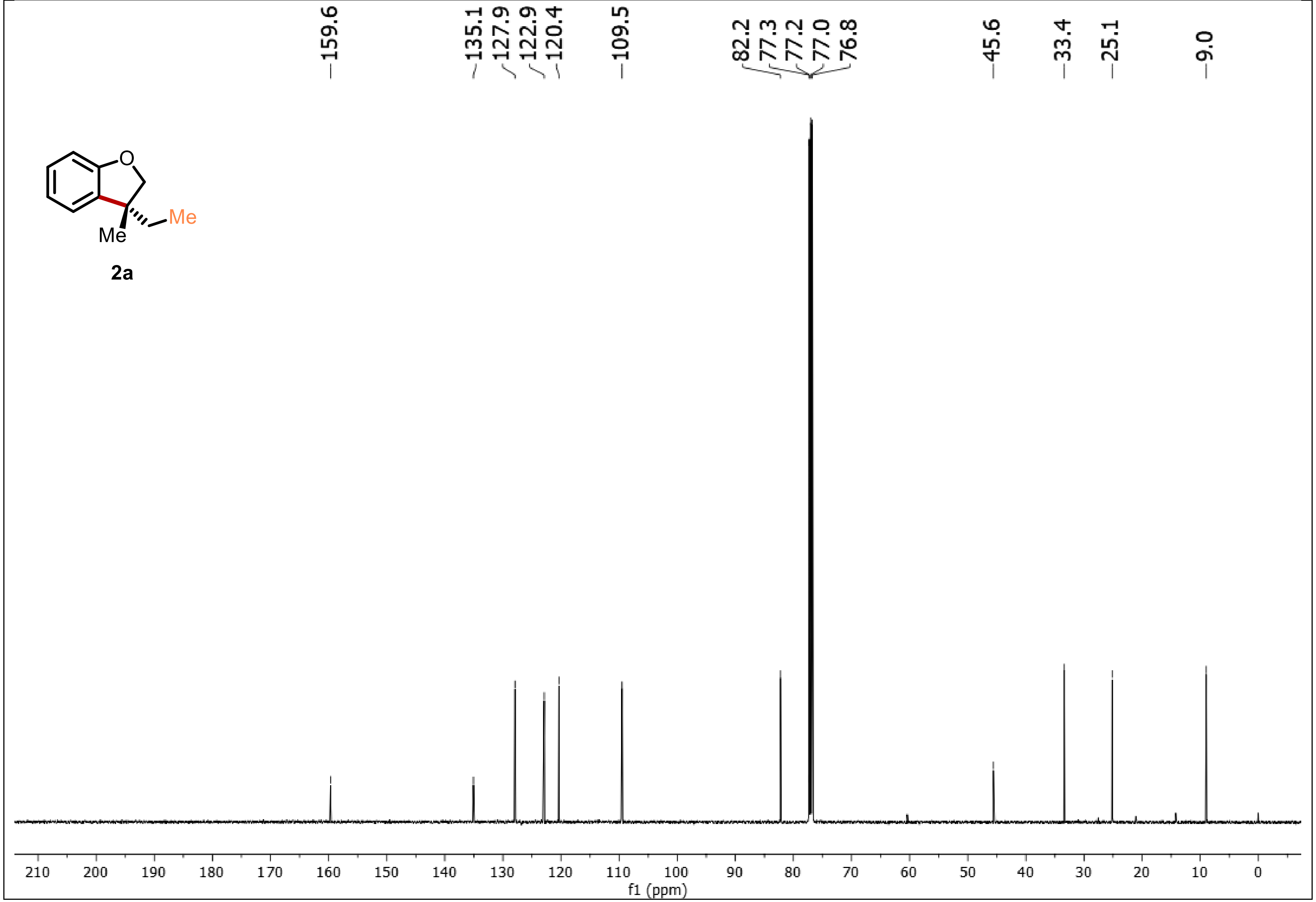

2b – ^{1}H NMR (300 MHz, $CDCl_3$)

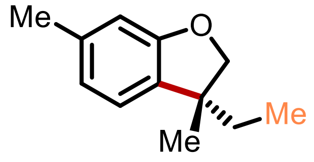

2b

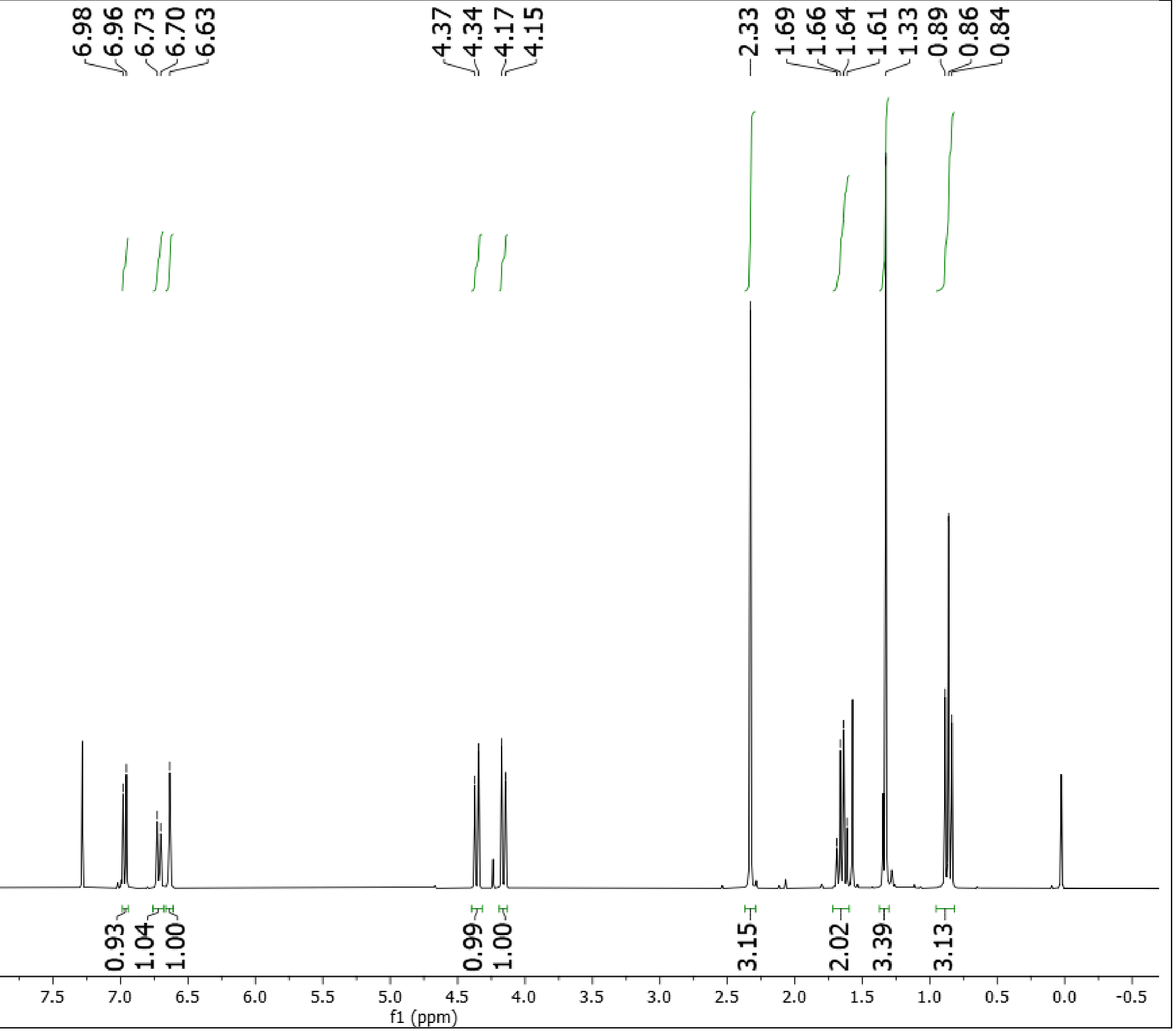

2b – $^{13}C\{^{1}H\}$ NMR (75 MHz, $CDCl_3$)

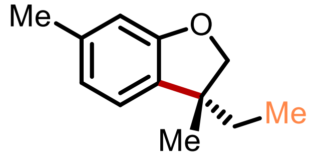

2b

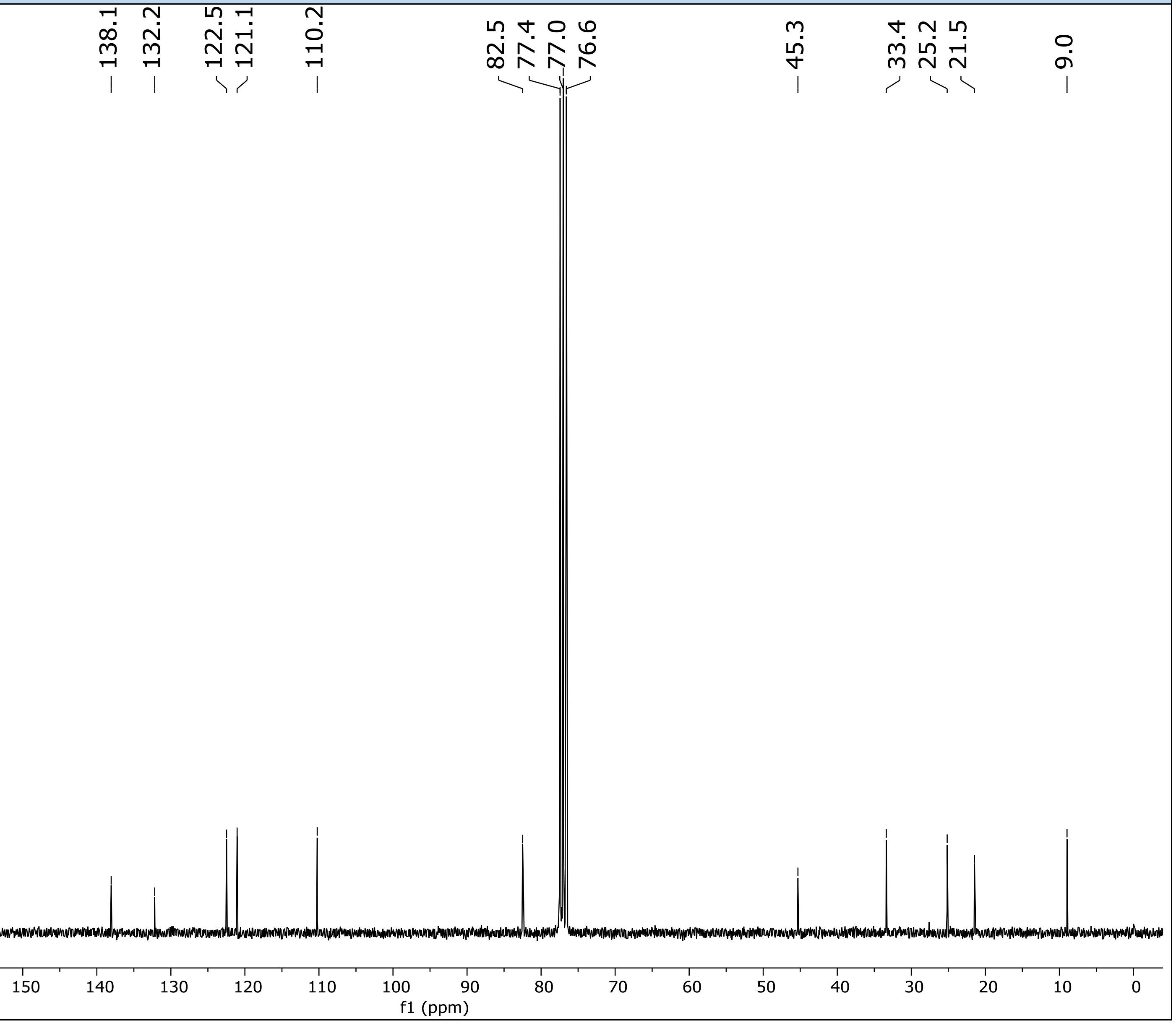

2c – ^{1}H NMR (400 MHz, $CDCl_3$)

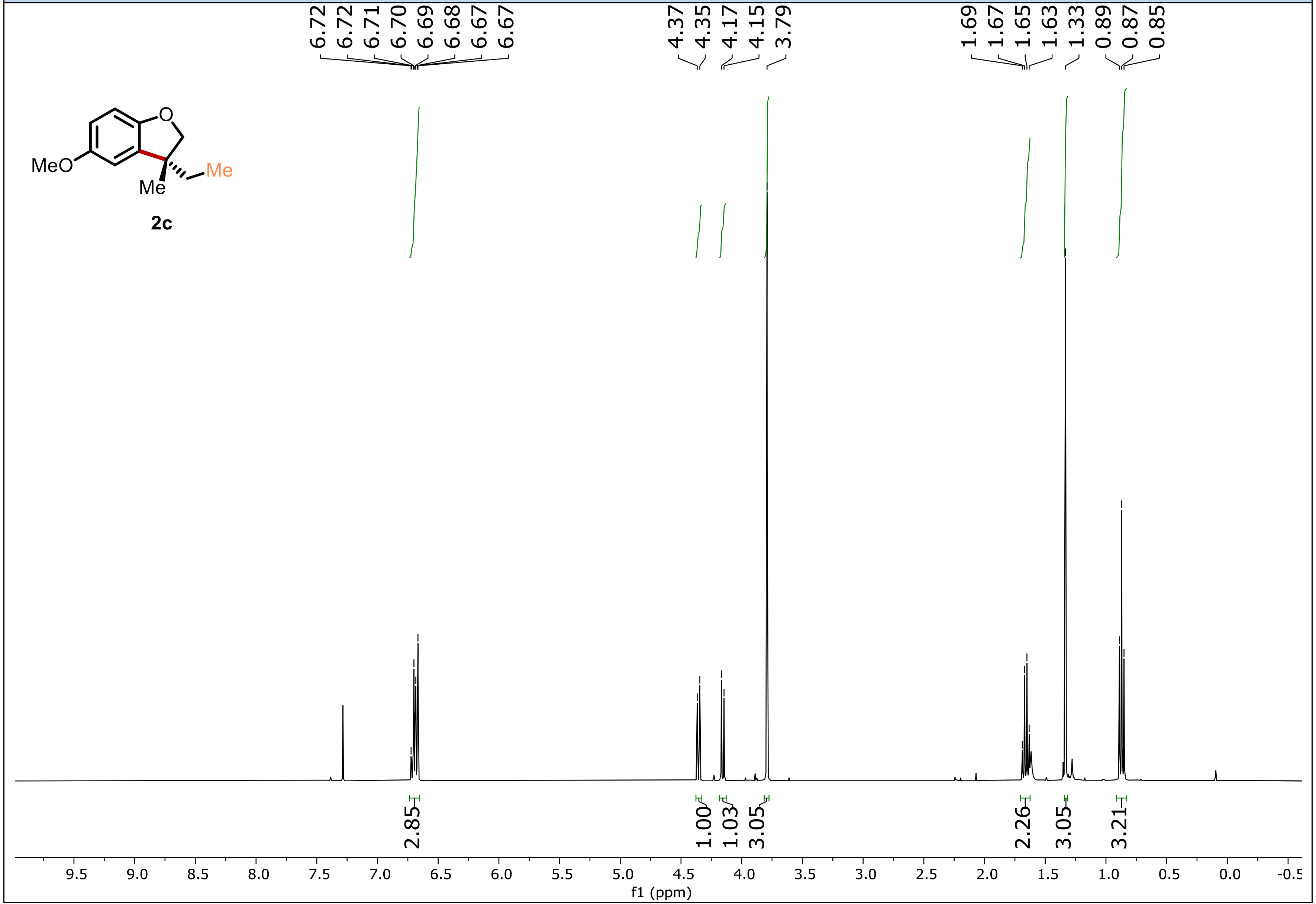

2c – $^{13}C\{^1H\}$ NMR (101 MHz, $CDCl_3$)

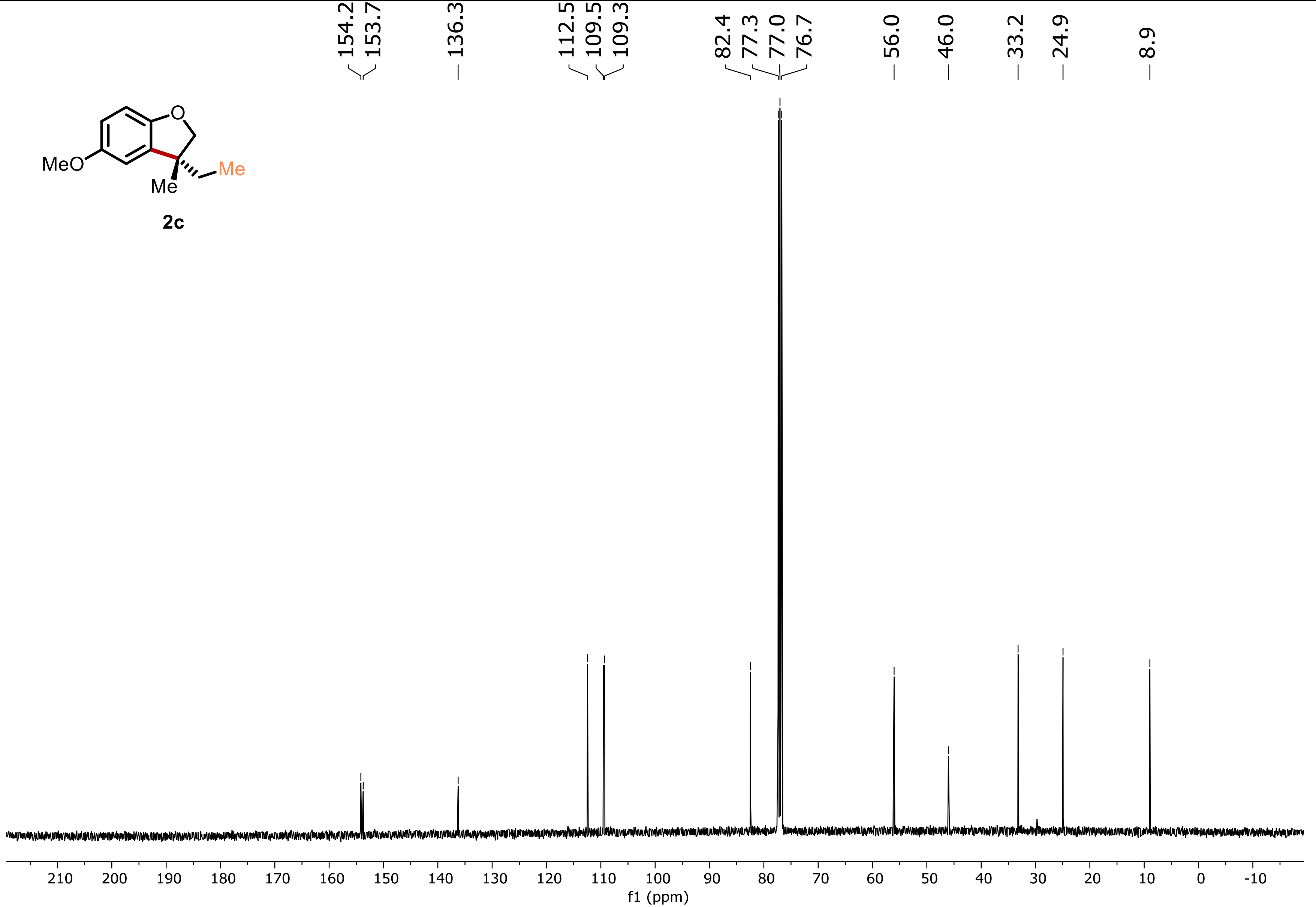

2d – ^{1}H NMR (600 MHz, $CDCl_3$)

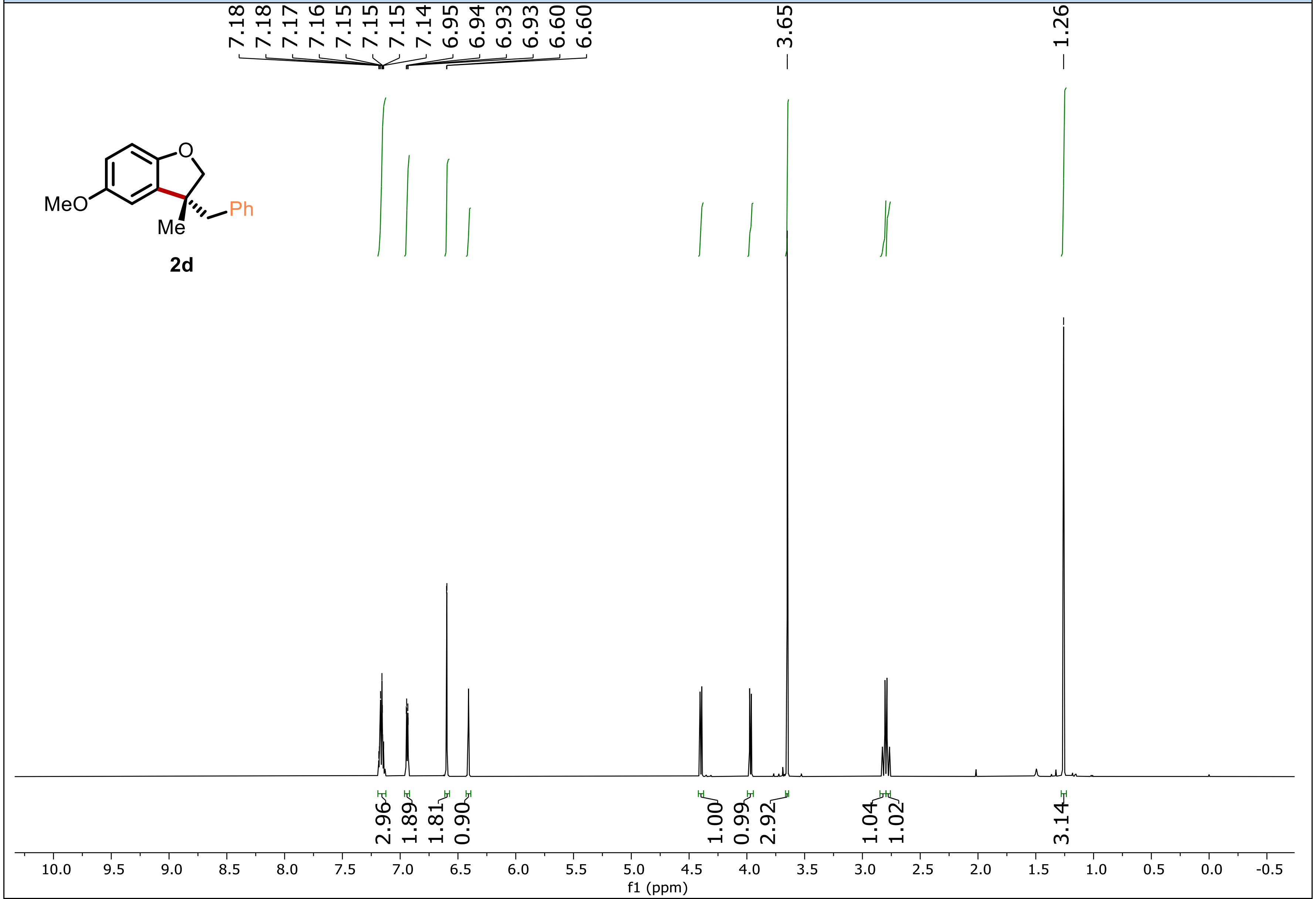

2d – $^{13}C\{^{1}H\}$ NMR (151 MHz, $CDCl_3$)

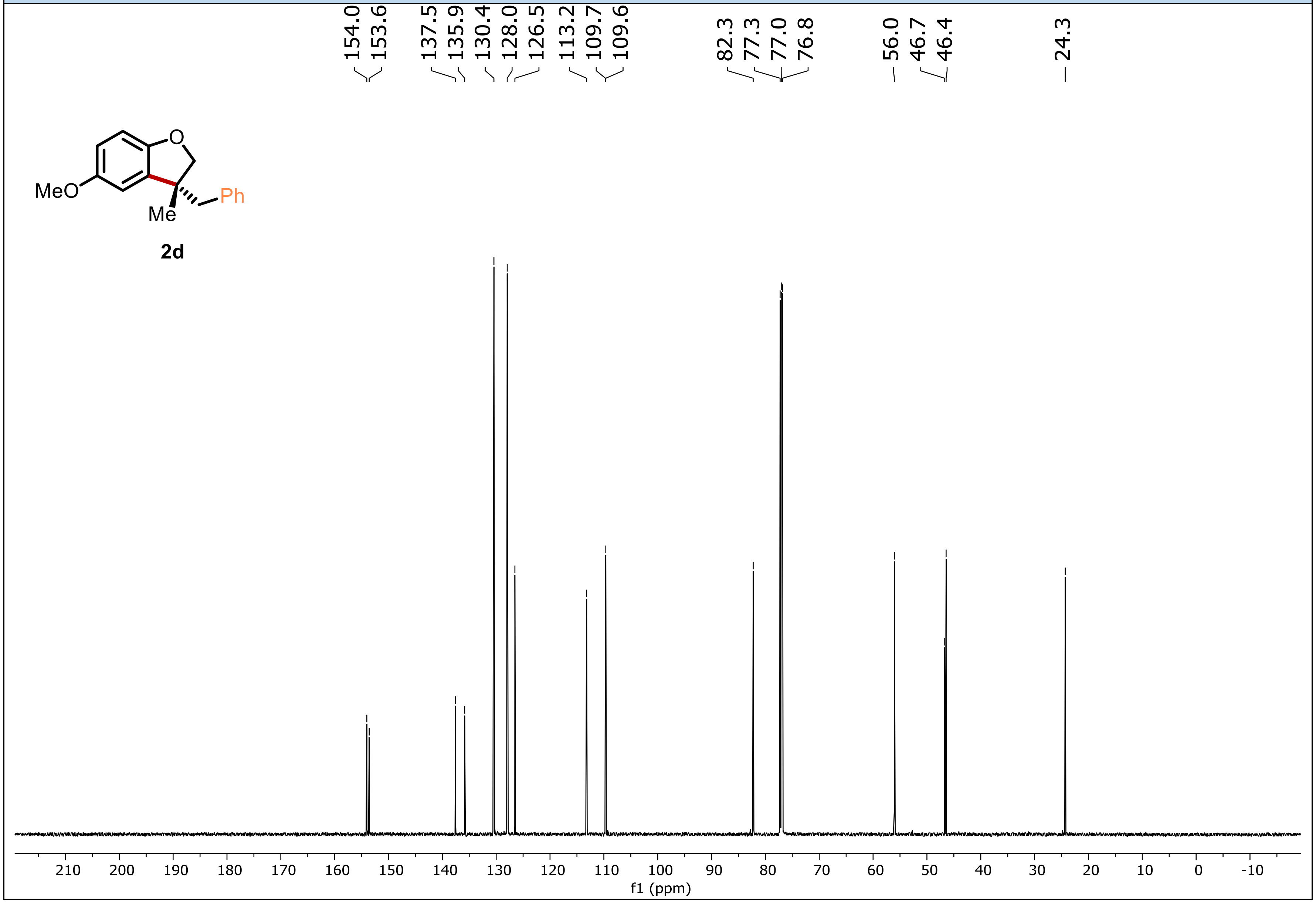

2e – ^{1}H NMR (600 MHz, $CDCl_3$)

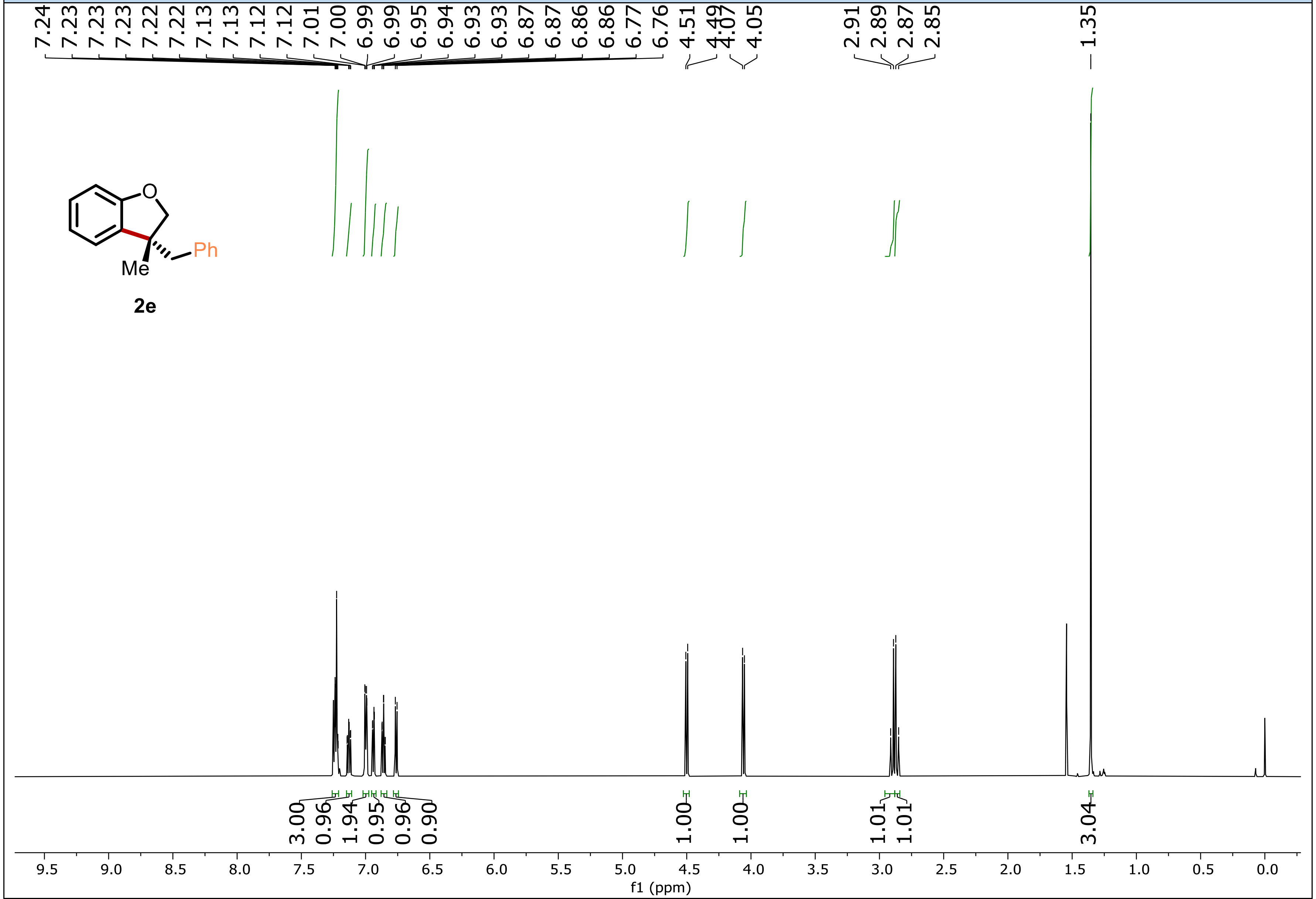

2d – $^{13}C\{^1H\}$ NMR (151 MHz, $CDCl_3$)

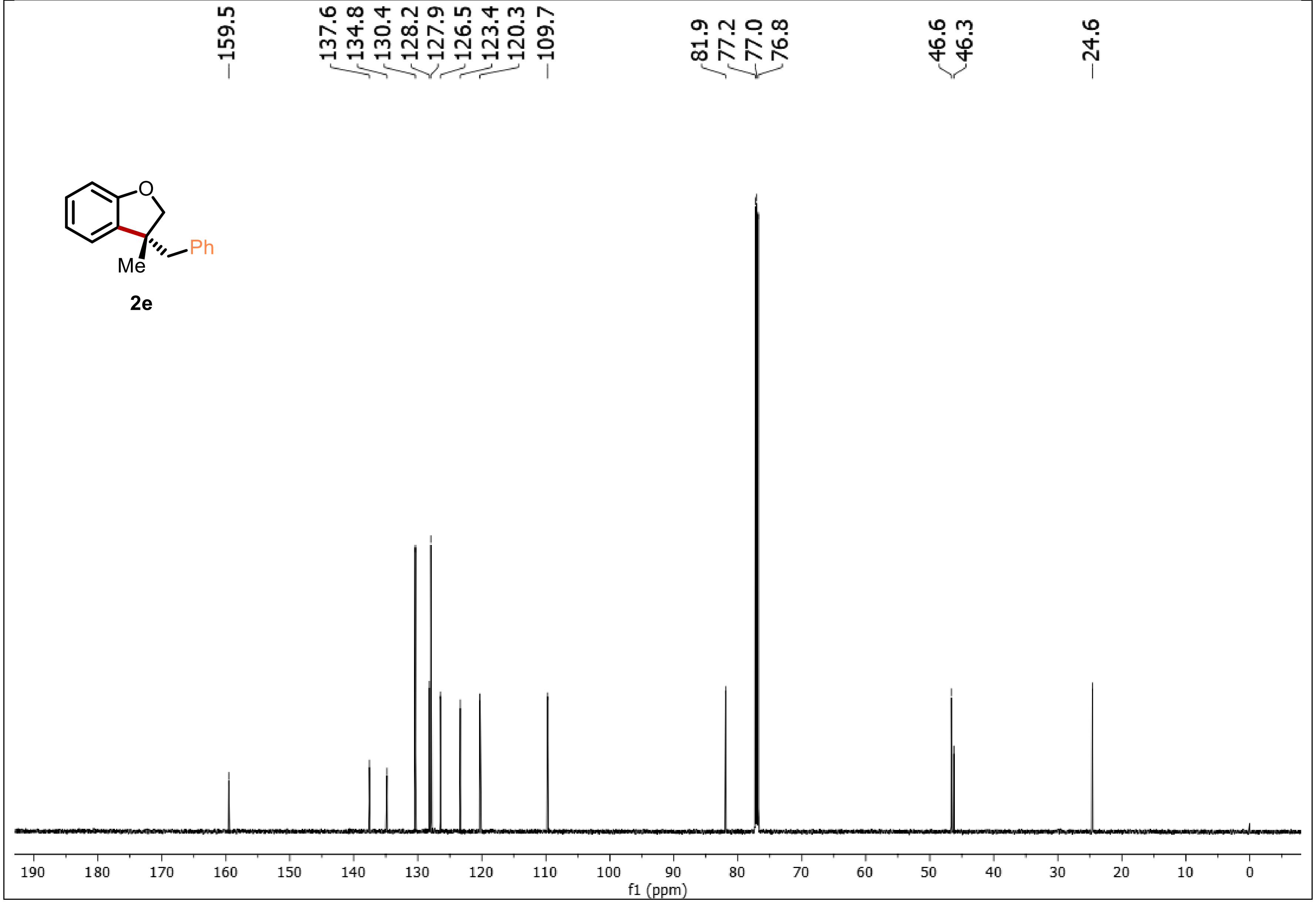

2f – ^{1}H NMR (300 MHz, $CDCl_3$)

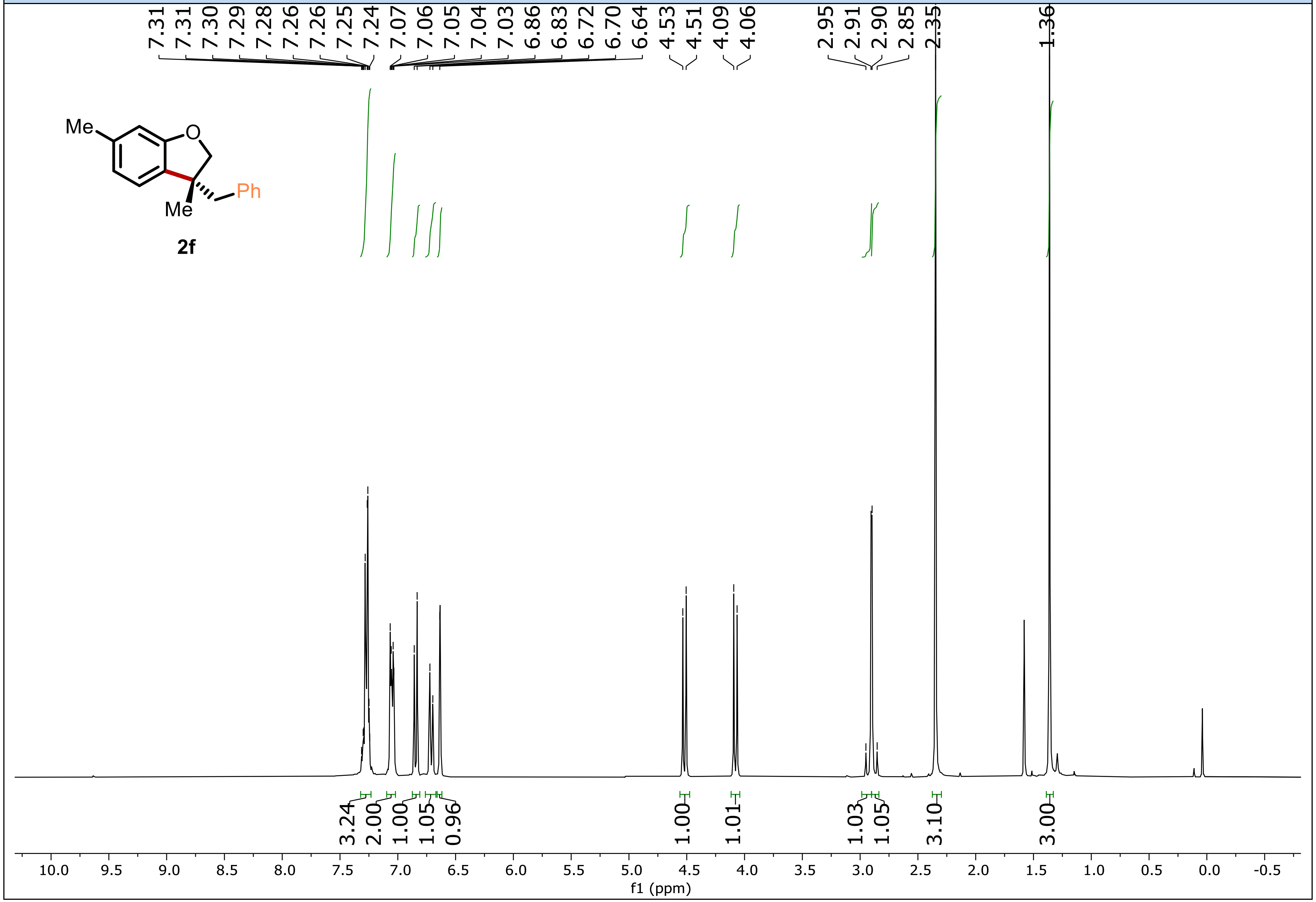

2f – $^{13}C\{^{1}H\}$ NMR (75 MHz, CDCl$_3$)

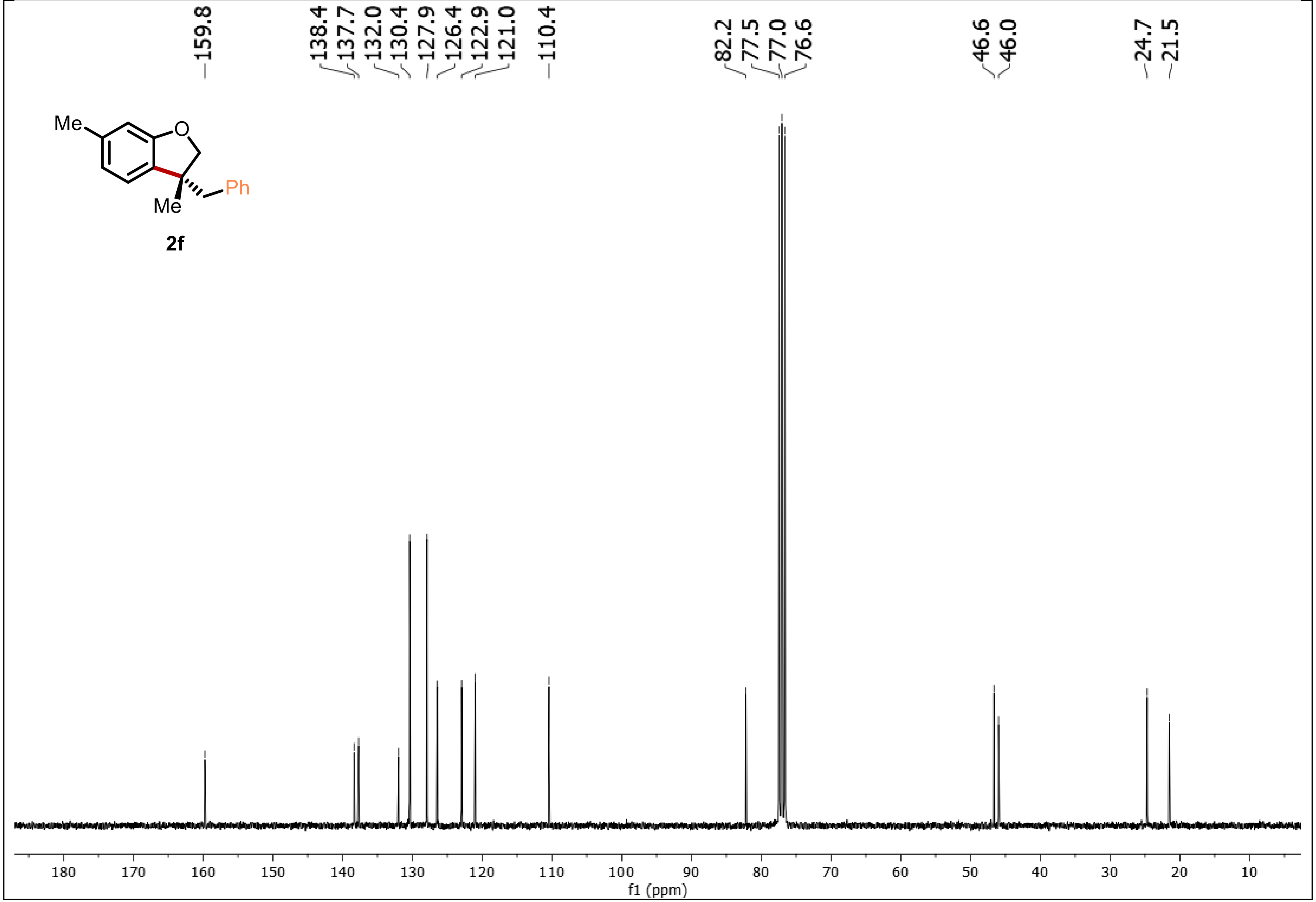

2g – ^{1}H NMR (300 MHz, $CDCl_3$)

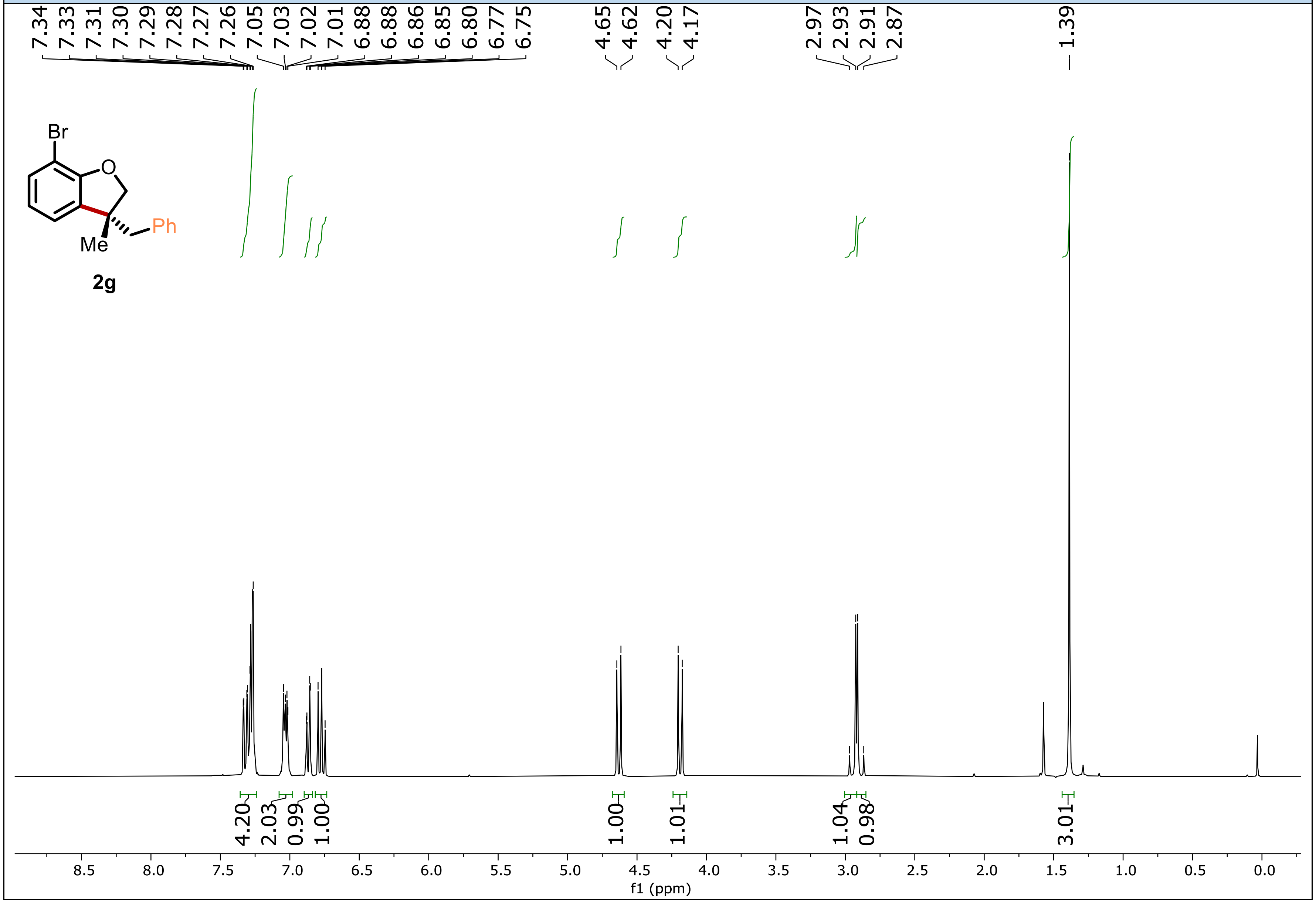

2g – $^{13}C\{^{1}H\}$ NMR (75 MHz, $CDCl_3$)

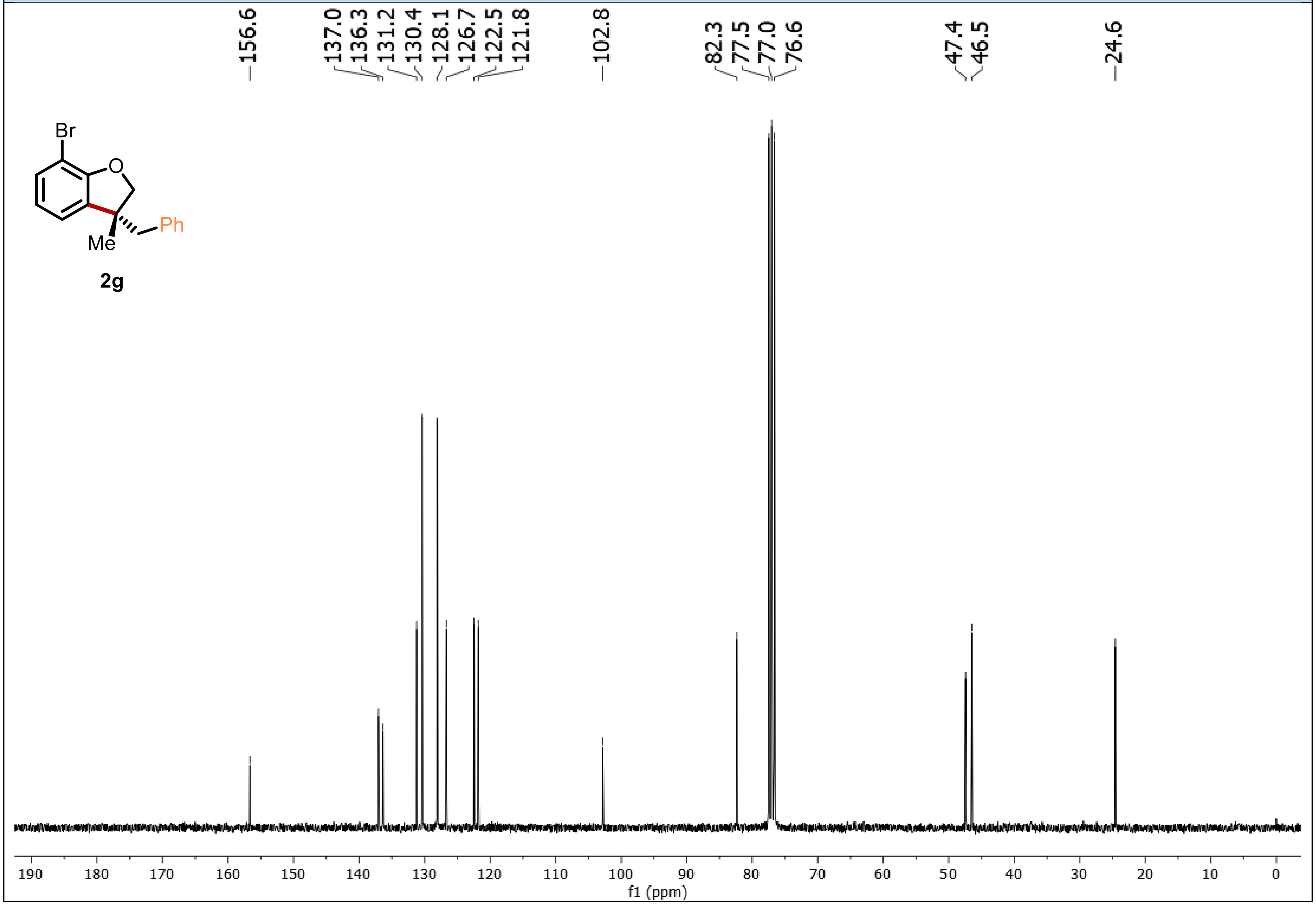

2h – ^{1}H NMR (300 MHz, $CDCl_3$)

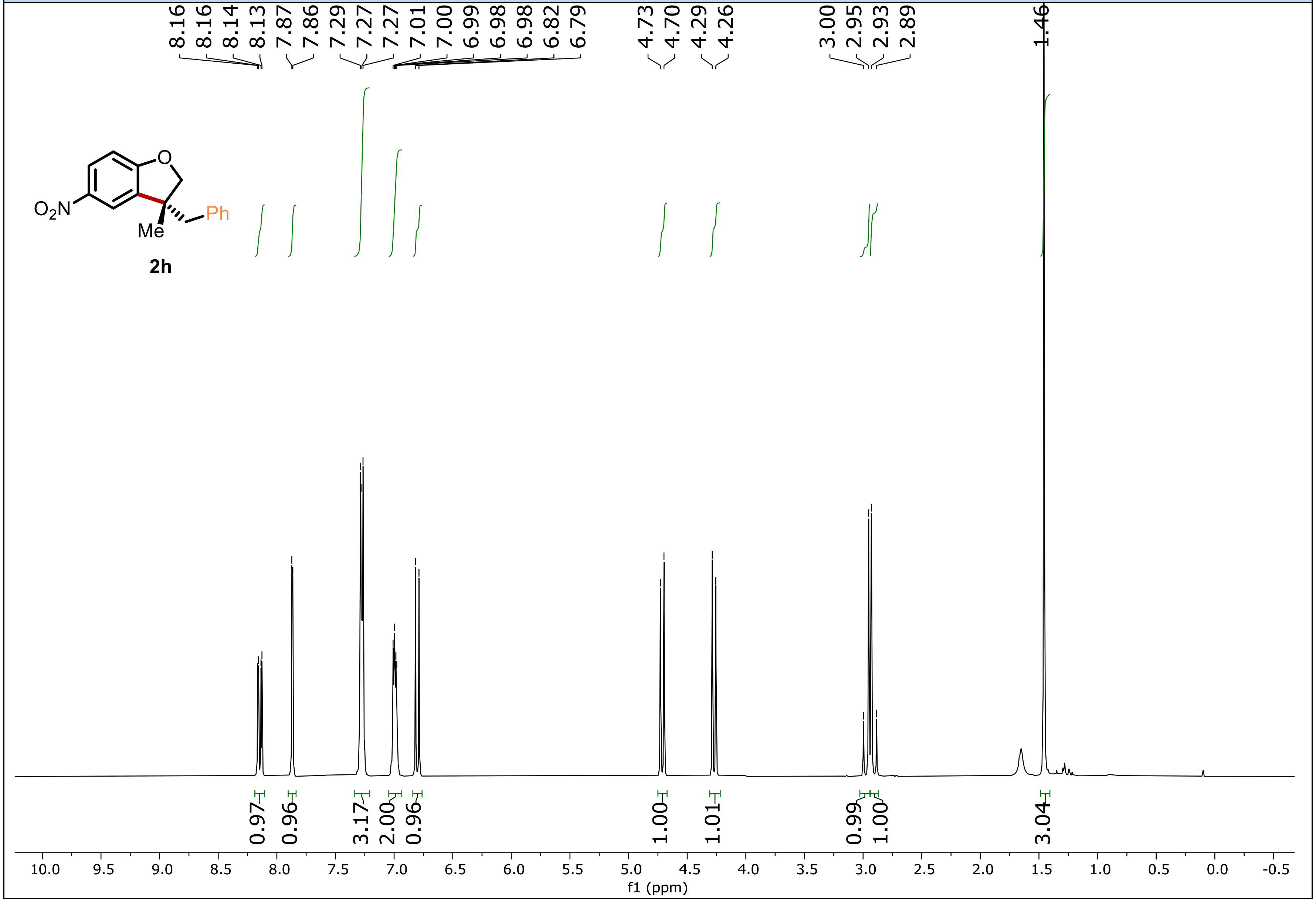

2h – $^{13}C\{^{1}H\}$ NMR (75 MHz, $CDCl_3$)

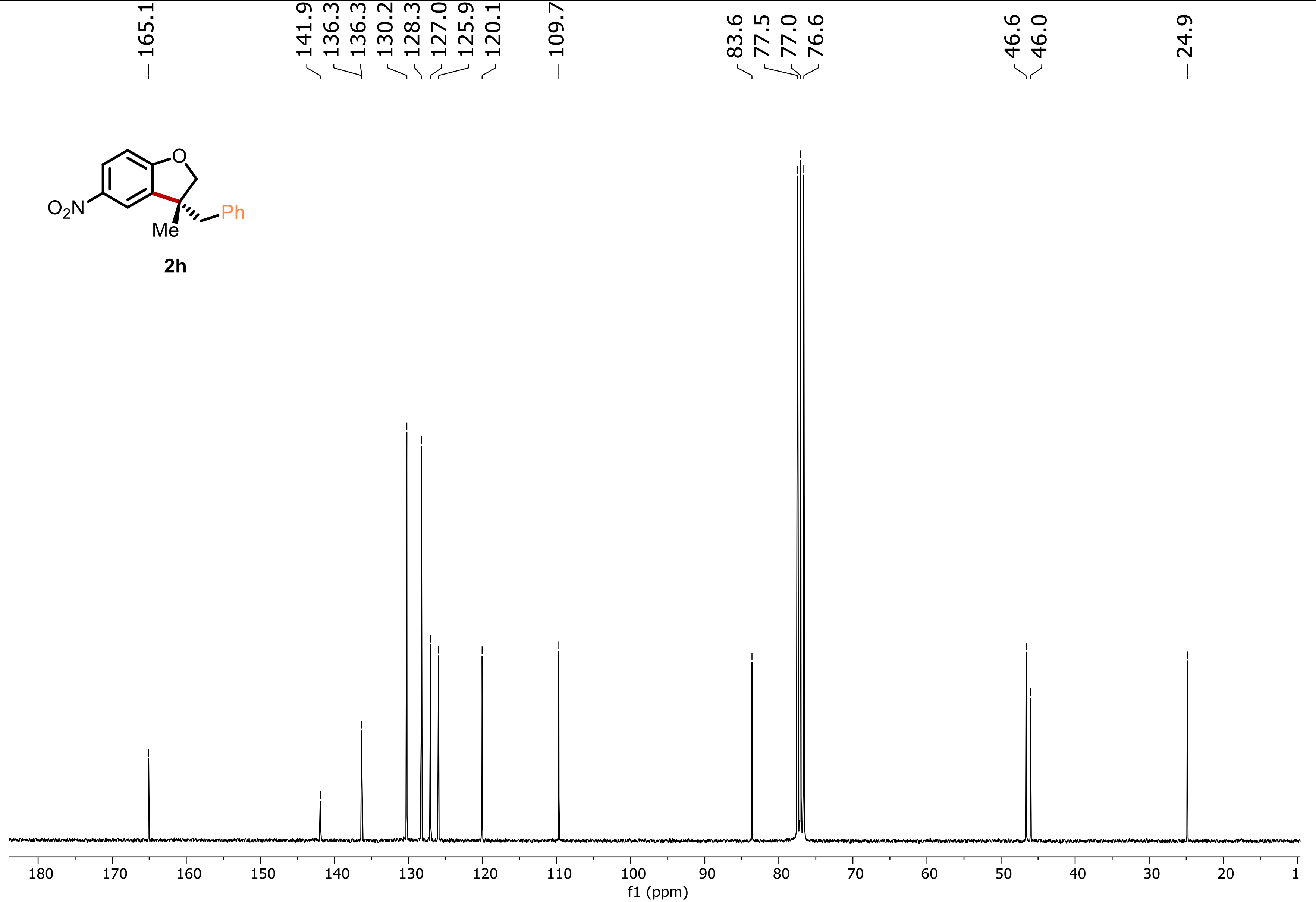

7. Chromatograms

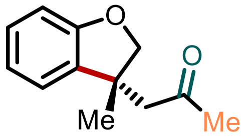

1a

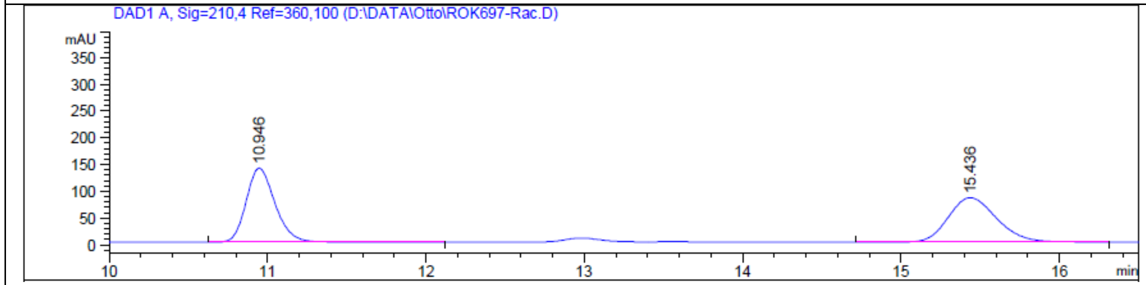

Signal 1: DAD1 A, Sig=210,4 Ref=360,100

Peak #	RetTime [min]	Type	Width [min]	Area [mAU*s]	Height [mAU]	Area %
1	10.946	BB	0.1962	1762.13074	138.75732	50.0890
2	15.436	BB	0.3284	1755.86584	83.15380	49.9110
Totals :				3517.99658	221.91113	

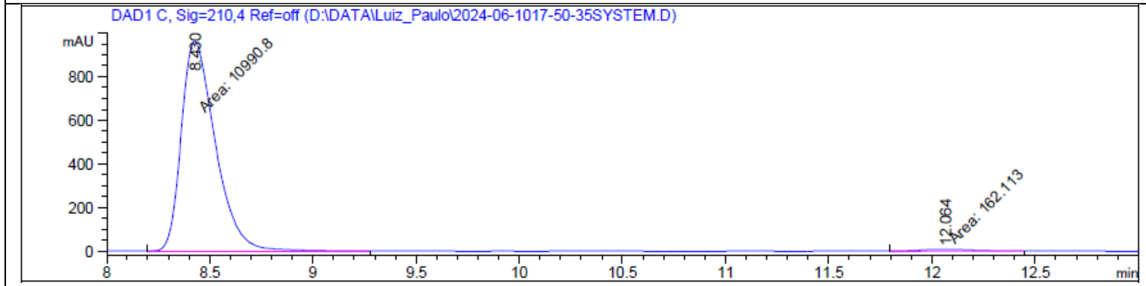

Signal 3: DAD1 C, Sig=210,4 Ref=off

Peak #	RetTime [min]	Type	Width [min]	Area [mAU*s]	Height [mAU]	Area %
1	8.430	MM	0.1904	1.09908e4	961.91425	98.5465
2	12.064	MM	0.3058	162.11253	8.83561	1.4535
Totals :				1.11529e4	970.74985	

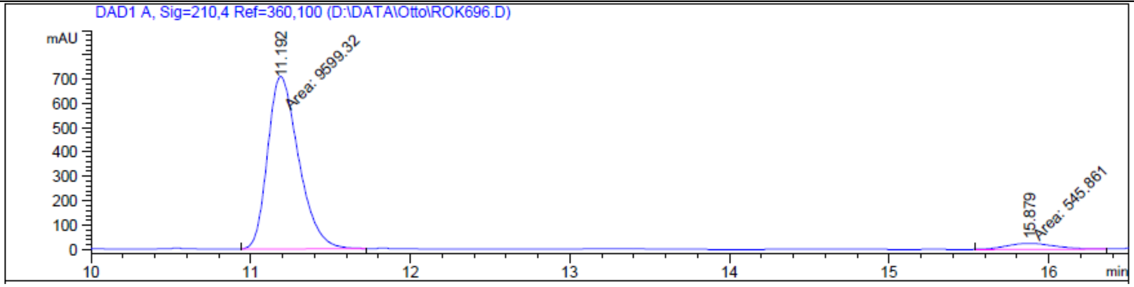

Signal 1: DAD1 A, Sig=210,4 Ref=360,100

Peak #	RetTime [min]	Type	Width [min]	Area [mAU*s]	Height [mAU]	Area %
1	11.192	MM	0.2259	9599.32031	708.23370	94.6195
2	15.879	MM	0.3587	545.86133	25.35944	5.3805
Totals :				1.01452e4	733.59314	

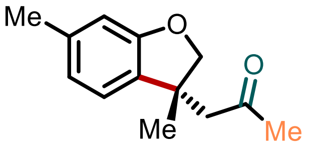

1b

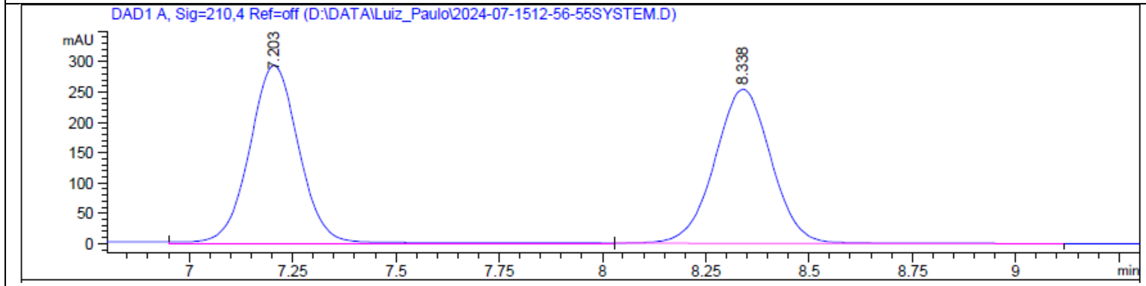

Signal 1: DAD1 A, Sig=210,4 Ref=off

Peak #	RetTime [min]	Type	Width [min]	Area [mAU*s]	Height [mAU]	Area %
1	7.203	VB	0.1285	2426.59106	293.34821	50.2520
2	8.338	BB	0.1485	2402.25806	253.53281	49.7480
Totals :				4828.84912	546.88101	

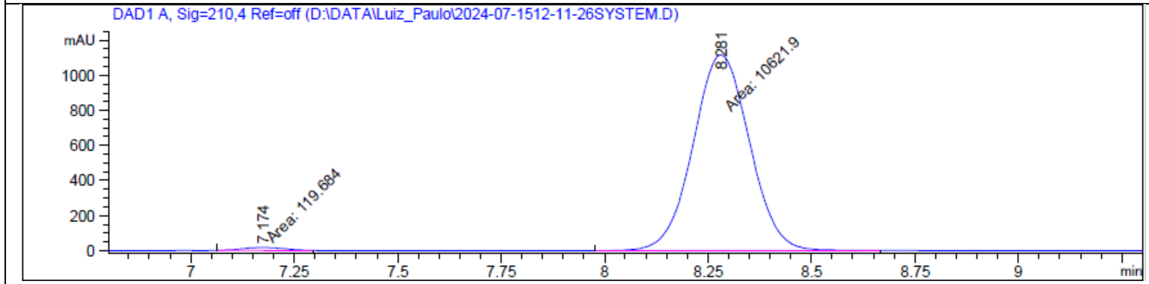

Signal 1: DAD1 A, Sig=210,4 Ref=off

Peak #	RetTime [min]	Type	Width [min]	Area [mAU*s]	Height [mAU]	Area %
1	7.174	MM	0.1179	119.68419	16.91997	1.1142
2	8.281	MM	0.1584	1.06219e4	1117.55994	98.8858
Totals :				1.07416e4	1134.47991	

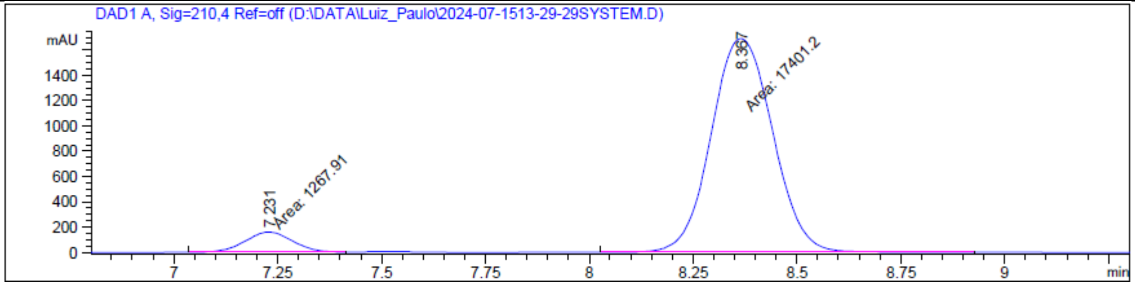

Signal 1: DAD1 A, Sig=210,4 Ref=off

Peak #	RetTime [min]	Type	Width [min]	Area [mAU*s]	Height [mAU]	Area %
1	7.231	MM	0.1329	1267.91248	159.02509	6.7915
2	8.367	MM	0.1714	1.74012e4	1692.11316	93.2085
Totals :				1.86691e4	1851.13824	

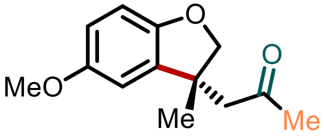

1c

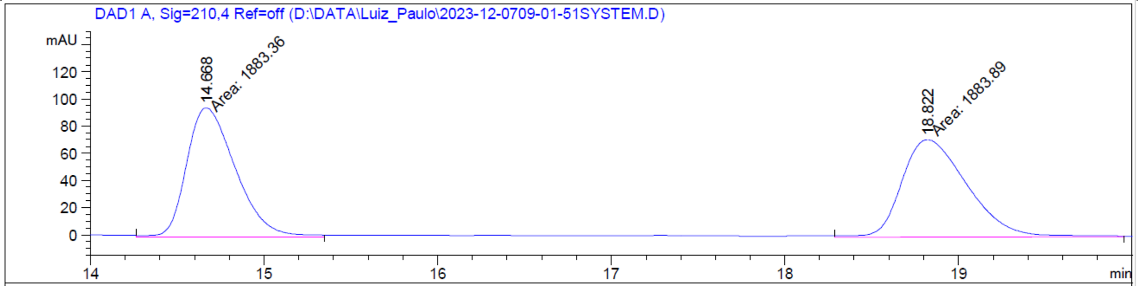

Signal 1: DAD1 A, Sig=210,4 Ref=off

Peak #	RetTime [min]	Type	Width [min]	Area [mAU*s]	Height [mAU]	Area %
1	14.668	MM	0.3289	1883.36047	95.43782	49.9930
2	18.822	MM	0.4373	1883.89087	71.79560	50.0070
Totals :				3767.25134	167.23342	

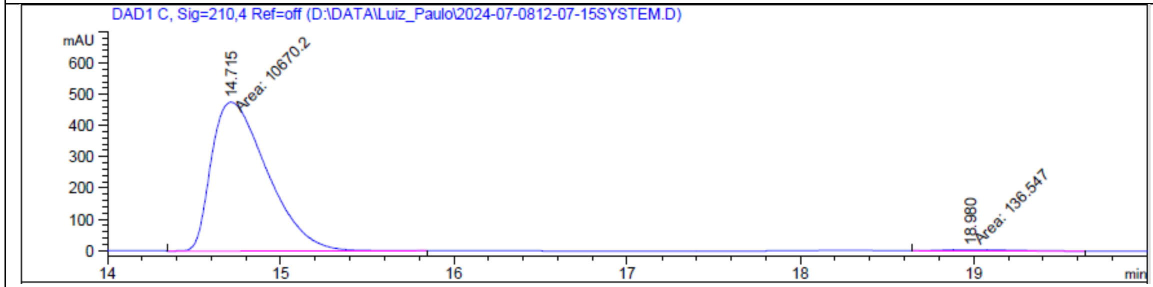

Signal 3: DAD1 C, Sig=210,4 Ref=off

Peak #	RetTime [min]	Type	Width [min]	Area [mAU*s]	Height [mAU]	Area %
1	14.715	MM	0.3733	1.06702e4	476.38248	98.7365
2	18.980	MM	0.4336	136.54720	5.24896	1.2635
Totals :				1.08067e4	481.63143	

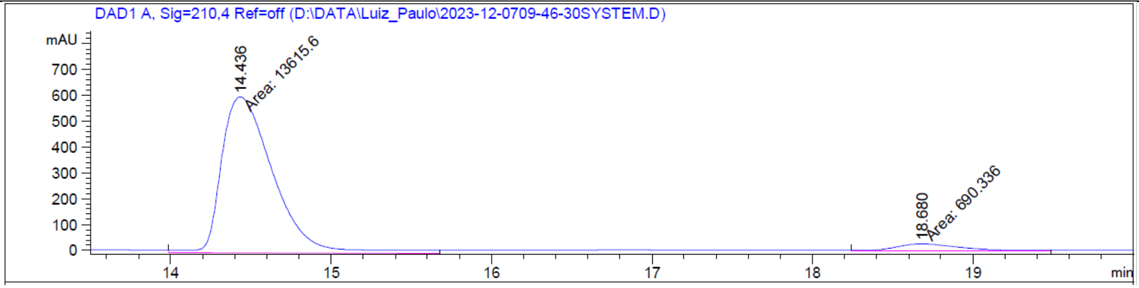

Signal 1: DAD1 A, Sig=210,4 Ref=off

Peak #	RetTime [min]	Type	Width [min]	Area [mAU*s]	Height [mAU]	Area %
1	14.436	MM	0.3762	1.36156e4	603.13104	95.1745
2	18.680	MM	0.4514	690.33588	25.49120	4.8255
Totals :				1.43059e4	628.62224	

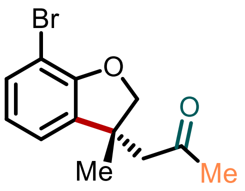

1d

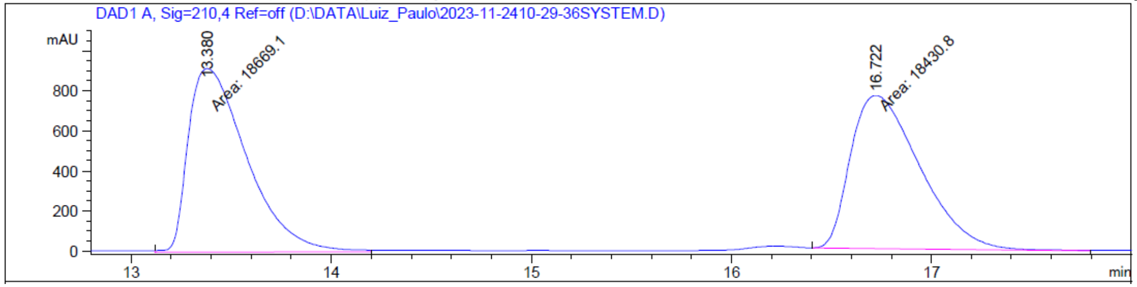

Signal 1: DAD1 A, Sig=210,4 Ref=off

Peak #	RetTime [min]	Type	Width [min]	Area [mAU*s]	Height [mAU]	Area %
1	13.380	MM	0.3393	1.86691e4	917.04169	50.3212
2	16.722	MM	0.4010	1.84308e4	766.04987	49.6788
Totals :				3.70999e4	1683.09155	

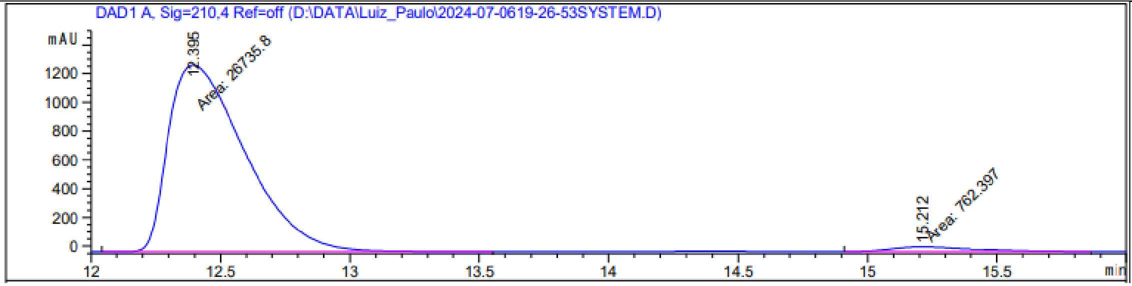

Signal 1: DAD1 A, Sig=210,4 Ref=off

Peak #	RetTime [min]	Type	Width [min]	Area [mAU*s]	Height [mAU]	Area %
1	12.395	MM	0.3431	2.67358e4	1298.78406	97.2275
2	15.212	MM	0.3718	762.39667	34.17860	2.7725
Totals :				2.74982e4	1332.96266	

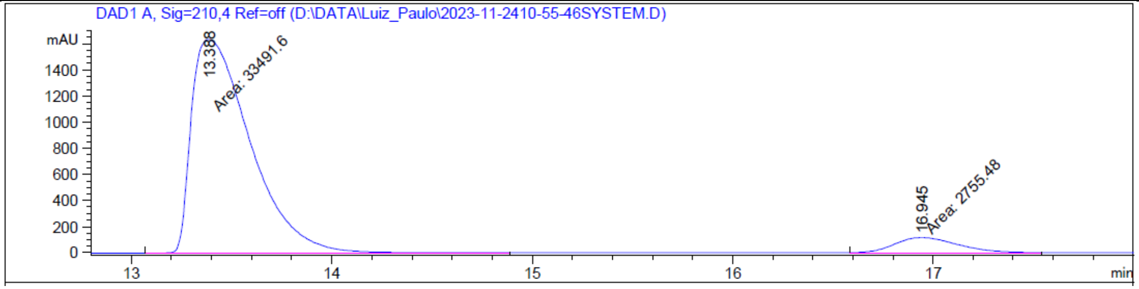

Signal 1: DAD1 A, Sig=210,4 Ref=off

Peak #	RetTime [min]	Type	Width [min]	Area [mAU*s]	Height [mAU]	Area %
1	13.388	MM	0.3411	3.34916e4	1636.47351	92.3981
2	16.945	MM	0.3829	2755.47876	119.94405	7.6019
Totals :				3.62471e4	1756.41756	

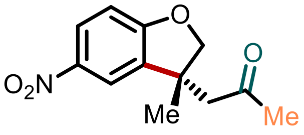

1e

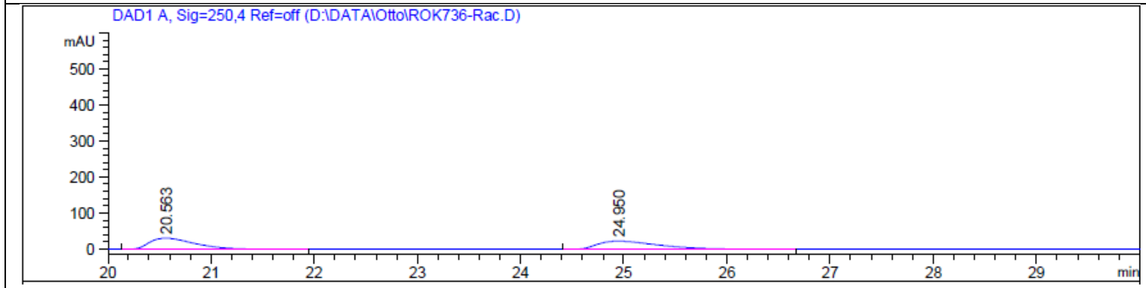

Signal 3: DAD1 C, Sig=210,4 Ref=off

Peak #	RetTime [min]	Type	Width [min]	Area [mAU*s]	Height [mAU]	Area %
1	20.563	BB	0.4753	4288.55225	140.01329	49.9430
2	24.951	BB	0.6278	4298.34082	104.08773	50.0570
Totals :				8586.89307	244.10102	

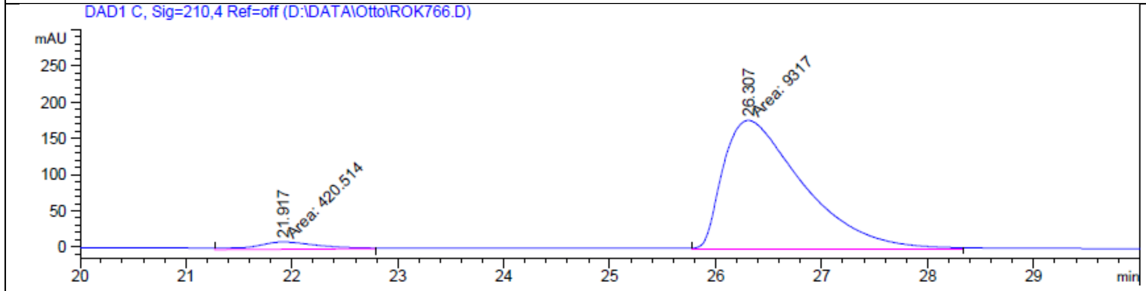

Signal 3: DAD1 C, Sig=210,4 Ref=off

Peak #	RetTime [min]	Type	Width [min]	Area [mAU*s]	Height [mAU]	Area %
1	21.917	MM	0.6941	420.51373	10.09689	4.3185
2	26.307	MM	0.8737	9317.00098	177.72514	95.6815
Totals :				9737.51471	187.82204	

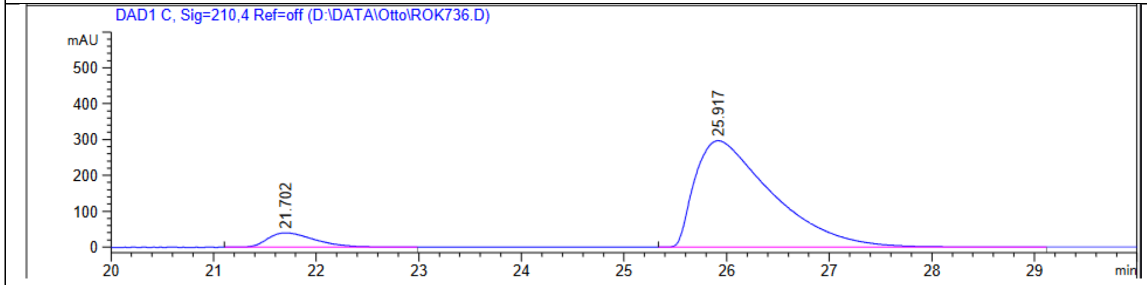

Signal 3: DAD1 C, Sig=210,4 Ref=off

Peak #	RetTime [min]	Type	Width [min]	Area [mAU*s]	Height [mAU]	Area %
1	21.702	BB	0.4981	1288.87695	40.19273	7.6450
2	25.917	BB	0.7813	1.55703e4	297.16141	92.3550
Totals :				1.68591e4	337.35414	

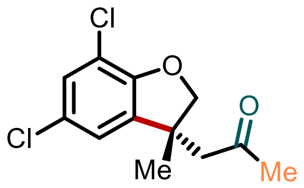

1f

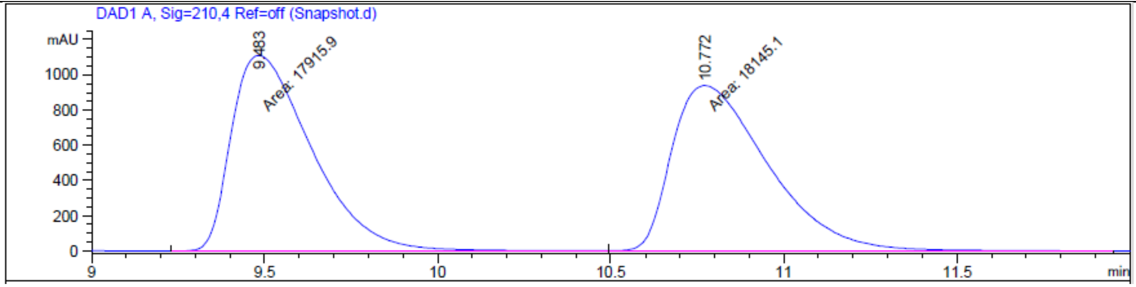

Signal 1: DAD1 A, Sig=210,4 Ref=off

Peak #	RetTime [min]	Type	Width [min]	Area [mAU*s]	Height [mAU]	Area %
1	9.483	MM	0.2687	1.79159e4	1111.06909	49.6821
2	10.772	MM	0.3223	1.81451e4	938.20557	50.3179

Totals : 3.60610e4 2049.27466

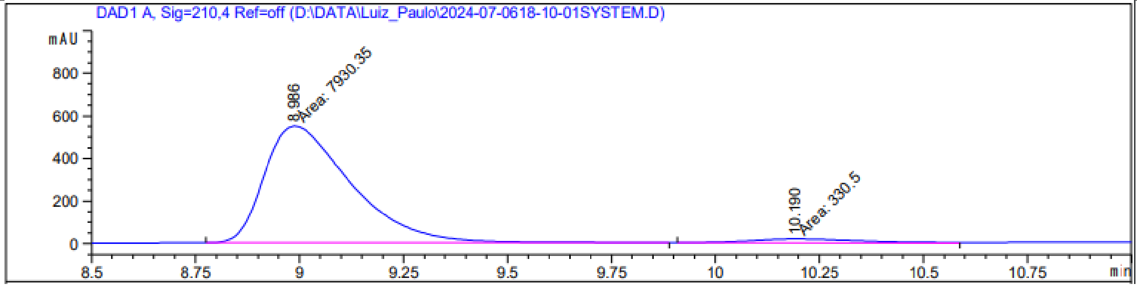

Signal 1: DAD1 A, Sig=210,4 Ref=off

Peak #	RetTime [min]	Type	Width [min]	Area [mAU*s]	Height [mAU]	Area %
1	8.986	MM	0.2413	7930.34521	547.67535	95.9992
2	10.190	MM	0.3053	330.49991	18.04042	4.0008

Totals : 8260.84512 565.71578

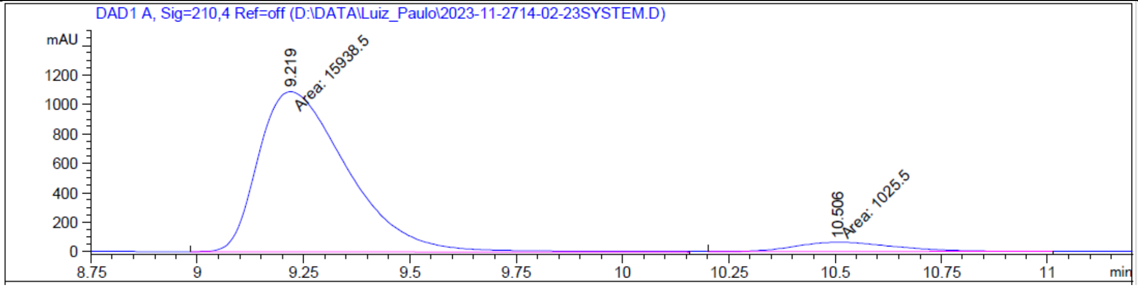

Signal 1: DAD1 A, Sig=210,4 Ref=off

Peak #	RetTime [min]	Type	Width [min]	Area [mAU*s]	Height [mAU]	Area %
1	9.219	MM	0.2438	1.59385e4	1089.37964	93.9548
2	10.506	MM	0.2721	1025.50195	62.81746	6.0452

Totals : 1.69640e4 1152.19710

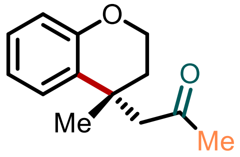

1g

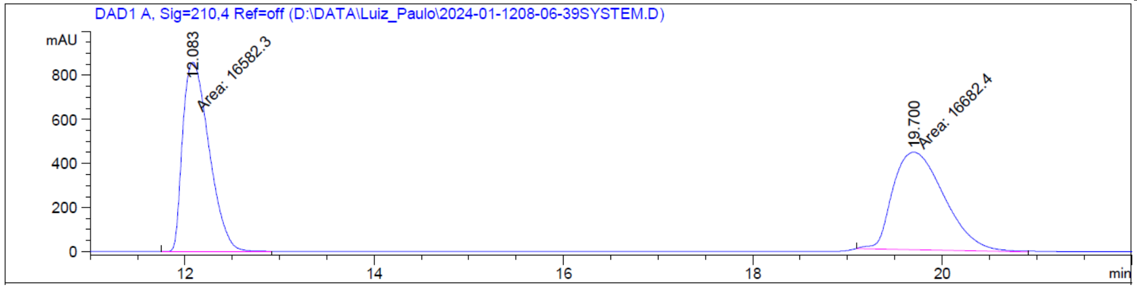

Signal 1: DAD1 A, Sig=210,4 Ref=off

Peak #	RetTime [min]	Type	Width [min]	Area [mAU*s]	Height [mAU]	Area %
1	12.083	MM	0.3222	1.65823e4	857.80029	49.8496
2	19.700	MM	0.6294	1.66824e4	441.76773	50.1504

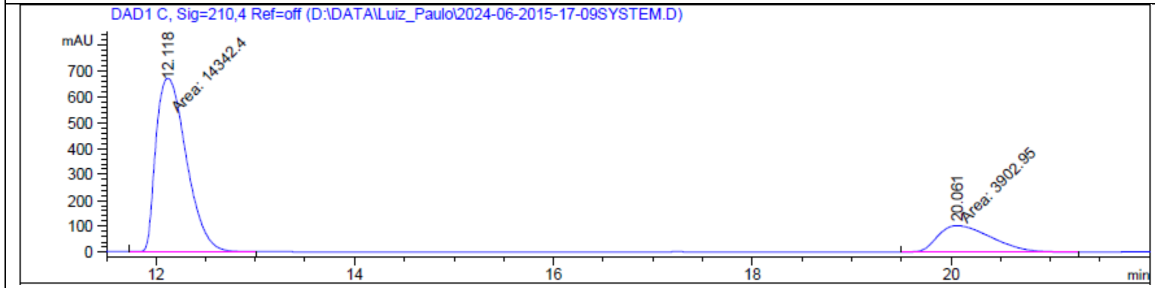

Signal 3: DAD1 C, Sig=210,4 Ref=off

Peak #	RetTime [min]	Type	Width [min]	Area [mAU*s]	Height [mAU]	Area %
1	12.118	MM	0.3564	1.43424e4	670.73254	78.6085
2	20.061	MM	0.6317	3902.94653	102.97867	21.3915
Totals :				1.82453e4	773.71121	

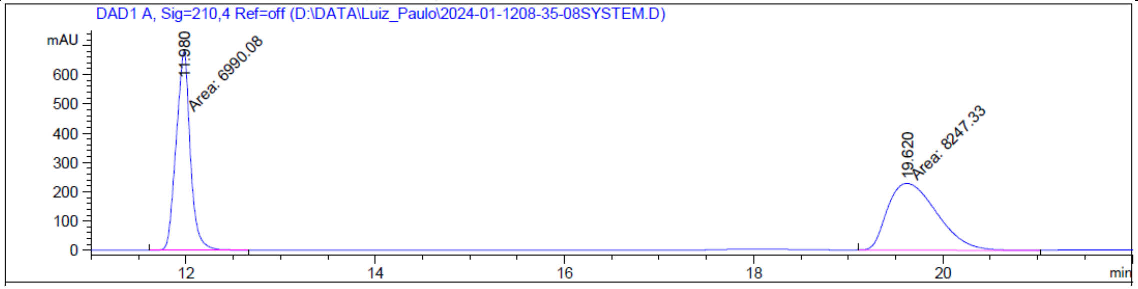

Signal 1: DAD1 A, Sig=210,4 Ref=off

Peak #	RetTime [min]	Type	Width [min]	Area [mAU*s]	Height [mAU]	Area %
1	11.980	MM	0.1717	6990.07861	678.56592	45.8745
2	19.620	MM	0.6036	8247.32520	227.73166	54.1255
Totals :				1.52374e4	906.29758	

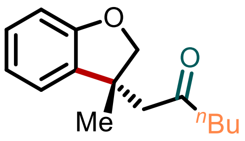

1h

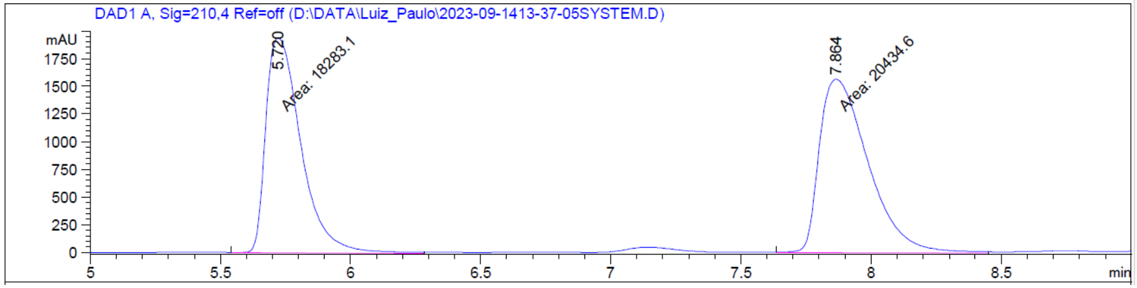

Signal 1: DAD1 A, Sig=210,4 Ref=off

Peak #	RetTime [min]	Type	Width [min]	Area [mAU*s]	Height [mAU]	Area %
1	5.720	MM	0.1570	1.82831e4	1940.61829	47.2215
2	7.864	MM	0.2175	2.04346e4	1565.85999	52.7785

Totals : 3.87177e4 3506.47827

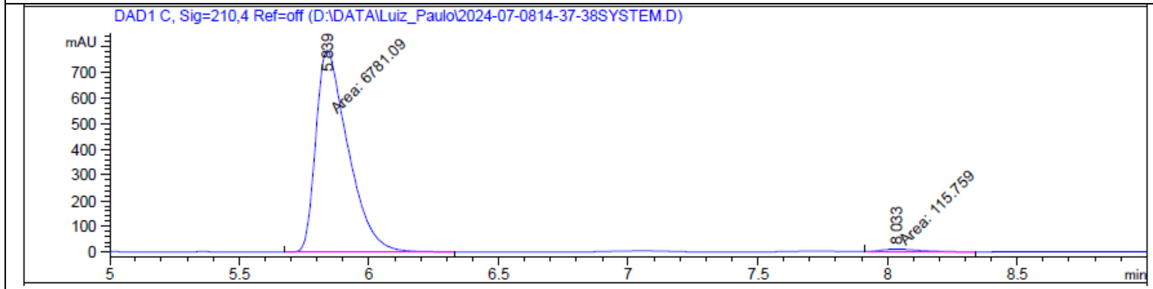

Signal 3: DAD1 C, Sig=210,4 Ref=off

Peak #	RetTime [min]	Type	Width [min]	Area [mAU*s]	Height [mAU]	Area %
1	5.839	MM	0.1440	6781.08594	784.89935	98.3216
2	8.033	PM	0.1836	115.75871	10.50649	1.6784

Totals : 6896.84465 795.40585

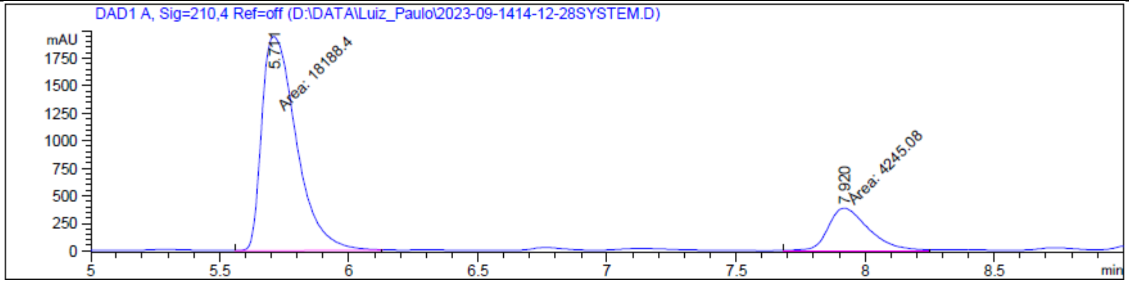

Signal 1: DAD1 A, Sig=210,4 Ref=off

Peak #	RetTime [min]	Type	Width [min]	Area [mAU*s]	Height [mAU]	Area %
1	5.711	MM	0.1557	1.81884e4	1947.03064	81.0771
2	7.920	MM	0.1826	4245.07715	387.55377	18.9229

Totals : 2.24335e4 2334.58441

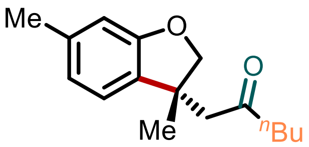

1i

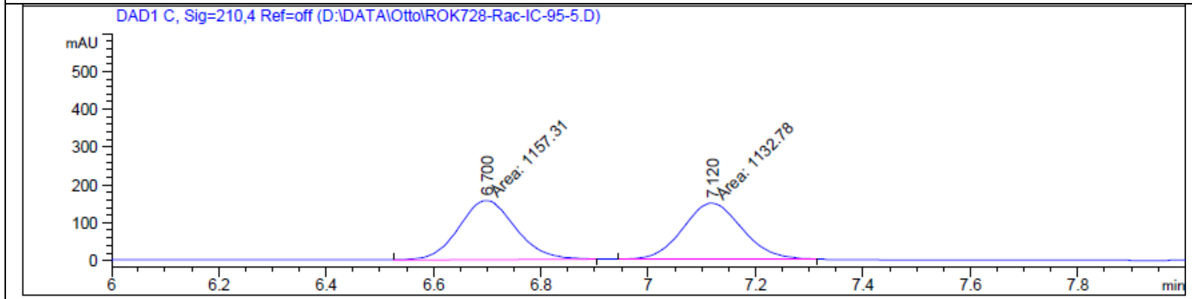

Signal 3: DAD1 C, Sig=210,4 Ref=off

Peak #	RetTime [min]	Type	Width [min]	Area [mAU*s]	Height [mAU]	Area %
1	6.700	MM	0.1228	1157.31274	157.10641	50.5356
2	7.120	MM	0.1274	1132.78015	148.18495	49.4644

Totals : 2290.09290 305.29137

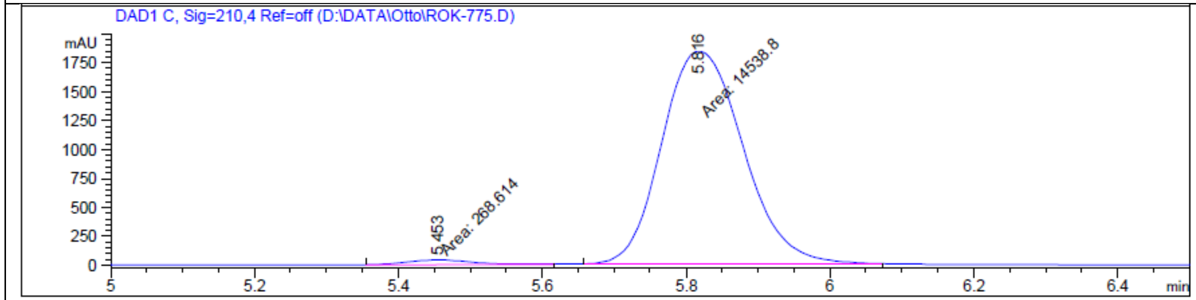

Signal 3: DAD1 C, Sig=210,4 Ref=off

Peak #	RetTime [min]	Type	Width [min]	Area [mAU*s]	Height [mAU]	Area %
1	5.453	MM	0.1074	268.61389	41.68488	1.8140
2	5.816	MM	0.1314	1.45388e4	1844.28784	98.1860

Totals : 1.48075e4 1885.97272

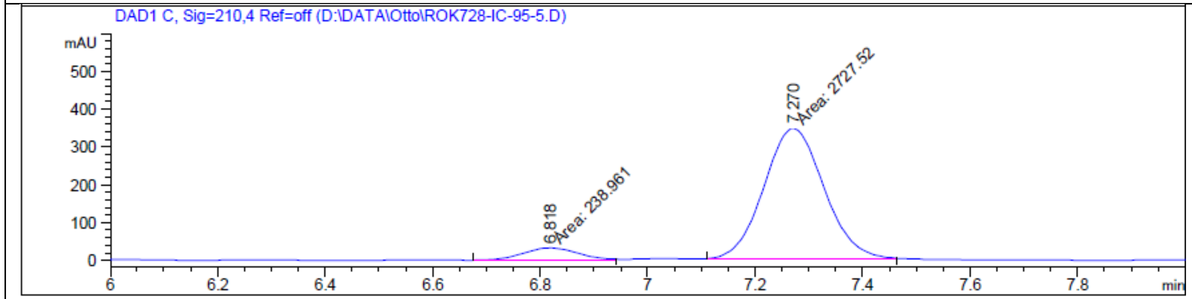

Signal 3: DAD1 C, Sig=210,4 Ref=off

Peak #	RetTime [min]	Type	Width [min]	Area [mAU*s]	Height [mAU]	Area %
1	6.818	MM	0.1204	238.96072	33.07135	8.0554
2	7.270	MM	0.1308	2727.52075	347.65637	91.9446

Totals : 2966.48148 380.72772

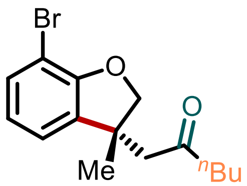

1j

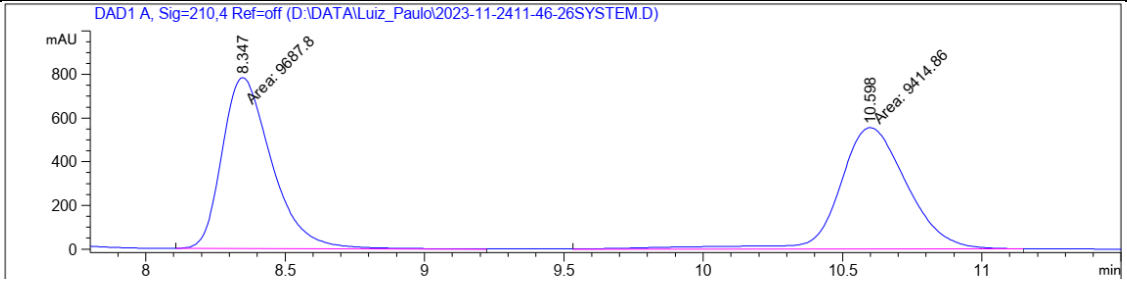

Signal 1: DAD1 A, Sig=210,4 Ref=off

Peak #	RetTime [min]	Type	Width [min]	Area [mAU*s]	Height [mAU]	Area %
1	8.347	MM	0.2060	9687.79688	783.95172	50.7144
2	10.598	MM	0.2823	9414.85840	555.89746	49.2856

Totals : 1.91027e4 1339.84918

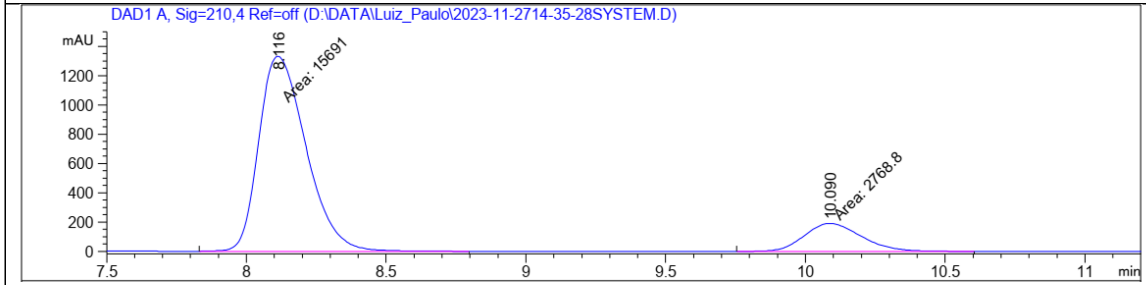

Signal 1: DAD1 A, Sig=210,4 Ref=off

Peak #	RetTime [min]	Type	Width [min]	Area [mAU*s]	Height [mAU]	Area %
1	8.116	MM	0.1964	1.56910e4	1331.69202	85.0009
2	10.090	MM	0.2409	2768.79883	191.55125	14.9991

Totals : 1.84598e4 1523.24327

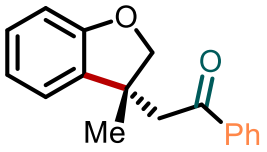

1k

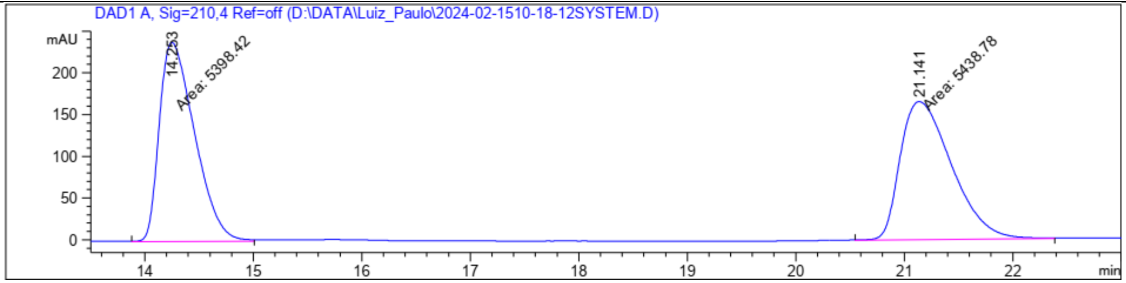

Signal 1: DAD1 A, Sig=210,4 Ref=off

Peak #	RetTime [min]	Type	Width [min]	Area [mAU*s]	Height [mAU]	Area %
1	14.253	MM	0.3772	5398.41895	238.53273	49.8138
2	21.141	MM	0.5483	5438.77637	165.31741	50.1862

Totals : 1.08372e4 403.85014

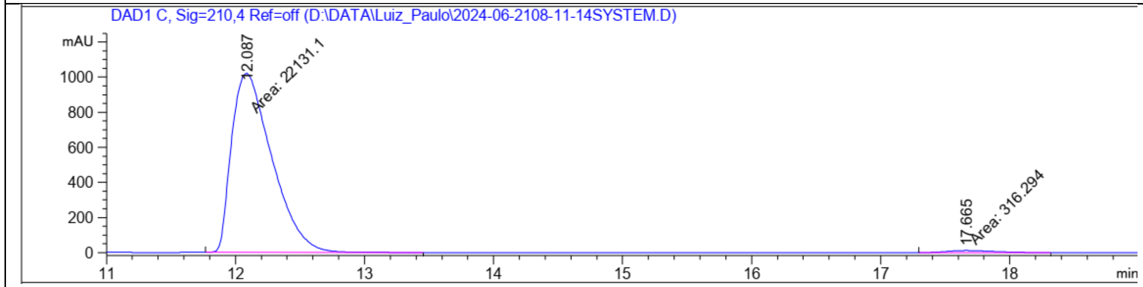

Signal 3: DAD1 C, Sig=210,4 Ref=off

Peak #	RetTime [min]	Type	Width [min]	Area [mAU*s]	Height [mAU]	Area %
1	12.087	MM	0.3615	2.21311e4	1020.20380	98.5910
2	17.665	PM	0.4333	316.29404	12.16583	1.4090

Totals : 2.24474e4 1032.36962

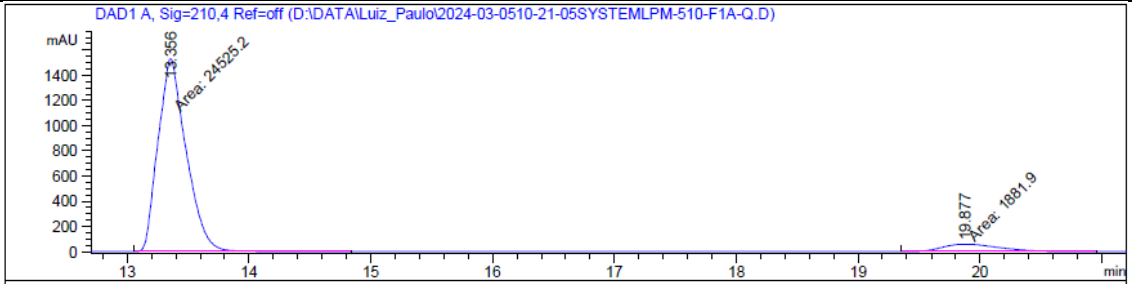

Signal 1: DAD1 A, Sig=210,4 Ref=off

Peak #	RetTime [min]	Type	Width [min]	Area [mAU*s]	Height [mAU]	Area %
1	13.356	MM	0.2653	2.43426e4	1529.09680	92.9226
2	19.879	MM	0.5296	1854.03394	58.34413	7.0774

Totals : 2.61967e4 1587.44093

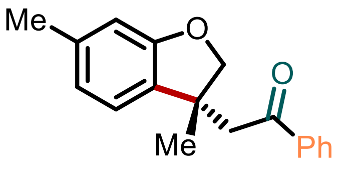

1l

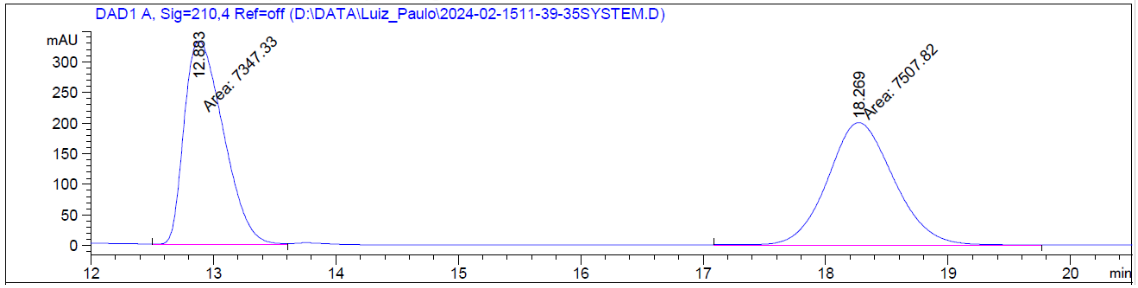

Signal 1: DAD1 A, Sig=210,4 Ref=off

Peak #	RetTime [min]	Type	Width [min]	Area [mAU*s]	Height [mAU]	Area %
1	12.883	MM	0.3692	7347.32568	331.64206	49.4598
2	18.269	MM	0.6261	7507.81689	199.84761	50.5402

Totals : 1.48551e4 531.48967

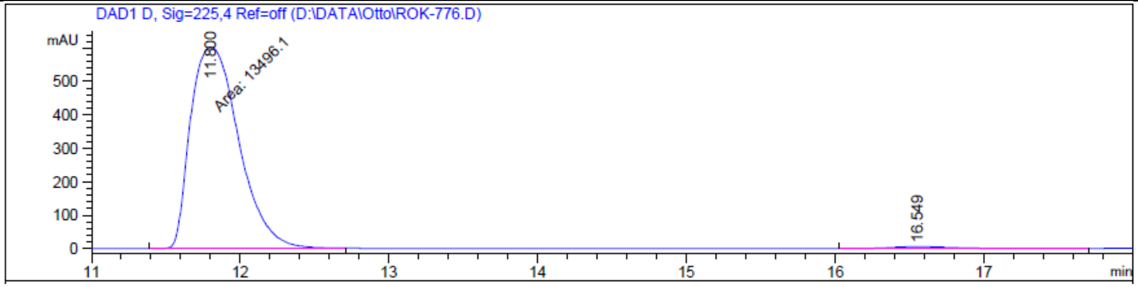

Signal 4: DAD1 D, Sig=225,4 Ref=off

Peak #	RetTime [min]	Type	Width [min]	Area [mAU*s]	Height [mAU]	Area %
1	11.800	MM	0.3742	1.34961e4	601.18738	98.8451
2	16.549	BB	0.4046	157.68118	5.77319	1.1549

Totals : 1.36538e4 606.96057

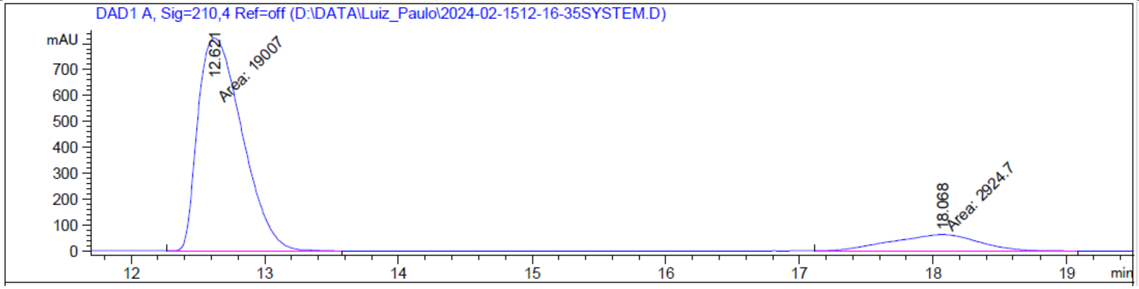

Signal 1: DAD1 A, Sig=210,4 Ref=off

Peak #	RetTime [min]	Type	Width [min]	Area [mAU*s]	Height [mAU]	Area %
1	12.621	MM	0.3850	1.90070e4	822.88593	86.6645
2	18.068	MM	0.7681	2924.70361	63.45905	13.3355

Totals : 2.19317e4 886.34498

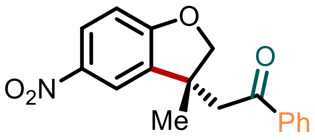

1m

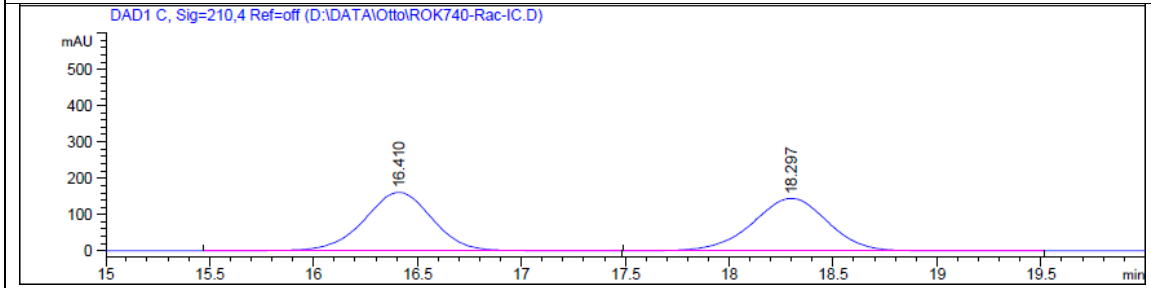

Signal 3: DAD1 C, Sig=210,4 Ref=off

Peak #	RetTime [min]	Type	Width [min]	Area [mAU*s]	Height [mAU]	Area %
1	16.410	BB	0.3516	3665.95044	161.05730	50.0414
2	18.297	BB	0.3870	3659.88550	144.75261	49.9586

Totals : 7325.83594 305.80991

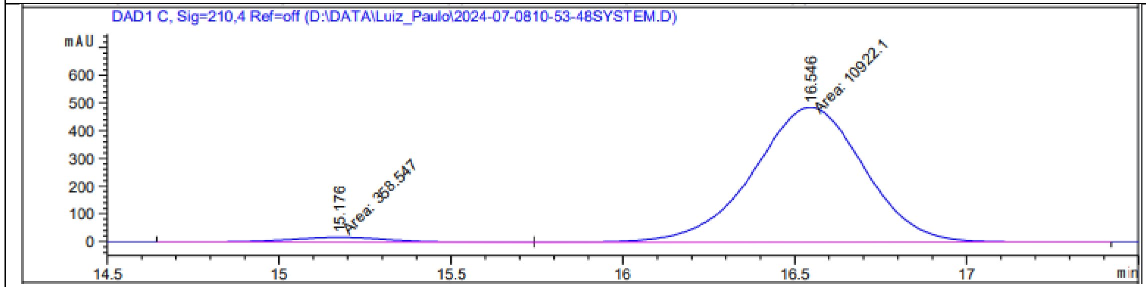

Signal 3: DAD1 C, Sig=210,4 Ref=off

Peak #	RetTime [min]	Type	Width [min]	Area [mAU*s]	Height [mAU]	Area %
1	15.176	MM	0.3409	358.54703	17.52746	3.1784
2	16.546	MM	0.3739	1.09221e4	486.84634	96.8216

Totals : 1.12806e4 504.37381

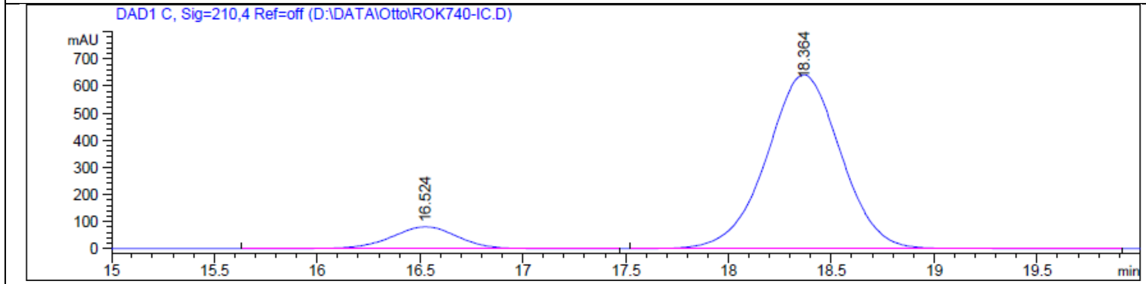

Signal 3: DAD1 C, Sig=210,4 Ref=off

Peak #	RetTime [min]	Type	Width [min]	Area [mAU*s]	Height [mAU]	Area %
1	16.524	BB	0.3467	1818.52478	80.74820	10.1641
2	18.364	BB	0.3845	1.60732e4	641.11914	89.8359

Totals : 1.78917e4 721.86734

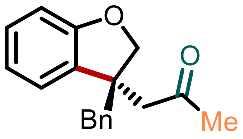

1n

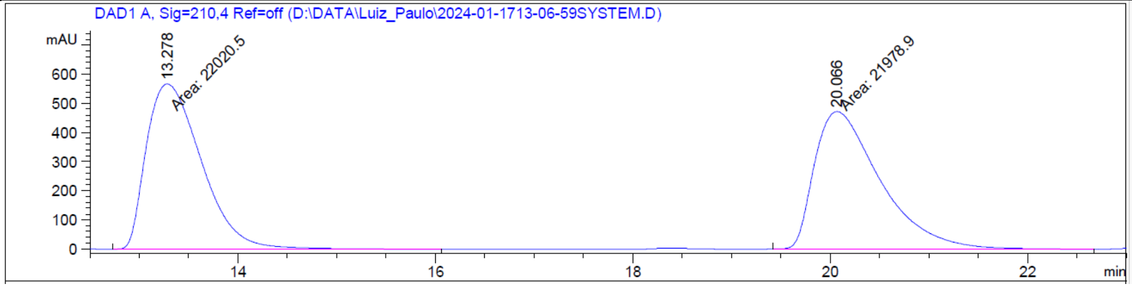

Signal 1: DAD1 A, Sig=210,4 Ref=off

Peak #	RetTime [min]	Type	Width [min]	Area [mAU*s]	Height [mAU]	Area %
1	13.278	MM	0.6467	2.20205e4	567.49133	50.0474
2	20.066	MM	0.7752	2.19789e4	472.56564	49.9526
Totals :				4.39994e4	1040.05698	

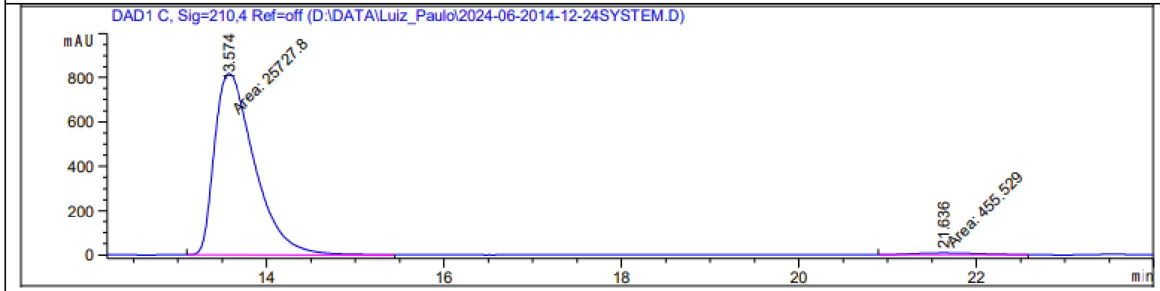

Signal 3: DAD1 C, Sig=210,4 Ref=off

Peak #	RetTime [min]	Type	Width [min]	Area [mAU*s]	Height [mAU]	Area %
1	13.574	MM	0.5231	2.57278e4	819.65802	98.2602
2	21.636	MM	0.8900	455.52896	8.53085	1.7398
Totals :				2.61833e4	828.18887	

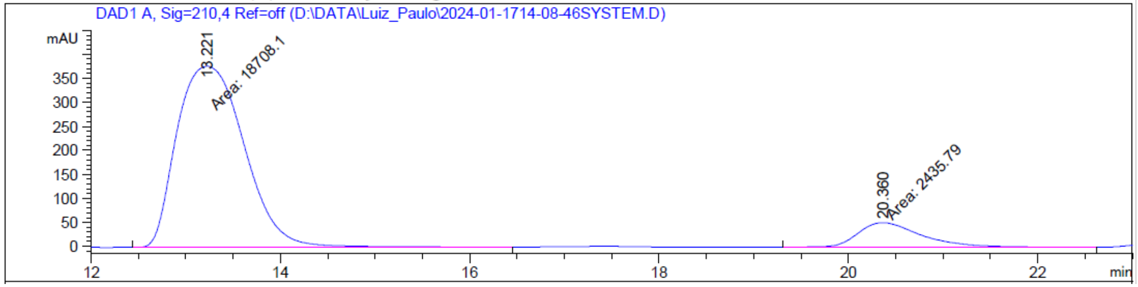

Signal 1: DAD1 A, Sig=210,4 Ref=off

Peak #	RetTime [min]	Type	Width [min]	Area [mAU*s]	Height [mAU]	Area %
1	13.221	MM	0.8268	1.87081e4	377.12115	88.4799
2	20.360	MM	0.7912	2435.79370	51.30882	11.5201
Totals :				2.11438e4	428.42998	

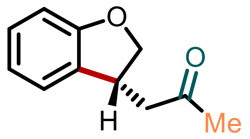

1o

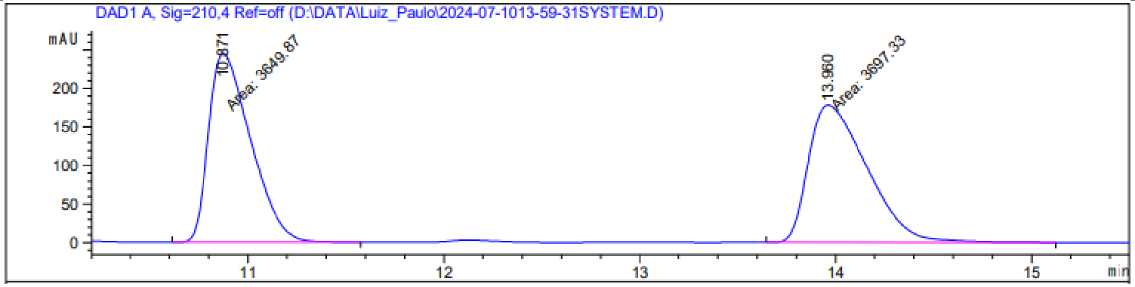

Signal 1: DAD1 A, Sig=210,4 Ref=off

Peak #	RetTime [min]	Type	Width [min]	Area [mAU*s]	Height [mAU]	Area %
1	10.871	MM	0.2480	3649.87451	245.27097	49.6770
2	13.960	MP	0.3463	3697.33179	177.94182	50.3230

Totals : 7347.20630 423.21278

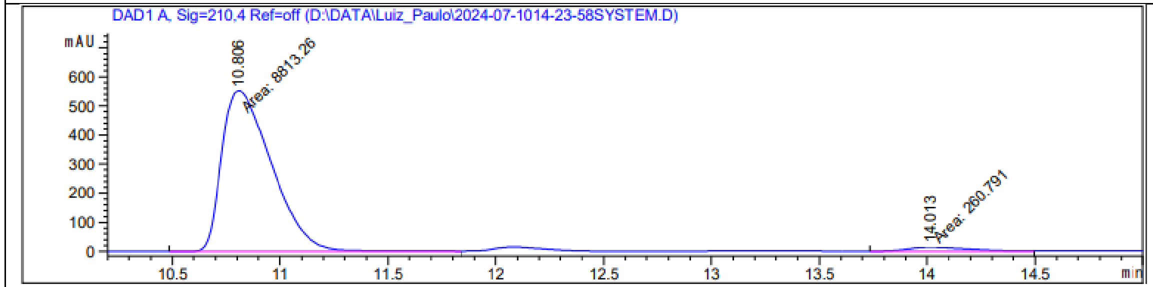

Signal 1: DAD1 A, Sig=210,4 Ref=off

Peak #	RetTime [min]	Type	Width [min]	Area [mAU*s]	Height [mAU]	Area %
1	10.806	MM	0.2660	8813.25977	552.19781	97.1260
2	14.013	MM	0.3150	260.79053	13.79644	2.8740

Totals : 9074.05029 565.99425

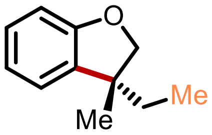

2a

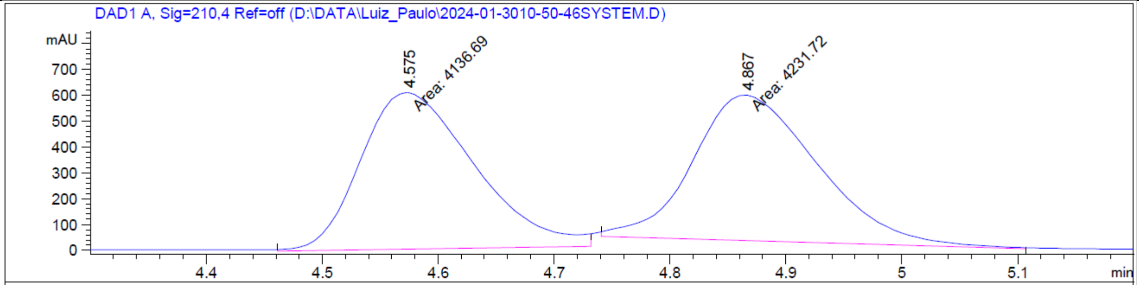

Signal 1: DAD1 A, Sig=210,4 Ref=off

Peak #	RetTime [min]	Type	Width [min]	Area [mAU*s]	Height [mAU]	Area %
1	4.575	MM	0.1135	4136.69141	607.44312	49.4322
2	4.867	MM	0.1252	4231.72363	563.22906	50.5678

Totals : 8368.41504 1170.67218

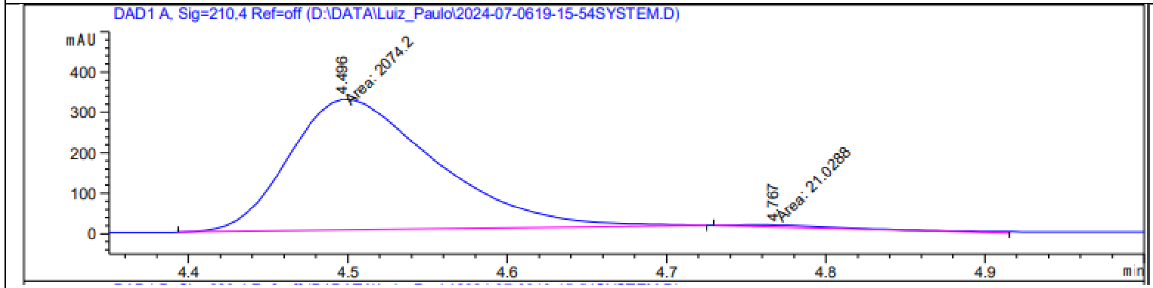

Signal 1: DAD1 A, Sig=210,4 Ref=off

Peak #	RetTime [min]	Type	Width [min]	Area [mAU*s]	Height [mAU]	Area %
1	4.496	PM	0.1064	2074.20288	324.96143	98.9963
2	4.767	MM	0.0823	21.02885	4.25794	1.0037

Totals : 2095.23173 329.21937

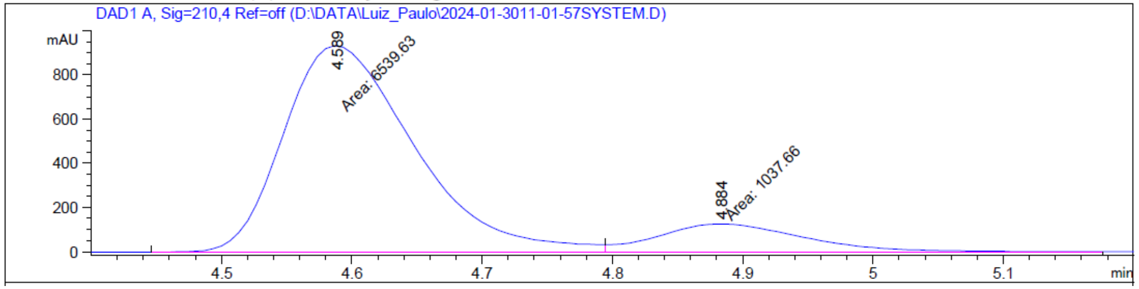

Signal 1: DAD1 A, Sig=210,4 Ref=off

Peak #	RetTime [min]	Type	Width [min]	Area [mAU*s]	Height [mAU]	Area %
1	4.589	MF	0.1162	6539.63330	938.27051	86.3056
2	4.884	FM	0.1336	1037.66309	129.44357	13.6944

Totals : 7577.29639 1067.71408

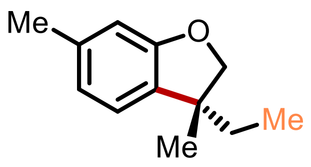

2b

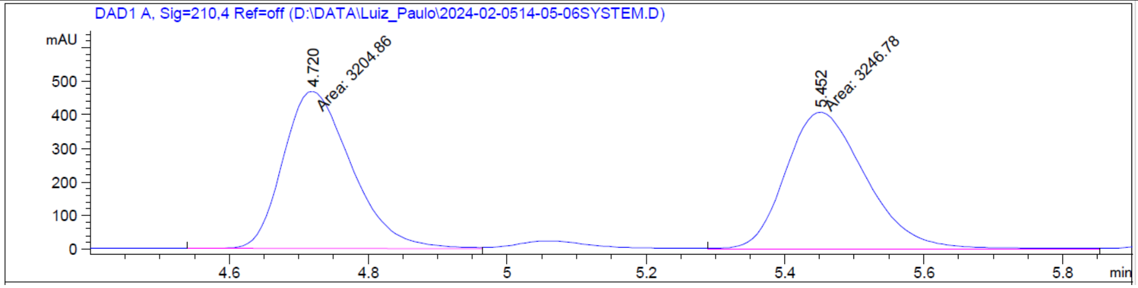

Signal 1: DAD1 A, Sig=210,4 Ref=off

Peak #	RetTime [min]	Type	Width [min]	Area [mAU*s]	Height [mAU]	Area %
1	4.720	MM	0.1139	3204.86108	468.78891	49.6751
2	5.452	MM	0.1331	3246.78296	406.55450	50.3249
Totals :				6451.64404	875.34341	

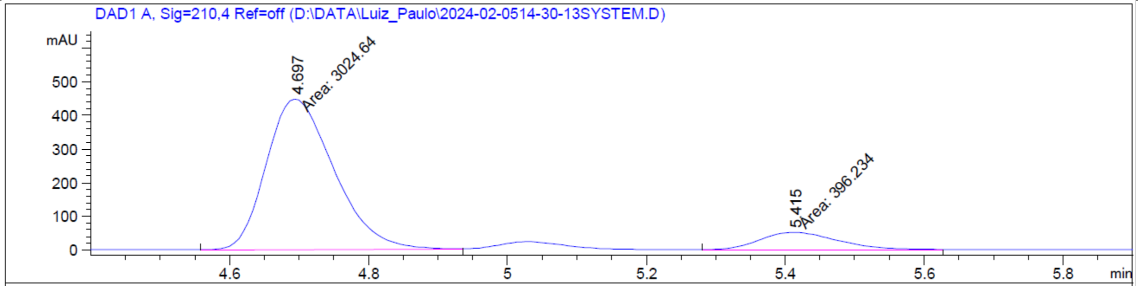

Signal 1: DAD1 A, Sig=210,4 Ref=off

Peak #	RetTime [min]	Type	Width [min]	Area [mAU*s]	Height [mAU]	Area %
1	4.697	MM	0.1125	3024.64429	448.14273	88.4172
2	5.415	MM	0.1276	396.23373	51.76438	11.5828
Totals :				3420.87802	499.90711	

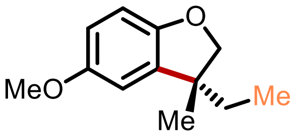

2c

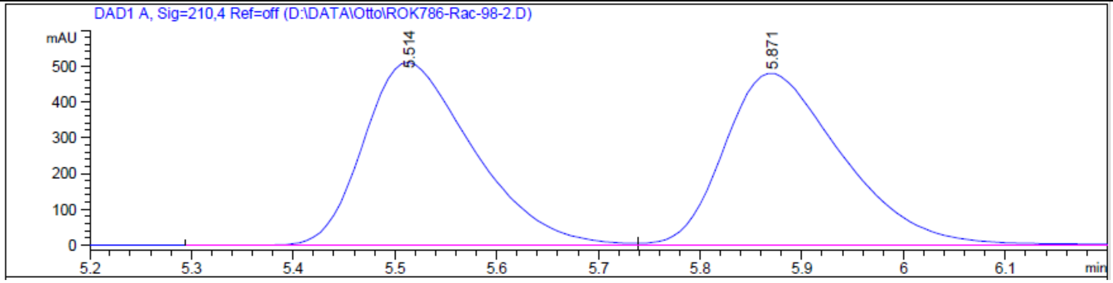

Signal 1: DAD1 A, Sig=210,4 Ref=off

Peak #	RetTime [min]	Type	Width [min]	Area [mAU*s]	Height [mAU]	Area %
1	5.514	BV	0.1132	3819.27246	510.78363	49.4672
2	5.871	VB	0.1227	3901.54663	480.45206	50.5328

Totals : 7720.81909 991.23569

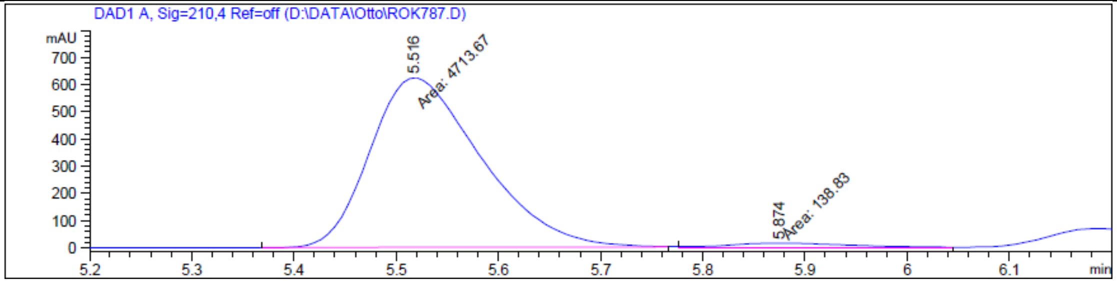

Signal 1: DAD1 A, Sig=210,4 Ref=off

Peak #	RetTime [min]	Type	Width [min]	Area [mAU*s]	Height [mAU]	Area %
1	5.516	MM	0.1257	4713.66846	625.02063	97.1390
2	5.874	MM	0.1386	138.83011	16.69037	2.8610

Totals : 4852.49857 641.71100

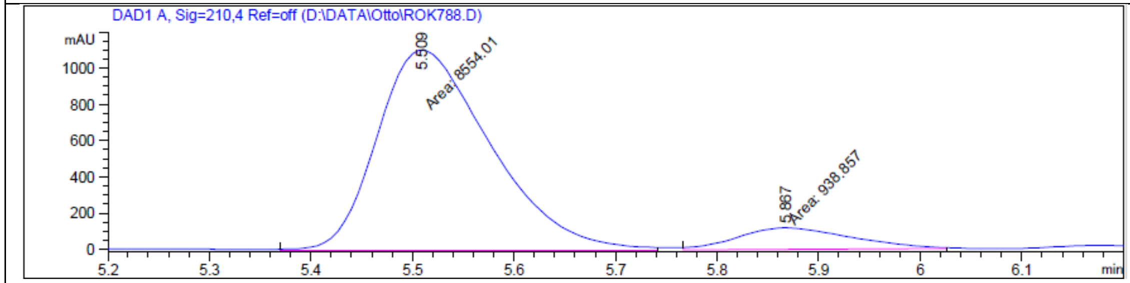

Signal 1: DAD1 A, Sig=210,4 Ref=off

Peak #	RetTime [min]	Type	Width [min]	Area [mAU*s]	Height [mAU]	Area %
1	5.509	MM	0.1289	8554.01172	1106.44702	90.1099
2	5.867	MM	0.1304	938.85706	119.95879	9.8901

Totals : 9492.86877 1226.40581

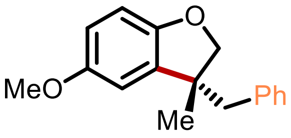

2d

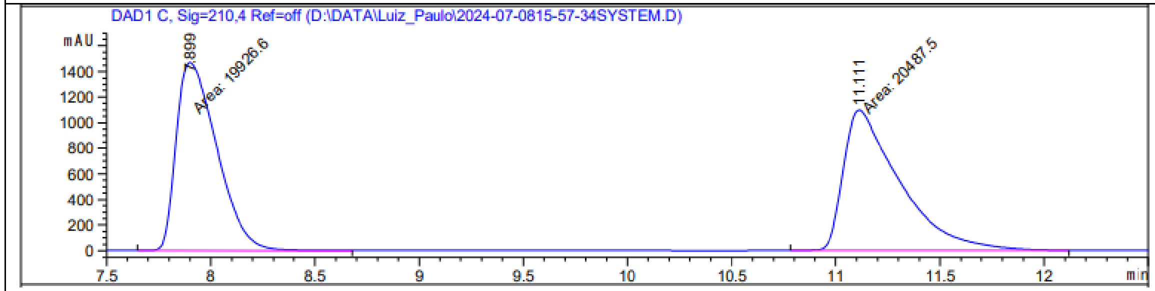

Signal 3: DAD1 C, Sig=210,4 Ref=off

Peak #	RetTime [min]	Type	Width [min]	Area [mAU*s]	Height [mAU]	Area %
1	7.899	MM	0.2262	1.99266e4	1468.12439	49.3060
2	11.111	MM	0.3118	2.04875e4	1095.07019	50.6940
Totals :				4.04141e4	2563.19458	

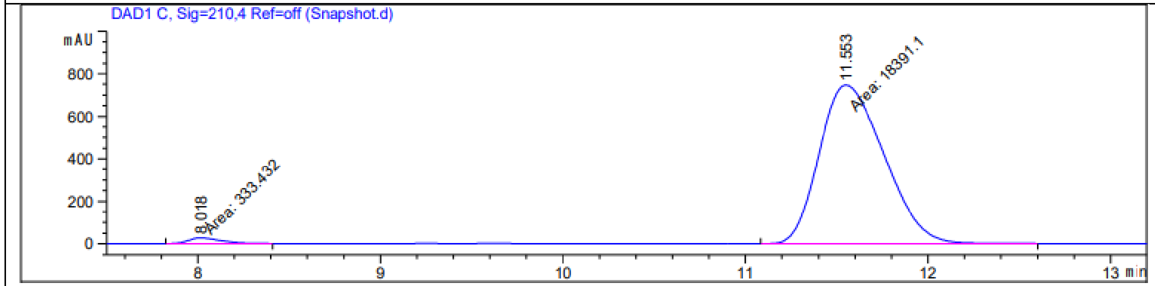

Signal 3: DAD1 C, Sig=210,4 Ref=off

Peak #	RetTime [min]	Type	Width [min]	Area [mAU*s]	Height [mAU]	Area %
1	8.018	MM	0.2080	333.43216	26.71289	1.7807
2	11.553	MM	0.4111	1.83911e4	745.66022	98.2193
Totals :				1.87246e4	772.37311	

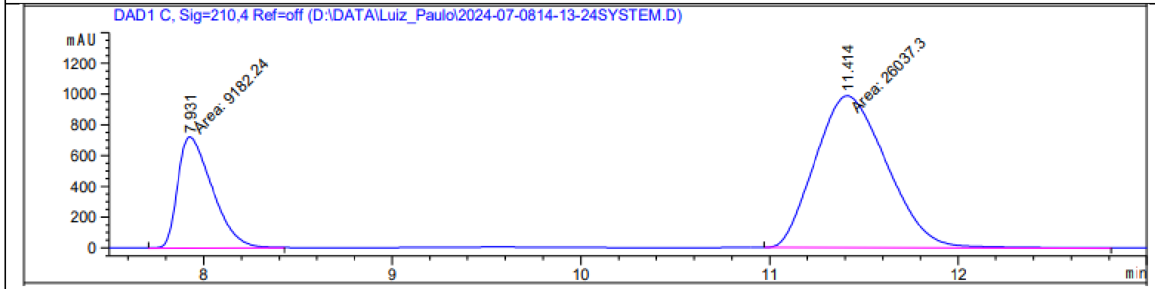

Signal 3: DAD1 C, Sig=210,4 Ref=off

Peak #	RetTime [min]	Type	Width [min]	Area [mAU*s]	Height [mAU]	Area %
1	7.931	MM	0.2117	9182.23730	723.00366	26.0714
2	11.414	PM	0.4394	2.60373e4	987.53784	73.9286
Totals :				3.52195e4	1710.54150	

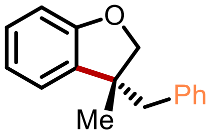

2e

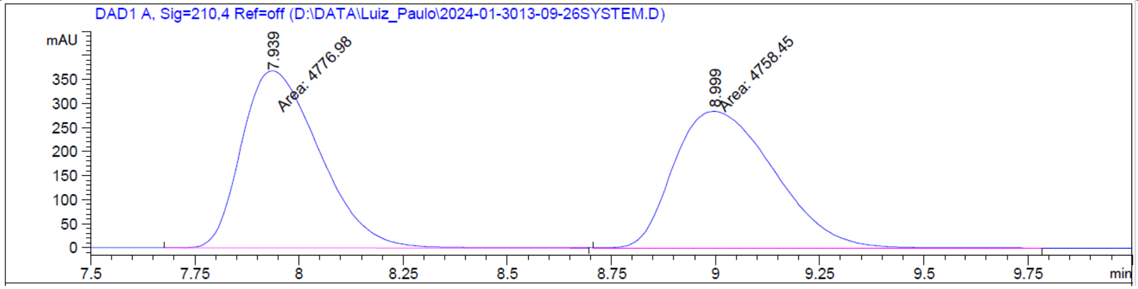

Signal 1: DAD1 A, Sig=210,4 Ref=off

Peak #	RetTime [min]	Type	Width [min]	Area [mAU*s]	Height [mAU]	Area %
1	7.939	MM	0.2163	4776.98096	368.07184	50.0972
2	8.999	MM	0.2796	4758.44678	283.67545	49.9028

Totals : 9535.42773 651.74728

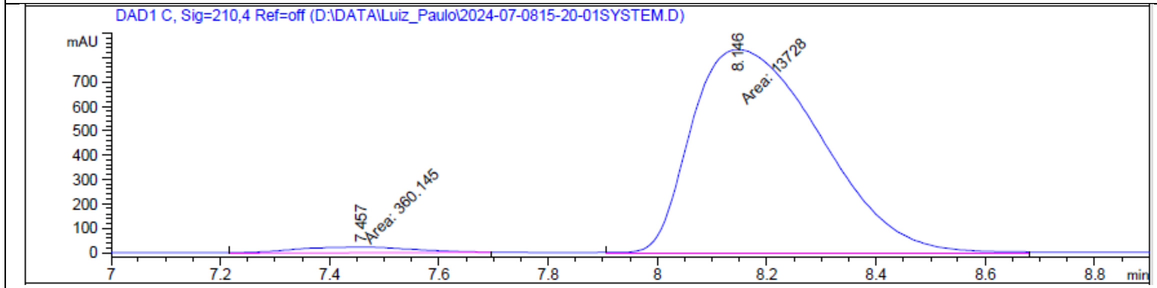

Signal 3: DAD1 C, Sig=210,4 Ref=off

Peak #	RetTime [min]	Type	Width [min]	Area [mAU*s]	Height [mAU]	Area %
1	7.457	MM	0.2531	360.14523	23.71863	2.5564
2	8.146	MM	0.2739	1.37280e4	835.37317	97.4436

Totals : 1.40881e4 859.09180

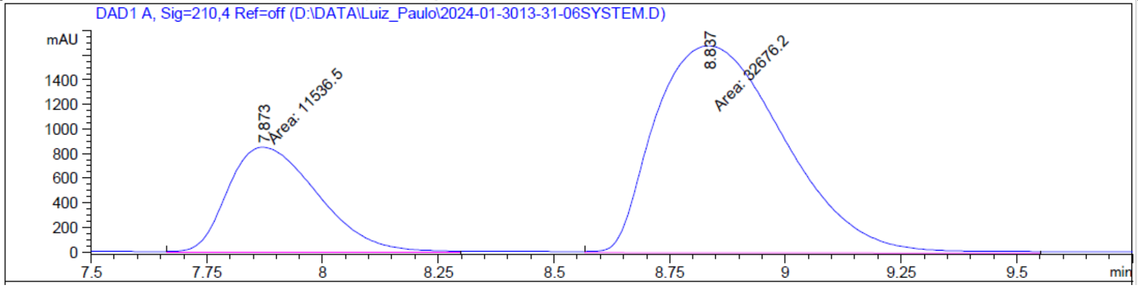

Signal 1: DAD1 A, Sig=210,4 Ref=off

Peak #	RetTime [min]	Type	Width [min]	Area [mAU*s]	Height [mAU]	Area %
1	7.873	MM	0.2252	1.15365e4	853.87836	26.0932
2	8.837	MM	0.3238	3.26762e4	1682.02991	73.9068

Totals : 4.42128e4 2535.90826

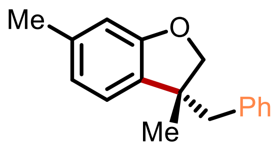

2f

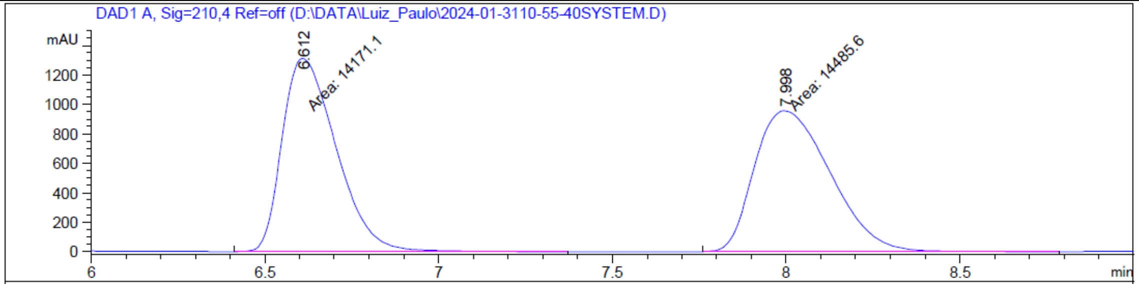

Signal 1: DAD1 A, Sig=210,4 Ref=off

Peak #	RetTime [min]	Type	Width [min]	Area [mAU*s]	Height [mAU]	Area %
1	6.612	MM	0.1802	1.41711e4	1310.73486	49.4512
2	7.998	MM	0.2526	1.44856e4	955.73859	50.5488
Totals :				2.86567e4	2266.47345	

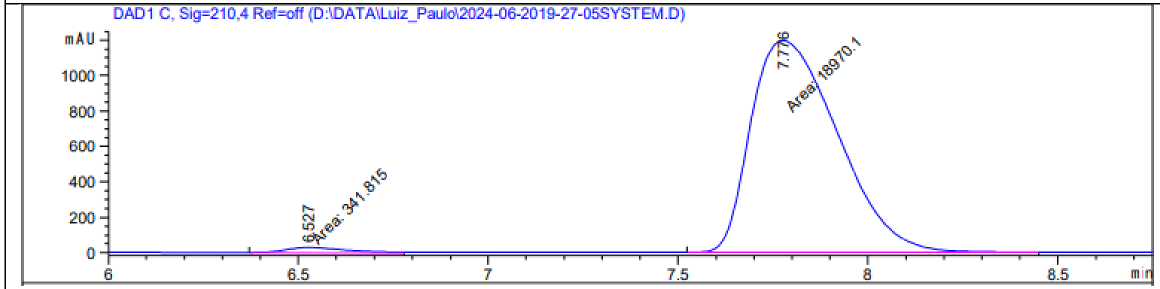

Signal 3: DAD1 C, Sig=210,4 Ref=off

Peak #	RetTime [min]	Type	Width [min]	Area [mAU*s]	Height [mAU]	Area %
1	6.527	MM	0.1891	341.81503	30.13287	1.7700
2	7.776	MM	0.2642	1.89701e4	1196.79346	98.2300
Totals :				1.93119e4	1226.92632	

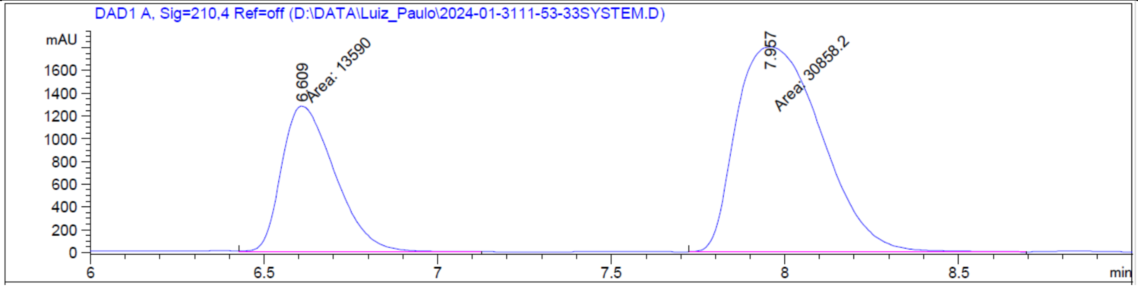

Signal 1: DAD1 A, Sig=210,4 Ref=off

Peak #	RetTime [min]	Type	Width [min]	Area [mAU*s]	Height [mAU]	Area %
1	6.609	MM	0.1771	1.35900e4	1278.94348	30.5749
2	7.957	MM	0.2850	3.08582e4	1804.58154	69.4251
Totals :				4.44482e4	3083.52502	

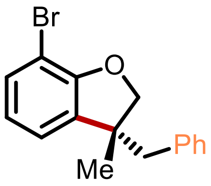

2g

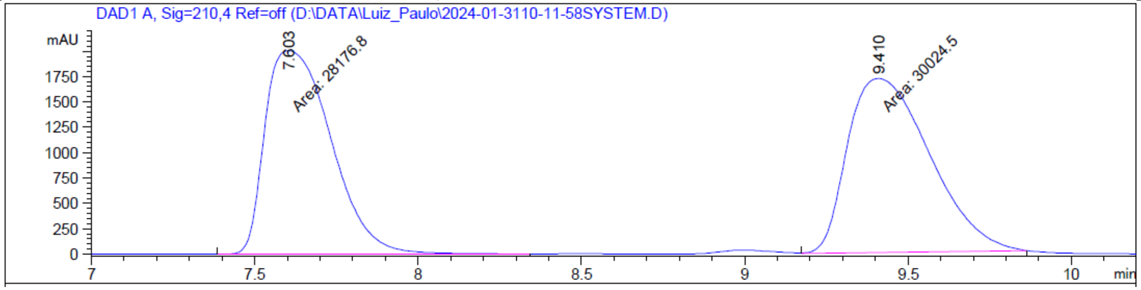

Signal 1: DAD1 A, Sig=210,4 Ref=off

Peak #	RetTime [min]	Type	Width [min]	Area [mAU*s]	Height [mAU]	Area %
1	7.603	MM	0.2342	2.81768e4	2005.36609	48.4127
2	9.410	MM	0.2915	3.00245e4	1716.81958	51.5873
Totals :				5.82014e4	3722.18567	

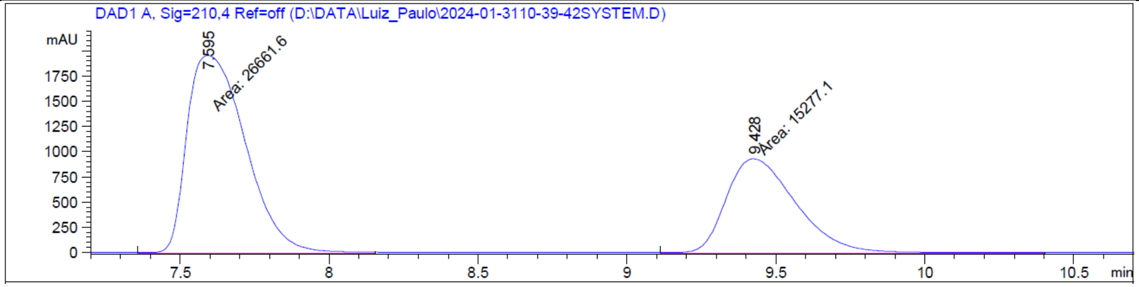

Signal 1: DAD1 A, Sig=210,4 Ref=off

Peak #	RetTime [min]	Type	Width [min]	Area [mAU*s]	Height [mAU]	Area %
1	7.595	MM	0.2270	2.66616e4	1957.75623	63.5728
2	9.428	MM	0.2727	1.52771e4	933.69012	36.4272
Totals :				4.19386e4	2891.44635	

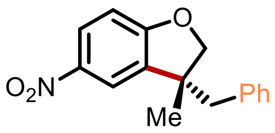

2h

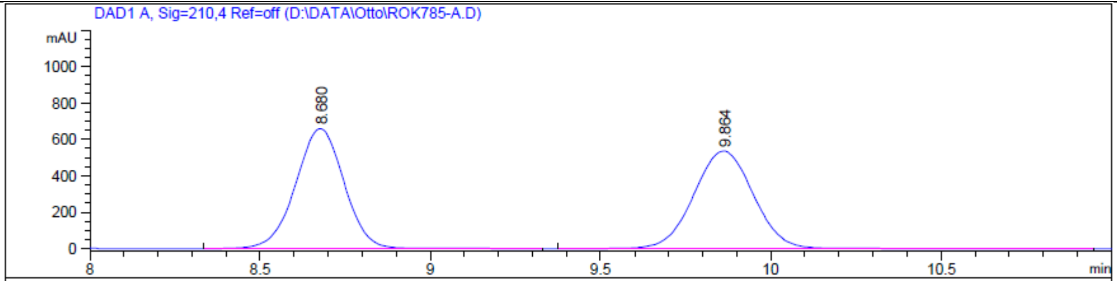

Signal 1: DAD1 A, Sig=210,4 Ref=off

Peak #	RetTime [min]	Type	Width [min]	Area [mAU*s]	Height [mAU]	Area %
1	8.680	BB	0.1536	6521.40674	657.96179	49.9638
2	9.864	BB	0.1923	6530.85498	535.30237	50.0362
Totals :				1.30523e4	1193.26416	

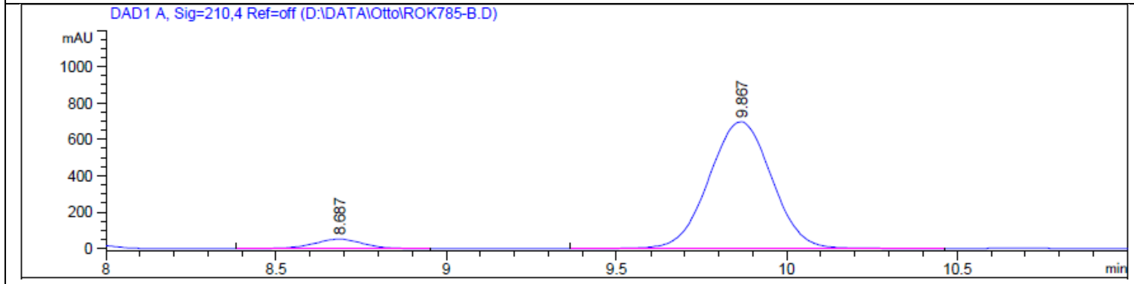

Signal 1: DAD1 A, Sig=210,4 Ref=off

Peak #	RetTime [min]	Type	Width [min]	Area [mAU*s]	Height [mAU]	Area %
1	8.687	BB	0.1532	506.28177	51.28068	5.3631
2	9.867	BB	0.2016	8933.77441	696.63977	94.6369
Totals :				9440.05618	747.92045	

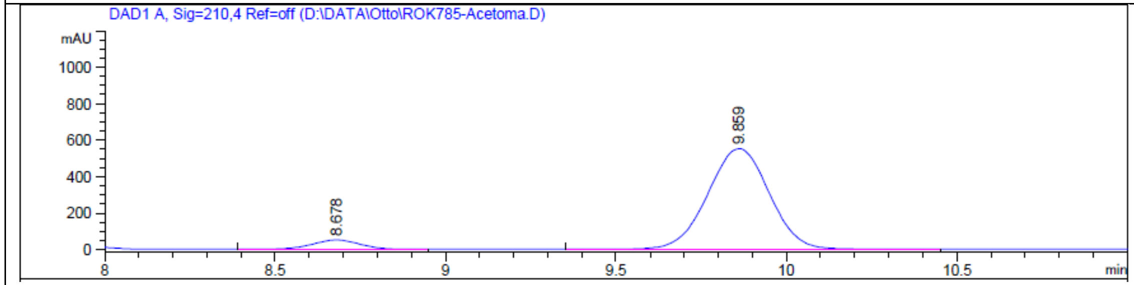

Signal 1: DAD1 A, Sig=210,4 Ref=off

Peak #	RetTime [min]	Type	Width [min]	Area [mAU*s]	Height [mAU]	Area %
1	8.678	BB	0.1533	511.21454	51.71057	6.8264
2	9.859	BB	0.1972	6977.58984	553.07135	93.1736
Totals :				7488.80438	604.78192	

8. Computational Data

DFT calculations were conduct in Gaussian16 software[10] using the hybrid functional B3LYP with the Grimme dispersion correction[11] and Alrich basis set Def2SVP[12]. The use of small basis set is due to computational time limitations. SMD[13] implicit solvation model was applied using acetone parameters as solvent. All structures were optimized and the thermal corrections were performed at the same level of theory. All transition states were confirmed by frequency analysis (one imaginary frequency) and further validated by Intrinsic Reaction Coordinate (IRC[14]) calculations, as implemented in Gaussian 16, to ensure that they correctly connect the intended reactants and products. Table S3 contains energy values for the optimized structures used to compute the thermochemistry of the catalytic cycle.

Table S3. Electronic, enthalpy corrected and free energy corrected energy for the optimized structures present in catalytic cycle. Values given in atomic units.

	Electronic Energy	Enthalpy	Gibbs Free Energy
A monodentate	-1161.414750	-1161.027719	-1161.106431
A + Acetone	-1354.500489	-1354.022118	-1354.112531
A + 2 Acetone	-1547.433798	-1546.863469	-1546.960792
Activated catalyst	-1733.365743	-1732.770320	-1732.876691
Re-B	-1623.927074	-1623.342424	-1623.440511
Re-TS1	-1623.918625	-1623.335737	-1623.431596
Si-B	-1623.938614	-1623.353682	-1623.450389
Si-TS1	-1623.924214	-1623.341122	-1623.437339
Int.1	-1623.958209	-1623.373485	-1623.471254
C	-1723.882069	-1723.293672	-1723.394903
D'	-1663.965711	-1663.343327	-1663.446634
TS 2	-1663.910723	-1663.290020	-1663.396378

[10] Gaussian 16, Revision C.01, M. J. Frisch, G. W. Trucks, H. B. Schlegel, G. E. Scuseria, M. A. Robb, J. R. Cheeseman, G. Scalmani, V. Barone, G. A. Petersson, H. Nakatsuji, X. Li, M. Caricato, A. V. Marenich, J. Bloino, B. G. Janesko, R. Gomperts, B. Mennucci, H. P. Hratchian, J. V. Ortiz, A. F. Izmaylov, J. L. Sonnenberg, D. Williams-Young, F. Ding, F. Lipparini, F. Egidi, J. Goings, B. Peng, A. Petrone, T. Henderson, D. Ranasinghe, V. G. Zakrzewski, J. Gao, N. Rega, G. Zheng, W. Liang, M. Hada, M. Ehara, K. Toyota, R. Fukuda, J. Hasegawa, M. Ishida, T. Nakajima, Y. Honda, O. Kitao, H. Nakai, T. Vreven, K. Throssell, J. A. Montgomery, Jr., J. E. Peralta, F. Ogliaro, M. J. Bearpark, J. J. Heyd, E. N. Brothers, K. N. Kudin, V. N. Staroverov, T. A. Keith, R. Kobayashi, J. Normand, K. Raghavachari, A. P. Rendell, J. C. Burant, S. S. Iyengar, J. Tomasi, M. Cossi, J. M. Millam, M. Klene, C. Adamo, R. Cammi, J. W. Ochterski, R. L. Martin, K. Morokuma, O. Farkas, J. B. Foresman, and D. J. Fox, Gaussian, Inc., Wallingford CT, 2016

[11] Grimme. S; Hansen. A; Brandenburg. J.C; Bannwarth. C; *Chem. Rev.* **2016**, 116, 9, 5105–5154

[12] Schäfer, *J; Horn. H; Ahlrihcs. R; Chem. Phys.* ***1992***, 97, 2571–2577

[13] Truhlar. D.G; Cramer. C. J; Marenich. A.V; *J. Phys. Chem. B* **2009**, 113, 18, 6378–6396

[14] Fukui. K; Acc. Chem. Res. 1981, 14, 12, 363–368

D	-1737.218028	-1736.621524	-1736.723300
TS 3	-1737.195587	-1736.600382	-1736.700486
Int. 2	-1737.237175	-1736.640000	-1736.740306
E	-1837.153581	-1836.553011	-1836.658939
F	-1777.239702	-1776.604798	-1776.711768
TS 4	-1777.206496	-1776.573541	-1776.680363
1a	-615.777628	-615.530783	-615.584047
2a	-502.523927	-502.288131	-502.335796
CO	-113.221493	-113.213078	-113.235508
F^-	-99.831645	-99.830700	-99.847219
$SnMe_4$	-373.920344	-373.766734	-373.816095
$SnMe_3F$	-433.840153	-433.720944	-433.769385
BOx^+	-571.794352	-571.588363	-571.640981
N_2	-109.435149	-109.426163	-109.447908
Acetone	-193.031822	-192.942586	-192.977216

8.1. Optimized Structures

*** A Monodentate

Pd 2.07668900 -0.27362800 0.66988900

N 0.11102600 -0.39268600 1.32475800

C -0.98954900 -0.00674300 0.77484000

C -0.23931600 -0.87568900 2.68534200

C -1.76771700 -1.02778700 2.60154000

H 0.24961200 -1.84244800 2.86020800

H -2.07634700 -2.06668900 2.40964300

N -0.18583400 1.33386900 -1.10357100

C -0.70490900 1.69381600 -2.43317500

C -2.21558300 1.38783600 -2.32499100

H -2.81911700 2.29337100 -2.15881500

O -2.12934600 -0.23525100 1.44112900

C -1.11248300 0.67510600 -0.52553300

O -2.30948400 0.56168500 -1.13565900

H -2.62705200 0.82231200 -3.17062900

H -2.31222800 -0.63551400 3.46888400

H -0.52809600 2.76298900 -2.62126200

C 0.04119000 0.89007100 -3.52544600

H -0.33038500 1.23463200 -4.50315900

H 1.10659800 1.16074400 -3.46267200

C 0.25796700 0.12366400 3.75186300

H 0.01552300 -0.30260100 4.73770300

H 1.35499800 0.16836600 3.66585500

C -0.12586800 -0.60486000 -3.40257200

C 0.63244000 -1.33930700 -2.47424200

C -1.07922500 -1.29048000 -4.17322600

C 0.43344300 -2.71245900 -2.31174200

H 1.37536100 -0.82863000 -1.85156000

C -1.27814800 -2.66599300 -4.01642400

H -1.67014500 -0.73907300 -4.90996100

C -0.52457100 -3.38167300 -3.08097700

H 1.03079500 -3.25941100 -1.57713900

H -2.02463500 -3.17970100 -4.62801500

H -0.68014900 -4.45633600 -2.95503900

C -0.33594300 1.50478100 3.61847600

C 0.17863300 2.41996200 2.68352300

C -1.44734900 1.88940600 4.38598200

C -0.41049500 3.67487300 2.51079700

H 1.04580800 2.13490300 2.08161100

C -2.03775900 3.14586200 4.21747500

H -1.85198100 1.19596500 5.12840300

C -1.52386100 4.04138600 3.27479200

H 0.00280600 4.37026100 1.77542000

H -2.90174300 3.42582500 4.82597300

H -1.98526800 5.02311500 3.13984400

*** A + Acetone

Pd 1.97641600 0.49932100 0.40236000

N 0.10544200 -0.50763100 1.44406300

C -0.96654900 -0.10764300 0.86659700

C -0.30301900 -1.09482700 2.73067100

C -1.83586200 -1.23354300 2.56989400

H 0.17237100 -2.07811000 2.85476900

H -2.14267300 -2.25896900 2.31744400

N 0.28222800 1.11886200 -0.73969600

C 0.10296200 1.71030300 -2.07777900

C -1.43509800 1.82199500 -2.18576000

H -1.79962600 2.83864100 -1.97914500

O -2.14575000 -0.38569800 1.42011100

C -0.86876200 0.68921800 -0.36139300

O -1.92929000 0.95327600 -1.11811800

H -1.85271500 1.46279000 -3.13369500

H -2.41976800 -0.87083800 3.42415700

H 0.57682500 2.70120700 -2.10608800

C 0.77371400 0.81756200 -3.14488100

H 0.63443200 1.31034300 -4.11952600

H 1.85191300 0.80681700 -2.92837900

C 0.15627800 -0.18610000 3.89409700

H -0.12996200 -0.68297500 4.83396500

H 1.25600900 -0.14601200 3.86085500

C 0.23531500 -0.59206700 -3.18576000

C 0.70258100 -1.56446800 -2.28413700

C -0.77842200 -0.95259400 -4.08829500

C 0.16133500 -2.85244400 -2.27530100

H 1.49252700 -1.30024800 -1.57484500

C -1.32092800 -2.24157600 -4.08346300

H -1.14351700 -0.21472600 -4.80799700

C -0.85574000 -3.19504000 -3.17287600

H 0.53813200 -3.59241200 -1.56415300

H -2.10882100 -2.50156100 -4.79524400

H -1.27919700 -4.20275000 -3.16679400

C -0.41973200 1.20802800 3.83970000

C 0.16476500 2.19388300 3.02566400

C -1.58138200 1.53865000 4.55641400

C -0.40419400 3.46529700 2.91827300

H 1.07084200 1.95457100 2.46065900

C -2.15224000 2.81125500 4.45333300

H -2.04134200 0.79045200 5.20787100

C -1.56809500 3.77781000 3.62908700

H 0.06637200 4.21564500 2.27719700

H -3.05605200 3.04833200 5.02077700

H -2.01380500 4.77245500 3.54613400

C 3.88378600 1.14852300 -0.02024400

O 3.96049500 0.31089700 0.98243000

C 4.08102500 2.63309200 0.26118900

H 3.61773900 3.25637300 -0.52048800

H 3.65267900 2.91062500 1.23566300

H 5.16162000 2.87715900 0.27843200

C 4.41288800 0.69701600 -1.37546500

H 4.01358100 1.31675200 -2.19350400

H 5.51602300 0.79569700 -1.39973500

H 4.16104600 -0.35543600 -1.57221800

*** A + 2 Acetone

N 0.22908500 -0.15655000 1.65740000

C -0.78718100 0.13112200 0.93043700

C -0.30183200 -0.59751600 2.96209300

C -1.80221900 -0.81204800 2.66062200

H 0.18124700 -1.53929700 3.25486800

H -2.05581200 -1.86900100 2.49389900

N 0.57973800 0.93182100 -0.85454700

C 0.41241300 1.37783100 -2.25205100

C -1.09224700 1.72067000 -2.31641900

H -1.28788600 2.79707100 -2.20421000

O -2.00760400 -0.10810200 1.39717500

C -0.60102100 0.73073100 -0.39611700

O -1.65080400 1.05011500 -1.14488600

H -1.60815900 1.33827400 -3.20488300

H -2.48109900 -0.37803700 3.40417400

H 1.02936100 2.26811100 -2.43244700

C 0.84985800 0.26577700 -3.23081200

H 0.66703100 0.63975500 -4.24979500

H 1.93438000 0.13323900 -3.12149400

C -0.02028500 0.46034500 4.05077900

H -0.46354500 0.08768900 4.98685400

H 1.06691400 0.50311700 4.20045600

C 0.13477500 -1.04565500 -3.01470400

C 0.53465100 -1.91980200 -1.98778400

C -0.97886100 -1.39753700 -3.79389100

C -0.16765200 -3.10207300 -1.74057800

H 1.39965000 -1.66506500 -1.37096900

C -1.68258000 -2.58129300 -3.54998100

H -1.29609000 -0.73612900 -4.60481900

C -1.28181100 -3.43552700 -2.51846900

H 0.15884800 -3.76669600 -0.93628900

H -2.54664900 -2.83666700 -4.16899400

H -1.83154600 -4.36024500 -2.32476400

C -0.56376800 1.82949200 3.72153700

C 0.11664800 2.67828700 2.82953900

C -1.78623900 2.26682800 4.25627100

C -0.41695900 3.92030800 2.47613300

H 1.06965500 2.35802700 2.40222600

C -2.32183000 3.51018400 3.90535300

H -2.32277900 1.62527600 4.96076300

C -1.64036200 4.33968000 3.00992500

H 0.12771400 4.56443800 1.78066800

H -3.27448900 3.83166000 4.33441700

H -2.05764800 5.31133700 2.73322000

C 3.93272100 0.66101000 -0.42816200

O 5.03788200 0.76786800 0.50999000

C 3.94987800 1.98640500 -1.17145800

H 3.20855900 1.98073000 -1.98320100

H 3.72785800 2.83329100 -0.50375300

H 4.94091600 2.16636900 -1.63377700

C 4.21218200 -0.51665400 -1.36023900

H 3.41753900 -0.64150600 -2.10821800

H 5.16676900 -0.36092600 -1.90245600

H 4.29535600 -1.45454900 -0.79245300

C 3.58818600 -0.11450900 2.07596900

C 3.70774400 1.04971400 3.05768800

H 4.48278100 0.83254900 3.82032500

H 3.99643300 1.97346400 2.53601100

H 2.76201500 1.23804800 3.58368200

C 3.32556400 -1.43110600 2.78796600

H 2.40634500 -1.36642700 3.38719400

H 3.21928500 -2.26596600 2.07803200

H 4.15365100 -1.67610100 3.48259500

O 4.88144000 -0.30743500 1.44177300

Pd 2.20116800 0.32719900 0.62627500

*** Activated Catalyst

Pd 0.06247100 0.00618500 0.02494400

N 0.04262900 0.02264000 2.21031000

C 1.23999700 0.00027200 2.67764500

C -0.86553800 -0.32057400 3.32011700

C 0.06470600 -0.16737500 4.54709700

H -1.68950500 0.40472500 3.35947400

H -0.06526800 0.79531500 5.05994300

N 2.05445900 0.18805200 0.46269000

C 3.28621400 0.54132600 -0.26932800

C 4.36006700 0.20845700 0.78736600

H 4.78333600 -0.79798600 0.66474300

O 1.41918300 -0.16518400 3.97012400

C 2.34975600 0.12469400 1.71752500

O 3.61214700 0.20141700 2.06075400

H 5.15866400 0.95232700 0.87828200

H 0.01061100 -0.98980700 5.26816200

H 3.38193900 -0.09394700 -1.15831700

C 3.23839600 2.02442900 -0.69811900

H 4.14537200 2.21564700 -1.29127200

H 2.37281700 2.15445800 -1.36221400

C -1.45420500 -1.73348200 3.10951200

H -2.07010200 -1.95954700 3.99370100

H -2.12926100 -1.68749100 2.24206500

C 3.15862100 2.98455600 0.46380000

C 1.93795500 3.21597100 1.12243600

C 4.30844500 3.62817200 0.94820400

C 1.87166600 4.05320900 2.23930900

H 1.03133100 2.73211400 0.75659900

C 4.24538800 4.46934800 2.06319200

H 5.26427100 3.47046700 0.44127700

C 3.02709000 4.68084700 2.71576600

H 0.91203900 4.21871200 2.73584500

H 5.15220700 4.96249400 2.42259800

H 2.97722100 5.33698500 3.58835600

C -0.40771800 -2.80204100 2.89825100

C 0.05071600 -3.10353300 1.60453000

C 0.17372400 -3.47685100 3.98401500

C 1.07445500 -4.03212900 1.40102300

H -0.40212900 -2.60305600 0.74579500

C 1.19810300 -4.40763800 3.78502000

H -0.18319500 -3.27641300 4.99749400

C 1.65706300 -4.68320000 2.49342700

H 1.41499000 -4.25039500 0.38550300

H 1.63747600 -4.92170400 4.64369500

H 2.45842500 -5.40990700 2.33788900

C 1.15736500 -1.78567800 -3.64279300

C 0.74791600 -1.50223200 -2.33533700

C 0.46259100 -0.19118900 -1.92758800

C 0.56874000 0.85286400 -2.86432600

C 0.98227600 0.56340200 -4.17661600

C 1.28107700 -0.74130300 -4.56429800

H 1.38000300 -2.81519100 -3.93351000

H 0.66569100 -2.32021700 -1.61472800

H 1.05964200 1.39584100 -4.88002700

H 1.60127000 -0.94209000 -5.58969500

O 0.36439300 2.17769100 -2.59766100

C -0.75065100 2.57461200 -1.79657700

H -1.64483500 1.99857100 -2.08391800

H -0.53624600 2.36790700 -0.73048000

C -0.98117100 4.05095000 -1.98011300

C 0.21293400 4.94085000 -1.77946200

H 0.64349200 4.79980900 -0.77368300

H 1.00924000 4.68681300 -2.49798900

H -0.04782200 6.00188300 -1.90482800

C -2.20635000 4.51281300 -2.26521700

H -2.40465500 5.58604600 -2.34953400

H -3.05225100 3.83432300 -2.41489400

N -1.88010100 -0.27994100 -0.36975600

N -2.95027800 -0.46306100 -0.56169000

*** Re-B

Pd 2.03768800 0.67265300 0.04371800

N 0.45631500 -0.10995400 1.22283300

C -0.70540200 0.04132000 0.68708400

C 0.23580400 -0.79594700 2.51885400

C -1.17625800 -1.37811900 2.31371200

H 0.98565000 -1.58519100 2.65277800

H -1.16522900 -2.40888200 1.93150000

N 0.25185600 1.25601700 -1.10646100

C -0.14741400 1.73660700 -2.44897800

C -1.66793100 1.92058900 -2.26948200

H -1.94224400 2.94610500 -1.98347900

O -1.74403100 -0.53548400 1.25154700

C -0.82270800 0.82797000 -0.54565900

O -1.98392300 1.05505400 -1.12553600

H -2.27321500 1.58841600 -3.11996200

H -1.83020100 -1.30170500 3.18898100

H 0.34921400 2.68918500 -2.67402700

C 0.22598300 0.69204600 -3.53184800

H -0.22172700 1.04170400 -4.47458500

H 1.31537100 0.70301100 -3.67255500

C 0.31173500 0.19925500 3.70122100

H -0.03317300 -0.34813900 4.59158200

H 1.36217100 0.45940200 3.86747200

C -0.23885800 -0.70717200 -3.20551400

C 0.53162500 -1.53266700 -2.36844200

C -1.47423700 -1.18972400 -3.66478400

C 0.07349000 -2.79574700 -1.98634500

H 1.49554900 -1.17492200 -1.99949700

C -1.93493600 -2.45487700 -3.28755400

H -2.08258400 -0.56552400 -4.32470400

C -1.16543700 -3.25947900 -2.44172600

H 0.68812700 -3.41913500 -1.33171200

H -2.89998500 -2.81257400 -3.65553300

H -1.52663300 -4.24699900 -2.14353300

C -0.50832400 1.44873900 3.49336900

C 0.00778200 2.51530000 2.73642000

C -1.81713000 1.55356400 3.98897400

C -0.76627900 3.64798200 2.47253300

H 1.02565300 2.45048600 2.34406200

C -2.59429900 2.68651500 3.72853000

H -2.23190100 0.73708200 4.58609300

C -2.07305900 3.73516800 2.96493000

H -0.34675700 4.46630300 1.88155000

H -3.61146300 2.74946400 4.12369500

H -2.68037200 4.62014500 2.75862000

C 3.39580300 -0.10220100 1.29178300

C 4.19074800 -1.17357800 0.85762900

C 3.52661600 0.34812000 2.60640100

C 5.00682600 -1.86737600 1.75809900

C 4.35895900 -0.32769300 3.51091800

H 2.97979700 1.23225000 2.93709000

C 5.07466200 -1.45303500 3.09244100

H 5.59962200 -2.70914600 1.39234000

H 4.44050600 0.02794100 4.54118100

H 5.71337500 -1.99308500 3.79582900

O 4.20233300 -1.53787800 -0.46346000

C 4.49690300 -0.50509500 -1.42084100

H 5.56896100 -0.57381900 -1.68028400

H 3.90400700 -0.73408000 -2.31598900

C 4.22137600 0.91573100 -0.96067600

C 3.20922400 1.64371800 -1.54979100

H 3.13483500 2.72168900 -1.37680600

H 2.69399400 1.24240400 -2.42123100

C 5.28350200 1.60343100 -0.14486100

H 5.74844200 0.94422300 0.59813200

H 6.07528600 1.91688300 -0.84885600

H 4.89560400 2.50036900 0.35643500

*** Re-TS1

Pd 0.97352607 0.44893579 -0.32577910

N -0.57348693 -0.45873521 0.95460390

C -1.74774593 -0.30449221 0.45714790

C -0.75339693 -1.13501321 2.25593390

C -2.19346393 -1.67753621 2.13259590

H -0.02122593 -1.94717021 2.35537190

H -2.22914293 -2.72303421 1.79424390

N -0.83439593 0.94872779 -1.33809810

C -1.24296893 1.45065979 -2.66556810

C -2.77289693 1.56063979 -2.49704010

H -3.09879993 2.57128379 -2.21265710

O -2.78081693 -0.85925221 1.06402290

C -1.89315593 0.47906079 -0.77722110

O -3.05946893 0.67844079 -1.35784910

H -3.35424593 1.20119279 -3.35330910

H -2.80783093 -1.54893821 3.03049790

H -0.78621593 2.43118479 -2.85311410

C -0.80143493 0.46287179 -3.77117810

H -1.15884993 0.87357279 -4.72764010

H 0.29646807 0.46155379 -3.80780410

C -0.56128193 -0.13489421 3.41909490

H -0.77031993 -0.68383221 4.35007390

H 0.49560007 0.15532479 3.43868690

C -1.31957593 -0.94156321 -3.57514410

C -0.68878193 -1.82118421 -2.67734310

C -2.47360193 -1.38370021 -4.24120210

C -1.20549593 -3.09770321 -2.44142610

H 0.20902607 -1.49714521 -2.14505010

C -2.99130093 -2.66182221 -4.01015310

H -2.96991093 -0.71824921 -4.95260510

C -2.36164193 -3.52147921 -3.10511410

H -0.70086693 -3.76422521 -1.73706710

H -3.89021393 -2.98683721 -4.54029210

H -2.76671593 -4.51993821 -2.92208810

C -1.44074393 1.08646779 3.31514490

C -1.06856593 2.16361779 2.49145590

C -2.67209193 1.15426679 3.98592990

C -1.90871393 3.26872579 2.33276690

H -0.11189893 2.13030879 1.96492790

C -3.51459693 2.25938279 3.83093390

H -2.97212993 0.33082679 4.63964890

C -3.13769793 3.31831479 2.99949390

H -1.60106193 4.09574079 1.68744090

H -4.46881193 2.29313079 4.36304590

H -3.79618693 4.18190579 2.87629290

C 2.60623307 -0.13559221 0.82609290

C 3.26408707 -1.37334621 0.74771290

C 2.63099307 0.56599779 2.04342290

C 3.72777207 -2.01447921 1.90600790

C 3.13184807 -0.04314421 3.19648990

H 2.21716507 1.57572179 2.10043790

C 3.64298107 -1.34855221 3.12874290

H 4.20598207 -2.99261821 1.81945190

H 3.11343107 0.49288179 4.14799990

H 4.02301507 -1.83357021 4.03130190

O 3.58210507 -1.89459821 -0.46065910

C 3.41531007 -0.98309321 -1.55013810

H 4.29749107 -1.09938721 -2.20110010

H 2.51469507 -1.25675121 -2.12053210

C 3.30426807 0.46139379 -1.07893710

C 2.29338907 1.24491679 -1.70587410

H 2.37230907 2.33537979 -1.62827010

H 1.87761507 0.87382079 -2.64675110

C 4.56067007 1.13457179 -0.58479410

H 5.16028707 0.48430679 0.06568390

H 5.16375007 1.39000079 -1.47479110

H 4.32971007 2.06633979 -0.05141710

*** Si-B

Pd 6.61265100 0.97152800 3.00293600

N 5.75987600 2.34078100 4.37490900

C 5.69231700 1.90138600 5.58315500

C 4.90079400 3.54016700 4.29500500

C 4.67433800 3.85787800 5.79008300

H 5.44791100 4.35136100 3.80071200

H 5.34338700 4.64615700 6.16163800

N 6.79105100 -0.07787800 4.90501400

C 7.57521000 -1.18185000 5.49478100

C 7.06605900 -1.17954100 6.95379200

H 6.27411500 -1.92194900 7.12612900

O 5.05109600 2.61589400 6.48202500

C 6.31366600 0.60507700 5.88394800

O 6.45238200 0.14672100 7.11105300

H 7.84914300 -1.27951200 7.71317700

H 3.63669700 4.08596300 6.05644900

H 7.32853000 -2.12717300 4.99416700

C 9.08906800 -0.90279200 5.33344800

H 9.62038100 -1.65495000 5.93699700

H 9.35777300 -1.07714100 4.28243400

C 3.62208300 3.24199300 3.48333900

H 3.01606200 4.16072200 3.49594600

H 3.92795200 3.06254700 2.44311100

C 9.48535900 0.49714400 5.73874300

C 9.47429400 1.53462300 4.79119900

C 9.79365200 0.81429400 7.07087700

C 9.74077700 2.85376400 5.16530800

H 9.23911300 1.30511900 3.74846000

C 10.06301400 2.13327600 7.44984800

H 9.82342800 0.01956600 7.82072000

C 10.03041600 3.15850800 6.49972200

H 9.72386000 3.64527600 4.41162200

H 10.29930500 2.36008700 8.49265200

H 10.23811200 4.18978300 6.79617400

C 2.82477700 2.07283100 4.00884900

C 3.14127100 0.76063700 3.61757000

C 1.78709400 2.25867500 4.93648400

C 2.45548900 -0.33396200 4.14939800

H 3.94014000 0.59936700 2.89062500

C 1.09687300 1.16579700 5.47030700

H 1.51259200 3.27265700 5.23960300

C 1.43260600 -0.13492600 5.08276100

H 2.71891200 -1.34588300 3.83016500

H 0.29138400 1.33208700 6.19021500

H 0.89410000 -0.98960800 5.50002400

C 6.46805600 2.35821200 1.55080500

C 5.77790300 2.26123800 0.33146300

C 7.06738600 3.58625400 1.87751100

C 5.61443600 3.39846900 -0.48102900

C 6.93642100 4.71046900 1.05645800

H 7.63644000 3.67510100 2.80607100

C 6.18242300 4.61701800 -0.11858100

H 5.05358500 3.28863000 -1.41223500

H 7.41153300 5.65226900 1.34156900

H 6.05111700 5.48894900 -0.76429700

O 5.26373000 1.11166900 -0.18603500

C 5.60496400 -0.15071800 0.36984300

H 4.84476900 -0.46387200 1.10352400

H 5.56965900 -0.85556400 -0.47858600

C 6.97937700 -0.21819600 0.98341900

C 7.16085400 -0.96732900 2.12566500

H 8.17222500 -1.22772600 2.44462300

H 6.33868000 -1.55740100 2.54040200

C 8.14955100 0.20855100 0.14016300

H 7.98090400 1.16190900 -0.37528300

H 9.06805500 0.28252700 0.73819900

H 8.30106400 -0.56331100 -0.63514000

*** Si-TS1

Pd 6.59083200 0.90105500 3.11145800

N 5.66684400 2.43936300 4.36354800

C 5.61664600 2.08833400 5.59698900

C 4.87781700 3.67630600 4.22545400

C 4.66038700 4.08368400 5.70387500

H 5.47523200 4.43197300 3.69851300

H 5.34407900 4.88200200 6.02436200

N 6.69366700 0.04941100 5.04600600

C 7.40251400 -1.06713400 5.69727700

C 6.90361100 -0.94880500 7.15668600

H 6.09999800 -1.66114300 7.38858900

O 5.01806500 2.88091600 6.46715700

C 6.21456000 0.79793300 5.97624200

O 6.31884600 0.39740500 7.22724900

H 7.69145800 -1.01025500 7.91561800

H 3.62957900 4.35175300 5.95969500

H 7.08335400 -2.01737900 5.24836600

C 8.92859200 -0.91634700 5.51083100

H 9.40405600 -1.73959300 6.06581300

H 9.15034500 -1.06763900 4.44482800

C 3.59496200 3.40797100 3.40602900

H 2.95684000 4.30076800 3.49889000

H 3.88768600 3.32072800 2.35117100

C 9.46596000 0.41739300 5.97153500

C 9.45319800 1.52772000 5.10950500

C 9.93707200 0.59698300 7.28198100

C 9.88132800 2.78298600 5.54820300

H 9.09556700 1.40505800 4.08392100

C 10.36806700 1.85155800 7.72459200

H 9.97090500 -0.25798400 7.96256700

C 10.33619400 2.95021000 6.86065600

H 9.86262400 3.63320700 4.86130100

H 10.73228200 1.96964600 8.74837200

H 10.67208600 3.93130300 7.20590400

C 2.85724900 2.16182300 3.83494200

C 3.08115000 0.94892300 3.16236500

C 1.99381800 2.15905600 4.94201600

C 2.47951300 -0.23586600 3.59372800

H 3.73244500 0.93854800 2.28786700

C 1.39092900 0.97502600 5.37880100

H 1.78344300 3.09384600 5.46740400

C 1.63702200 -0.22817200 4.71044900

H 2.66759200 -1.16770700 3.05395000

H 0.72260000 0.99443400 6.24356500

H 1.16585100 -1.15339600 5.05177800

C 6.50949900 2.00681500 1.32644400

C 5.48582900 2.05390300 0.36746900

C 7.26469300 3.17234500 1.55913200

C 5.07988100 3.27069100 -0.19974000

C 6.88015200 4.38743900 0.98940800

H 8.12735000 3.14152900 2.22909900

C 5.76856700 4.43595000 0.13272400

H 4.26097300 3.27549300 -0.92218700

H 7.44399100 5.29594000 1.21292600

H 5.46212800 5.38646600 -0.31083100

O 4.96008500 0.90226100 -0.11045800

C 5.63086500 -0.26525000 0.36466300

H 5.04328000 -0.71577700 1.17990500

H 5.67697400 -0.97375200 -0.47774800

C 7.03465200 0.05006300 0.86354000

C 7.42762800 -0.64608000 2.05086800

H 8.50160800 -0.74829700 2.24095800

H 6.81940000 -1.50420500 2.36105700

C 8.08741700 0.37867900 -0.16891100

H 7.71932300 1.06917700 -0.93945900

H 8.98084400 0.80982000 0.30305200

H 8.38023600 -0.56746300 -0.65785300

*** Int. 1

Pd 2.01926900 0.68228500 0.16206100

N 0.29606000 -0.16030700 0.98226800

C -0.81375200 0.31838400 0.53909000

C -0.01609700 -0.99235700 2.15836800

C -1.54617200 -1.16219200 2.02000600

H 0.49956600 -1.95798300 2.07292400

H -1.82730700 -2.11955000 1.55972900

N 0.36938400 1.74432700 -0.94513400

C 0.11601100 2.50610100 -2.18396000

C -1.41111400 2.73310300 -2.11606600

H -1.67430100 3.72053500 -1.71064900

O -1.93884800 -0.10224400 1.08055500

C -0.77161300 1.31680800 -0.54013700

O -1.87055500 1.73169400 -1.14764600

H -1.94252800 2.56055000 -3.05866000

H -2.10723600 -1.00917700 2.94848100

H 0.65929800 3.45996700 -2.15598900

C 0.60132600 1.68942200 -3.40540100

H 0.23007900 2.20047600 -4.30744500

H 1.69740500 1.73948300 -3.42220600

C 0.44674900 -0.28964400 3.45334600

H 0.17146900 -0.94734400 4.29192400

H 1.54394800 -0.23183500 3.42636400

C 0.16012400 0.24553000 -3.37558600

C 0.98929400 -0.72922200 -2.79523400

C -1.10122300 -0.14883500 -3.84812500

C 0.56558100 -2.05447900 -2.67252000

H 1.97684000 -0.44040700 -2.43144200

C -1.52982100 -1.47472100 -3.72789100

H -1.75657500 0.58992000 -4.31628200

C -0.70039200 -2.43092700 -3.13381500

H 1.22738100 -2.79540600 -2.21640400

H -2.51623300 -1.76170100 -4.10131100

H -1.03572500 -3.46682100 -3.03865000

C -0.14870900 1.08469100 3.64115600

C 0.42851000 2.20671900 3.02126900

C -1.32153500 1.26689000 4.39142800

C -0.15733000 3.46997700 3.13565800

H 1.34356500 2.08408600 2.43604900

C -1.90962400 2.52996200 4.50949400

H -1.77574600 0.40853200 4.89367000

C -1.33175500 3.63518300 3.87767600

H 0.30770000 4.32914600 2.64509700

H -2.82188600 2.65068000 5.09936100

H -1.79079400 4.62287300 3.96882600

C 3.36947300 -0.44227000 1.15293200

H 3.04445800 -1.49231300 1.17046700

H 3.48631700 -0.04808200 2.17313700

C 4.55920500 -0.13576800 0.25561500

C 4.14809800 1.17393300 -0.43794000

C 4.61729500 -1.07222900 -0.98527100

C 4.30339000 1.01256100 -1.83525100

C 3.86262200 2.44558600 0.10214300

H 3.71293900 -1.69686800 -1.05709700

C 4.14451200 2.07835100 -2.71306700

C 3.67391500 3.52186400 -0.78381100

H 3.84926600 2.60297000 1.18270000

C 3.81719200 3.32920200 -2.16139100

H 4.27745300 1.94502600 -3.78762700

H 3.43369600 4.51183100 -0.39196700

H 3.67824900 4.17775300 -2.83591500

O 4.66076700 -0.23668800 -2.17528000

H 5.50580100 -1.71691800 -1.00055000

C 5.90329800 -0.02562500 0.97760000

H 5.85563400 0.73620400 1.77073700

H 6.16946700 -0.98998000 1.44011800

H 6.70289000 0.25736800 0.27345900

*** C

Pd 1.61261400 0.45712500 -0.63895000

N 0.23971800 -0.14056000 0.76865900

C -0.99238200 0.16112500 0.53193600

C 0.29604800 -0.70530000 2.13131700

C -1.19231600 -1.00076500 2.41112800

H 0.89061800 -1.62813500 2.12416000

H -1.45766500 -2.05206900 2.23034100

N -0.35827000 1.38512300 -1.39197600

C -0.95546600 1.99437000 -2.59134700

C -2.47230800 1.96840300 -2.26229100

H -2.86280000 2.96110600 -1.99894000

O -1.90723600 -0.19571200 1.41610400

C -1.31889400 0.92657000 -0.67826000

O -2.57087300 1.13079200 -1.06430000

H -3.10159400 1.51914500 -3.03942300

H -1.53717400 -0.69016200 3.40377300

H -0.60105700 3.03137100 -2.67884400

C -0.52695000 1.23546700 -3.86725000

H -1.13331800 1.62853600 -4.69867400

H 0.52073600 1.49875400 -4.06913000

C 0.94717100 0.30407300 3.10229600

H 0.95054300 -0.16349900 4.09875300

H 1.99433800 0.43046000 2.79466200

C -0.65734100 -0.26595200 -3.76425800

C 0.45076000 -1.03733700 -3.37325000

C -1.87728200 -0.91957200 -3.99990700

C 0.33735400 -2.41973300 -3.20439000

H 1.40308800 -0.53690600 -3.18458300

C -1.99401100 -2.30324000 -3.82988200

H -2.74583600 -0.34324100 -4.32844400

C -0.88772800 -3.05774000 -3.42674900

H 1.21179300 -2.99895200 -2.89555300

H -2.95272800 -2.79381200 -4.01792600

H -0.97850500 -4.13896000 -3.29383600

C 0.25036300 1.64223000 3.14974500

C 0.49009500 2.61103400 2.15902900

C -0.68691900 1.93213800 4.15410500

C -0.19645700 3.82783200 2.16695700

H 1.21720800 2.40483300 1.37072200

C -1.37463300 3.14972700 4.16522700

H -0.87577700 1.19607700 4.94032700

C -1.13453900 4.10029400 3.16844000

H 0.00513900 4.56644900 1.38671500

H -2.09936900 3.35638500 4.95704200

H -1.67204800 5.05201600 3.17519400

C 3.11872200 -0.63215100 0.17614300

H 3.06898900 -1.61517700 -0.32934500

H 2.85299000 -0.79513500 1.23096600

C 4.55028900 -0.08623000 0.09976000

C 4.67615300 1.41452400 0.30577400

C 5.17013400 -0.22270600 -1.31837500

C 5.50533900 1.92991100 -0.69931900

C 4.16655900 2.25669400 1.28539200

H 4.38110100 -0.28495900 -2.07693000

C 5.83815900 3.28277400 -0.75710000

C 4.48079700 3.62729300 1.24410600

H 3.52418600 1.86385900 2.07584700

C 5.30940400 4.12714500 0.23292500

H 6.47989700 3.66678900 -1.55301300

H 4.07418600 4.30152900 2.00177800

H 5.54810500 5.19386800 0.20573100

O 5.93057400 0.98655400 -1.57300700

H 5.85871100 -1.07600200 -1.40354100

C 5.42809700 -0.81839700 1.13840900

H 5.08602900 -0.59938800 2.16231000

H 5.38278100 -1.90954400 0.98503500

H 6.48131400 -0.50295900 1.05597100

F 2.73542500 1.10610800 -2.17007400

*** D'

Pd 1.96080100 0.25645000 -0.85978100

N 0.40874200 -0.25858500 0.67570500

C -0.77241900 0.10560200 0.33059400

C 0.31424600 -0.74921000 2.06097600

C -1.21186700 -0.89532500 2.26021200

H 0.82328300 -1.71960600 2.14237400

H -1.55883600 -1.93244700 2.14620300

N 0.05537100 1.00852300 -1.70189200

C -0.45832900 1.47899300 -2.99977000

C -1.93145500 1.80694700 -2.66858400

H -2.09424500 2.87763300 -2.47754100

O -1.78503900 -0.11913900 1.16012300

C -0.96682500 0.77083200 -0.96311000

O -2.17367900 1.09730200 -1.41107100

H -2.65661900 1.44371300 -3.40587800

H -1.58835400 -0.47588700 3.20076200

H 0.09062200 2.37754600 -3.31287900

C -0.26850100 0.38428400 -4.07572000

H -0.66193500 0.78658900 -5.02199700

H 0.81215100 0.23028600 -4.20389800

C 0.99534400 0.24310600 3.02722400

H 0.92174200 -0.18178400 4.04005700

H 2.06045900 0.27606100 2.75993800

C -0.94665500 -0.92090700 -3.73740200

C -0.33503900 -1.83985300 -2.86644200

C -2.22283600 -1.22432200 -4.23834600

C -0.98749300 -3.01849200 -2.49571400

H 0.65837000 -1.61776300 -2.46589900

C -2.87725900 -2.40450600 -3.87145000

H -2.70761900 -0.52828500 -4.92839000

C -2.26331900 -3.30364100 -2.99432500

H -0.49542200 -3.71839500 -1.81504800

H -3.87011400 -2.62228600 -4.27374400

H -2.77436500 -4.22561200 -2.70511000

C 0.40205800 1.63042300 2.99814500

C 0.69587700 2.51577400 1.94548000

C -0.48680900 2.05552600 3.99856800

C 0.11240900 3.78410400 1.89316400

H 1.38773700 2.20549000 1.15993400

C -1.07203100 3.32485500 3.94901700

H -0.71835800 1.38407900 4.83007200

C -0.77599500 4.19291000 2.89368500

H 0.35897100 4.45727100 1.06786300

H -1.75959300 3.63696100 4.73951200

H -1.23142300 5.18580200 2.85372700

C 3.53786700 -0.63358100 0.10994900

H 3.76561600 -1.58513000 -0.40879900

H 3.07660300 -0.91644700 1.07297800

C 4.89167800 0.04811800 0.43142400

C 4.77048600 1.52650500 0.76709200

C 5.84480800 0.11712700 -0.80188600

C 5.43625200 2.27462900 -0.21161700

C 4.14891300 2.18224000 1.82378200

H 5.44419800 -0.41006600 -1.67687000

C 5.46577900 3.66820600 -0.19151100

C 4.16967600 3.58667800 1.87385400

H 3.64126900 1.61512500 2.60585000

C 4.81602300 4.31706400 0.87024500

H 5.98408900 4.22680800 -0.97374800

H 3.67280200 4.10815200 2.69512000

H 4.82405300 5.40954300 0.91323200

O 6.02624200 1.50842200 -1.16220500

H 6.84050500 -0.29463800 -0.56947200

C 5.57835600 -0.71755800 1.57965200

H 4.95668200 -0.69636900 2.48865900

H 5.73305400 -1.77366000 1.30189100

H 6.55961100 -0.27988400 1.82681400

C 3.09549600 0.75467400 -2.47593500

H 4.02176500 1.29978400 -2.25522600

H 3.36594800 -0.15013300 -3.05119900

H 2.47451000 1.39534700 -3.12672600

*** TS2

Pd 1.00250727 -0.25862824 -1.31198544

N -0.64157573 -0.76330824 1.52561856

C -1.77095473 -0.53739224 0.97777456

C -0.93547973 -1.19919424 2.90128456

C -2.44400973 -1.52084524 2.85674156

H -0.33992473 -2.09129524 3.14315056

H -2.64333873 -2.59629224 2.73083856

N -1.05195373 0.30657176 -1.22084944

C -1.73428473 0.81560676 -2.43417744

C -3.14333873 1.15107376 -1.91278644

H -3.26875673 2.22280176 -1.69703444

O -2.89461573 -0.83906624 1.65638656

C -1.96757773 0.08181876 -0.34719544

O -3.22129973 0.43473576 -0.65254244

H -3.96208073 0.80544676 -2.55524644

H -3.01921573 -1.13587324 3.70811356

H -1.21331173 1.71581976 -2.78644244

C -1.69297673 -0.24050024 -3.56012144

H -2.17258273 0.20720576 -4.44434144

H -0.63283073 -0.41237724 -3.80335544

C -0.54937573 -0.07874024 3.89762656

H -0.66791573 -0.48340824 4.91455656

H 0.51987427 0.13857576 3.75156356

C -2.36699773 -1.54270124 -3.20188144

C -1.69441573 -2.51181424 -2.43682044

C -3.69541573 -1.79654424 -3.58035144

C -2.33624173 -3.69060424 -2.04887244

H -0.65776573 -2.32634924 -2.13921344

C -4.34007873 -2.97639724 -3.19542744

H -4.22954473 -1.06120124 -4.18848644

C -3.66367773 -3.92571824 -2.42346744

H -1.79573073 -4.43069124 -1.45259044

H -5.37437173 -3.15457424 -3.50134444

H -4.16659773 -4.84770824 -2.12028044

C -1.36960273 1.18000576 3.74763456

C -1.13225773 2.07978576 2.69305256

C -2.42627173 1.45644376 4.63037956

C -1.93250773 3.21237376 2.52143956

H -0.31821873 1.88660976 1.99488956

C -3.22748273 2.59057076 4.46353656

H -2.62078973 0.77399576 5.46237756

C -2.98557573 3.47133676 3.40508556

H -1.72943273 3.89648076 1.69324656

H -4.04346973 2.78630076 5.16420956

H -3.61147773 4.35747676 3.27193856

C 2.73394827 -1.36693924 -0.81542244

H 3.20397127 -2.15597924 -1.41544244

H 1.87049527 -1.86263424 -0.30379244

C 3.72367627 -0.86173224 0.26405456

C 3.49756827 0.59222176 0.64782856

C 5.18815827 -0.77306824 -0.25921644

C 4.54574327 1.36157576 0.13203956

C 2.45440327 1.20951576 1.33067756

H 5.35422627 -1.33477024 -1.18861844

C 4.58346527 2.74957176 0.26443956

C 2.47682727 2.60500676 1.49068356

H 1.62350027 0.61766776 1.71840056

C 3.52781527 3.36165576 0.95553256

H 5.40923127 3.32919576 -0.15323744

H 1.66811427 3.10325276 2.02955956

H 3.53196427 4.44762276 1.08192556

O 5.48384227 0.62051876 -0.51500244

H 5.90035127 -1.13194124 0.50266256

C 3.63841427 -1.80105024 1.47756656

H 2.62044127 -1.79515924 1.89677356

H 3.87958127 -2.83689424 1.18680656

H 4.33957827 -1.49218424 2.26903856

C 2.80684727 -0.16086024 -2.42347544

H 3.83852827 0.10111876 -2.17466444

H 2.80690727 -0.87502224 -3.25731144

H 2.31740627 0.78654476 -2.75137444

*** D

Pd 1.93764400 -0.30875900 0.16468900

N -0.03727600 -1.05026100 0.97567800

C -1.00973500 -0.30725100 0.59073400

C -0.54881800 -1.90182300 2.06593700

C -2.07757500 -1.73174900 1.90788000

H -0.24517900 -2.94337600 1.89499900

H -2.53948300 -2.55572700 1.34642900

N 0.47515300 0.99623500 -0.74528200

C 0.40668600 1.95237500 -1.86941100

C -1.02430100 2.50858600 -1.71939400

H -1.05899300 3.45926200 -1.16945900

O -2.21316400 -0.52172900 1.08429400

C -0.73732100 0.77654000 -0.36782000

O -1.70091700 1.50531800 -0.88478800

H -1.58186500 2.59243100 -2.65865800

H -2.62016000 -1.56145600 2.84431500

H 1.15641900 2.74339500 -1.73634100

C 0.67493100 1.21432000 -3.19826900

H 0.55379300 1.95031300 -4.00781200

H 1.72540800 0.89903300 -3.19239500

C 0.02007200 -1.43118200 3.42405800

H -0.41583800 -2.08187400 4.19750800

H 1.10393900 -1.61489700 3.41659100

C -0.22025500 0.02028000 -3.42104700

C 0.09143600 -1.22155500 -2.84012800

C -1.40492700 0.13016600 -4.16611300

C -0.76487500 -2.31541900 -2.98500800

H 1.01719600 -1.33377600 -2.27227500

C -2.26386800 -0.96320000 -4.31451900

H -1.65492700 1.08419700 -4.63804700

C -1.94876700 -2.18856700 -3.71937000

H -0.50063600 -3.27251400 -2.52803200

H -3.18154800 -0.85725800 -4.89876900

H -2.61940400 -3.04394700 -3.83415900

C -0.26224000 0.02028700 3.72840700

C 0.57751200 1.03218000 3.23132900

C -1.39713300 0.39716000 4.46422400

C 0.28231200 2.38045800 3.44832300

H 1.47116900 0.75846600 2.66396300

C -1.69497600 1.74541400 4.68444200

H -2.05300800 -0.37597800 4.87317800

C -0.85876200 2.74194700 4.17241000

H 0.94888300 3.15156000 3.05315900

H -2.58306800 2.01750700 5.26062200

H -1.09092600 3.79616500 4.34342000

C 3.55833900 0.69577900 -0.64444400

H 3.99782300 1.24636400 0.20264800

H 3.05601800 1.40690400 -1.31292200

C 4.65775600 -0.05506600 -1.41834100

C 4.07499000 -1.00984800 -2.45164300

C 5.41923600 -1.08032800 -0.54696800

C 4.09902200 -2.29999100 -1.90578400

C 3.55747600 -0.80750600 -3.72564200

H 5.46511400 -0.81952700 0.51787400

C 3.55307000 -3.39695500 -2.56662900

C 3.02229400 -1.90248900 -4.42440200

H 3.56876500 0.18574000 -4.17940500

C 3.00755700 -3.17445000 -3.84109500

H 3.57519400 -4.39207600 -2.11799000

H 2.60664000 -1.75818800 -5.42397100

H 2.57716300 -4.01576900 -4.39002800

O 4.71967600 -2.34209600 -0.69159200

H 6.44741700 -1.21859100 -0.92199900

C 5.61305900 0.98871100 -2.01381900

H 5.06968000 1.69069400 -2.66458500

H 6.09779200 1.57378000 -1.21601300

H 6.39753700 0.50082400 -2.61318500

C 3.03781900 -1.40534700 1.22825100

O 3.52444600 -2.05129100 2.02892400

*** TS3

Pd 1.86808000 -0.17261000 0.14496700

N 0.02056000 -0.92473900 0.89884100

C -1.02469600 -0.28204100 0.51859800

C -0.38239300 -1.82645600 1.99244300

C -1.92528200 -1.78273900 1.88019200

H -0.00190400 -2.83688200 1.79059700

H -2.33550100 -2.65305600 1.35003000

N 0.31968000 1.06604100 -0.89887000

C 0.15622000 1.97149900 -2.05168000

C -1.30902200 2.43274700 -1.89385900

H -1.40070100 3.40467200 -1.38898200

O -2.18945200 -0.60571000 1.03792500

C -0.86283200 0.78767200 -0.48107700

O -1.89391100 1.42493400 -0.99838400

H -1.88986700 2.43148300 -2.82298100

H -2.45114600 -1.63726600 2.83011500

H 0.85369600 2.81583200 -1.96930500

C 0.44593200 1.19753300 -3.35627900

H 0.29761400 1.89722300 -4.19287700

H 1.50683800 0.91497100 -3.34305000

C 0.19680300 -1.33696700 3.33693000

H -0.15130900 -2.03725500 4.11149700

H 1.29088600 -1.42970900 3.27824200

C -0.41484100 -0.02985000 -3.52740700

C -0.09703600 -1.22239100 -2.85340400

C -1.57713900 0.00251600 -4.31414200

C -0.92578200 -2.34245000 -2.94851000

H 0.81225900 -1.27434300 -2.25182700

C -2.40747500 -1.11825300 -4.41467700

H -1.83222800 0.91711600 -4.85642400

C -2.08699900 -2.29286200 -3.72722100

H -0.65762300 -3.25973500 -2.41795000

H -3.30756300 -1.07348500 -5.03320400

H -2.73594300 -3.16902400 -3.80358400

C -0.19266200 0.07891900 3.68695400

C 0.55888100 1.16740900 3.21147600

C -1.34115100 0.34475400 4.45012900

C 0.16508500 2.48168100 3.47603600

H 1.46266600 0.97959000 2.62639900

C -1.73829200 1.65851600 4.71694900

H -1.92809500 -0.48877500 4.84484500

C -0.98887200 2.73181600 4.22619600

H 0.76488200 3.31394600 3.09839200

H -2.63526600 1.84364800 5.31349800

H -1.29799500 3.75929200 4.43416700

C 4.00643900 0.48914400 -0.23238500

H 4.55241700 0.98526900 0.58072500

H 3.45124800 1.27175400 -0.77065500

C 4.94398000 -0.26379200 -1.19881500

C 4.16055700 -0.99011100 -2.28296400

C 5.63569400 -1.48917900 -0.55578600

C 4.05192500 -2.33444800 -1.90185400

C 3.60359400 -0.55777200 -3.48031000

H 5.85189900 -1.39248500 0.51338400

C 3.34786600 -3.26473200 -2.66203600

C 2.91293900 -1.48446900 -4.27865300

H 3.71014800 0.48152000 -3.79979000

C 2.77792400 -2.81394500 -3.86285700

H 3.27160700 -4.30705700 -2.34645400

H 2.46751500 -1.16327400 -5.22239200

H 2.22514100 -3.52018900 -4.48714600

O 4.72989900 -2.60225300 -0.74935600

H 6.57265600 -1.71215000 -1.09392000

C 5.96468800 0.74531300 -1.73702000

H 5.45873700 1.59095300 -2.22749600

H 6.58277400 1.14840600 -0.91915200

H 6.62917300 0.26701900 -2.47276500

C 3.28484700 -0.79479200 1.10046200

O 3.90572200 -1.26536300 1.95424300

*** Int. 2

Pd 1.95565000 0.94573300 0.84066700

N 0.02909500 0.06445400 1.13865000

C -0.96316300 0.65412000 0.57505200

C -0.52032700 -0.88100900 2.12501200

C -2.00089800 -0.95594000 1.69385800

H -0.01643100 -1.84884000 2.01706800

H -2.21571300 -1.83281700 1.06708800

N 0.46734800 2.26466200 -0.45333700

C 0.45653600 3.10248000 -1.67076100

C -1.05625000 3.26459600 -1.94680000

H -1.45468300 4.21844200 -1.57215500

O -2.18434400 0.23206700 0.85045500

C -0.71088900 1.76758200 -0.35183900

O -1.67111300 2.19593500 -1.15923100

H -1.34556000 3.12386200 -2.99492000

H -2.72026600 -0.89524800 2.51812200

H 0.91568500 4.07644000 -1.46304200

C 1.26293700 2.39961100 -2.79703500

H 0.91092300 2.80796300 -3.75764000

H 2.31816200 2.67964500 -2.68846700

C -0.28262700 -0.35200100 3.55709100

H -0.68046200 -1.10720200 4.25196900

H 0.80531900 -0.30077000 3.71377200

C 1.14790100 0.89356700 -2.76119700

C 2.20573400 0.12869900 -2.24239600

C -0.03336400 0.23270300 -3.13279800

C 2.07986500 -1.25338300 -2.07294800

H 3.14058100 0.62582400 -1.97868800

C -0.16625200 -1.14819400 -2.95592400

H -0.86445500 0.80110300 -3.55696400

C 0.88656700 -1.89499800 -2.41657100

H 2.91407900 -1.82787600 -1.66321400

H -1.09722500 -1.64368200 -3.24331100

H 0.78028600 -2.97343600 -2.27529600

C -0.91461300 0.99313900 3.82128300

C -0.26546100 2.17788900 3.43358400

C -2.18628700 1.09195000 4.40831400

C -0.87434500 3.42249200 3.61168000

H 0.72636800 2.11942000 2.98179200

C -2.79799300 2.33612400 4.59105200

H -2.70073700 0.18315700 4.73228800

C -2.14613900 3.50568900 4.18830700

H -0.35142500 4.33051000 3.30020300

H -3.78766000 2.39153700 5.05177000

H -2.62428800 4.47833600 4.32943200

C 2.93278400 -0.51376300 1.69602300

O 2.48379500 -1.62523600 1.72921800

C 4.29449000 -0.13199900 2.25754000

H 4.09729000 0.23988900 3.27817800

H 4.91656900 -1.03656000 2.34585600

C 4.96559700 0.96775200 1.44075900

C 3.99175300 2.09187400 1.10498900

C 5.31779800 0.51561200 0.00283000

C 4.29315200 2.51976500 -0.21272100

C 3.23569000 2.93109200 1.95700800

H 4.62817200 -0.27035800 -0.34981100

H 6.35302300 0.17273700 -0.11563500

C 3.82502500 3.72820400 -0.71957100

C 2.73889000 4.13737400 1.44370700

H 3.09521000 2.66930600 3.00763900

C 3.04391600 4.52340100 0.13105300

H 4.08301800 4.05084800 -1.72882800

H 2.13390600 4.78864700 2.07675400

H 2.67072800 5.48034400 -0.24156100

C 6.19900000 1.52114700 2.16777800

H 5.91074300 1.97932100 3.12595600

H 6.91870900 0.71247900 2.37034400

H 6.70304500 2.28621500 1.55763600

O 5.13034200 1.67837000 -0.84773000

*** E

Pd 2.11760500 0.68618800 0.14286300

N 0.40741900 0.09941400 1.17045800

C -0.71685700 0.47544800 0.66347200

C 0.09679300 -0.66946100 2.38992700

C -1.39729800 -0.98989300 2.18425800

H 0.70761500 -1.58085000 2.41685100

H -1.56176400 -1.98310600 1.74243500

N 0.40120200 1.91777100 -0.85249900

C 0.12067400 2.72060900 -2.05312200

C -1.42762600 2.80103100 -2.04680400

H -1.79617800 3.77203500 -1.68719400

O -1.83178400 0.00255300 1.19455000

C -0.71558200 1.43371600 -0.45165600

O -1.83576000 1.79177200 -1.06614600

H -1.90455900 2.55495800 -3.00231200

H -2.01768500 -0.86464300 3.07870400

H 0.56099600 3.72003400 -1.92816000

C 0.75922500 2.06630900 -3.29957800

H 0.42833600 2.64369400 -4.17739200

H 1.84752600 2.19090900 -3.20808600

C 0.40230900 0.18621300 3.64006400

H 0.28070000 -0.46401100 4.51940600

H 1.46157600 0.47720200 3.58885200

C 0.43027000 0.60040200 -3.45866100

C 1.30381800 -0.37253300 -2.94236700

C -0.76206200 0.17488500 -4.06609900

C 0.98146100 -1.73065600 -3.01018400

H 2.23811100 -0.05021200 -2.47605000

C -1.08579300 -1.18412400 -4.13632900

H -1.44393200 0.91328700 -4.49562700

C -0.21759300 -2.14165100 -3.60224900

H 1.67267300 -2.46919900 -2.59528500

H -2.01890200 -1.49555800 -4.61330600

H -0.47085200 -3.20377800 -3.65498200

C -0.47616500 1.40729300 3.77264500

C -0.26981000 2.53986100 2.96494100

C -1.55541900 1.41790500 4.67108600

C -1.12342700 3.64314600 3.04584000

H 0.56322500 2.55780000 2.26301300

C -2.40931500 2.52198000 4.75650400

H -1.72613200 0.54944600 5.31317000

C -2.19829400 3.63773200 3.94067600

H -0.94484700 4.51104000 2.40559800

H -3.24285000 2.50990400 5.46362100

H -2.86626200 4.50048800 4.00457700

C 3.29367000 -0.61349000 0.99011900

O 3.30954200 -1.75528700 0.59194300

C 4.15642800 -0.18410200 2.17631400

H 3.46602500 0.00981800 3.01369400

H 4.76874700 -1.05797800 2.45243200

C 5.06262900 1.04553400 1.99631700

C 4.33775200 2.35620300 1.73752900

C 5.93755800 0.96890500 0.71476200

C 5.01630300 3.02878500 0.71242100

C 3.26118700 2.95875700 2.37467000

H 5.44040200 0.38253000 -0.07130600

H 6.94695800 0.57727400 0.90209200

C 4.62727600 4.29516600 0.28179200

C 2.84472200 4.23375100 1.95237500

H 2.73774800 2.44592500 3.18424800

C 3.52369600 4.88709800 0.91706500

H 5.16026000 4.79894900 -0.52715100

H 1.98946800 4.71336400 2.43327200

H 3.19142200 5.87712300 0.59319800

C 5.94178400 1.19384100 3.25285100

H 5.31942500 1.37486000 4.14328700

H 6.53657300 0.28210500 3.42469200

H 6.63423400 2.04280200 3.14252000

O 6.06411900 2.32351100 0.22283200

F 3.59268000 1.16242500 -1.16825200

*** F

Pd 2.32236700 0.66382400 -0.11621600

N 0.62460300 -0.03555000 1.17166300

C -0.52417600 0.26667300 0.68110400

C 0.35266800 -0.71098700 2.45616200

C -1.12144500 -1.12733000 2.29421900

H 1.01406800 -1.57926500 2.57371400

H -1.23446800 -2.15055800 1.90618900

N 0.47127700 1.62848300 -0.99368200

C 0.07697300 2.36250900 -2.20882600

C -1.44743600 2.52372800 -2.01348300

H -1.72041400 3.50679100 -1.60306700

O -1.61671100 -0.21125800 1.26505900

C -0.60732700 1.16810900 -0.47521300

O -1.77593200 1.52169900 -0.99766800

H -2.04753200 2.30988800 -2.90532300

H -1.73680400 -0.99383400 3.19126700

H 0.58285500 3.33743600 -2.22995300

C 0.48267000 1.56994400 -3.47313800

H 0.19309600 2.17835300 -4.34381100

H 1.57825600 1.48839300 -3.47424000

C 0.59447400 0.27442300 3.62598200

H 0.44262200 -0.28610000 4.56098800

H 1.64894600 0.58104800 3.59235200

C -0.14814400 0.20120900 -3.55934400

C 0.39299200 -0.88503200 -2.84914200

C -1.31864000 -0.00796700 -4.30683900

C -0.22716700 -2.13700100 -2.87328400

H 1.30475600 -0.74172200 -2.26305800

C -1.94010000 -1.26036100 -4.33503100

H -1.74531200 0.82101100 -4.87815200

C -1.39876000 -2.32860300 -3.61343800

H 0.20999600 -2.96750700 -2.31248900

H -2.84997300 -1.40182800 -4.92424600

H -1.88398800 -3.30788000 -3.63305700

C -0.29747600 1.49196100 3.59647600

C -0.04104000 2.54885900 2.70464500

C -1.43091200 1.57614400 4.42079800

C -0.90099900 3.64749900 2.62903200

H 0.83735400 2.50810400 2.06044800

C -2.29075300 2.67667000 4.35078800

H -1.64000000 0.76895900 5.12824500

C -2.03151500 3.71430500 3.45010600

H -0.68338300 4.45552800 1.92556000

H -3.16704000 2.72272000 5.00269000

H -2.70455400 4.57357600 3.39235400

C 3.79698400 -0.42206400 0.61058400

O 4.38827300 -1.27899400 -0.02728000

C 4.14270100 -0.27037200 2.09813400

H 3.22727500 -0.10380300 2.67920400

H 4.59387000 -1.22270600 2.42523900

C 5.13278600 0.87644200 2.43735200

C 4.57096600 2.24156700 2.08054500

C 6.39979100 0.84917100 1.53347100

C 5.28866400 2.75345100 0.99460800

C 3.51034800 2.96308900 2.61551600

H 6.50987200 -0.09008500 0.97595100

H 7.31266400 1.02514500 2.12614800

C 4.96852400 3.97527800 0.40667300

C 3.16851500 4.19932400 2.04245400

H 2.94149900 2.57018200 3.46099000

C 3.89024300 4.69133400 0.94779400

H 5.53411600 4.34994100 -0.44856200

H 2.33452000 4.77508100 2.44966500

H 3.61458300 5.65284600 0.50644900

C 5.50578800 0.77146900 3.92179700

H 4.60429100 0.80746900 4.55345300

H 6.02477000 -0.17873500 4.12757400

H 6.16753000 1.59978900 4.21923500

O 6.27924100 1.92667000 0.57493100

C 3.62906200 1.32589600 -1.55324700

H 3.33300900 2.34695800 -1.85327700

H 4.68668200 1.33663000 -1.25976500

H 3.52026800 0.65671200 -2.42622700

*** TS 4

Pd 2.19597000 0.41253400 0.21162000

N 0.32766700 -0.01683100 1.22992800

C -0.75862400 0.41691400 0.70088900

C -0.04710300 -0.74574500 2.45216500

C -1.57293000 -0.91906000 2.27960300

H 0.46994800 -1.71491000 2.46776500

H -1.84686600 -1.91732200 1.90857000

N 0.43901500 1.75962100 -0.86124900

C 0.18106800 2.52614600 -2.08854800

C -1.36061300 2.67716600 -2.08553300

H -1.68756700 3.67456200 -1.75735500

O -1.91953000 0.04706200 1.23646800

C -0.69413200 1.33306100 -0.44682500

O -1.80500200 1.71439500 -1.07997800

H -1.84555200 2.42147900 -3.03567100

H -2.16150500 -0.67649800 3.17260700

H 0.67580200 3.50558300 -2.01605600

C 0.76764200 1.78378900 -3.31119700

H 0.62406900 2.42919400 -4.19144900

H 1.85143000 1.68868000 -3.14239400

C 0.38606900 0.06077300 3.69603300

H 0.28071100 -0.59466600 4.57348900

H 1.45912800 0.27922300 3.57575000

C 0.15367800 0.42663400 -3.55405600

C 0.55737300 -0.68998300 -2.80126500

C -0.86784600 0.25661200 -4.50263400

C -0.05198400 -1.93419100 -2.98158400

H 1.34928400 -0.57565800 -2.05471500

C -1.47777300 -0.98835800 -4.68798800

H -1.18543100 1.11053400 -5.10759600

C -1.07452700 -2.08785500 -3.92419900

H 0.27662800 -2.78790300 -2.38267700

H -2.26997700 -1.09917700 -5.43314900

H -1.55091200 -3.06131800 -4.06676100

C -0.39860300 1.33275400 3.91514600

C -0.23974500 2.44759400 3.07222900

C -1.34395700 1.41351700 4.95046500

C -1.00373700 3.60252200 3.25821000

H 0.48479800 2.40929200 2.26039700

C -2.10712500 2.56961800 5.14213800

H -1.47980600 0.55767100 5.61739700

C -1.94115200 3.66865000 4.29413300

H -0.86411700 4.45581600 2.58919200

H -2.83450200 2.61070900 5.95718600

H -2.53728600 4.57286800 4.44125500

C 3.71324900 -0.74168900 0.75702100

O 3.64734200 -1.96552600 0.60561000

C 4.42643600 -0.25370000 2.04299800

H 3.62879200 -0.27265200 2.80597200

H 5.13450900 -1.05252200 2.32485300

C 5.16251400 1.10173700 2.16810200

C 4.41410600 2.28301000 1.58113300

C 6.48264900 1.15379500 1.33502300

C 5.16644600 2.82858700 0.53639700

C 3.17859500 2.83051800 1.90752000

H 6.70114200 0.20542200 0.82271800

H 7.34868400 1.41177800 1.96473500

C 4.70683300 3.90536600 -0.22091500

C 2.69542500 3.92121300 1.16470600

H 2.59599200 2.41985100 2.73310900

C 3.45220600 4.44190900 0.10819400

H 5.30488200 4.30743200 -1.04115300

H 1.72369600 4.35641700 1.40755400

H 3.06603900 5.28586300 -0.46957600

C 5.46731400 1.33357500 3.65909700

H 4.53495300 1.38724300 4.24265700

H 6.07593500 0.50926900 4.06588500

H 6.01881900 2.27535100 3.80496000

O 6.34368100 2.18782900 0.33246000

C 4.11380300 0.14764100 -0.85004100

H 3.91736400 1.17722700 -1.20121200

H 5.18382500 0.12488700 -0.61520600

H 3.91719800 -0.58258200 -1.64581000

*** 1a

C -1.15524400 0.48901200 0.79135700

C -1.90463800 1.55486500 0.27095900

C -1.36124400 2.83605300 0.25386900

C -0.06573000 3.03500400 0.76097900

C 0.67149300 1.95638400 1.26386500

C 0.13521400 0.66006400 1.28841700

C -3.20083500 -0.38932100 0.45619600

H -1.93113600 3.66914700 -0.15948800

H 0.36960300 4.03724200 0.75907400

H 1.67955100 2.12330100 1.65279400

H 0.69729500 -0.18629200 1.68852900

H -3.75255100 -0.33843000 1.41127500

H -3.61312200 -1.20570800 -0.15221800

O -1.81998400 -0.69563900 0.74940000

C -3.22011400 0.99053100 -0.25614200

C -4.46698700 1.78857900 0.13205300

H -4.47902100 2.76187800 -0.37388700

H -5.37776900 1.23964800 -0.16056800

H -4.50070700 1.95243200 1.22037300

C -3.12811000 0.75853800 -1.78193500

H -4.06485300 0.28794700 -2.13722100

H -2.33431600 0.02517300 -2.00015700

C -2.90681800 1.96672200 -2.67471800

O -3.13746700 3.10927500 -2.32557100

C -2.37955500 1.65634200 -4.05550900

H -1.31619600 1.37183900 -3.96834500

H -2.90569500 0.79642400 -4.49984300

H -2.46488400 2.53746000 -4.70598800

*** 2a

C -1.11660400 0.47999900 0.98625900

C -1.80137600 1.47649200 0.27784800

C -1.22302200 2.72971200 0.10931900

C 0.04571500 2.97623200 0.66249400

C 0.71822200 1.96669900 1.36170600

C 0.14543700 0.69726800 1.53646100

C -3.15553600 -0.41203700 0.60282200

H -1.74594100 3.51337300 -0.44553300

H 0.50968600 3.95845700 0.54481000

H 1.70515600 2.16820200 1.78676900

H 0.66066300 -0.09336100 2.08585300

H -3.79464300 -0.26639600 1.49111800

H -3.51029700 -1.29406200 0.05332600

O -1.80968100 -0.68485200 1.05993800

C -3.09605500 0.88140900 -0.25543400

C -4.31573500 1.77532000 -0.01614000

H -4.27725600 2.67244300 -0.65467200

H -5.25262300 1.24273800 -0.24209200

H -4.35871000 2.10438000 1.03358700

C -2.90740200 0.55250900 -1.76013600

H -2.60950900 1.48255900 -2.27354600

H -2.04908100 -0.13525600 -1.85064100

C -4.12310200 -0.04956900 -2.46125500

H -3.86932800 -0.33113900 -3.49604200

H -4.48310600 -0.95929900 -1.95288000

H -4.96332300 0.66045000 -2.51062500

*** SnMe4

Sn -0.03511300 0.42658400 -0.00013700

C 0.04975400 2.21370700 1.23305500

H 0.96471000 2.20870300 1.84613700

H 0.05345800 3.11359300 0.59805100

H -0.82457100 2.25525200 1.90155500

C -1.83977200 0.46552500 -1.20987400

H -1.89077400 -0.43335900 -1.84427200

H -2.73044500 0.49353300 -0.56261300

H -1.84370100 1.35669600 -1.85706400

C -0.05543000 -1.33211800 1.27603100

H 0.85274600 -1.35978300 1.89853700

H -0.93699100 -1.31229900 1.93595500

H -0.09243600 -2.24601600 0.66246400

C 1.70544600 0.35921200 -1.29926600

H 2.62780700 0.34447900 -0.69753300

H 1.67668200 -0.54593700 -1.92609100

H 1.72422400 1.24397500 -1.95493500

*** SnMe3F

Sn -0.01465400 0.03792400 0.29167000

C -1.86057200 0.78532000 -0.52299600

H -2.02712500 0.33104500 -1.51198600

H -2.69569200 0.52046500 0.14252100

H -1.80415600 1.87871300 -0.63169600

C -0.03541100 -2.04314400 0.83718900

H 0.88579600 -2.29452800 1.38370400

H -0.91168300 -2.25471600 1.46792800

H -0.09167500 -2.65313200 -0.07772900

C 1.76328400 0.69738300 -0.72447100

H 2.65416200 0.36235000 -0.17264600

H 1.77960900 0.26459400 -1.73674800

H 1.76355400 1.79501400 -0.80012700

F 0.10505200 0.96474100 2.05538700

*** BOx+

C -0.87459500 0.53899900 0.05460000

C 0.37734400 0.09532800 -0.29192100

C 1.48708600 0.98837700 -0.17547800

C 1.33935600 2.33945400 0.27900000

C 0.03253800 2.73244000 0.63044800

C -1.03827200 1.86308600 0.52183000

H -1.73206700 -0.13004900 -0.02813600

H 0.55773800 -0.91972700 -0.64915100

H -0.10111300 3.75596800 0.98391800

H -2.03412600 2.21465500 0.80022100

O 2.28140000 3.25180100 0.41460400

C 3.64293400 3.09310700 -0.05853900

H 4.16030200 2.35570700 0.57205900

H 3.60305400 2.74440200 -1.10488200

C 4.31741000 4.43446300 0.00870900

C 3.67951000 5.53494700 -0.79096200

H 3.59720200 5.24756000 -1.85386500

H 2.65384300 5.73242700 -0.43719600

H 4.25605600 6.46839500 -0.72252600

C 5.44138600 4.58040300 0.72207000

H 5.97713500 5.53414400 0.74614500

H 5.86644300 3.75077800 1.29495200

N 2.68952000 0.44645600 -0.49224200

N 3.61663900 -0.10908500 -0.76126500

8.1. Computed normal modes of transition states.

Table S4: Frequencies and intensities for transition state normal modes. The 'i' stands for the imaginary unity.

TS	#	Frequency (cm^{-1})	Intensity ($km\ mol^{-1}$)
**ReTS1**	1	241.0i	91.4
	2	16.5	1.3
	3	20.6	0.3
	4	23.0	0.1
	5	34.7	0.3
	6	41.1	0.7
	7	45.9	0.1
	8	56.6	0.4
	9	67.9	0.2
	10	80.3	1.0
	11	89.0	0.7
	12	101.5	0.9
	13	110.0	2.0
	14	123.6	1.2

	15	132.8	4.7
	16	139.4	0.6
	17	144.9	2.9
	18	161.1	2.6
	19	169.6	2.1
	20	191.2	12.7
	21	202.7	10.2
	22	215.7	6.9
	23	228.5	2.9
	24	235.5	4.4
	25	267.1	3.6
	26	275.6	1.7
	27	279.7	5.2
	28	285.2	2.1
	29	306.6	2.3
	30	323.6	2.3
	31	333.7	3.8
	32	366.5	2.9
	33	385.6	2.1
	34	391.9	14.4
	35	400.8	2.6
	36	420.1	2.5
	37	426.0	1.0
	38	430.9	4.2
	39	435.1	3.2
	40	444.8	7.5
	41	477.1	6.1

	42	489.6	5.9
	43	494.2	1.5
	44	505.0	11.2
	45	507.4	1.3
	46	530.7	19.6
	47	543.3	8.5
	48	562.8	3.3
	49	578.5	18.6
	50	621.7	9.9
	51	634.8	0.3
	52	635.2	2.0
	53	641.2	75.5
	54	642.3	8.0
	55	667.7	5.2
	56	726.0	64.4
	57	732.9	63.6
	58	736.9	20.2
	59	739.9	10.0
	60	761.7	1.2
	61	769.3	0.4
	62	773.7	15.1
	63	774.4	71.1
	64	777.0	35.5
	65	779.6	3.8
	66	819.1	9.0
	67	848.9	2.4
	68	854.8	3.1

	69	857.0	5.0
	70	862.8	49.9
	71	864.8	19.6
	72	870.4	1.8
	73	871.9	0.5
	74	873.8	3.2
	75	880.2	0.7
	76	900.4	1.3
	77	939.8	69.4
	78	943.1	26.8
	79	946.5	15.4
	80	951.0	9.5
	81	956.2	106.3
	82	958.6	4.1
	83	966.4	35.1
	84	971.5	21.7
	85	999.8	19.5
	86	1009.7	2.6
	87	1010.2	0.4
	88	1011.3	0.9
	89	1015.7	1.6
	90	1015.9	0.2
	91	1017.4	5.2
	92	1018.1	0.4
	93	1025.3	22.7
	94	1030.4	58.3
	95	1033.4	0.3

	96	1036.5	1.2
	97	1037.8	6.0
	98	1045.5	7.1
	99	1049.5	19.8
	100	1049.8	2.3
	101	1050.4	6.8
	102	1052.5	29.1
	103	1054.3	55.6
	104	1063.0	107.3
	105	1076.9	8.5
	106	1079.6	15.7
	107	1100.8	25.5
	108	1103.1	24.9
	109	1120.3	28.1
	110	1152.3	4.5
	111	1155.0	0.0
	112	1156.4	0.0
	113	1179.6	0.1
	114	1182.3	0.6
	115	1192.3	7.2
	116	1195.5	13.8
	117	1209.2	5.1
	118	1215.3	22.0
	119	1217.8	13.3
	120	1227.7	3.5
	121	1227.9	0.8
	122	1253.2	19.0

	123	1268.9	75.0
	124	1272.0	137.6
	125	1279.0	34.7
	126	1284.2	48.9
	127	1291.2	92.1
	128	1304.0	18.7
	129	1307.1	67.3
	130	1335.1	60.6
	131	1340.3	14.8
	132	1343.6	5.0
	133	1350.4	25.5
	134	1359.7	32.3
	135	1367.8	0.7
	136	1368.2	2.5
	137	1370.1	20.1
	138	1376.7	0.2
	139	1387.9	9.2
	140	1403.4	112.4
	141	1414.5	4.0
	142	1441.1	13.9
	143	1446.6	4.4
	144	1446.9	6.8
	145	1447.3	10.4
	146	1458.6	19.7
	147	1470.7	9.7
	148	1472.9	18.6
	149	1478.3	7.2

	150	1478.8	5.5
	151	1481.7	39.4
	152	1493.0	162.5
	153	1501.9	30.8
	154	1524.6	18.4
	155	1525.5	19.1
	156	1551.6	598.0
	157	1615.6	12.1
	158	1622.2	75.5
	159	1636.9	0.6
	160	1637.0	0.8
	161	1661.1	4.6
	162	1661.4	3.2
	163	1698.5	156.0
	164	1707.6	18.0
	165	3028.4	14.2
	166	3051.0	56.0
	167	3071.8	24.5
	168	3075.7	21.0
	169	3094.6	21.4
	170	3095.9	18.6
	171	3108.8	21.4
	172	3118.2	12.2
	173	3120.3	14.7
	174	3121.3	9.3
	175	3123.4	16.8
	176	3129.9	13.5

	177	3153.7	2.3
	178	3163.5	9.1
	179	3172.8	4.0
	180	3172.8	4.8
	181	3176.4	0.5
	182	3176.4	1.0
	183	3179.0	1.2
	184	3182.1	3.4
	185	3182.1	3.2
	186	3184.2	6.6
	187	3185.3	14.3
	188	3187.3	1.5
	189	3190.5	26.4
	190	3191.3	30.3
	191	3197.4	9.3
	192	3199.4	13.5
	193	3200.0	14.1
	194	3205.0	12.5
	195	3224.0	2.1
**SiTS1**	1	266.5i	88.7
	2	16.8	1.0
	3	19.4	0.2
	4	24.4	0.2
	5	32.4	0.4
	6	34.0	0.2
	7	52.8	0.6
	8	55.0	0.2

	9	66.9	0.5
	10	78.8	1.1
	11	83.9	0.7
	12	103.4	3.0
	13	109.0	1.4
	14	111.6	0.3
	15	119.0	1.1
	16	140.4	0.8
	17	149.8	4.4
	18	154.9	4.7
	19	172.4	2.4
	20	180.7	0.3
	21	199.9	13.6
	22	226.1	12.6
	23	234.3	4.6
	24	242.9	5.7
	25	266.0	1.7
	26	271.3	2.4
	27	275.2	4.1
	28	279.3	0.6
	29	296.9	1.4
	30	314.4	0.1
	31	332.9	3.2
	32	376.3	1.0
	33	382.6	3.4
	34	386.7	13.3
	35	392.1	4.4

	36	417.2	1.8
	37	423.0	1.1
	38	427.1	3.2
	39	435.0	2.4
	40	451.6	6.9
	41	475.4	6.4
	42	490.0	2.5
	43	502.4	2.3
	44	506.1	5.0
	45	509.4	7.0
	46	538.5	8.1
	47	548.9	19.1
	48	563.7	5.8
	49	578.0	23.1
	50	628.7	7.5
	51	635.2	0.4
	52	636.0	1.6
	53	637.1	4.2
	54	645.8	69.3
	55	672.2	4.1
	56	724.8	52.2
	57	731.8	70.6
	58	734.7	4.9
	59	738.9	28.2
	60	753.3	5.7
	61	767.9	2.0
	62	770.6	72.4

	63	776.6	16.6
	64	777.8	31.5
	65	781.1	0.9
	66	820.2	8.4
	67	848.5	3.5
	68	852.8	4.9
	69	855.4	8.8
	70	862.6	29.3
	71	864.1	20.3
	72	869.9	1.3
	73	871.0	2.3
	74	872.2	0.8
	75	877.7	1.6
	76	898.1	3.5
	77	936.1	41.8
	78	942.4	22.0
	79	947.8	19.4
	80	954.4	128.0
	81	955.6	31.0
	82	958.6	6.4
	83	967.4	3.5
	84	975.1	18.1
	85	1003.1	3.8
	86	1010.2	18.6
	87	1011.1	2.8
	88	1012.0	6.8
	89	1014.9	0.9

	90	1016.5	1.1
	91	1018.1	0.5
	92	1019.8	19.5
	93	1030.7	15.2
	94	1032.3	15.7
	95	1034.2	18.4
	96	1035.4	6.5
	97	1038.2	5.9
	98	1048.5	4.4
	99	1049.3	13.2
	100	1051.6	7.7
	101	1053.9	8.6
	102	1055.6	45.3
	103	1057.1	35.8
	104	1070.3	119.8
	105	1085.5	16.8
	106	1090.3	19.8
	107	1103.8	24.2
	108	1107.6	24.2
	109	1120.2	28.7
	110	1152.8	3.2
	111	1157.8	0.1
	112	1158.2	0.0
	113	1183.8	0.4
	114	1189.6	2.6
	115	1193.5	5.3
	116	1198.6	13.6

	117	1212.5	5.9
	118	1216.2	11.9
	119	1217.9	11.6
	120	1228.6	2.9
	121	1231.4	4.2
	122	1258.6	5.1
	123	1269.3	76.4
	124	1276.8	141.6
	125	1281.9	54.8
	126	1287.6	61.2
	127	1297.4	71.1
	128	1309.0	6.6
	129	1311.6	54.1
	130	1328.8	54.8
	131	1343.6	6.8
	132	1350.6	6.8
	133	1351.0	22.3
	134	1360.1	39.2
	135	1367.5	2.5
	136	1368.1	0.2
	137	1375.2	24.1
	138	1381.1	0.2
	139	1386.6	8.0
	140	1404.7	116.9
	141	1419.7	7.9
	142	1441.4	15.4
	143	1443.7	3.2

	144	1444.5	6.9
	145	1446.1	4.8
	146	1457.6	18.9
	147	1468.5	3.0
	148	1474.7	27.3
	149	1479.3	1.5
	150	1480.7	9.3
	151	1484.9	41.9
	152	1491.5	62.7
	153	1495.8	131.7
	154	1526.4	17.7
	155	1529.4	15.4
	156	1551.1	614.7
	157	1616.2	20.1
	158	1624.0	68.5
	159	1637.3	0.7
	160	1638.8	0.9
	161	1661.6	3.4
	162	1662.9	4.8
	163	1699.6	173.6
	164	1712.8	18.9
	165	3029.3	17.4
	166	3052.5	52.1
	167	3063.9	22.3
	168	3070.9	21.1
	169	3098.1	9.4
	170	3100.3	19.0

	171	3102.6	14.7
	172	3111.8	23.1
	173	3114.5	24.9
	174	3115.7	14.7
	175	3119.9	11.1
	176	3130.4	11.1
	177	3133.9	2.7
	178	3159.0	10.5
	179	3169.6	4.0
	180	3174.6	3.3
	181	3174.8	2.6
	182	3178.1	0.2
	183	3180.0	6.6
	184	3182.1	9.0
	185	3183.7	1.6
	186	3183.9	3.9
	187	3187.4	4.0
	188	3190.1	28.2
	189	3190.5	26.9
	190	3196.3	4.8
	191	3197.9	10.1
	192	3199.1	17.6
	193	3199.5	14.7
	194	3205.5	11.2
	195	3223.3	5.2
**TS 2**	1	466.9i	191.3
	2	9.4	0.4

	3	14.3	0.3
	4	16.1	0.4
	5	19.6	0.5
	6	27.2	0.1
	7	33.1	0.4
	8	36.0	0.2
	9	45.6	0.4
	10	53.1	0.4
	11	62.5	0.2
	12	69.7	0.2
	13	73.4	0.3
	14	75.6	0.4
	15	93.3	0.2
	16	100.8	2.5
	17	112.3	1.0
	18	125.1	4.8
	19	137.0	1.4
	20	148.0	1.0
	21	163.8	3.8
	22	176.0	1.6
	23	196.0	2.5
	24	212.7	6.4
	25	218.9	2.4
	26	224.9	1.8
	27	241.7	3.9
	28	267.1	1.4
	29	268.4	3.0

	30	269.7	3.0
	31	295.7	4.6
	32	317.7	0.9
	33	325.0	0.2
	34	343.5	11.3
	35	380.8	1.3
	36	388.7	4.6
	37	400.0	0.1
	38	409.5	0.9
	39	420.5	5.2
	40	425.1	0.2
	41	429.4	0.0
	42	438.4	6.0
	43	456.4	5.7
	44	470.1	5.1
	45	490.8	4.9
	46	492.5	7.8
	47	496.5	4.0
	48	503.1	8.7
	49	508.7	0.7
	50	551.6	0.5
	51	578.7	2.0
	52	583.9	10.5
	53	587.0	1.7
	54	625.0	10.5
	55	634.3	0.5
	56	635.1	0.4

	57	645.3	83.3
	58	659.7	12.6
	59	668.7	5.1
	60	725.9	65.8
	61	729.0	66.7
	62	738.6	1.0
	63	745.4	3.1
	64	760.6	1.0
	65	765.3	40.8
	66	767.3	0.5
	67	776.5	66.2
	68	776.7	23.7
	69	778.9	5.1
	70	832.6	11.0
	71	847.8	2.4
	72	852.1	20.7
	73	852.7	18.8
	74	855.9	4.2
	75	857.9	2.1
	76	871.0	0.6
	77	874.0	1.4
	78	875.5	0.5
	79	881.1	1.5
	80	885.7	60.9
	81	897.7	2.2
	82	904.9	6.4
	83	937.8	22.3

	84	948.2	3.7
	85	949.4	4.7
	86	954.2	20.0
	87	967.3	9.3
	88	975.6	40.4
	89	984.2	28.0
	90	985.6	167.9
	91	1005.2	45.4
	92	1008.0	1.1
	93	1010.4	0.2
	94	1011.7	115.6
	95	1012.8	13.8
	96	1014.5	0.5
	97	1016.7	10.4
	98	1019.9	5.4
	99	1029.2	126.6
	100	1033.3	1.3
	101	1034.0	9.0
	102	1041.1	27.7
	103	1049.8	2.8
	104	1049.8	11.5
	105	1051.2	5.9
	106	1055.4	11.1
	107	1071.4	6.6
	108	1076.1	4.9
	109	1083.1	12.6
	110	1088.7	13.4

	111	1100.3	15.8
	112	1102.3	22.2
	113	1115.0	27.4
	114	1151.8	5.1
	115	1154.4	0.1
	116	1155.9	0.1
	117	1162.4	9.0
	118	1180.9	1.1
	119	1181.4	0.7
	120	1187.5	2.4
	121	1193.7	14.9
	122	1199.4	14.8
	123	1208.1	53.6
	124	1209.6	38.1
	125	1214.5	35.6
	126	1222.0	12.0
	127	1225.7	68.6
	128	1227.5	9.1
	129	1227.8	31.1
	130	1234.8	11.0
	131	1261.2	64.4
	132	1274.3	77.9
	133	1280.2	49.6
	134	1287.4	104.1
	135	1290.3	68.3
	136	1303.9	28.9
	137	1305.8	15.7

	138	1338.8	46.5
	139	1343.8	3.6
	140	1347.5	15.0
	141	1366.1	36.2
	142	1367.9	1.0
	143	1368.3	0.2
	144	1377.4	6.8
	145	1378.0	7.9
	146	1384.5	1.1
	147	1386.8	27.3
	148	1392.9	8.7
	149	1410.1	3.5
	150	1419.4	5.0
	151	1433.5	0.8
	152	1435.0	14.8
	153	1444.4	261.7
	154	1446.6	26.2
	155	1452.3	10.4
	156	1454.9	8.2
	157	1475.9	11.1
	158	1477.9	5.2
	159	1478.6	3.2
	160	1481.8	27.2
	161	1491.0	155.4
	162	1494.3	27.1
	163	1517.9	186.1
	164	1524.1	27.1

	165	1524.8	11.3
	166	1635.9	0.6
	167	1636.5	0.8
	168	1651.2	30.5
	169	1660.5	3.7
	170	1660.7	6.1
	171	1664.0	57.1
	172	1694.9	42.0
	173	1724.8	56.7
	174	2845.8	47.0
	175	2906.3	44.6
	176	3025.0	30.7
	177	3044.7	106.3
	178	3050.1	28.2
	179	3057.5	28.7
	180	3073.1	37.4
	181	3081.1	36.8
	182	3095.2	44.6
	183	3104.7	9.6
	184	3106.9	40.3
	185	3107.2	14.9
	186	3111.8	26.2
	187	3112.5	20.6
	188	3114.5	80.5
	189	3120.1	54.1
	190	3127.8	43.6
	191	3146.1	1.6

	192	3155.3	12.9
	193	3162.4	9.6
	194	3170.7	5.5
	195	3170.8	4.9
	196	3177.7	3.6
	197	3177.9	2.6
	198	3178.2	5.1
	199	3186.2	29.2
	200	3186.2	32.0
	201	3192.3	8.1
	202	3193.2	14.1
	203	3196.7	21.6
	204	3196.8	19.7
	205	3200.8	5.3
	206	3204.7	9.6
	207	3224.4	1.3
**TS 3**	1	323.2i	88.1
	2	13.8	0.0
	3	17.7	1.3
	4	24.2	0.4
	5	36.0	0.9
	6	41.4	0.4
	7	45.4	1.1
	8	63.6	0.1
	9	68.1	0.3
	10	74.9	0.0
	11	79.8	2.7

	12	85.9	1.0
	13	103.7	1.9
	14	107.6	0.2
	15	115.3	0.2
	16	121.7	0.4
	17	131.2	2.2
	18	142.6	8.2
	19	149.7	0.7
	20	161.2	4.6
	21	175.1	2.9
	22	185.3	2.1
	23	210.7	3.0
	24	220.8	3.0
	25	233.5	3.9
	26	253.3	15.1
	27	255.0	9.8
	28	270.6	2.7
	29	275.3	6.5
	30	300.3	4.4
	31	305.1	1.7
	32	320.6	3.0
	33	324.8	3.5
	34	372.8	30.4
	35	382.1	1.8
	36	386.7	4.6
	37	415.2	4.7
	38	424.4	5.4

	39	426.0	9.8
	40	434.5	1.8
	41	436.8	3.9
	42	439.1	1.1
	43	472.5	4.9
	44	480.6	4.7
	45	491.1	8.3
	46	502.0	0.3
	47	504.8	10.2
	48	508.3	7.0
	49	516.4	1.9
	50	548.8	2.3
	51	571.9	5.7
	52	580.4	24.8
	53	584.7	3.7
	54	610.2	20.3
	55	625.5	14.8
	56	634.0	0.3
	57	635.9	1.6
	58	645.1	72.7
	59	663.2	3.1
	60	732.5	53.5
	61	735.0	62.5
	62	735.5	25.5
	63	737.0	2.5
	64	758.2	2.8
	65	767.9	91.2

	66	770.4	3.3
	67	776.2	27.9
	68	779.5	0.8
	69	782.9	31.2
	70	803.2	40.1
	71	851.5	13.2
	72	852.6	24.8
	73	854.1	4.7
	74	854.7	16.8
	75	864.9	22.5
	76	870.0	5.3
	77	878.3	1.4
	78	881.1	0.9
	79	882.2	2.1
	80	890.1	2.1
	81	899.8	4.1
	82	938.0	99.6
	83	946.7	3.2
	84	949.1	5.3
	85	955.3	111.4
	86	959.7	7.9
	87	959.8	20.4
	88	974.2	5.3
	89	992.3	118.7
	90	1011.5	43.5
	91	1012.8	13.7
	92	1014.5	0.4

	93	1014.9	1.5
	94	1018.5	1.7
	95	1018.9	0.4
	96	1023.1	3.3
	97	1028.9	24.1
	98	1033.3	44.7
	99	1036.0	23.8
	100	1036.3	1.4
	101	1038.5	14.8
	102	1049.7	9.5
	103	1051.7	6.8
	104	1052.5	1.3
	105	1056.7	23.6
	106	1076.5	6.5
	107	1078.7	18.7
	108	1085.7	17.1
	109	1088.0	11.7
	110	1104.3	29.2
	111	1107.6	27.7
	112	1115.3	10.2
	113	1147.5	14.7
	114	1155.8	17.2
	115	1156.1	0.2
	116	1157.2	0.0
	117	1183.2	0.3
	118	1185.3	2.0
	119	1185.7	1.1

	120	1194.8	7.0
	121	1197.9	6.4
	122	1204.4	43.8
	123	1209.9	5.2
	124	1214.8	14.0
	125	1227.1	4.8
	126	1228.3	1.8
	127	1230.7	2.4
	128	1268.2	137.4
	129	1271.1	79.1
	130	1281.3	35.3
	131	1285.8	65.2
	132	1291.6	46.5
	133	1295.3	61.1
	134	1301.3	19.1
	135	1309.3	55.4
	136	1342.2	20.7
	137	1345.0	3.1
	138	1350.2	29.1
	139	1367.6	0.6
	140	1367.7	0.1
	141	1373.1	20.3
	142	1377.9	14.2
	143	1379.9	1.7
	144	1389.1	1.5
	145	1392.6	0.7
	146	1406.5	123.5

	147	1411.8	7.9
	148	1440.8	2.8
	149	1441.0	10.3
	150	1450.2	15.7
	151	1452.5	3.8
	152	1465.6	13.8
	153	1474.2	32.2
	154	1478.3	10.9
	155	1479.2	13.4
	156	1484.1	20.2
	157	1495.3	20.8
	158	1511.3	150.4
	159	1525.7	16.3
	160	1526.7	19.3
	161	1553.1	598.1
	162	1636.3	0.2
	163	1637.7	0.8
	164	1653.1	18.0
	165	1661.0	2.0
	166	1661.2	3.4
	167	1667.1	56.7
	168	1703.8	185.0
	169	1712.7	73.8
	170	2055.7	1036.2
	171	3034.5	24.1
	172	3051.4	88.4
	173	3069.3	25.0

	174	3073.5	21.5
	175	3085.5	4.0
	176	3098.5	18.0
	177	3104.1	11.2
	178	3115.3	19.8
	179	3118.7	19.3
	180	3120.4	24.3
	181	3126.4	26.0
	182	3126.7	13.1
	183	3141.8	3.3
	184	3147.3	1.6
	185	3171.5	18.3
	186	3173.0	4.3
	187	3174.7	3.7
	188	3177.9	1.1
	189	3179.8	0.9
	190	3181.2	3.1
	191	3183.0	0.6
	192	3185.8	14.3
	193	3187.9	14.6
	194	3188.4	6.4
	195	3189.3	8.2
	196	3191.7	29.2
	197	3194.3	28.4
	198	3199.6	10.7
	199	3201.0	7.7
	200	3201.7	15.6

	201	3207.9	6.0
**TS 4**	1	450.0i	132.5
	2	17.1	0.5
	3	19.0	0.7
	4	20.9	0.6
	5	31.1	0.1
	6	33.9	1.0
	7	35.4	0.4
	8	36.4	0.3
	9	55.1	2.6
	10	58.9	0.6
	11	66.4	0.6
	12	72.7	0.2
	13	76.3	1.4
	14	80.3	0.1
	15	89.5	1.2
	16	103.3	0.8
	17	113.2	1.6
	18	121.2	1.2
	19	135.1	4.7
	20	147.4	4.0
	21	152.4	1.6
	22	162.2	1.9
	23	185.5	2.6
	24	201.6	2.6
	25	223.8	2.6
	26	230.9	6.4

	27	234.2	5.4
	28	247.7	1.5
	29	263.5	4.2
	30	265.4	3.1
	31	268.9	1.6
	32	291.2	2.6
	33	294.4	1.8
	34	304.7	5.1
	35	318.0	0.1
	36	337.7	4.4
	37	350.5	9.8
	38	369.0	2.4
	39	378.7	2.7
	40	383.6	19.3
	41	410.3	0.8
	42	427.1	0.3
	43	431.4	2.3
	44	432.9	5.1
	45	436.7	9.7
	46	455.3	7.7
	47	480.5	7.8
	48	487.4	4.1
	49	497.0	6.7
	50	497.5	8.7
	51	502.5	3.7
	52	508.7	1.2
	53	540.4	9.2

	54	557.8	4.4
	55	580.6	20.6
	56	602.7	12.9
	57	618.4	15.8
	58	624.6	21.2
	59	634.1	3.1
	60	635.3	0.3
	61	642.7	66.8
	62	666.1	28.2
	63	727.9	28.2
	64	729.8	61.7
	65	734.1	58.9
	66	736.8	48.0
	67	751.8	12.9
	68	755.8	3.6
	69	763.0	70.7
	70	765.4	0.9
	71	773.7	28.1
	72	774.9	29.5
	73	778.2	13.0
	74	809.0	25.2
	75	848.9	6.6
	76	851.7	4.2
	77	853.1	9.0
	78	856.3	8.2
	79	860.7	5.4
	80	867.8	0.1

	81	875.3	2.5
	82	875.5	3.8
	83	879.6	1.3
	84	890.1	124.6
	85	898.5	2.6
	86	930.9	39.7
	87	938.1	3.2
	88	946.5	10.5
	89	952.2	16.4
	90	954.7	14.5
	91	960.3	21.9
	92	961.9	26.1
	93	963.0	147.9
	94	985.1	45.7
	95	1001.5	0.4
	96	1007.1	66.3
	97	1013.4	1.8
	98	1014.6	73.0
	99	1015.4	6.3
	100	1016.1	7.1
	101	1018.4	2.7
	102	1023.3	26.8
	103	1032.4	87.0
	104	1034.5	2.6
	105	1035.9	4.2
	106	1040.4	7.5
	107	1044.4	202.6

	108	1049.8	14.4
	109	1050.1	4.0
	110	1052.9	11.3
	111	1057.4	6.8
	112	1080.8	4.9
	113	1084.6	13.6
	114	1089.2	16.4
	115	1102.5	23.6
	116	1104.4	22.0
	117	1114.0	37.9
	118	1131.9	6.4
	119	1151.1	1.5
	120	1155.5	0.1
	121	1156.4	0.0
	122	1173.6	14.1
	123	1181.4	0.6
	124	1184.5	0.6
	125	1196.0	3.2
	126	1198.2	5.5
	127	1201.8	9.4
	128	1211.3	16.2
	129	1215.6	18.1
	130	1217.8	57.2
	131	1227.1	2.8
	132	1228.0	3.4
	133	1246.6	179.1
	134	1249.3	26.6

	135	1251.1	47.3
	136	1281.7	42.4
	137	1285.0	120.8
	138	1287.2	75.9
	139	1290.9	27.4
	140	1302.1	37.3
	141	1305.3	15.1
	142	1323.0	4.3
	143	1341.2	37.5
	144	1346.0	7.1
	145	1349.0	15.6
	146	1367.4	8.2
	147	1368.4	0.2
	148	1369.8	26.3
	149	1378.5	0.2
	150	1381.8	12.0
	151	1386.6	10.0
	152	1392.1	78.2
	153	1398.1	2.7
	154	1407.1	27.4
	155	1413.5	5.4
	156	1421.6	12.6
	157	1437.1	10.4
	158	1439.3	7.8
	159	1453.5	13.2
	160	1454.5	5.3
	161	1465.5	85.1

	162	1478.0	6.6
	163	1478.8	2.0
	164	1479.9	34.0
	165	1487.9	36.8
	166	1495.3	1.4
	167	1517.2	569.3
	168	1521.3	185.7
	169	1525.1	33.1
	170	1525.9	29.7
	171	1616.3	670.5
	172	1636.4	0.7
	173	1636.4	1.4
	174	1651.7	32.7
	175	1661.0	4.6
	176	1661.2	5.0
	177	1664.2	53.3
	178	1693.3	54.3
	179	1711.7	8.0
	180	3014.3	19.6
	181	3019.2	31.7
	182	3026.1	53.5
	183	3055.4	32.2
	184	3055.5	26.1
	185	3057.8	101.5
	186	3069.7	39.2
	187	3084.3	19.4
	188	3087.9	30.0

	189	3093.7	47.7
	190	3106.7	16.6
	191	3109.7	39.2
	192	3111.5	21.8
	193	3112.7	26.3
	194	3113.0	27.1
	195	3118.7	54.4
	196	3125.6	20.6
	197	3145.2	11.7
	198	3162.8	9.6
	199	3165.7	7.6
	200	3170.5	5.0
	201	3171.8	5.3
	202	3174.5	6.5
	203	3177.8	2.3
	204	3177.8	4.5
	205	3177.9	2.7
	206	3186.1	27.8
	207	3186.6	30.1
	208	3192.4	14.0
	209	3196.7	22.0
	210	3197.5	19.3
	211	3201.7	15.2
	212	3206.3	4.8
	213	3225.0	0.8
